# Dimensionality reduction for visualizing spatially resolved profiling data using SpaSNE

**DOI:** 10.1093/gigascience/giaf002

**Published:** 2025-02-17

**Authors:** Yuansheng Zhou, Chen Tang, Xue Xiao, Xiaowei Zhan, Tao Wang, Guanghua Xiao, Lin Xu

**Affiliations:** Quantitative Biomedical Research Center, Peter O'Donnell Jr. School of Public Health, University of Texas Southwestern Medical Center, Dallas, TX 75390, USA; Quantitative Biomedical Research Center, Peter O'Donnell Jr. School of Public Health, University of Texas Southwestern Medical Center, Dallas, TX 75390, USA; Quantitative Biomedical Research Center, Peter O'Donnell Jr. School of Public Health, University of Texas Southwestern Medical Center, Dallas, TX 75390, USA; Quantitative Biomedical Research Center, Peter O'Donnell Jr. School of Public Health, University of Texas Southwestern Medical Center, Dallas, TX 75390, USA; Center for the Genetics of Host Defense, University of Texas Southwestern Medical Center, Dallas, TX 75390, USA; Quantitative Biomedical Research Center, Peter O'Donnell Jr. School of Public Health, University of Texas Southwestern Medical Center, Dallas, TX 75390, USA; Center for the Genetics of Host Defense, University of Texas Southwestern Medical Center, Dallas, TX 75390, USA; Quantitative Biomedical Research Center, Peter O'Donnell Jr. School of Public Health, University of Texas Southwestern Medical Center, Dallas, TX 75390, USA; Department of Bioinformatics, University of Texas Southwestern Medical Center, Dallas, TX 75390, USA; Quantitative Biomedical Research Center, Peter O'Donnell Jr. School of Public Health, University of Texas Southwestern Medical Center, Dallas, TX 75390, USA; Department of Pediatrics, Division of Hematology/Oncology, University of Texas Southwestern Medical Center, Dallas, TX 75390, USA

**Keywords:** spatially resolved omics, dimensionality reduction, low-dimensional visualization, molecular data structure, spatial organization of cells

## Abstract

**Background:**

Spatially resolved profiling technologies to quantify transcriptomes, epigenomes, and proteomes have been emerging as groundbreaking methods for comprehensive molecular characterizations. Dimensionality reduction and visualization is an essential step to analyze and interpret spatially resolved profiling data. However, state-of-the-art dimensionality reduction methods for single-cell sequencing data, such as the t-distributed stochastic neighbor embedding (t-SNE) and uniform manifold approximation and projection (UMAP), were not tailored for spatially resolved profiling data.

**Results:**

Here we developed a spatially resolved t-SNE (SpaSNE) method to integrate both spatial and molecular information. We applied it to a variety of public spatially resolved profiling datasets that were generated from 3 experimental platforms and consisted of cells from different diseases, tissues, and cell types. To compare the performances of SpaSNE, t-SNE, and UMAP, we applied them to 4 spatially resolved profiling datasets obtained from 3 distinct experimental platforms (Visium, STARmap, and MERFISH) on both diseased and normal tissues. Comparisons between SpaSNE and these state-of-the-art approaches reveal that SpaSNE achieves more accurate and meaningful visualization that better elucidates the underlying spatial and molecular data structures.

**Conclusions:**

This work demonstrates the broad application of SpaSNE for reliable and robust interpretation of cell types based on both molecular and spatial information, which can set the foundation for many subsequent analysis steps, such as differential gene expression and trajectory or pseudotime analysis on the spatially resolved profiling data.

## Background

Due to the capability to uncover spatial organization and intercellular communication, spatially resolved profiling technologies on DNA, RNA, and proteins have become one of the latest frontiers for cutting-edge research in both basic biology and medicine. While a large number of distinct spatial profiling platforms have been developed so far, a recent review [1] proposed that spatially resolved profiling technologies can be primarily categorized into 2 major directions: imaging-based approaches (e.g., STARMap [2] and seqFISH [3]) and next-generation sequencing (NGS)–based approaches (e.g., Slide-seq [4] and Visium by 10X Genomics). These innovative technologies are promising to transform the way that we think about cell differentiation, tissue development, and disease progression in a spatial fashion and therefore could lead to novel discoveries on elucidating detailed cellular and molecular mechanisms, as well as identifying effective biomarkers and therapeutic targets [1, 5, 6].

Dimensionality reduction and visualization is an essential step to analyze and interpret the spatially resolved profiling data from DNA, RNA, and proteins [7, 8]. Different from the clustering methods (e.g., BayesSpace [9] or SpaGCN [10]), the aim of developing dimensionality reduction and visualization approaches for spatially resolved profiling data is to visualize the cells in a low-dimensional space while maintaining the underlying molecular and spatial data structures (e.g., gene expression variabilities of different cell types [11, 12] and spatial closeness of various cell types [13, 14]). Among the published methods, the t-distributed stochastic neighbor embedding (t-SNE) [15–17] and the uniform manifold approximation and projection (UMAP) [18] have been the most widely used tools for dimensionality reduction and visualization of single-cell sequencing data. Compared with the linear dimensionality reduction methods such as principal component analysis (PCA), both t-SNE and UMAP have great advantages in reliably visualizing cell clusters in single-cell sequencing datasets [15–20]. Some recent variants of t-SNE and UMAP further extended the power of these 2 algorithms in revealing gene expression variabilities of single cells [12] or visualizing multimodal omics data [21]. Therefore, recent spatially resolved profiling studies have been using either t-SNE [22–28] or UMAP [2, 13, 14, 29–32] for data visualization. However, different from routine single-cell omics data with molecular information alone, the most unique feature of spatially resolved profiling data is that it contains both molecular information from NGS and spatial organization information from images. The current design of t-SNE or UMAP does not leverage both molecular and spatial information simultaneously for analyzing spatially resolved profiling data. Therefore, new dimensionality reduction and visualization algorithms that are able to integrate both molecular and spatial information are in urgent need, because they can visualize cell clusters in the context of tissues’ spatial organization and are promising to help uncover more biological insights in the studies of cellular communications [13, 22–24, 33–35] or developmental trajectories [14, 29–31, 36] in the spatial fashion.

Here we developed a spatially resolved t-SNE (SpaSNE) method by adapting t-SNE to more adequately leverage both molecular and spatial information in the spatially resolved profiling data. SpaSNE could provide a comprehensive low-dimensional visualization that better preserves the molecular data structure and spatial organization of cells simultaneously. Because spatially resolved gene expression profiling technologies are well developed so far, in this study, we will mainly use spatially resolved gene expression datasets to demonstrate the utility of SpaSNE. To compare the performances of SpaSNE, t-SNE, and UMAP, we applied them to 4 spatially resolved profiling datasets obtained from 3 distinct experimental platforms (Visium, STARmap, and MERFISH) on both diseased and normal tissues. The analytical results showed that SpaSNE achieves the most accurate embedding and most meaningful visualization of the spatially resolved profiling data.

## Methods

### Preprocessing of data

The 4 spatial gene expression datasets used in this article are presented in Supplementary Table S1 (the details are also included in the “Data Availability” section). For the human breast cancer dataset, there are a total of 2,518 spots in the image, but only 1,272 of them were annotated by a pathologist, and the rest of the spots cannot be determined. We selected these 1,272 spots to evaluate the performance of our algorithm based on the ground-truth annotation. For the mouse hypothalamus dataset, we took the left side of the whole slide, which contains 2,693 cells. The annotations of the 4 datasets are provided in Supplementary Tables S2–S5. Given a unique molecular identifier (UMI) count matrix, we first used the “scanpy” Python package to normalize the counts. Each cell or spot has a total count equal to the median of total counts per cell. We then transformed them to a natural log scale. For the human breast cancer, human prostate cancer, and mouse visual cortex datasets, we reduced the dimensionality to 200 principal components before performing the embedding. For the mouse hypothalamus dataset, we used all 161 genes without dimensionality reduction.

### Data annotations

The annotations of the human breast cancer, mouse visual cortex, and mouse hypothalamus datasets were obtained from original papers or websites (the details are included in the “Data Availability” section). The human prostate cancer dataset was annotated by the HD-Staining [37] algorithm developed for classifying cell nuclei and cell types in the pathology images. The annotations of the 4 datasets were provided in the metadata files in Supplementary Tables S2–S5. The metadata include the spatial positions and different kinds of cell-type labels for each cell/spot.

### Differential expression analysis

The differential expression analysis results in Supplementary Figs. S2–S4 and S7–S12 were obtained by using the scanpy toolkit, and the Wilcoxon method was used for human breast cancer and prostate cancer data to generate the results in Supplementary Figs. S2–S4 and  S7–S8. The “*t*-test” method was used for mouse visual cortex and hypothalamus to generate the results in Supplementary Figs. S9–S12.

### SpaSNE’s embedding

The t-SNE algorithm [16] has been widely used in nonlinear dimensionality reduction and visualization for gene expression data. Given a dataset with *N* spots or cells with gene expression vectors $( {{x}_1,{x}_2,\ldots,\ {x}_N} ),$, t-SNE defines the pairwise similarities of data points ${p}_{ij}\ $ by the following form:


(1) \begin{eqnarray*}
{p}_{j|i} = \frac{{{\mathrm{exp}}\left( { - \|{x}_i - {x}_j{\|}^2/2\sigma _i^2} \right)\ }}{{\mathop \sum \nolimits_{i \neq j} {\mathrm{exp}}\left( { - \|{x}_i - {x}_j{\|}^2/2\sigma _i^2} \right)}}
\end{eqnarray*}

(2) \begin{eqnarray*}
{p}_{ij} = \frac{{{p}_{i|j} + {p}_{j|i}}}{{2N}}
\end{eqnarray*}

where ${\sigma }_{i\ }$ is the variance of the Gaussian distribution that centers on ${x}_j.$ In the low-dimensional ($d = 2\ or\ 3$) representation, the pairwise similarities of points $( {{y}_1,{y}_2,\ldots,\ {y}_N} )$ are defined as


(3) \begin{eqnarray*}
{q}_{ij} = \frac{{{{\left( {1 + \|{y}_i - {y}_j{\|}^2} \right)}}^{ - 1}}}{{\mathop \sum \nolimits_{i \neq j} {{\left( {1 + \|{y}_i - {y}_j{\|}^2} \right)}}^{ - 1}}}
\end{eqnarray*}

The loss function ${L}_t\ $ is defined as the discrepancy between data and embedding points, which is measured by the Kullback–Leibler (KL) divergence of the pairwise similarities:


(4) \begin{eqnarray*}
{L}_t = KL(P|{\mathrm{|}}Q{\mathrm{)}} = \ \mathop \sum \limits_i \mathop \sum \limits_j {p}_{ij}\log \left( {\frac{{{p}_{ij}}}{{{q}_{ij}}}} \right)
\end{eqnarray*}

The loss function $\ {L}_t$ is minimized to achieve the optimal low-dimensional representation of the data. The gradient of the loss function ${L}_t$ with respect to ${y}_i$ is calculated as


(5) \begin{eqnarray*}
\frac{{\partial {L}_t}}{{\partial {y}_i}} = 4\mathop \sum \limits_j \left( {{p}_{ij} - {q}_{ij}} \right)\left( {{y}_i - {y}_j} \right){\left( {1 + \|{y}_i - {y}_j{\|}^2} \right)}^{ - 1}
\end{eqnarray*}

With this definition, t-SNE only preserves the local structure of the gene expression because equations (1) and (3) are only sensitive to small-scale distance variations.

The spatially resolved profiling data provide the spatial positions of spots or cells $( {{z}_1,{z}_2,\ldots,{z}_N} )$, which cannot be used in t-SNE. SpaSNE improves t-SNE by introducing 2 new loss functions to preserve both the large-scale gene expression distances and the spatial distances of data. The first loss function measures the KL divergence ${L}_g$ between the large-scale gene expression distances ${\hat{p}}_{ij}\ $ and the large-scale embedding distances $\ {\hat{q}}_{ij}$:


(6) \begin{eqnarray*}
{\hat{p}}_{ij} = \frac{{1 + \|{x}_i - {x}_j{\|}^2}}{{\mathop \sum \nolimits_{i \neq j} \left( {1 + \|{x}_i - {x}_j{\|}^2} \right)}}
\end{eqnarray*}

(7) \begin{eqnarray*}
\ {\hat{q}}_{ij} = \frac{{1 + \|{y}_i - {y}_j{\|}^2}}{{\mathop \sum \nolimits_{i \neq j} \left( {1 + \|{y}_i - {y}_j{\|}^2} \right)}}
\end{eqnarray*}

(8) \begin{eqnarray*}
{L}_g = KL(\hat{P}|{\mathrm{|}}\hat{Q}{\mathrm{)}} = \ \mathop \sum \limits_i \mathop \sum \limits_j {\hat{p}}_{ij}\log \left( {\frac{{{{\hat{p}}}_{ij}}}{{\ {{\hat{q}}}_{ij}}}} \right)
\end{eqnarray*}

Introducing ${L}_g$ helps preserve large-scale intercluster structure of gene expression because equations (6) and (7) are sensitive to large-scale distance variations. The second loss function measures the KL divergence ${L}_s\ $ between the large-scale spatial distances $\ {\hat{s}}_{ij}\ $ from image and the large-scale embedding distances $\ {\hat{q}}_{ij}$:


(9) \begin{eqnarray*}
\ {\hat{s}}_{ij} = \frac{{1 + \|{z}_i - {z}_j{\|}^2}}{{\mathop \sum \nolimits_{i \neq j} \left( {1 + \|{z}_i - {z}_j{\|}^2} \right)}}
\end{eqnarray*}

(10) \begin{eqnarray*}
{L}_s = KL(\hat{S}|{\mathrm{|}}\hat{Q}{\mathrm{)}} = \ \mathop \sum \limits_i \mathop \sum \limits_j \ {\hat{s}}_{ij}\log \left( {\frac{{\ {{\hat{s}}}_{ij}}}{{\ {{\hat{q}}}_{ij}}}} \right)
\end{eqnarray*}

Integrating the 2 new loss functions to the original loss function ${L}_t$ of t-SNE, we get the total loss function with 2 weighting parameters $\alpha $ and $\beta $:


(11) \begin{eqnarray*}
{L}_{\textit{total}} = \ {L}_t + {\mathrm{\ }}\alpha {L}_g{\mathrm{\ }} + {\mathrm{\ }}\beta {L}_s
\end{eqnarray*}

The gradient of the loss function ${L}_{\textit{total}}$ has a simple form:


(12) \begin{eqnarray*}
\frac{{\partial {L}_t}}{{\partial {y}_i}} = 4\mathop \sum \limits_j \left[ {\left( {{p}_{ij} - {q}_{ij}} \right) - \alpha \left( {{{\hat{p}}}_{ij} - \ {{\hat{q}}}_{ij}} \right) - \beta \left( {\ {{\hat{s}}}_{ij} - \ {{\hat{q}}}_{ij}} \right)} \right]\left( {{y}_i - {y}_j} \right){\left( {1 + \|{y}_i - {y}_j{\|}^2} \right)}^{ - 1}\\
\end{eqnarray*}

### Quantitative evaluation of the embedding quality

Three quantitative measures were defined to evaluate the embedding quality: (i) Pearson correlation coefficient (${r}_g$) between the pairwise Euclidean distances of the gene expressions and the embedding distances of points, and this metric is equivalent to the Shephard diagram [38], which was usually used to measure the goodness of fit by low-dimensional visualization algorithms [18, 39–41]; (ii) Pearson correlation coefficient (${r}_s$) between pairwise spatial distances and embedding distances of points, which was used to measure the preservation of the spatial structure; and (iii) silhouette score (*s*), which was used to measure the goodness of clustering [41]. This metric was widely used as an evaluation tool for clustering quality analysis [40, 42–44]. When using the ground-truth annotation as the predicted cluster labels, it measures the consistency between the clusters of embedding points and the ground-truth annotation of points. An alternative method of evaluating the embedding quality is the “trustworthiness” metric, which measures the preservation of the local structure of data [45]. For spatially resolved data, both the local structure of spatial positions and gene expressions should be considered. The design of SpaSNE leads to increased trustworthiness of spatial structure and decreased trustworthiness of transcriptomic structure compared with t-SNE. However, SpaSNE achieves a higher value of the product of the 2 scores than t-SNE (Supplementary Fig. S15). The metric of the product of the 2 trustworthiness scores gives similar results as the silhouette score, and it might be used as an alternative metric to replace the silhouette score when the ground-truth annotation is not available. In our analysis, ${r}_g$ and ${r}_s$ were calculated using the “scipy” python package, and silhouette score and trustworthiness were calculated using the “sklearn” python package.

### Parameters of visualization algorithms

We applied 3 algorithms for visualization of the spatially resolved gene expression profiling data: t-SNE, UMAP, and SpaSNE. We ran UMAP using the “umap” Python package [18] with default parameters.

For SpaSNE, we screened the combination of parameters $\alpha $ and $\beta $ on the 4 datasets and showed how the parameters influence gene expression preservation (${r}_g$), spatial structure preservation (${r}_s$), and the stability of the embeddings. The 2 parameters $\alpha $ and $\beta $ in equation (11) represent the weights of the large-scale gene expression loss function ${L}_g$ and the spatial loss function ${L}_s$. Therefore, a larger $\alpha $ leads to a larger ${r}_g$ and a smaller ${r}_s$, and a larger $\beta $ leads to a larger ${r}_s$ and a smaller ${r}_g$. In addition to the ratio of $\alpha \ $ and $\beta $, the magnitude of $\alpha \ $ and $\beta $ may also influence the stability of the embedding because the contribution of the local cost function ${L}_t\ $ in the original t-SNE will be weakened by a large $\alpha $ and $\beta $ (equation (11)), and the embedding will become more unstable, especially when the data size is small. We measured stability by the standard deviation $( {std} )$ of ${r}_g$ in multiple repeated embeddings of SpaSNE with a given set of parameters (Supplementary Fig. S16b, d, f, h). Smaller $std\ $ is preferred when selecting the parameters.

To determine the optimal combination of parameters for a given data, we developed a heuristic screening approach that consists of 2 stages: rough screening and fine screening. In rough screening, we screened the 2 parameters on a larger scale to determine the range where the optimal parameters may fall (Supplementary Fig. S16a, c, e, g). In fine screening, we determined the optimal parameter with a finer resolution (Supplementary Fig. S16b, d, f, h). Here we use the example of the human breast cancer dataset to demonstrate the 2-stage screening process in detail:

1. Running 100 repeats of t-SNE with default parameters on human breast cancer datasets and calculating (${r}_g$, ${r}_s$) for each repeat. The maximal value of ${r}_g$ is marked as ${r}_{\textit{thres}}$.
2. Performing rough screening with SpaSNE.
  2.1. Taking $\alpha {\mathrm{\ }}$ and $\beta $ from ${\mathrm{\{ }}( {\alpha ,\beta } ){\mathrm{|\ }}\alpha \in [ {2,{\mathrm{\ }}5,{\mathrm{\ }}10,{\mathrm{\ }}20,{\mathrm{\ }}30,50} ],{\mathrm{\ }}\beta \in {\mathrm{\ }}[ {1,{\mathrm{\ }}5,{\mathrm{\ }}10,{\mathrm{\ }}15,{\mathrm{\ }}25} ]\} .$
  2.2. In each parameter combination, running 10 repeats of SpaSNE, setting ${r}_g = 0$ if ${r}_g \le {r}_{\textit{thres}}$ in each repeat, calculating (${r}_g$, ${r}_s$) for each repeat, and selecting the optimal embedding that gives a maximal value of ${r}_g \times {r}_s$ in the 10 repeats and recording the optimal $r_g^{opt}$ and $r_s^{opt}$.
  2.3. Showing the values of $r_g^{opt} \times r_s^{opt}$ for all the parameter combinations by heatmap (Supplementary Fig. S16a).
3. Performing fine screening with SpaSNE.
  3.1. Based on the heatmap results in step 2.3, selecting the range where the optimal parameters may fall: ${\mathrm{\{ }}( {\alpha ,\beta } ){\mathrm{|}}\ \alpha \in [ {5,\ 6,\ 7,..,\ 20} ],\ \beta \in \ [ {1,\ 2,\ 3,\ldots,\ 10} ]\} $.
  3.2. In each parameter combination, running 20 repeats of SpaSNE, setting ${r}_g = 0$ if ${r}_g \le {r}_{\textit{thres}}$ in each repeat, calculating (${r}_g$, ${r}_s$) for each repeat and standard deviation ($std$) of ${r}_g$ of the 20 repeats, and selecting the optimal embedding that gives a maximal value of ${r}_g \times {r}_s$ in the 20 repeats and recording the optimal values $r_g^{opt}$, $r_s^{opt}$, and $std$.
  3.3. Showing the values of $r_g^{opt} \times r_s^{opt}$ (left), $std$ (middle), and $r_g^{opt} \times r_s^{opt} \times \ {\mathrm{exp}} ( {1 - std} )$ (right) for all the parameter combinations by heatmap (Supplementary Fig. S16b).
4. Determining the optimal parameter combination by selecting the maximal value of $r_g^{opt} \times r_s^{opt} \times \ {\mathrm{exp}} ( {1 - std} )$ obtained in step 3.3.
5. Running 100 repeats of SpaSNE with the optimal parameter combination obtained in step 4 and selecting the embedding with the maximal values of ${r}_g \times {r}_s$.

The results of the 4 datasets show that the optimal parameters depend on both the size and the type of the data. For example, both the human breast cancer and prostate cancer datasets are generated from the 10X Visium platform, and the optimal parameters are larger for the dataset with a larger size (comparing the human prostate cancer dataset with *N* = 4,371, $\alpha $ = 30, to the human breast cancer dataset with *N* = 1,272, $\alpha $ = 9) (Supplementary Fig. S16b, d). However, the mouse hypothalamus MERFISH dataset has a larger size than the mouse visual cortex dataset but smaller optimal parameters (comparing the mouse visual cortex dataset with *N* = 1,207, $\alpha $ = 14, to mouse hypothalamus dataset with *N* = 2,693, $\alpha $ = 10) (Supplementary Fig. S16b, h). Despite the complex dependence of parameters on the data types, our heuristic 2-stage screening approach works for all 4 diverse datasets and can hopefully be applied to other types of data. The default parameters for SpaSNE were set as $\alpha = 10,{\mathrm{\ }}\beta = 5$ if spatial information is available and $\alpha = 5,{\mathrm{\ }}\beta = 0$ if the spatial information is not available. The ranges used in tough and fine screenings and the optimal parameters for the 4 datasets can be found in Supplementary Table S1.

All 3 algorithms were initialized with the default setting: UMAP was initialized using a spectral embedding of the fuzzy 1-skeleton, and both t-SNE and SpaSNE were initialized from a truncated eigenvector matrix with the dimension of 50. The stopping criterion for SpaSNE is the same as t-SNE, which stops when the maximal iteration (1,000 by default) is reached. The perplexity values in SpaSNE and t-SNE were set as the default value, which is 50. Increasing perplexity will improve the global structure preservation (${r}_g$) in SpaSNE, but this parameter does not influence the embedding of SpaSNE as much as that of t-SNE (Supplementary Fig. S17). The reason is that perplexity determines the number of neighbors in local structure preservation, while SpaSNE embedding largely depends on the 2 added parameters $\alpha $ and $\beta $, which preserves the global gene expression structure and spatial structure. The perplexity’s influence becomes weaker as $\alpha $ and $\beta $ grow larger (comparing human breast cancer dataset with $\alpha = 9\ $ to human prostate cancer dataset with $\alpha = 30$) (Supplementary Fig. S17a, b).

### Computational complexity of SpaSNE, t-SNE, and UMAP

The computational complexity of t-SNE (implemented by Barnes-Hut-SNE [46]) is ${\mathrm O}( {\textit{NlogN}} ).{\mathrm{\ }}$ The computational complexity of UMAP is empirically ${\mathrm O}( {{N}^{1.14}} )$ [47]. The computational cost of SpaSNE consists of 3 parts: the local loss of gene expression ${{\mathrm{L}}}_{\mathrm{t}}$, global loss of gene expression ${{\mathrm{L}}}_{\mathrm{g}}$, and global loss of spatial positions ${{\mathrm{L}}}_{\mathrm{s}}$ (equation (11)). By applying the vantage-point trees approximation used in Barnes-Hut-SNE, the cost of ${{\mathrm{L}}}_{\mathrm{t}}$ can be reduced from ${\mathrm O}( {{N}^2} )$ to ${\mathrm O}( {\textit{NlogN}} ).{\mathrm{\ }}$ However, the global loss ${L}_g{\mathrm{\ }}$ and ${L}_s$ cannot be approximated by the local structure–based strategy in Barnes-Hut-SNE or the nearest-neighbor-descent algorithm [48] used in UMAP. Thus, the computational cost in the current form of SpaSNE is ${\mathrm O}( {{N}^2} ).$ The running time of SpaSNE on a MacBook Pro with a 2-GHz Quad-Core Intel Core i5 processer and 16-GB 3733 MHz LPDDR4X memory varies from 18 seconds for the human breast cancer dataset with 1,272 spots to 3 minutes for the human prostate cancer dataset with 4,371 spots. One possible approach to reducing computational time for large datasets is to mimic the strategy in the SpaceFlow algorithm [49] algorithm to use a fixed number of randomly selected edges to approximate the pairwise distance calculation in global terms ${\hat{p}}_{ij},$  ${\hat{q}}_{ij}{\mathrm{\ }}$, and ${\hat{s}}_{ij}$ (equations (6)–(9)). In this way, the ${\mathrm O}( {{N}^2} )$ in global loss will be constant, and the total cost will become ${\mathrm O}( {\textit{NlogN}} ).$

## Results

### Overview of SpaSNE

**Figure 1:**
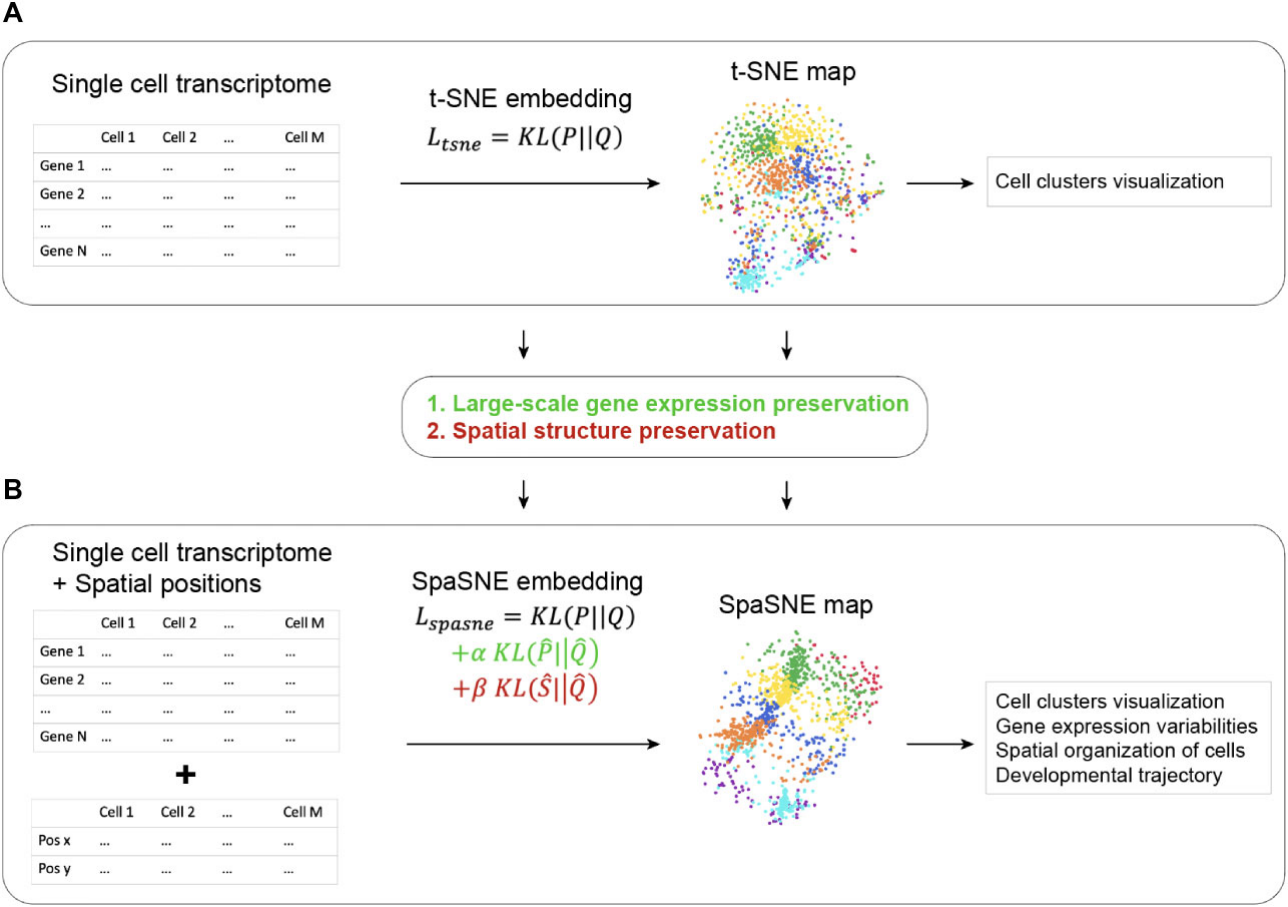
Workflow of t-SNE and SpaSNE methods. (A) Workflow of single-cell transcriptomic data analysis. (B) Workflow of spatially resolved transcriptomic data analysis. SpaSNE adapts t-SNE by introducing 2 parameters, $\alpha $ and $\beta $, to better preserve large-scale gene expression distances and spatial structure. The dataset used for visualization is mouse visual cortex STRAmap data^2^.

As one of the most widely used dimensionality reduction tools for single-cell sequencing data analysis, t-SNE has recently been adopted to analyze spatially resolved gene expression profiling data. It takes the gene expression data as input and performs dimensionality reduction and visualization for the data. The primary purpose of t-SNE is to preserve the small-scale local structure of gene expression (i.e., cell clustering) by minimizing the loss function ${{\boldsymbol{L}}}_{{\boldsymbol{tsne}}},{\boldsymbol{\ }}$ which is the KL divergence between similarities of data points and embedding points. Therefore, the t-SNE map was mainly used to generate a low-dimensional visualization map that reliably displays the clustering of cells (Fig. 1A). SpaSNE extends the function of t-SNE by not only preserving the local structure of gene expression but also maintaining the large-scale intercluster structure of gene expression and integrating spatial information of the cells. SpaSNE takes both gene expression data and spatial positions as input. It introduces 2 new loss functions to preserve large-scale gene expression distances and spatial distances, respectively (see Methods). The contributions of these 2 loss functions are controlled by 2 independent parameters ${\boldsymbol{\alpha }}$ and ${\boldsymbol{\beta }}$, which can be adjusted by users to balance gene expression preservation and spatial structure preservation. With this adaptation, SpaSNE can generate a low-dimensional visualization map that not only displays the clustering of cells as t-SNE does but also reveals intercluster features in spatially resolved expression profiling data, including gene expression variabilities of different cell clusters, spatial organization of cell types, and developmental trajectory of tissues (Fig. 1B). We will provide detailed examples to show all these applications of SpaSNE in the following sections. Because spatially resolved epigenomics and proteomics profiling technologies are now under development, here we focused on using available spatially resolved gene expression profiling datasets to demonstrate the utility of SpaSNE.

### Application to the diseased breast tissue data based on the Visium spatial transcriptomics technology

**Figure 2:**
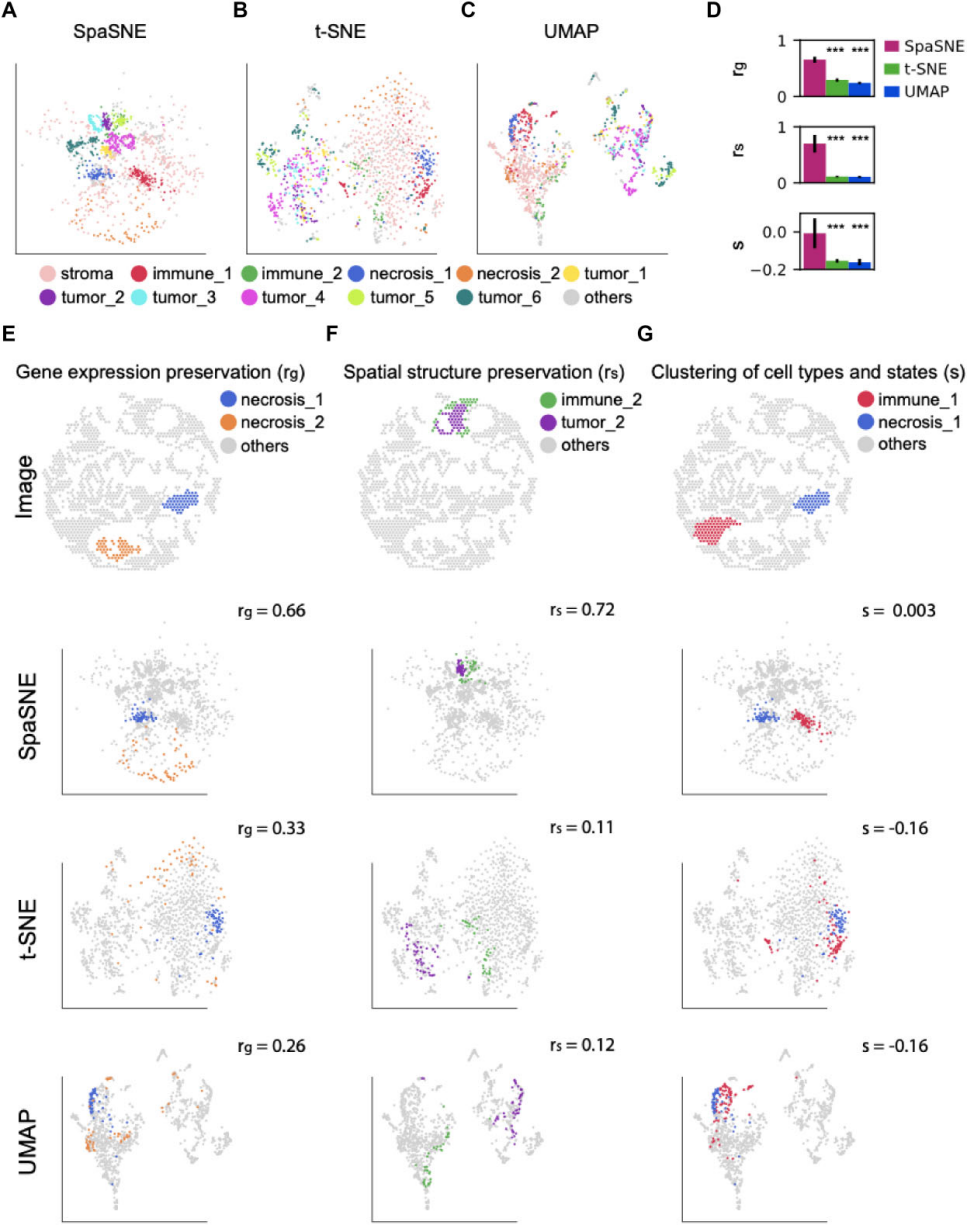
Visualizations of human breast cancer data. (A–C) Two-dimensional visualization of cells colored according to ground-truth labels from pathologist annotations in (A) SpaSNE, (B) t-SNE, and (C) UMAP embeddings. Tumor, immune, and other regions that have different spatial locations are labeled by different numbers. (D) Using 3 quantitative measures to evaluate SpaSNE, t-SNE, and UMAP embeddings: Pearson correlation coefficient between embedding distances and gene expression distances (${r}_g$), Pearson correlation coefficient between embedding distances and spatial distances (${r}_s$), and Silhouette score of embedding (*s*). The error bars show a 95% confidence interval of 100 embedding repeats. The asterisks above the bar plots represent *P* values of a 2-sided *t*-test between results of SpaSNE and t-SNE/UMAP: ***P < 0.001. (E–G) Visualization of cells in raw image, SpaSNE, t-SNE, and UMAP embeddings by highlighting different pairs of cell states: (E) necrosis_1 (blue) and necrosis_2 (orange), (F) immune_2 (green) and tumor_2 (purple), and (G) immune_1 (red) and necrosis_1 (blue).

We first analyzed spatially resolved transcriptomics data of the human breast cancer tissues from the 10X Genomics data portal (Supplementary Fig. S1a). We extracted 1,272 annotated spots from the original dataset and performed SpaSNE, t-SNE, and UMAP embeddings to compare their performances on visualization of cells. We showed 10 cell clusters, which were colored according to both cell types (based on pathological annotation from 10X Genomics data portal) and spatial locations in SpaSNE, t-SNE, and UMAP embeddings, respectively (Fig. 2A–C). SpaSNE presented separated and compact clusters for most of the immune and tumor cells of different spatial locations (Fig. 2A). t-SNE and UMAP produced 2 large clusters that distinguished tumor and nontumor cells based on gene expression information. However, they could not distinguish the cell clusters with distinct spatial locations. For example, t-SNE and UMAP both presented the 6 tumor clusters with different spatial locations (tumor 1–6) as 1 large, disperse cluster and therefore lost the spatial information for them (Fig. 2B, C). The separation of cell clusters with different spatial locations is important because the cell states are influenced by their neighboring cells. For example, cells in tumor_2 and tumor_5, which are surrounded by immune cells and stroma cells, respectively (Supplementary Fig. S2a), have distinct expression patterns of marker genes such as IFI27, LGALS3BP, and B2M (Supplementary Fig. S2b, c). The genes that are highly expressed in tumor_2 (surrounded by immune cells) are involved in biological processes related to immune responses with high enrichment scores in Gene Ontology analysis (Supplementary Fig. S3a–c, −log10(p) >9), while the genes that are highly expressed in tumor_5 (surrounded by stroma cells) are involved in translation activities with low enrichment scores (Supplementary Fig. S3d–f, −log10(p) < 7). Similarly, cells in immune_2 and immune_1, which are surrounded by tumor and stroma, respectively (Supplementary Fig. S2a), have distinct expression patterns of marker genes such as ISG15, IFI6, and IFI27 (Supplementary Fig. S2b, c). The highly expressed genes in immune_2 are involved in immune responses, while the highly expressed genes in immune_1 are involved in other biological processes (Supplementary Fig. S4a–f). These results showed that SpaSNE could produce a more delicate visualization that distinguishes different cell states of the same cell type that interact with different spatial environments by leveraging both gene expression and spatial information. To comprehensively evaluate the performances of SpaSNE, t-SNE, and UMAP in a quantitative manner, we defined 3 quantitative measures: (i) Pearson correlation coefficient (${r}_g$) between pairwise gene expression distances and embedding distances of points, which was used to measure gene expression preservation; (ii) Pearson correlation coefficient (${r}_s$) between pairwise spatial position distances and embedding distances of points, which was used to measure spatial structure preservation; and (iii) silhouette score (*s*), which was used to measure the consistency of clustering with the ground-truth annotations. The comparison of the 3 algorithms showed that SpaSNE outperformed t-SNE and UMAP in all 3 measures (Fig. 2D).

The quantitative advantages of SpaSNE indicate better performances in revealing the underlying data structures. To demonstrate this, we highlighted several representative cell types with different spatial locations from the visualization maps in Fig. 2A–C to examine the performances of SpaSNE in detail (Fig. 2E–G). First, we highlighted 2 types of necrosis cells that have different levels of gene expression variabilities: necrosis_1 (blue) and necrosis_2 (orange) (“image” panel in Fig. 2E). The cells in necrosis_2 have a higher overall gene expression variability than the cells in necrosis_1 (Supplementary Fig. S5a). The difference in gene expression variability of these 2 cell clusters cannot be reflected in the image but can be revealed in SpaSNE, t-SNE, and UMAP maps (Fig. 2E). In the SpaSNE map, the necrosis_2 cluster has a larger size and smaller point density than that of necrosis_1 (Fig. 2E), which is consistent with the smaller gene expression variability of necrosis_2 (Supplementary Fig. S5a). t-SNE and UMAP also displayed similar properties, but the differences of cluster sizes and point densities between the 2 clusters are not as big as in SpaSNE (Fig. 2E). This example showed that the SpaSNE map can better reveal the gene expression variabilities of different cell clusters, which cannot be displayed by image alone.

In addition, we want to highlight that the better performance of SpaSNE in revealing gene expression variabilities can be explained by the higher ${r}_g$ value that reflects better preservation of gene expression distances (${r}_g$ = 0.66 in SpaSNE, 0.33 in t-SNE, and 0.26 in UMAP). When the gene expression preservation was tuned to be extremely high ($\alpha = 9,\ \beta = 0,\ {r}_g$ = 0.90, Supplementary Fig. S6c), the difference in gene expression variabilities was even larger, but the spatial structure preservation became worse (${r}_s$ = 0.11, Supplementary Fig. S6c). SpaSNE allows users to have the flexibility to adjust the preservation of gene expression and spatial structure according to their own research purposes.

Second, we highlighted 2 different cell types that were spatially close to each other: immune_2 (green) and tumor_2 (purple) (“image” panel in Fig. 2F). We observed that SpaSNE was able to preserve the relative spatial distances of these 2 cell populations by keeping them close to each other, while both t-SNE and UMAP displayed these 2 cell populations far away from each other without keeping the spatial contacts between them (Fig. 2F). The preservation of spatial organization in SpaSNE is due to the preservation of spatial distances (${r}_s$ = 0.72 in SpaSNE, 0.11 in t-SNE, and 0.12 in UMAP). When the spatial distances preservation was tuned to be extremely high ($\alpha = 0,\ \beta = 4,{\mathrm{\ }}{r}_s$ = 0.97, Supplementary Fig. S6d), the spatial structure more approximated the image, but the gene expression preservation became worse (${r}_g$ = 0.16, Supplementary Fig. S6d). This example indicated that SpaSNE could outperform t-SNE and UMAP in preserving spatial organization of cells in the micro-environment (e.g., in human cancers) without harming the capability in distinguishing distinct cell populations.

Third, we highlighted 2 different cell types in 2 spatially separated regions: immune_1 (red) and necrosis_1 (blue) (“image” panel in Fig. 2G). We observed that SpaSNE presented these 2 cell populations as 2 distinct clusters indicated by the pathological annotation, while both t-SNE and UMAP displayed them close to each other, though they are different cell types and spatially separated from each other (Fig. 2G). The better performance of SpaSNE in cell cluster separation is attributed to the higher clustering quality (*s* = 0.003 in SpaSNE, −0.16 in t-SNE, and −0.16 in UMAP). This example showed that the SpaSNE map better distinguishes cell clusters than t-SNE and UMAP, especially for the cell types that cannot be distinguished by gene expression information alone.

In summary, the above 3 examples show that SpaSNE gives an integrated low-dimensional visualization for spatially resolved profiling data and preserves information of both image and gene expression. SpaSNE visualization better reveals gene expression variabilities of cell clusters that are not visible from the image. It also outperforms t-SNE and UMAP in preserving the spatial organization of cells and better distinguishing different cell clusters.

### Application to the diseased prostate tissue data

**Figure 3:**
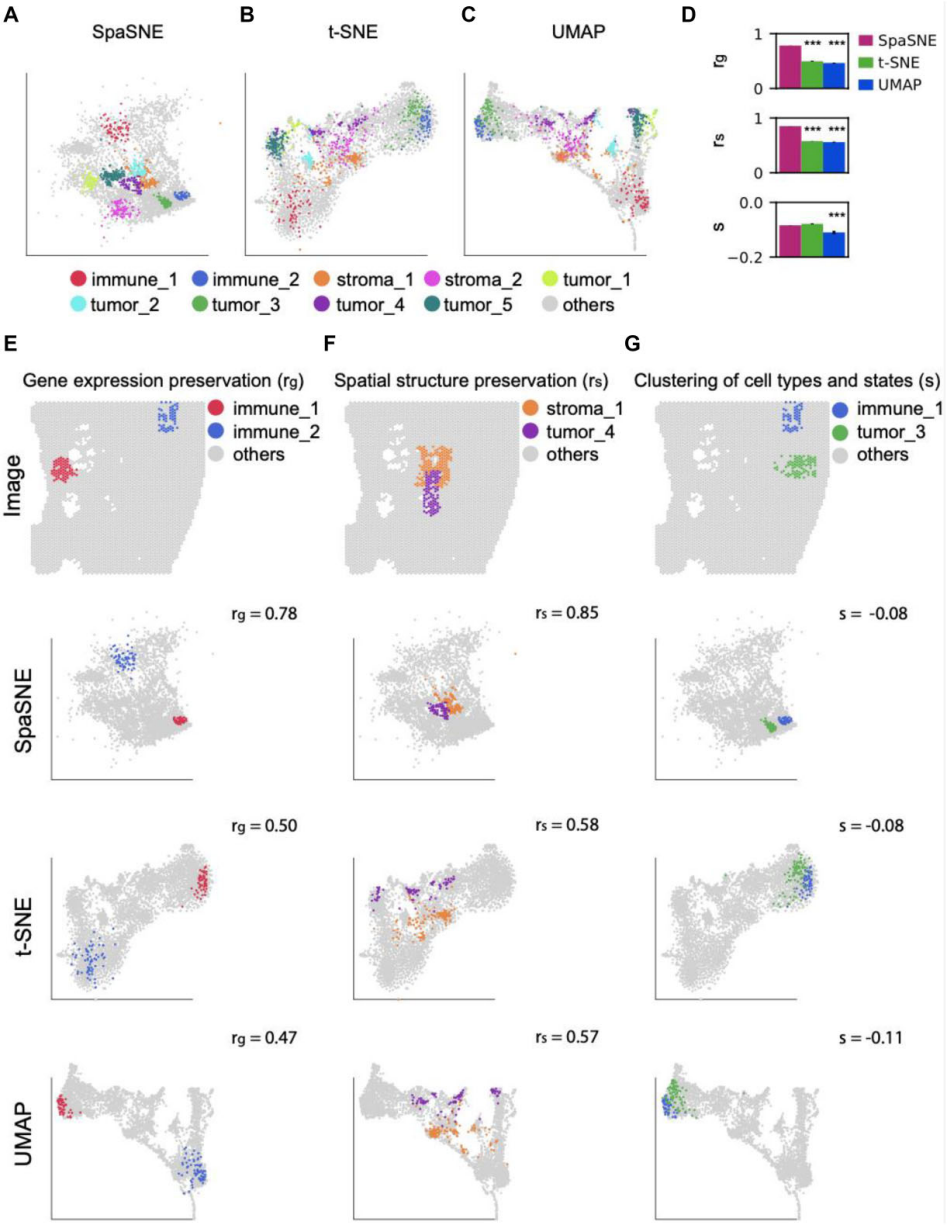
Visualizations of human prostate cancer data. (A–C) Two-dimensional visualization of cells colored according to ground-truth labels in (A) SpaSNE, (B) t-SNE, and (C) UMAP embeddings. Tumor, immune, and other regions that have different spatial locations are labeled by different numbers. (D) Using 3 quantitative measures to evaluate SpaSNE, t-SNE, and UMAP embeddings as in Fig. 1D. (E–G) Visualization of cells in raw image, SpaSNE, t-SNE, and UMAP embeddings by highlighting different pairs of cell states: (E) immune_1 (red) and immune_2 (blue), (F) stroma_1 (orange) and tumor_4 (purple), and (G) immune_1 (blue) and tumor_3 (green).

To demonstrate the general applicability of SpaSNE on different diseased tissue types, we shifted from the breast cancer tissues of female patients to the prostate cancer tissues of male patients, which were also obtained from the 10X Genomics data portal. This dataset consists of 4,371 spots with 3 highly mixed cell types: immune, stroma, and tumor cells. The cell-type annotations were defined by the HD-Staining [37] algorithm that was developed for classifying cell nuclei and cell types in the images (Supplementary Fig. S1b). We performed SpaSNE, t-SNE, and UMAP embeddings on this dataset and colored the cells according to cell types and spatial locations (Fig. 3A–C). SpaSNE presented separated and compact clusters for most of the colored cells, while t-SNE and UMAP could not well distinguish many of the cell clusters, for example, tumor 1 (light green) and tumor_2 (cyan) (Fig. 3B, C). The more delicate cell clusters separated by SpaSNE represent different cell states with different spatial environments. For example, cells in immune_2 and immune_1 are surrounded by tumor cells and stroma cells, respectively, and have distinct expression patterns of marker genes such as CNN1, DES, and TMEFF2 (Supplementary Fig. S7a–c). The genes highly expressed in immune_2 cells are involved in biological processes, including smooth muscle and smooth muscle cells, which play important roles in prostate cancer [50], with high enrichment scores (−log10(p) > 10) (Supplementary Fig. S8a–c). The genes highly expressed in immune_1 cells are involved in vesicle-related biological processes with low enrichment scores (−log10(p) < 4) (Supplementary Fig. S8d–f). SpaSNE also outperformed t-SNE and UMAP in the 3 quantitative measures (Fig. 3D), which is consistent with the results in human breast cancer data (Fig. 2D). We then evaluated the 3 qualitative performances accordingly (Fig. 3E–G) following the steps in Fig. 2E–G. First, we highlighted 2 types of immune cells: immune_1(red) and immune_2 (blue). The cells in immune_1 have a larger overall gene expression variability than cells in immune_2 (Supplementary Fig. S5b), which is consistent with a larger cluster size of immune_1 than that of immune_2 in the SpaSNE map. t-SNE and UMAP also displayed similar properties, but the differences in sizes between the 2 clusters were not as big as in SpaSNE (Fig. 3E). Second, we highlighted stroma_1 (orange) and tumor_4 (purple) that were spatially close to each other. The relative spatial distances between these 2 cell populations were better preserved in SpaSNE than in t-SNE and UMAP (Fig. 3F). Third, we highlighted immune_1 (blue) and tumor_3 (green) that were spatially far from each other. These 2 cell populations were presented as 2 tight and separable clusters in SpaSNE. t-SNE and UMAP displayed similar properties but were slightly less separable (Fig. 3G). The above 3 qualitative evaluations were consistent with the results in human breast cancer data (Fig. 3E–G).

In summary, despite the differences in the disease types and data sources, SpaSNE could outperform t-SNE and UMAP in revealing gene expression variabilities of cell clusters, preserving the spatial organization of cells, and distinguishing different cell clusters. These results demonstrated SpaSNE’s potential to serve as a reliable tool for visualizing molecular and spatial information in diverse spatially resolved profiling datasets.

### Application to normal tissues based on image-based spatially resolved profiling platforms

We have demonstrated the advantages of SpaSNE on NGS-based experimental platforms. Next, we will apply SpaSNE to image-based spatially resolved profiling platforms. Different from the diseased tissues, the cells in normal tissues are more homogeneous in gene expression and are usually labeled by tissue types (e.g., developmental layers), and the spatial information mainly represents the global organization of the tissues (e.g., developmental trajectory). Therefore, instead of examining the visualization of gene expression variabilities and spatial closeness in diseased tissues (Figs. 2–3), we focused on the following 2 comparisons among SpaSNE, t-SNE, and UMAP by analyzing the spatially resolved data from normal tissues: (i) distinguishing different tissue types and (ii) revealing the global organization of the tissues.

**Figure 4:**
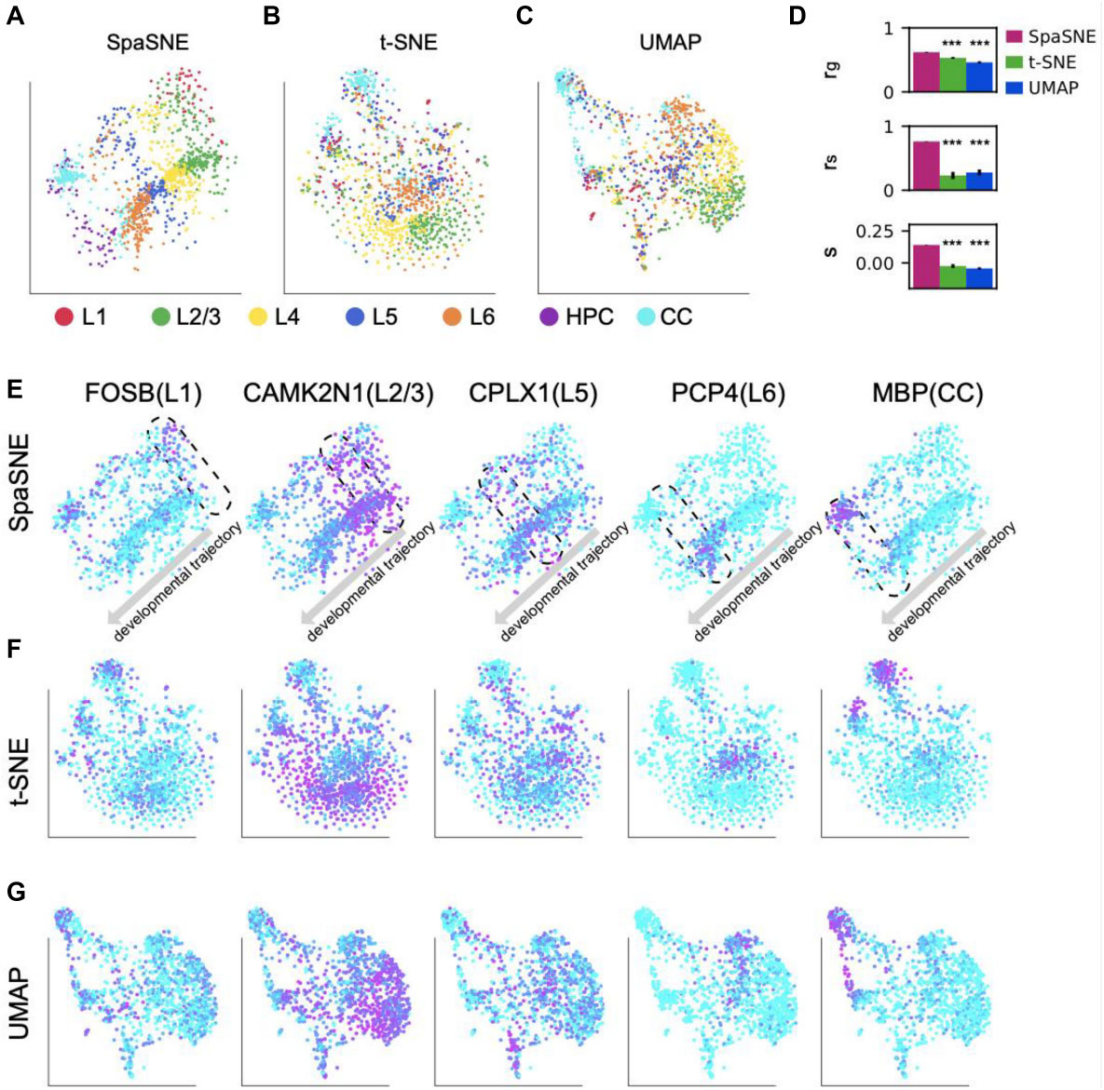
Visualizations of mouse visual cortex STARmap data. (A–C) Two-dimensional visualization of cells colored according to ground-truth labels in (A) SpaSNE, (B) t-SNE, and (C) UMAP embeddings. (D) Using 3 quantitative measures to evaluate SpaSNE, t-SNE, and UMAP embeddings as in Fig. 1D. (E, F) Gene expression patterns of 5 layer marker genes FOSB, CAMK2N1, CPLX1, PCP4, and MBP in (E) SpaSNE, (F) t-SNE, and (G) UMAP embeddings. The dashed boxes in (E) highlight the areas with high gene expression, and the arrows represent the developmental trajectory. Magenta represents high expression and cyan low expression.

We first analyzed a mouse visual cortex STARmap dataset [2] that was obtained from normal eyes. In this dataset, 1,020 genes were measured in 1,207 cells from 7 layers: hippocampus (HPC), corpus callosum (CC), layer 1 (L1), layer 2/3 (L2/3), layer 4 (L4), layer 5 (L5), and layer 6 (L6) (Supplementary Fig. S1c). We performed SpaSNE, t-SNE, and UMAP’s embeddings for this dataset and observed that SpaSNE better distinguishes these 7 layers than t-SNE and UMAP (Fig. 4A–C). This unique feature of SpaSNE can be useful for developmental biologists who are interested in studying the tissue- and organ-level morphogenesis, where the cells organize themselves into distinct layers, but the gene expression differences might be subtle. SpaSNE also outperformed t-SNE and UAMP in the 3 quantitative measures (Fig. 4D). To study whether SpaSNE can reveal the developmental trajectory in normal tissues, we mimicked a classic analysis approach in the original UMAP publication [18]. In their analysis, the authors utilized several known marker genes to represent different cell types and studied the impacts of dimensionality reduction (e.g., UMAP and t-SNE) on visualization of the differentiation trajectory based on the expression trend of these marker genes. Here, we performed differential expression analysis based on the 6 layers and found that the differentially expressed genes are involved in biological processes, including system development and neurogenesis (Supplementary Fig. S9a–c). By comparing each layer with the rest of the layers, we identified layer-specific markers genes and selected 5 of them for visualization: FOSB(L1), CAMK2N1(L2/3), CPLX1(L5), PCP1(L6), and MBP(CC) (Supplementary Fig. S10). The expressions of the 5 marker genes peaked at different areas (dashed boxes) and formed a clear developmental trajectory that moves sequentially from the top right to the bottom left in the SpaSNE map, as shown by the arrows in Fig. 4E. In t-SNE and UMAP, the expression of the 4 markers genes did not show a smooth trend, and the developmental trajectory is not as clear as in SpaSNE (Fig. 4F–G).

**Figure 5:**
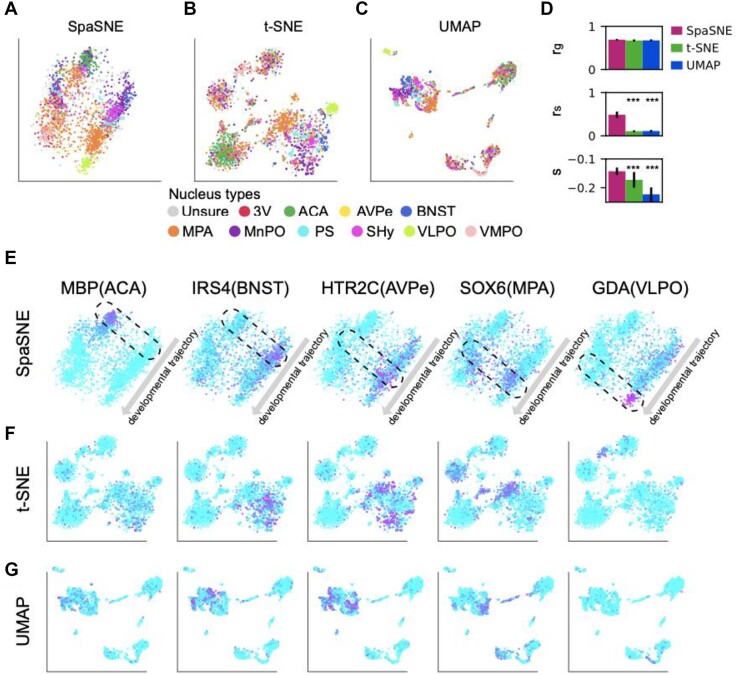
Visualizations of mouse hypothalamus MERFISH data. (A–C) Two-dimensional visualization of cells colored according to ground-truth nucleus type labels in (A) SpaSNE, (B) t-SNE, and (C) UMAP embeddings. (D) Using 3 quantitative measures to evaluate SpaSNE, t-SNE, and UMAP embeddings as in Fig. 1D. (E–G) Gene expression patterns of 5 nucleus marker genes MBP, IRS4, HTR2C, SOX6, and GDA in (E) SpaSNE, (F) t-SNE, and (G) UMAP embeddings. The dashed boxes in (E) highlight the areas with high gene expression, and the arrows represent the nucleus organization. Magenta represents high expression and cyan low expression.

Besides the STARmap experimental platform, we analyzed another type of image-based spatially resolved expression profiling platform: MERFISH. This MERFISH dataset [25] contains 5,665 cells and 161 genes from the mouse brain hypothalamus. Since the whole hypothalamus image is symmetric, we took 2,693 cells from the left half of the image for analysis. The cells were colored according to the nucleus types (Fig. 5, Supplementary Fig. S1d). We performed SpaSNE, t-SNE, and UMAP’s embeddings for this dataset (Fig. 5A–C). We observed that SpaSNE better distinguishes different nucleus types compared with t-SNE and UMAP (Fig. 5A–C). SpaSNE also outperformed t-SNE and UAMP in the 3 quantitative measures (Fig. 5D). Following the analysis in Supplementary Figs. S9–S10, we performed differential expression analysis based on the 11 nucleus types and found that the differentially expressed genes are involved in biological processes, including multicellular organismal process and nervous system development (Supplementary Fig. S11a–c). By comparing each layer with the rest of the layers, we identified layer-specific marker genes and selected 5 of them for visualization: MBP(ACA), IRS4(BNST), HTR2C (AVPe), SOX6(MPA), and GDA(VLPO) (Supplementary Fig. S12). The expressions of the 5 marker genes peaked at different areas (dashed boxes) and formed a clear trajectory from the top right to the bottom left in the SpaSNE map, as shown by the arrows in Fig. 5E, while the gene expression patterns in the t-SNE or UMAP maps are not as clear as in SpaSNE (Fig. 5F, G).

In summary, the above analyses on 2 normal tissue datasets consistently show that SpaSNE outperforms t-SNE and UMAP in (i) distinguishing different tissue types (e.g., developmental layers) and (ii) revealing the global organization of the tissues (e.g., developmental trajectory), regardless of organ types, data sources, and experimental platforms.

## Discussion

SpaSNE extends the function of t-SNE by not only preserving the local structure of molecular data (e.g., gene expression data) but also maintaining the large-scale structure of molecular data and integrating the spatial information of the cells. With this adaptation, SpaSNE better preserves both molecular data structure and spatial organization of spatially resolved profiling data, which leads to multiple advantages over t-SNE and UMAP. First and most importantly, SpaSNE outperforms t-SNE and UMAP in presenting more accurate and delicate clustering of the cell types with different spatial locations, which is the key step for multiple subsequent statistical and bioinformatics analyses that require correct information of cell types, including but not limited to the differential expression between different cell types, cellular communications among various cell types, and network/pathway-based analysis on each cell type.

Second, SpaSNE can preserve both the spatial organization of cells in the micro-environment and the developmental trajectory in the tissues. Exploring cellular communications in the micro-environment [13, 22–24, 33, 34] and developmental process [14, 29–31, 36] have been the primary goals of many spatially resolved profiling studies [51]. The better spatial structure preservation of SpaSNE over t-SNE and UMAP can support the better preservation of cellular communications in the micro-environment and the spatial organization in developmental tissues. Therefore, SpaSNE could serve as an ideal dimensionality reduction and visualization tool in these research directions, regardless of tissue types and experimental platforms.

Third, SpaSNE offers tunable parameters to adjust the users’ requests for the preservation of molecular or spatial information. SpaSNE is capable of integrating 2 independent sources of data—molecular data (e.g., gene expression data) and spatial position data of cells into a single map. These 2 aspects represent different biological information and are balanced by the 2 weighting parameters $\alpha $ and $\beta $. Emphasizing the gene expression information (larger $\alpha $ and smaller $\beta $) would enhance the gene expression preservation but diminish the spatial structure preservation (${r}_g$ = 0.90, ${r}_s$ = 0.11, Supplementary Fig. S6a–c), while emphasizing spatial structure (smaller $\alpha $ and larger $\beta $) would make the visualization more like the image but not able to reliably reveal gene expression variabilities (${r}_g$ = 0.16, ${r}_s$ = 0.97, Supplementary Fig. S6d). SpaSNE allows users to have the flexibility to adjust the balance between gene expression preservation and spatial structure preservation according to their own research purposes, so that users can make the most use of spatially resolved profiling data for data interpretation and hypothesis generation.

Fourth, SpaSNE is a data integration method that is capable of integrating multiple independent features from the same samples (e.g., spatial positions and gene expression that do not share common features but are from the same samples). Traditional data integration methods, such as MultiMAP [52], were usually designed to integrate multiple related features from 2 or more different samples (e.g., single-cell ATAC sequencing and single-cell RNA sequencing data that share common genes but are from different samples). SpaSNE and MultiMAP serve as 2 complementary methods. When applied to spatially resolved profiling data (e.g., STARmap [2]), MultiMAP helps to improve the clustering of cells by leveraging transcriptomics information from another single-cell RNA sequencing data from a different sample. However, we found that MultiMAP cannot preserve the spatial structure of the cells when comparing with ground-truth spatial position annotation from the original image [2] (Supplementary Fig. S13a, c). SpaSNE can make better use of spatially resolved profiling data by preserving both gene expression and spatial organization of the cells (Supplementary Fig. S13a, d). These 2 methods could potentially be integrated to build a more powerful visualization method that can integrate both independent and related features from multiomics datasets.

We have demonstrated that SpaSNE outperforms t-SNE and UAMP in achieving more accurate clustering for diseased tissues and more meaningful global structure for normal tissues. The advantages in clustering and global structure preservation in low-dimensional visualization could have direct impacts on downstream analyses, such as cellular communications among different cell types or cells at different spatial locations, differential gene expression across cell types or along developmental trajectories, and so on. Working on these directions is warranted in our follow-up studies.

Despite its extensive utility, SpaSNE has several limitations. First, SpaSNE was designed as a visualization tool in 2-dimensional space with similar purposes as t-SNE or UMAP but not as a clustering tool for spatially resolved cell-type clustering tasks such as SpatialPCA [8]. Thus, there is no direct comparison between SpaSNE and SpatialPCA. However, a comparison cannot still be made if setting the embedding dimension of SpatialPCA to be 2, and we showed that SpaSNE outperformed SpatialPCA (d = 2) in the quantitative evaluations on all the 4 datasets (Supplementary Fig. S14a–h).

Second, we realized that published spatially resolved profiling datasets usually contain a relatively limited number of cells (or spots) in each slide, for which the SpaSNE package is efficient at completing the analysis (Supplementary Table S1). However, spatially resolved profiling datasets are rapidly growing, and handling large datasets might be needed in the near future. The current SpaSNE package has not been optimized for handling datasets with a large number of cells. A further improvement in this direction may be considered in our follow-up research of the algorithm development. Third, in this study, we have demonstrated that SpaSNE is suitable for both NGS-based (Figs. 2–3) and imaging-based spatially resolved experimental platforms (Figs. 4–5). Because new spatially resolved profiling technologies are still emerging, a more complete evaluation of these new spatially resolved profiling platforms by SpaSNE would be considered in our follow-up study. Fourth, the design of SpaSNE assumes that the spatial information in spatially resolved transcriptomics data will contribute to the identification of cell states or the global organization of the cells. It may be less effective when there is no correlation between the phenotype and spatial positions of cells [53]. In addition, its performance may be compromised in situations where spatial transcriptomics measurements are taken at multicellular resolution where a single spot contains multiple cell types, or in subcellular resolution where a single cell covers multiple spatial positions such as Visium HD data [54]. We may adapt SpaSNE to analyze such datasets by incorporating current decomposition [55] or aggregation [56] methods for the preprocessing of data in the future. For the 4 datasets, we found that SpaSNE achieved better embedding quality for the human breast cancer dataset and human prostate cancer dataset generated from the 10X Visium platform than the mouse visual cortex dataset from the STARmap platform and mouse hypothalamus dataset from the MERFISH platform. The reason might be that 10X Visium platform measures a larger number of genes than STARmap and MERFISH and therefore helps better define cell types. Last but not least, we are working on developing a plug-in to run SpaSNE in the popular single-cell and spatial data analysis software platforms and toolkits (e.g., Seurat [57]), in order to support wider applications of SpaSNE on a variety of rapidly emerging spatially resolved profiling datasets.

We currently focused on using available spatially resolved gene expression profiling data to demonstrate that SpaSNE can serve as a powerful dimensionality reduction and visualization tool for analyzing the spatially resolved profiling datasets with both molecular and spatial information. The design of SpaSNE allows it to analyze not only spatially resolved gene transcriptomic data but also other types of datasets with similar data structures such as spatially resolved epigenomic [58] and proteomic datasets [59]. Nowadays, biological and medical researches are trending toward a large number of dimensions in tens of thousands of cells or spots with the spatial organization’s information. Providing a reliable and robust interpretation of cell types based on both molecular and spatial information by a dimensionality reduction approach can set the foundation for many subsequent analysis steps (e.g., differential gene expression, epigenetic regulation, or protein expression among cell types with spatial organization patterns) and therefore would play an important role in analyzing various spatially resolved profiling data.

## Conclusions

This study highlights the versatile utility of SpaSNE in facilitating the accurate and resilient interpretation of cell types by leveraging a combination of molecular and spatial information. This framework establishes a solid groundwork for various subsequent analytical procedures, including, but not limited to, differential gene expression, trajectory analysis, and pseudotime analysis, thereby enhancing the depth and precision of spatially resolved profiling data exploration.

## Availability of Source Code and Requirements

Project name: SpaSNE

Project homepage: [60]

Operating system(s): Linux or MacOS

Programming language: Python and C++

Other requirements: Python 3.8 and GCC 11.4

License: BSD 3-clause license

RRID:SCR_026223

SpaSNE is also archived in Software Heritage [61]. The guidelines for installing the SpaSNE package and the tutorials for screening optimal parameters of SpaSNE and performing SpaSNE embeddings with different parameters on human breast cancer data are available on the GitHub page [62]. The SpaSNE software was adapted from the bhtsne scripts [63].

## Supplementary Material

giaf002_Supplement_Files

giaf002_Authors_Response_To_Reviewer_Comments

giaf002_GIGA-D-24-00148

giaf002_GIGA-D-24-00148_R1

giaf002_Reviewer_1_Report_Original_Submission Etienne Becht -- 6/3/2024

giaf002_Reviewer_1_Report_Revision_1 Etienne Becht -- 11/26/2024

giaf002_Reviewer_2_Report_Original_Submission Raffaele A Calogero, B.Sc. -- 8/5/2024

giaf002_Reviewer_3_Report_Original_Submission Zhixiang Lin -- 8/6/2024

## Data Availability

The test datasets are available from links [64–67].

## Additional Files

**Supplementary Fig. S1**. Images of the 4 spatially resolved transcriptomics data. (a) Image of human breast cancer data colored by 5 cell types: fat, stroma, immune, necrosis, and tumor. (b) Image of human prostate cancer data colored by 3 cell types: immune, stroma, and tumor. (c) Image of mouse visual cortex data colored according to 7 layers: hippocampus (HPC), corpus callosum (CC), layer 1 (L1), layer 2/3 (L2/3), layer 4 (L4), layer 5 (L5), and layer 6 (L6). (d) Image of mouse visual cortex data colored according to 10 nucleus types: 3V, ACA, AVPe, BNST, MPA, MnPO, PS, SHy, VLPO, and VMPO.

**Supplementary Fig. S2**. Differential gene expressions of selected cell clusters in human breast cancer tissue. (a) Annotation of human breast cancer cell clusters according to cell types (left) and cell states (right). The cell states annotation is the same as in Fig. 2A–C in the main text. (b) Violin plots of top differentially expressed genes in tumor_2, tumor_5, immune_1, immune_2, and the rest of the cell types. (c) Mean expression values of top differentially expressed genes in the selected cell types.

**Supplementary Fig. S3**. Comparison of 2 tumor subtypes. (a–c) Differentially expressed genes analysis with tumor_5 as reference. (a) Violin plots of the top differentially expressed genes. (b) The top 15 Gene Ontology Biological Processes obtained from the top 30 differentially expressed genes. (c) Expressions of 3 differentially expressed genes IFI27, LGALS3BP, and B2M. (d–f) Differentially expressed genes analysis with tumor_2 as reference. (d) Violin plots of the top differentially expressed genes. (e) The top 15 Gene Ontology Biological Processes obtained from the top 30 differentially expressed genes. (f) Expressions of 3 differentially expressed genes FTH1, TMEM258, and CYP4Z1.

**Supplementary Fig. S4**. Comparison of 2 immune subtypes. (a–c) Differentially expressed genes analysis with immune_1 as reference. (a) Violin plots of the top differentially expressed genes. (b) The top 15 Gene Ontology Biological Processes obtained from the top 30 differentially expressed genes. (c) Expressions of 3 differentially expressed genes ISG15, IFI6, and IFI27. (d–f) Differentially expressed genes analysis with immune_2 as reference. (d) Violin plots of the top differentially expressed genes. (e) The top 15 Gene Ontology Biological Processes obtained from the top 30 differentially expressed genes. (f) Expressions of 3 differentially expressed genes HMOX1, PSAP, and FTL.

**Supplementary Fig. S5**. Gene expression variability in different cell states. (a) Box plots of standard deviation of gene expression in cells of necrosis_1 and necrosis_2 in human breast cancer data. (b) Box plots of the standard deviation of gene expression in cells of immune_1 and immune_2 in human prostate cancer data.

**Supplementary Fig. S6**. SpaSNE results on human breast cancer with extreme parameters. (a) Visualization of cells in the raw image, SpaSNE, t-SNE, and UMAP embeddings by highlighting different pairs of cell states. (b–d) SpaSNE embeddings with different combinations of parameters (*α, β*): (*α* = 9, *β* = 4) in (b), (*α* = 9, *β* = 0) in (c), and (*α* = 0, *β* = 4) in (d). The 2 parameters *α* and *β* represent the weights of the global gene expressions’ loss function *L_g_* and the spatial loss function *L_s_*.

**Supplementary Fig. S7**. Differential gene expressions of selected cell clusters in human prostate cancer tissue. (a) Annotation of human prostate cancer cell clusters according to cell types (left) and cell states (right). This annotation is the same as in Fig. 3A–C in the main text. (b) Violin plots of top differentially expressed genes in tumor_1, tumor_2, immune_1, immune_2, and the rest of the cell types. (c) Mean expression values of top differentially expressed genes in the selected cell types.

**Supplementary Fig. S8**. Comparison of 2 immune subtypes. (a–c) Differentially expressed genes analysis with immune_2 as reference. (a) Violin plots of the top differentially expressed genes. (b) The top 15 Gene Ontology Biological Processes obtained from the top 30 differentially expressed genes. (c) Expressions of 3 differentially expressed genes CNN1, DES, and TPM1. (d–f) Differentially expressed genes analysis with immune_1 as reference. (d) Violin plots of the top differentially expressed genes. (e) The top 15 Gene Ontology Biological Processes obtained from the top 30 differentially expressed genes. (f) Expressions of 3 differentially expressed genes TMEFF2, KLK3, and PLA2G2A.

**Supplementary Fig. S9**. Differential gene expressions of 7 layers in mouse visual cortex tissue. (a) Violin plots of top differentially expressed genes in the 7 layers. (b) Mean expression values of top differentially expressed genes in the 7 layers. (c) The top 15 Gene Ontology Biological Processes obtained from 70 differentially expressed genes.

**Supplementary Fig. S10**. Differentially expressed genes from the comparison between each layer type and the rest of the layers in mouse visual cortex data. The genes marked red are the genes shown in Fig. 4E–G in the main text.

**Supplementary Fig. S11**. Differential gene expressions of 7 layers in mouse hypothalamus tissue. (a) Violin plots of top differentially expressed genes in the 7 layers. (b) Mean expression values of top differentially expressed genes in the 7 layers. (c) The top 15 Gene Ontology Biological Processes obtained from 90 differentially expressed genes.

**Supplementary Fig. S12**. Differentially expressed genes from the comparison between each layer type and the rest of the layers in mouse hypothalamus data. The genes marked red are the genes shown in Fig. 5E–G in the main text.

**Supplementary Fig. S13**. Comparison between UMAP, MultiMAP, and SpaSNE in preserving spatial organization of cells. (a) Spatial organization of cells from the image in STARmap data colored by 4 excitatory neuron types (eL2/3, eL4, eL5, eL6) and other cells (Other). (b–d) Visualization of cells by (b) UMAP, (c) MultiMAP, and (d) SpaSNE. In MultiMAP, 1,207 cells from STARmap data were coembedded with 10,000 randomly selected cells from scRNA data used in MultiMAP publication. *r_g_* and *r_s_* above panels in (b–d) represent the quantitative measures of gene expression preservation and spatial structure preservation, respectively.

**Supplementary Fig. S14**. Comparison of SpaSNE with SpatialPCA (d = 2) on visualization of the 4 datasets. (a) SpatialPCA visualization on human breast cancer dataset with d = 2. (b) SpaSNE visualization on human breast cancer dataset. (c) SpatialPCA visualization on human prostate cancer dataset with d = 2. (d) SpaSNE visualization on human prostate cancer dataset. (e) SpatialPCA visualization on mouse visual cortex dataset with d = 2. (f) SpaSNE visualization on mouse visual cortex dataset. (g) SpatialPCA visualization on mouse hypothalamus dataset with d = 2. (h) SpaSNE visualization on mouse hypothalamus dataset.

**Supplementary Fig. S15**. Embedding performances of SpaSNE using the metric of trustworthiness (tw). (a) Evaluation of t-SNE embedding on human breast cancer data with trustworthiness score for spatial local structure (tw_spatial), trustworthiness score for transcriptomic local structure (Tw spatial), and the product of the 2 scores (tw_prod). (b) Evaluation of SpaSNE embedding with the 3 scores. (c, d) The same analyses on mouse visual cortex data.

**Supplementary Fig. S16**. Rough and fine screenings of parameters *α* and *β* of SpaSNE on the 4 datasets. For each dataset, a rough screening was first performed to determine the range of optimal parameters on a large scale (a, c, e, g), and then a fine screening was performed to find the optimal parameters with a fine resolution (b, d, f, h). In each embedding, setting *r_g_* = 0 if *r_g_* ≤ *r_thres_*, where *r_thres_* is the maximal *r_g_* in 100 repeats of t-SNE embeddings. The heatmaps for rough tuning represent values of *r_g_* × *r_s_* with different combinations of parameters. The heatmaps for fine tuning represent values of *r_g_* × *r_s_* (left), std (middle), and *r_g_* × *r_s_* × exp (1 − std)(right). The std is the standard deviation of *r_g_* in multiple repeats of SpaSNE embeddings in each parameter combination. The parameter ranges used in screening and optimal parameters for the 4 datasets can be found in Supplementary Table S1. (a, b) Rough (a) and fine (b) screening on human breast cancer data. (c, d) Rough (c) and fine (d) screening on human prostate cancer data. (e, f) Rough (e) and fine (f) screening on mouse visual cortex data. (g, h) Rough (g) and fine (h) screening on mouse hypothalamus data.

**Supplementary Fig. S17**. SpaSNE embeddings on the 4 datasets with different perplexity values (ppl). Three perplexity values were applied on each dataset: ppl = 5, 50, 100. The default value in SpaSNE is 50. (a–d) Results on human breast cancer dataset (a), human prostate cancer dataset (b), mouse visual cortex dataset (c), and mouse hypothalamus dataset (d).

**Supplementary Table S1**. Introducing the 4 datasets and running time of SpaSNE.

**Supplementary Table S2**. Annotations for human breast cancer. cell_type: Annotation for Supplementary Fig. 1a. sub_type_1: Annotation for Fig. 2a–c. sub_type_2: Annotation for Fig. 2e. sub_type_3: Annotation for Fig. 2f. sub_type_4: Annotation for Fig. 2g. sub_type_5: Annotation for calculating silhouette scores in Fig. 2d.

**Supplementary Table S3**. Annotations for human prostate cancer. cell_type: Annotation for Supplementary Fig. 1b. sub_type_1: Annotation for Fig. 3a–c. sub_type_2: Annotation for Fig. 3e. sub_type_3: Annotation for Fig. 3f. sub_type_4: Annotation in Fig. 3g. sub_type_5: Annotation for calculating silhouette scores in Fig. 3d.

**Supplementary Table S4**. Annotations for mouse visual cortex data. layer_type: Annotation for Supplementary Fig. 1c and Fig. 4a–c and for calculating silhouette scores in Fig. 4d.

**Supplementary Table S5**. Annotations for mouse hypothalamus data. nucleus_type: Annotation for Supplementary Fig. 1d and Fig. 5a–c and for calculating silhouette scores in Fig. 5d.

## Abbreviations

KL: Kullback–Leibler; NGS: next-generation sequencing; PCA: principal component analysis; SpaSNE: spatially resolved t-SNE; t-SNE: t-distributed stochastic neighbor embedding; UMAP: uniform manifold approximation and projection; UMI: unique molecular identifier.
