# Supplementary material for: Dimensionality reduction for visualizing spatially resolved profiling data using SpaSNE
Source: Gigascience. 2025 Feb 17;14:giaf002. doi: 10.1093/gigascience/giaf002 (PMC11831803; doi:10.1093/gigascience/giaf002)

Supplementary Figure S1. Images of the four spatially resolved transcriptomics data. (**a**) Image of human breast cancer data colored by five cell types: fat, stroma, immune, necrosis, and tumor. (**b**) Image of human prostate cancer data colored by three cell types: immune, stroma, and tumor. (**c**) Image of mouse visual cortex data colored according to seven layers: hippocampus (HPC), corpus callosum (CC), layer 1 (L1), layer 2/3 (L2/3), layer 4 (L4), layer 5 (L5), and layer 6 (L6). (**d**) Image of mouse visual cortex data colored according to ten nucleus types: 3V, ACA, AVPe, BNST, MPA, MnPO, PS, SHy, VLPO, and VMPO.

Supplementary Figure S2. Differential gene expressions of selected cell clusters in human breast cancer tissue. (a) Annotation of human breast cancer cell clusters according to cell types (left) and cell states (right). The cell states annotation is the same as in Fig.2a-c in the manuscript. (b) Violin plots of top differentially expressed genes in tumor_2, tumor_5, immune_1, immune_2, and the rest cell types. (c) Mean expression values of top differentially expressed genes in the selected cell types.

Supplementary Figure S3. Comparison of two tumor subtypes. (a-c) Differentially expressed genes analysis with tumor_5 as reference. (a) Violin plots of the top differentially expressed genes. (b) The top 15 Gene Ontology Biological Processes obtained from the top 30 differentially expressed genes**.** (c) Expressions of three differentially expressed genes IFI27, LGALS3BP, and B2M. (d-f) Differentially expressed genes analysis with tumor_2 as reference. (d) Violin plots of the top differentially expressed genes. (e) The top 15 Gene Ontology Biological Processes obtained from the top 30 differentially expressed genes**.** (f) Expressions of three differentially expressed genes FTH1, TMEM258, and CYP4Z1.

Supplementary Figure S4. Comparison of two immune subtypes. (a-c) Differentially expressed genes analysis with immune_1 as reference. (a) Violin plots of the top differentially expressed genes. (b) The top 15 Gene Ontology Biological Processes obtained from the top 30 differentially expressed genes**.** (c) Expressions of three differentially expressed genes ISG15, IFI6 and IFI27. (d-f) Differentially expressed genes analysis with immune_2 as reference. (d) Violin plots of the top differentially expressed genes. (e) The top 15 Gene Ontology Biological Processes obtained from the top 30 differentially expressed genes**.** (f) Expressions of three differentially expressed genes HMOX1, PSAP, and FTL.

Supplementary Figure S5. Gene expression variability in different cell states. (a) Box plots of standard deviation of gene expression in cells of necrosis_1 and necrosis_2 in human breast cancer data. **(b)** Box plots of the standard deviation of gene expression in cells of immune_1 and immune_2 in human prostate cancer data.

Supplementary Figure S6. SpaSNE results on human breast cancer with extreme parameters. (**a**) Visualization of cells in the raw image, SpaSNE, t-SNE, and UMAP embeddings by highlighting different pairs of cell states. (**b-d**) SpaSNE embeddings with different combinations of parameters (α, β): (α = 9, β = 4) in (**b**), (α = 9, β = 0) in (**c**),

and (α = 0, β = 4) in (**d**). The two parameters α and β represent the weights of the global gene expressions' loss function L_g and the spatial loss function L_s.

Supplementary Figure S7. Differential gene expressions of selected cell clusters in human prostate cancer tissue. (a) Annotation of human prostate cancer cell clusters according to cell types (left) and cell states (right). This annotation is the same as in Fig.3a-c in the manuscript. (b) Violin plots of top differentially expressed genes in tumor_1, tumor_2, immune_1, immune_2, and the rest cell types. (c) Mean expression values of top differentially expressed genes in the selected cell types.

Supplementary Figure S8. Comparison of two immune subtypes. (a-c) Differentially expressed genes analysis with immune_2 as reference. (a) Violin plots of the top differentially expressed genes. (b) The top 15 Gene Ontology Biological Processes obtained from the top 30 differentially expressed genes**.** (c) Expressions of three differentially expressed genes CNN1, DES, and TPM1. (d-f) Differentially expressed genes analysis with immune_1 as reference. (d) Violin plots of the top differentially expressed genes. (e) The top 15 Gene Ontology Biological Processes obtained from the top 30 differentially expressed genes**.** (f) Expressions of three differentially expressed genes TMEFF2, KLK3, and PLA2G2A.

Supplementary Figure S9. Differential gene expressions of seven layers in mouse visual cortex tissue. (a) Violin plots of top differentially expressed genes in the seven layers. (b) Mean expression values of top differentially expressed genes in the seven layers. (c) The top 15 Gene Ontology Biological Processes obtained from 70 differentially expressed genes**.**

Supplementary Figure S10. Differentially expressed genes from the comparison between each layer type and the rest layers in mouse visual cortex data. The genes marked red are the genes shown in Fig. 4e-g in the manuscript.

Supplementary Figure S11. Differential gene expressions of seven layers in mouse hypothalamus tissue. (a) Violin plots of top differentially expressed genes in the seven layers. (b) Mean expression values of top differentially expressed genes in the seven layers. (c) The top 15 Gene Ontology Biological Processes obtained from 90 differentially expressed genes**.**

Supplementary Figure S12. Differentially expressed genes from the comparison between each layer type and the rest layers in mouse hypothalamus data. The genes marked red are the genes shown in Fig. 5e-g in the manuscript.

Supplementary Figure S13. Comparison between UMAP, MultiMAP, and SpaSNE in preserving spatial organization of cells. (**a**) Spatial organization of cells from the image in STARmap data colored by four excitatory neuron types (eL2/3, eL4, eL5, eL6) and other cells (Other). (**b-c**) Visualization of cells by (**b**) UMAP, (**c**) MultiMAP, and

(**d**) SpaSNE. In MultiMAP, 1207 cells from STARmap data were co-embedded with 10000 randomly selected cells from scRNA data used in MultiMAP publication. r_g and r_s above panels in (**b-d**) represent the quantitative measures of gene expression preservation and spatial structure preservation respectively**.**

Supplementary Figure S14. Comparison of SpaSNE with SpatialPCA (d = 2) on visualization of the four datasets. (a) SpatialPCA visualization on human breast cancer dataset with d = 2. (b) SpaSNE visualization on human breast cancer dataset. (c) SpatialPCA visualization on human prostate cancer dataset with d = 2. (d) SpaSNE visualization on human prostate cancer dataset. (e) SpatialPCA visualization on mouse visual cortex dataset with d = 2. (f) SpaSNE visualization on mouse visual cortex dataset. (g) SpatialPCA visualization on Mouse hypothalamus dataset with d = 2. (h) SpaSNE visualization on Mouse hypothalamus dataset.

Supplementary Figure S15. Embedding performances of SpaSNE using the metric of trustworthiness (tw). (a) Evaluation of t-SNE embedding on human breast cancer data with trustworthiness score for spatial local structure (tw_spatial), trustworthiness score for transcriptomic local structure (Tw spatial), and the product of the two scores (tw_prod). (**b**) Evaluation of SpaSNE embedding with the three scores. (**c-d**) The same analyses on mouse visual cortex data.

Supplementary Figure S16. Rough and fine screenings of parameters $\boldsymbol{\alpha}$ and $\boldsymbol{\beta}$ of SpaSNE on the four datasets. For each dataset, a rough screening was first performed to determine the range of optimal parameters on a large scale (a, c, e, g), then a fine screening was performed to find the optimal parameters with a fine resolution (b, d, f, h). In each embedding, setting $\mathrm{r_g} = 0$ if $\mathrm{r_g} \leq \mathrm{r_{thres}}$, where $\mathrm{r_{thres}}$ is the maximal $\mathrm{r_g}$ in 100 repeats of t-SNE embeddings. The heatmaps for rough tuning represent values of $\mathrm{r_g} \times \mathrm{r_s}$ with different combinations of parameters. The heatmaps for fine tuning represent values of $\mathrm{r_g} \times \mathrm{r_s}$ (left), std (middle) and $\mathrm{r_g} \times \mathrm{r_s} \times \exp{(1 - \mathrm{std})}$(right). The std is the standard deviation of $\mathrm{r_g}$ in multiple repeats of SpaSNE embeddings in each parameter combination. The parameter ranges used in screening and optimal parameters for the four datasets can be found in **Supplementary Table S1**. (a-b) Rough (a) and fine (b) screening on human breast cancer data. (c-d) Rough (c) and fine (d) screening on human prostate cancer data. (e-f) Rough (e) and fine (f) screening on mouse visual cortex data. (g-h) Rough (g) and fine (h) screening on mouse hypothalamus data.

Supplementary Figure S17. SpaSNE embeddings on the four datasets with different perplexity values (ppl). Three perplexity values were applied on each dataset: ppl = 5, 50, 100. The default value in SpaSNE is 50. (a-d) Results on human breast cancer dataset (a), human prostate cancer dataset (b), mouse visual cortex dataset (c), and mouse hypothalamus dataset (d).

Supplementary Figure S1. Images of the four spatially resolved profiling data.

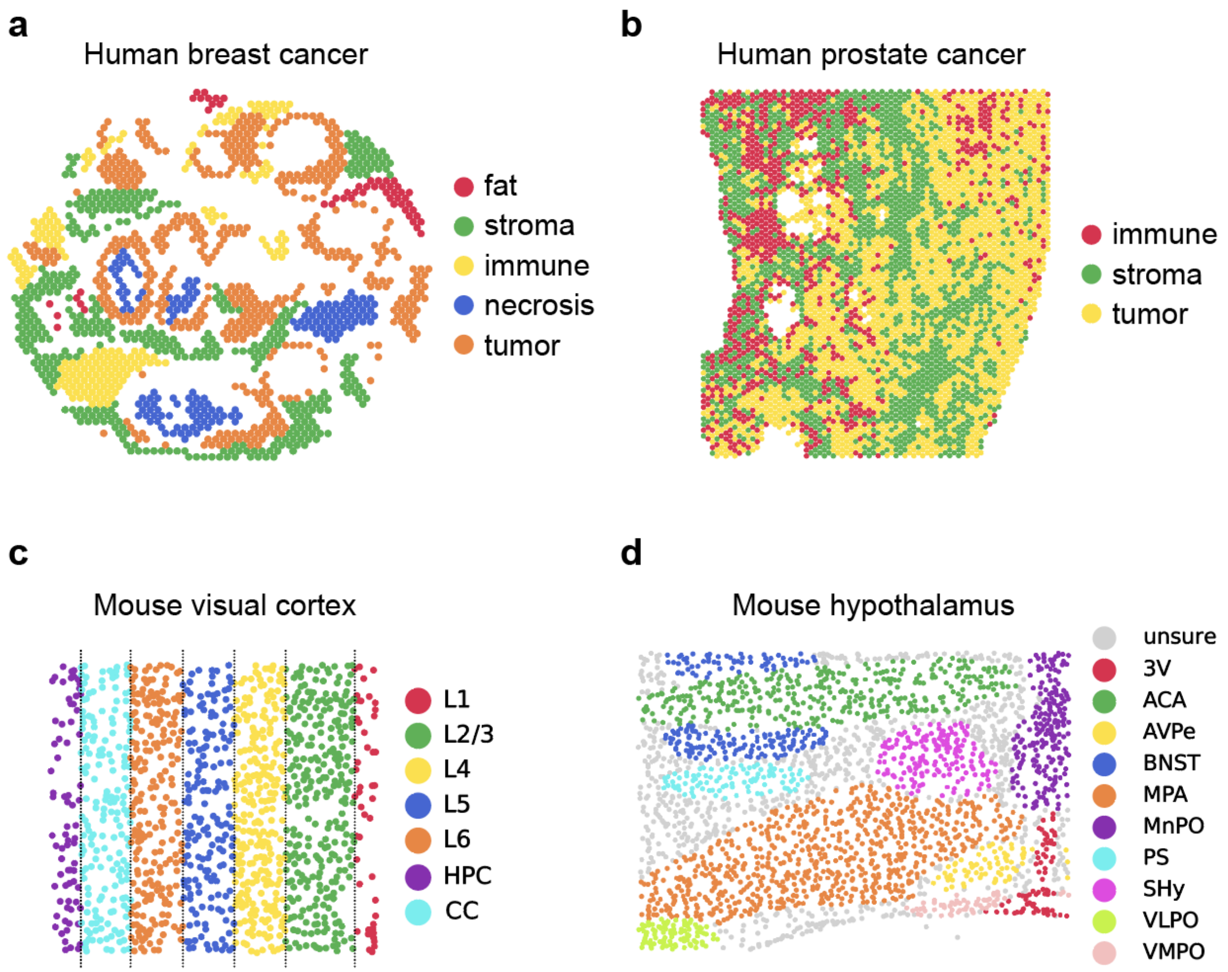

Supplementary Figure S2. Differential gene expressions of selected cell types in human breast cancer tissue.

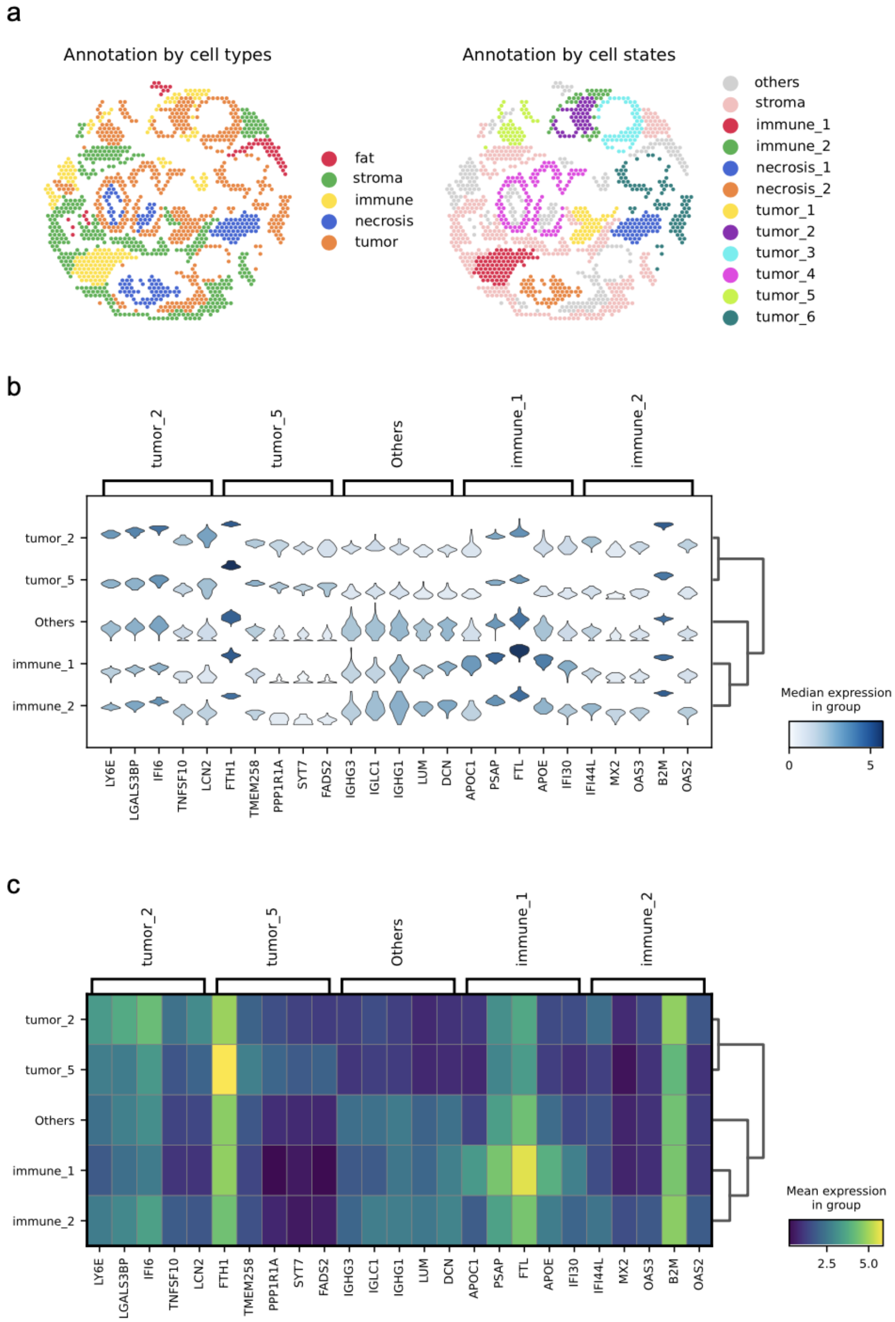

Supplementary Figure S3. Comparison of two tumor subtypes.

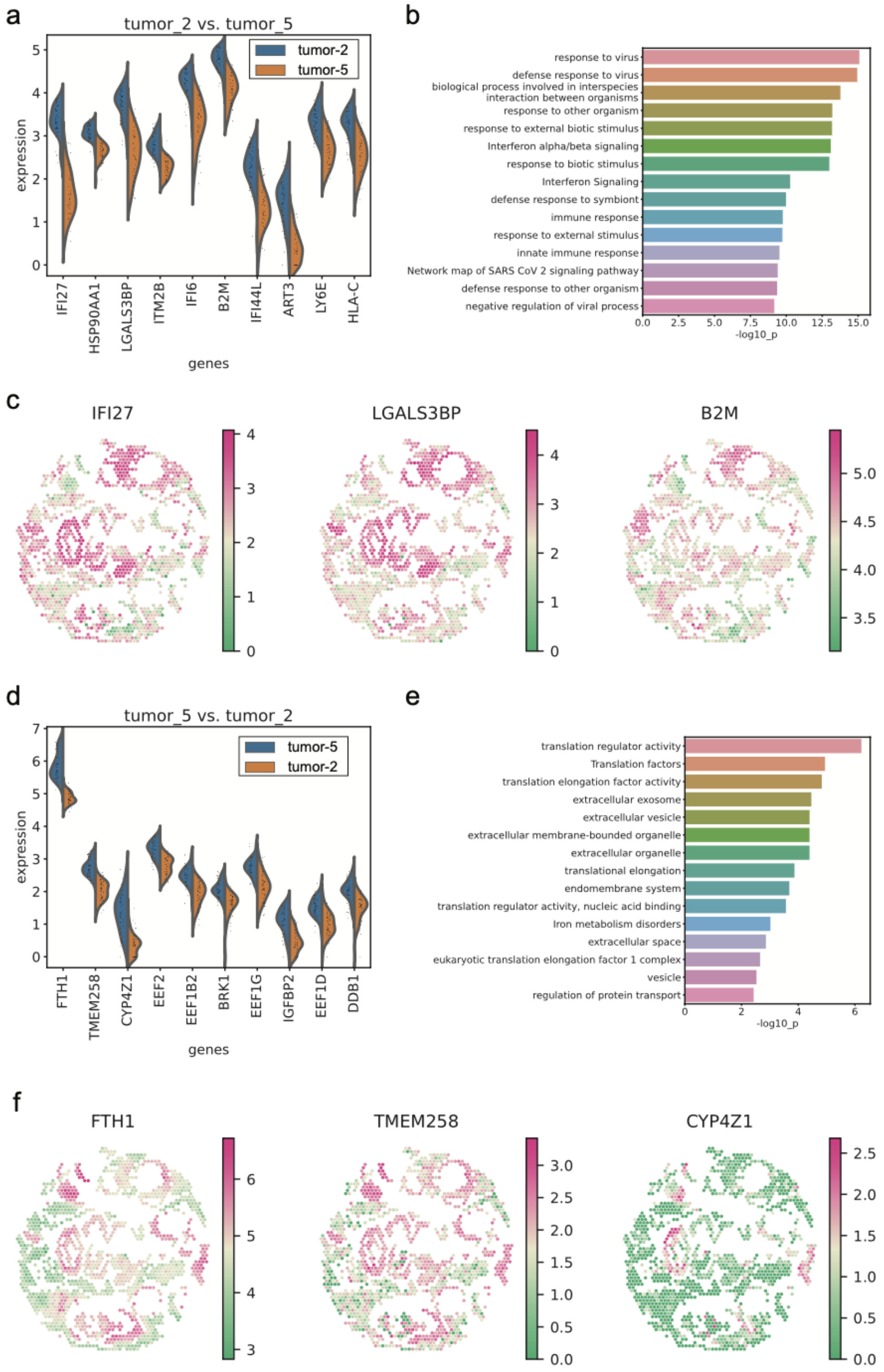

Supplementary Figure S4. Comparison of two immune subtypes.

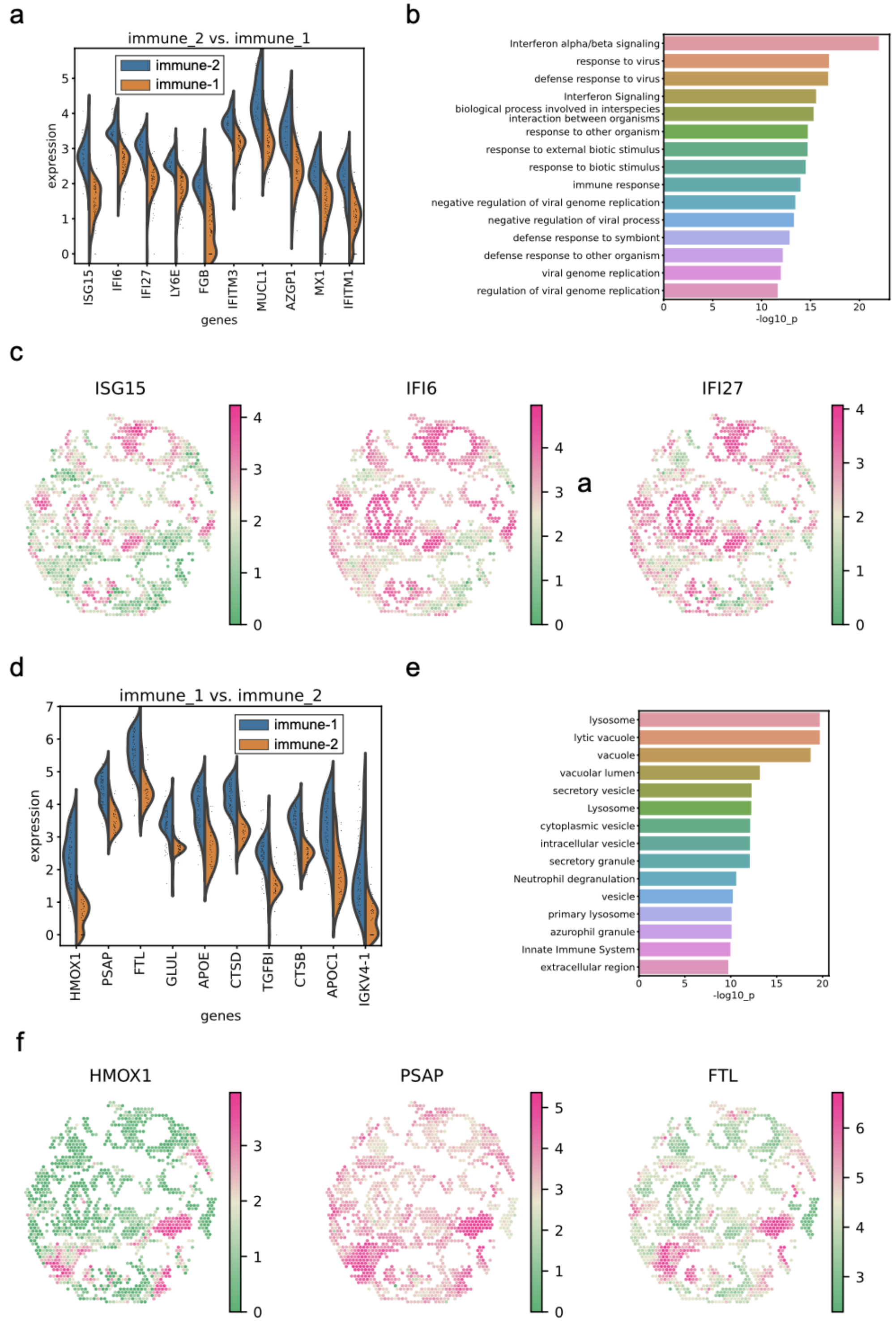

Supplementary Figure S5. Gene expression variability in different cell states.

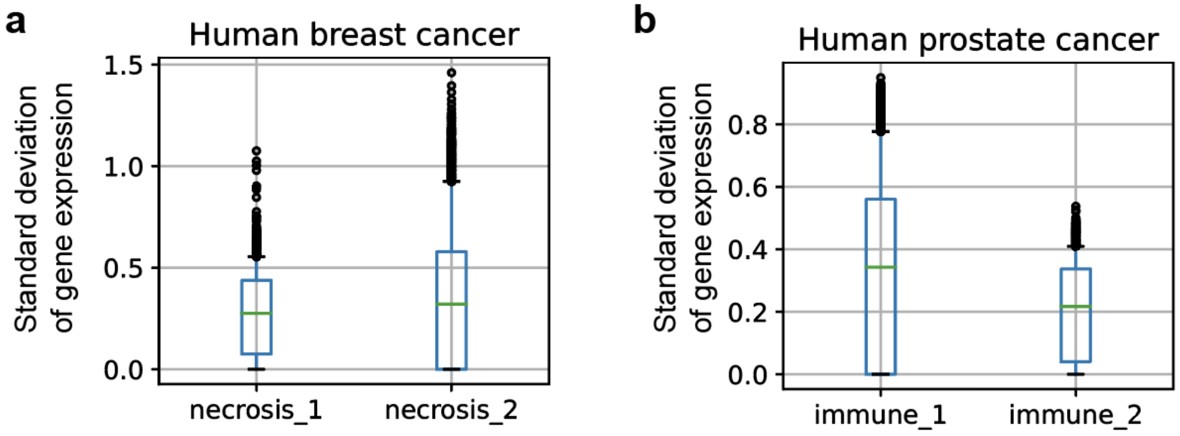

Supplementary Figure S6. SpaSNE results on human breast cancer with extreme parameters.

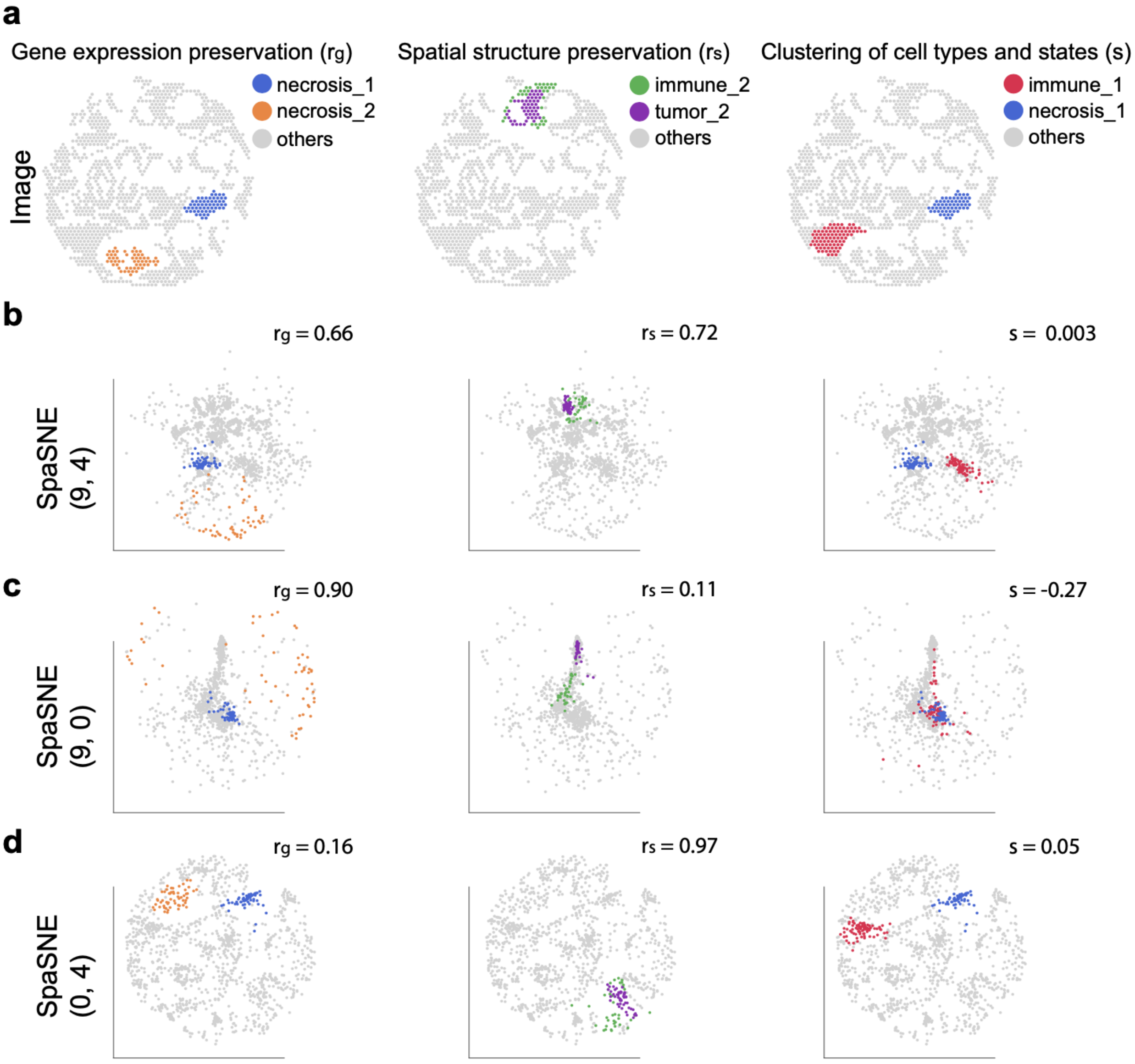

Supplementary Figure S7. Differential gene expressions of selected cell clusters in human prostate cancer tissue.

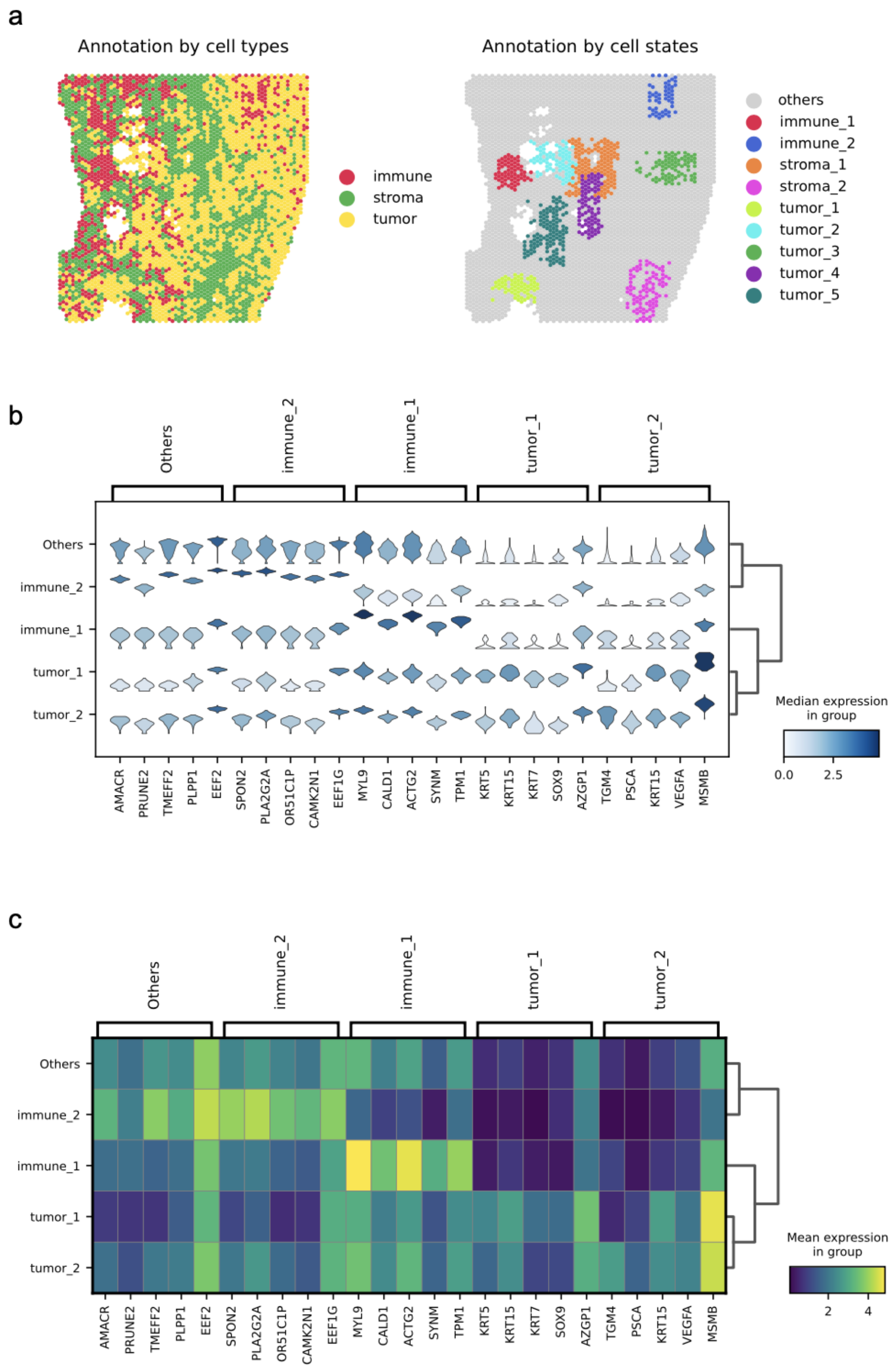

Supplementary Figure S8. Comparison of two immune subtypes.

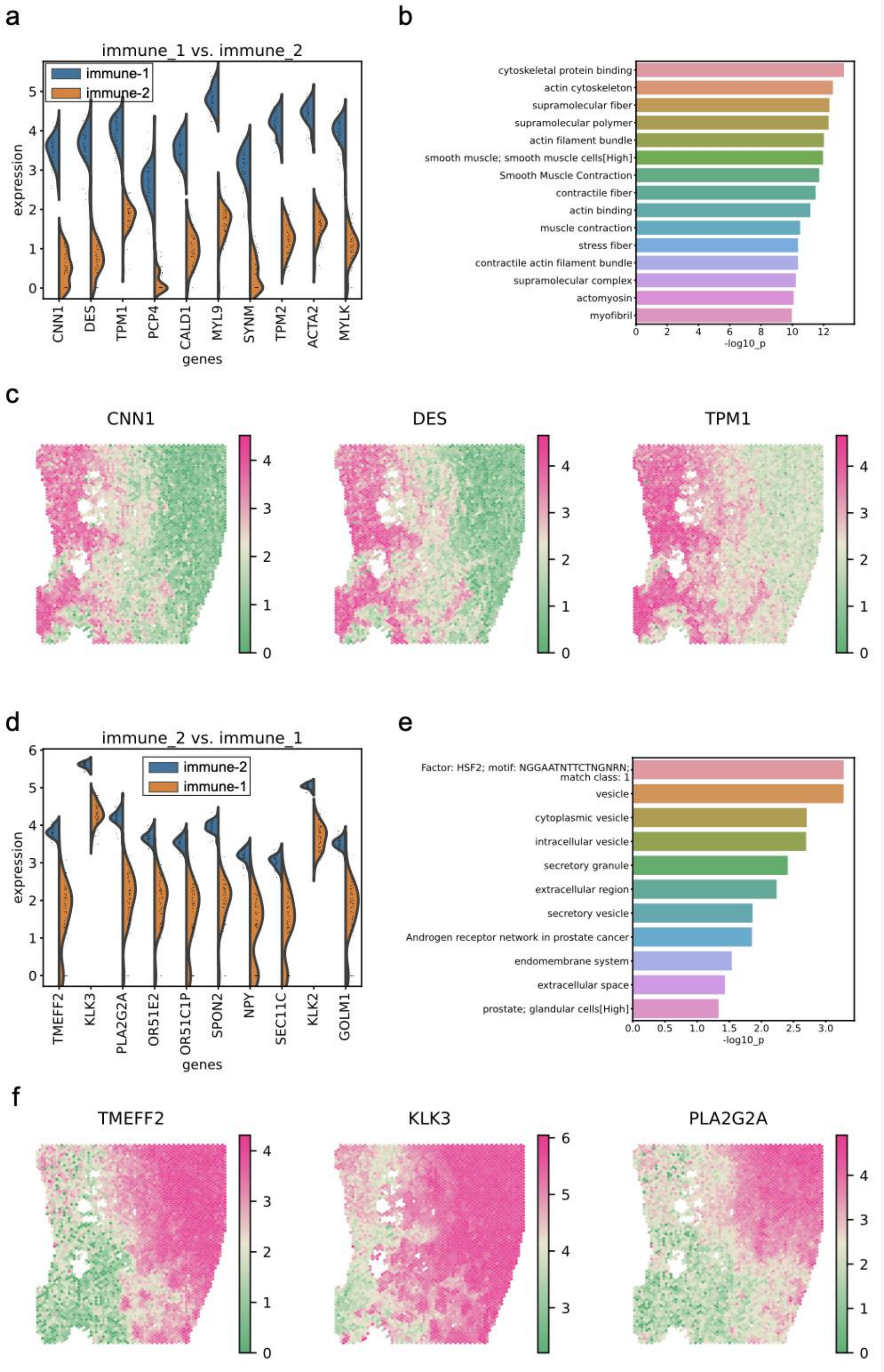

Supplementary Figure S9. Differential gene expressions of seven layers in mouse visual cortex tissue.

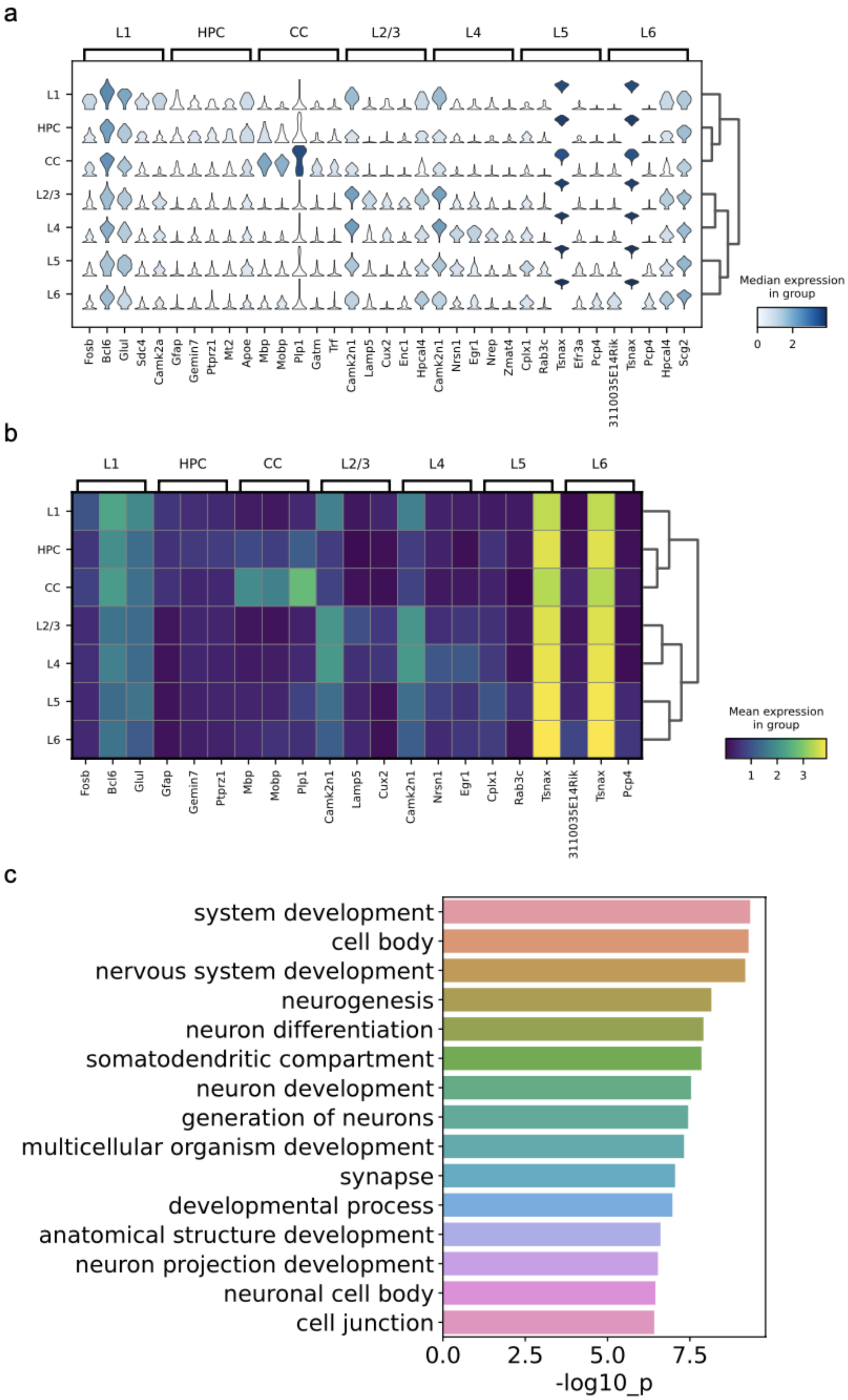

Supplementary Figure S10. Differentially expressed genes from comparison between each layer type and the rest layers in mouse visual cortex data.

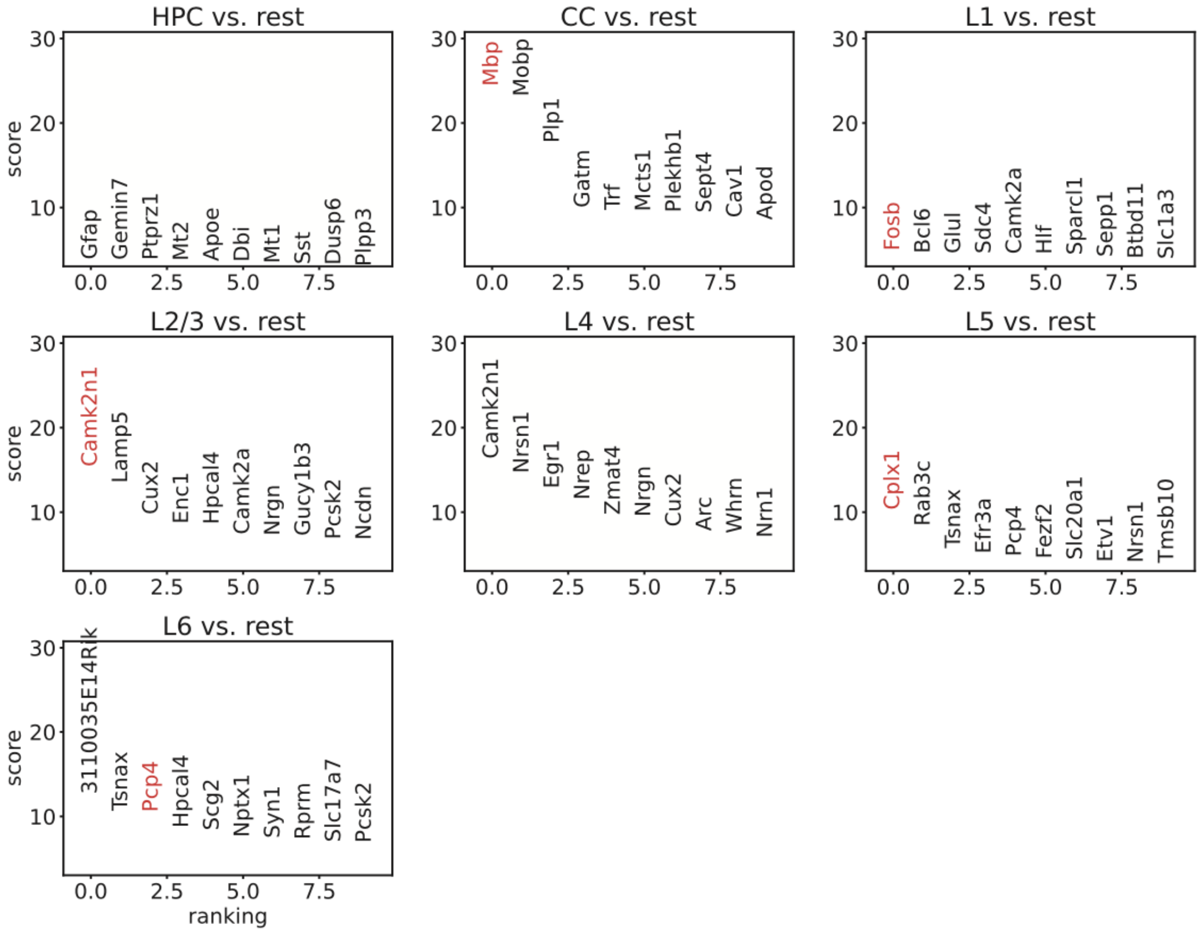

Supplementary Figure S11. Differential gene expressions of seven layers in mouse hypothalamus tissue.

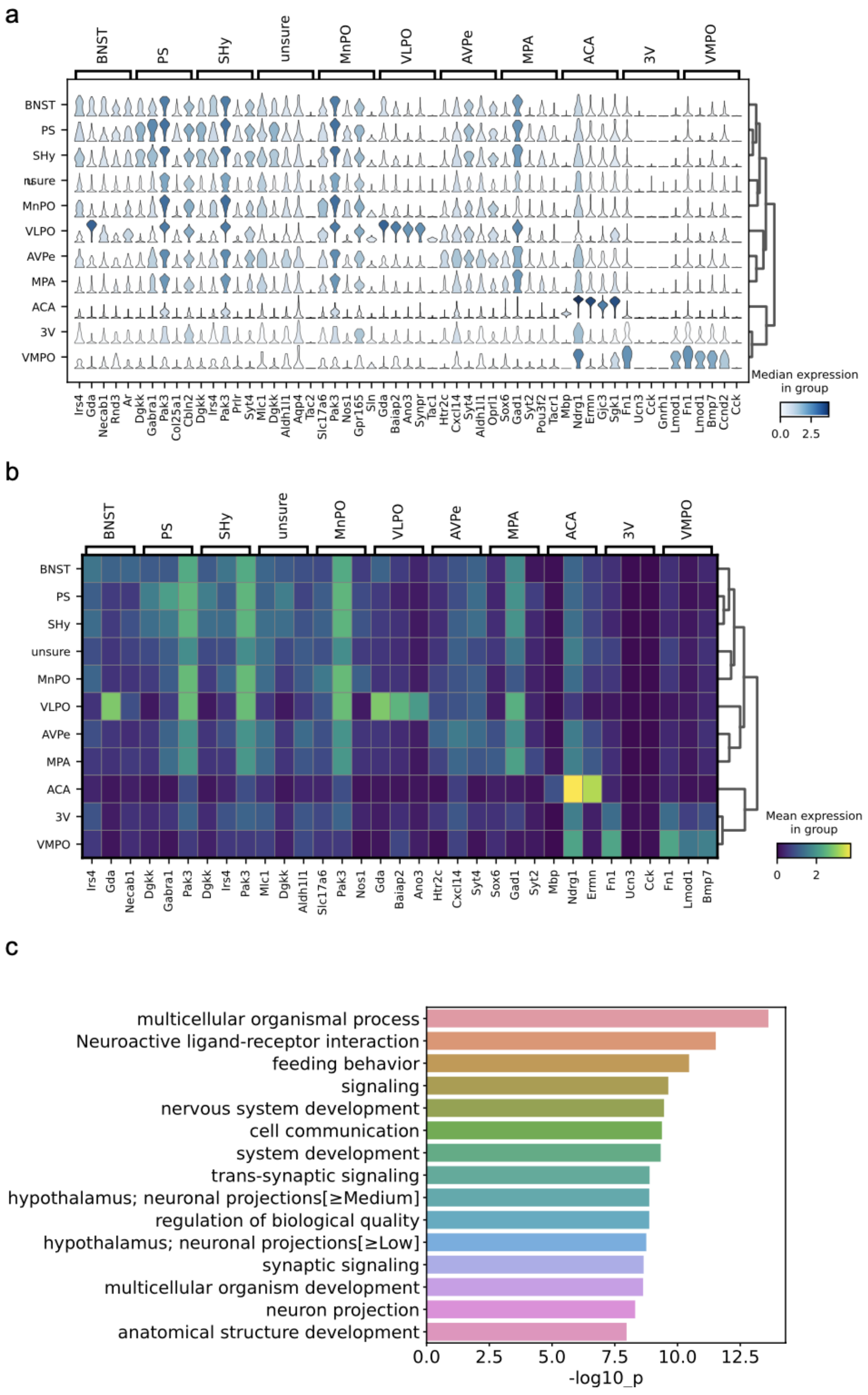

Supplementary Figure S12. Differentially expressed genes from comparison between each layer type and the rest layers in mouse hypothalamus data.

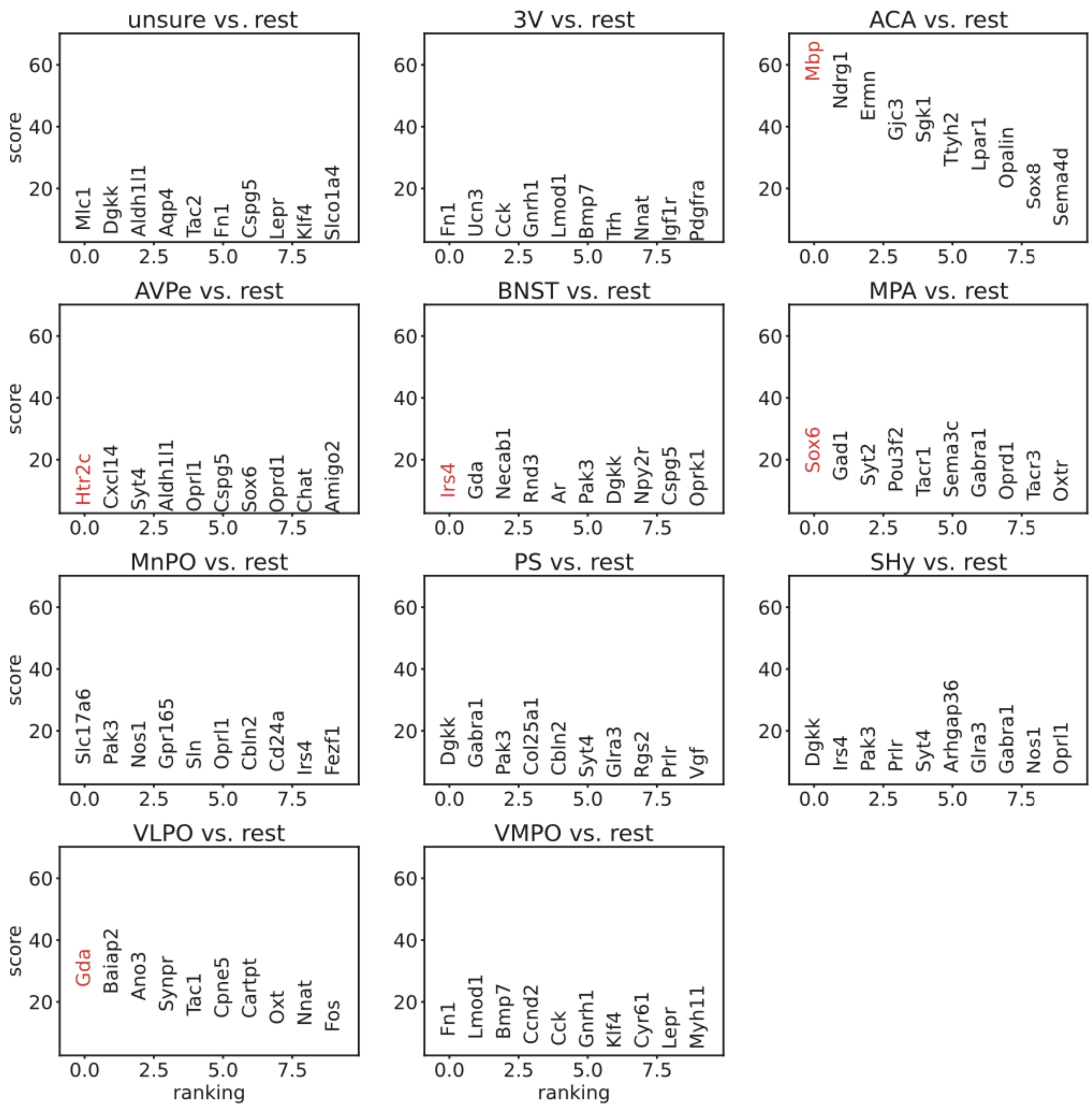

Supplementary Figure S13. Comparison between UMAP, MultiMAP, and SpaSNE in preserving spatial organization of cells.

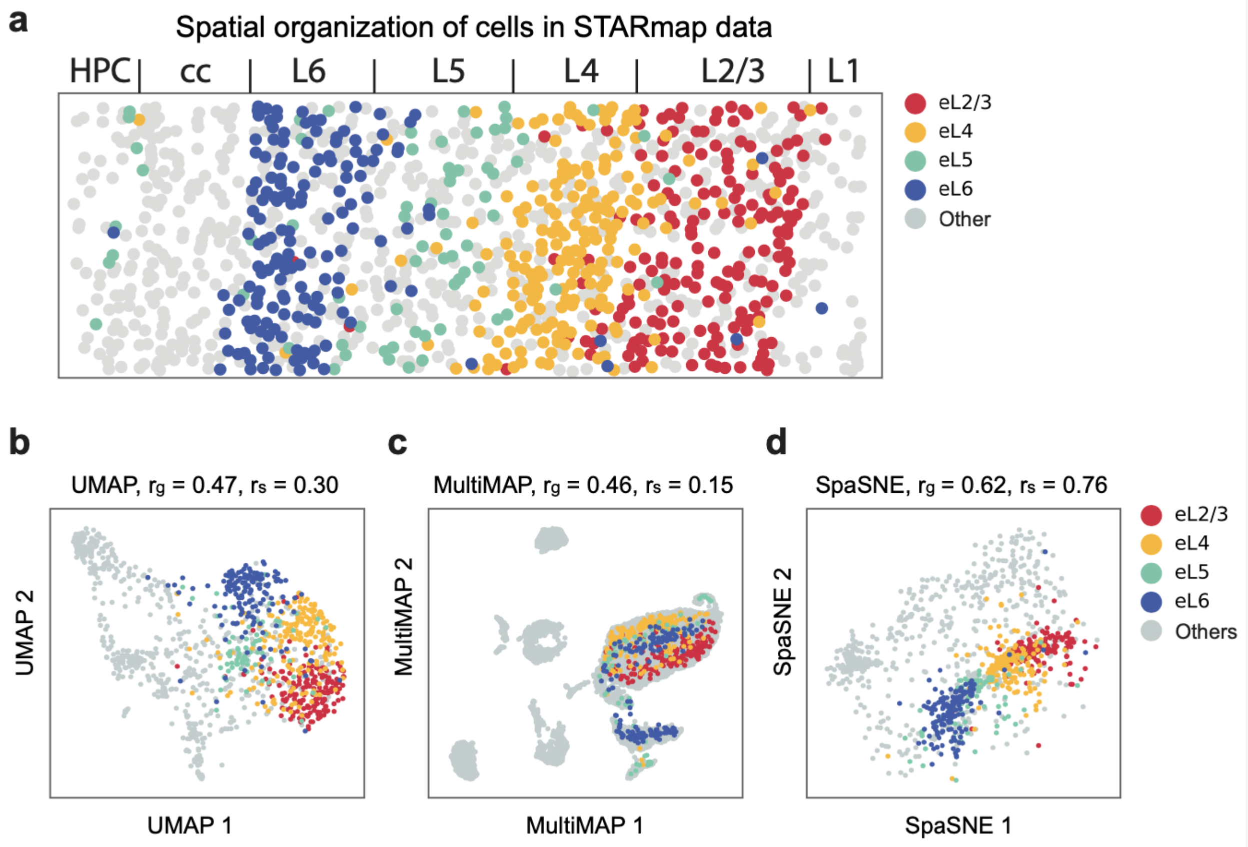

Supplementary Figure S14. Comparison of SpaSNE with SpatialPCA (d = 2) on visualization of the four datasets.

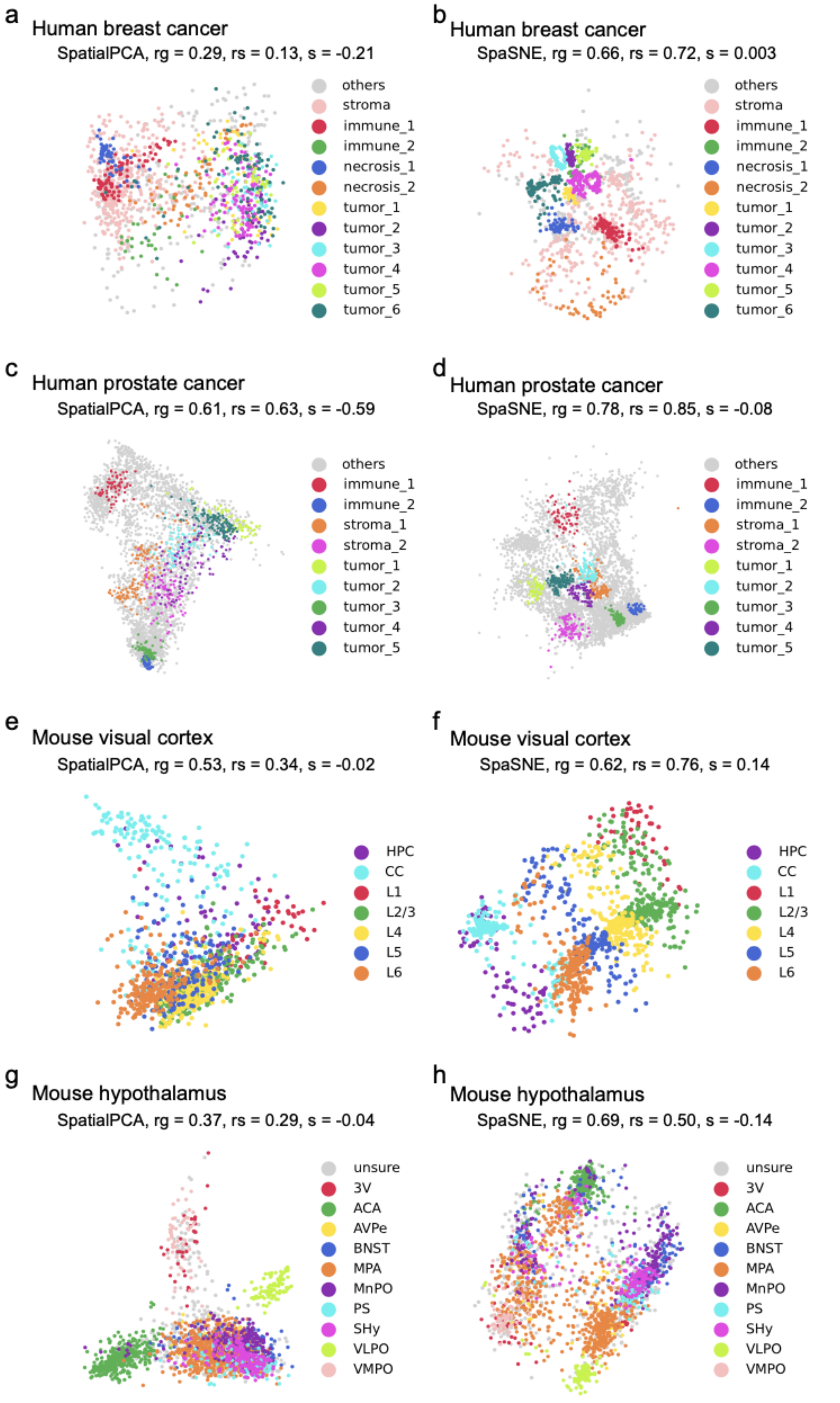

Supplementary Figure S15. Embedding performances of SpaSNE using metric of trustworthiness (tw).

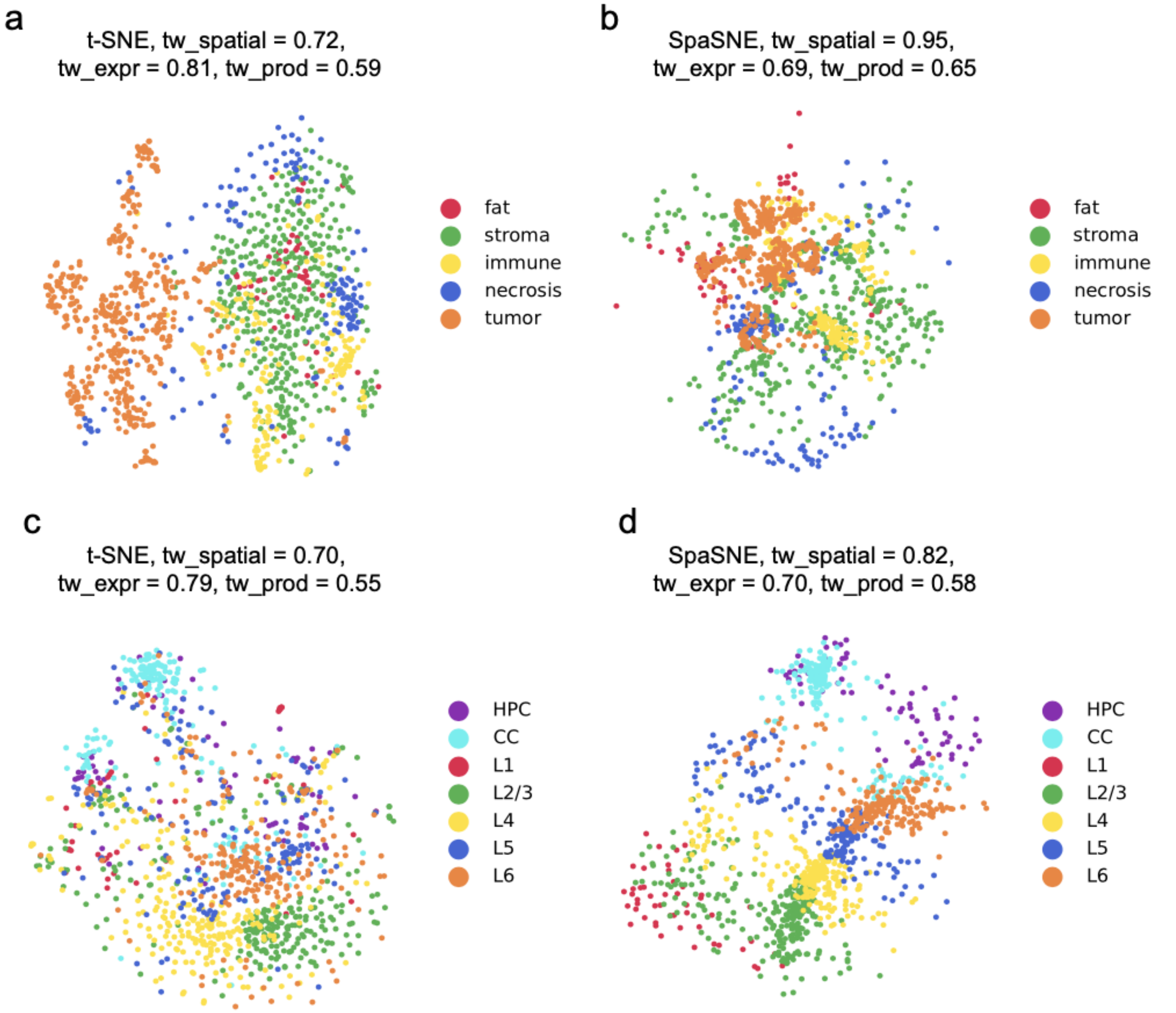

Supplementary Figure S16. Rough and fine screenings of parameters α and β of SpaSNE on the four datasets.

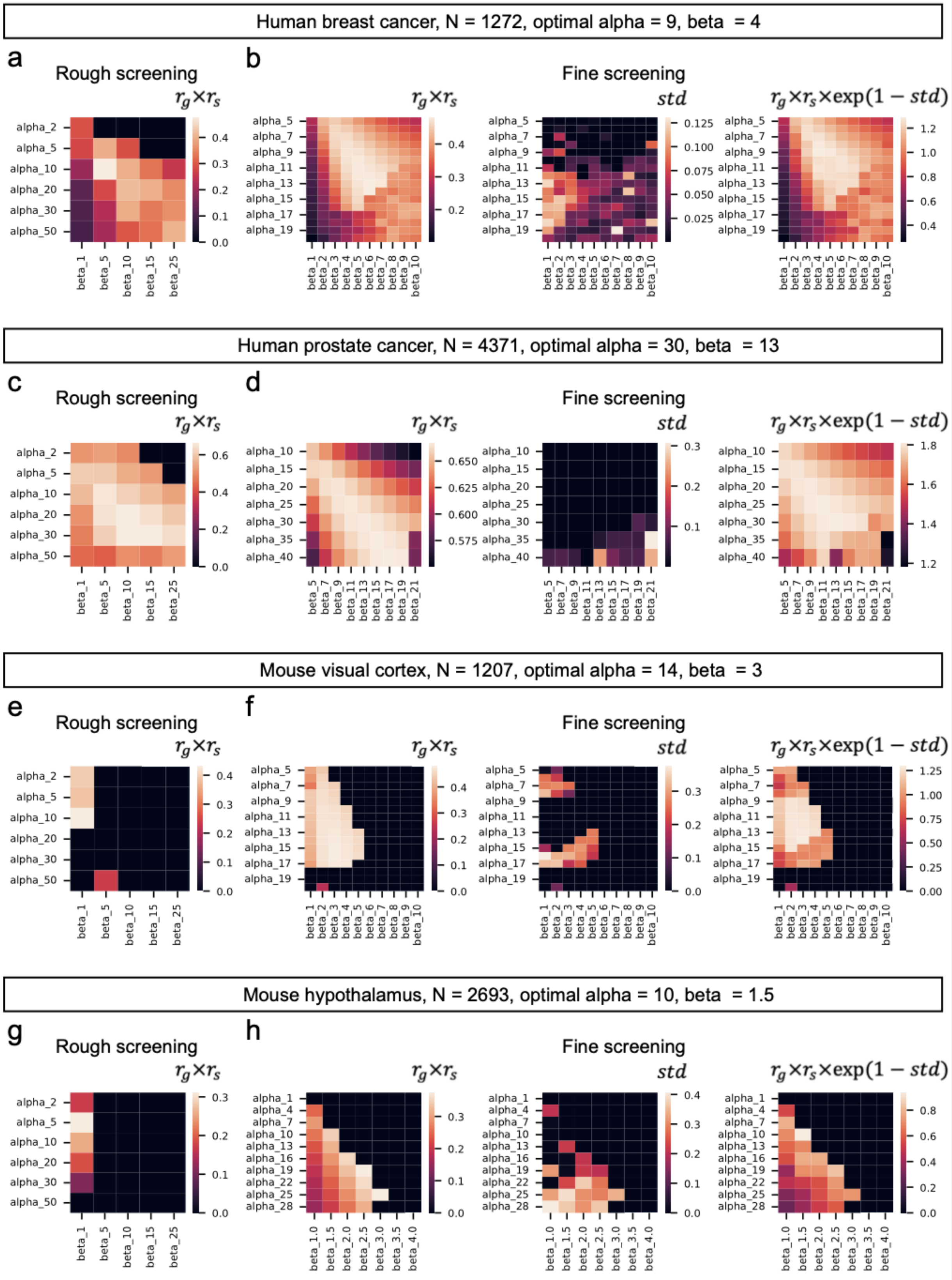

Supplementary Figure S17. SpaSNE embeddings on the four datasets with different perplexity values (ppl).

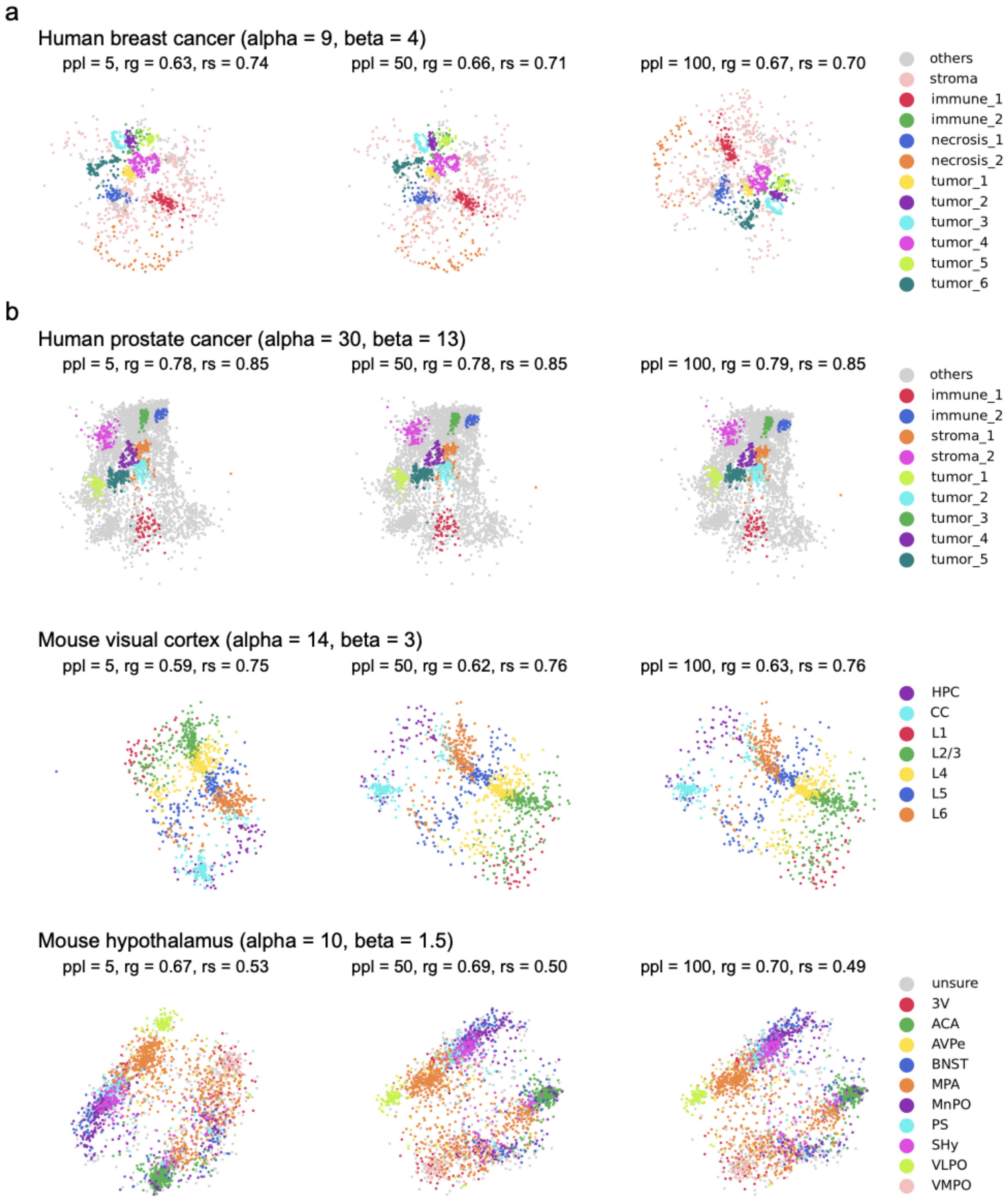

Supplement: giaf002_Supplement_Files [file giaf002_supplement_files.zip › Supplementary Figures.pdf]
