# Supplementary material for: Dimensionality reduction for visualizing spatially resolved profiling data using SpaSNE
Source: Gigascience. 2025 Feb 17;14:giaf002. doi: 10.1093/gigascience/giaf002 (PMC11831803; doi:10.1093/gigascience/giaf002)
Supplement: giaf002_GIGA-D-24-00148 [file giaf002_giga-d-24-00148.pdf]

GigaScience

Dimensionality reduction for visualizing spatially resolved profiling data using SpaSNE

--Manuscript Draft--

Manuscript Number:	GIGA-D-24-00148
Full Title:	Dimensionality reduction for visualizing spatially resolved profiling data using SpaSNE
Article Type:	Research
Funding Information:	
Abstract:	Background<br>Spatially resolved profiling technologies to quantify transcriptomes, epigenomes, and proteomes have been emerging as groundbreaking methods for comprehensive molecular characterizations. Dimensionality reduction and visualization is an essential step to analyze and interpret spatially resolved profiling data. However, state-of-the-art dimensionality reduction methods for single cell sequencing data, such as the t-SNE and UMAP, were not tailored for spatially resolved profiling data.<br>Findings<br>Here we developed a spatially resolved t-SNE (SpaSNE) method to integrate both spatial and molecular information. We applied it to a variety of public spatially resolved profiling datasets that were generated from three experimental platforms and consisted of cells from different diseases, tissues, and cell types. To compare the performances of SpaSNE, t-SNE, and UMAP, we applied them to four spatially resolved profiling datasets obtained from three distinct experimental platforms (Visium, STARmap, and MERFISH) on both diseased and normal tissues. Comparisons between SpaSNE and these state-of-the-art approaches reveal that SpaSNE achieves more accurate and meaningful visualization that better elucidates the underlying spatial and molecular data structures.<br>Conclusions<br>This work demonstrates the broad application of SpaSNE for reliable and robust interpretation on cell types based on both molecular and spatial information, which can set the foundation for many subsequent analysis steps, such as differential gene expression and trajectory or pseudotime analysis on the spatially resolved profiling data.
Corresponding Author:	Lin Xu, Ph.D.<br>UT Southwestern: The University of Texas Southwestern Medical Center<br>Dallas, TX UNITED STATES
Corresponding Author Secondary Information:	
Corresponding Author's Institution:	UT Southwestern: The University of Texas Southwestern Medical Center
Corresponding Author's Secondary Institution:	
First Author:	Lin Xu, Ph.D.
First Author Secondary Information:	
Order of Authors:	Lin Xu, Ph.D.
	Yuansheng Zhou, PhD
	Chen Tang, PhD
	Xue Xiao, PhD
	Xiaowei Zhan, PhD
	Tao Wang, PhD
	Guanghua Xiao, PhD
Order of Authors Secondary Information:	
Additional Information:	

Powered by Editorial Manager® and ProduXion Manager® from Aries Systems Corporation

Question	Response
Are you submitting this manuscript to a special series or article collection?	No
**Experimental design and statistics**<br><br>Full details of the experimental design and statistical methods used should be given in the Methods section, as detailed in our Minimum Standards Reporting Checklist. Information essential to interpreting the data presented should be made available in the figure legends.<br><br>Have you included all the information requested in your manuscript?	Yes
**Resources**<br><br>A description of all resources used, including antibodies, cell lines, animals and software tools, with enough information to allow them to be uniquely identified, should be included in the Methods section. Authors are strongly encouraged to cite Research Resource Identifiers (RRIDs) for antibodies, model organisms and tools, where possible.<br><br>Have you included the information requested as detailed in our Minimum Standards Reporting Checklist?	Yes
**Availability of data and materials**<br><br>All datasets and code on which the conclusions of the paper rely must be either included in your submission or deposited in publicly available repositories (where available and ethically appropriate), referencing such data using a unique identifier in the references and in the "Availability of Data and Materials" section of your manuscript.	Yes

Have you have met the above requirement as detailed in our Minimum Standards Reporting Checklist?	

Powered by Editorial Manager® and ProduXion Manager® from Aries Systems Corporation

Manuscript Click here to access/download;Manuscript;Manuscript.pdf

Dimensionality reduction for visualizing spatially resolved profiling data using SpaSNE

Yuansheng Zhou[1], Chen Tang[1], Xue Xiao[1], Xiaowei Zhan[1,2], Tao Wang[1,2], Guanghua Xiao[1,3 *], Lin Xu[1,4 *]

[1]Quantitative Biomedical Research Center, Peter O'Donnell Jr. School of Public Health, University of Texas Southwestern Medical Center, Dallas, TX, USA
[2]Center for the Genetics of Host Defense, University of Texas Southwestern Medical Center, Dallas, TX, USA
[3]Department of Bioinformatics, University of Texas Southwestern Medical Center, Dallas, TX 75390, USA
[4]Department of Pediatrics, Division of Hematology/Oncology, University of Texas Southwestern Medical Center, Dallas, TX, USA

*Corresponding Authors:
Guanghua Xiao, PhD (Guanghua.Xiao@UTSouthwestern.edu)
Lin Xu, PhD (Lin.Xu@UTSouthwestern.edu)

Abstract

Background

Spatially resolved profiling technologies to quantify transcriptomes, epigenomes, and proteomes have been emerging as groundbreaking methods for comprehensive molecular characterizations. Dimensionality reduction and visualization is an essential step to analyze and interpret spatially resolved profiling data. However, state-of-the-art dimensionality reduction methods for single cell sequencing data, such as the t-SNE and UMAP, were not tailored for spatially resolved profiling data.

Results

Here we developed a spatially resolved t-SNE (SpaSNE) method to integrate both spatial and molecular information. We applied it to a variety of public spatially resolved profiling datasets that were generated from three experimental platforms and consisted of cells from different diseases, tissues, and cell types. To compare the performances of SpaSNE, t-SNE, and UMAP, we applied them to four spatially resolved profiling datasets obtained from three distinct experimental platforms (Visium, STARmap, and MERFISH) on both diseased and normal tissues. Comparisons between SpaSNE and these state-of-the-art approaches reveal that SpaSNE achieves more accurate and meaningful visualization that better elucidates the underlying spatial and molecular data structures.

Conclusions

This work demonstrates the broad application of SpaSNE for reliable and robust interpretation on cell types based on both molecular and spatial information, which can set the foundation for many subsequent analysis steps, such as differential gene expression and trajectory or pseudotime analysis on the spatially resolved profiling data.

Keywords

Spatially resolved omics, dimensionality reduction, low dimensional visualization, molecular data structure, spatial organization of cells.

Background

Due to the capability to uncover spatial organization and intercellular communication, spatially resolved profiling technologies on DNA, RNA, and proteins have become one of the latest frontiers for cutting edge researches in both basic biology and medicine. While a large number of distinct spatial profiling platforms have been developed so far, a recent review[1] proposed that spatially resolved profiling technologies can be primarily categorized as two major directions: imaging-based approaches (e.g. STARMap[2] and seqFISH[3]) and NGS-based approaches (e.g. Slide-seq[4] and Visium by 10X Genomics). These innovative technologies are promising to transform the way that we think about cell differentiation, tissue development, and disease progression in a spatial fashion, and therefore could lead to novel discoveries on elucidating detailed cellular and molecular mechanisms, as well as identifying effective biomarkers and therapeutic targets[1, 5, 6].

Dimensionality reduction and visualization is an essential step to analyze and interpret the spatially resolved profiling data from DNA, RNA, and proteins[7, 8]. Different from the clustering methods (e.g. BayesSpace[9] or SpaGCN[10]), the aim of developing dimensionality reduction and visualization approaches for spatially resolved profiling data is to visualize the cells in a low dimensional space while maintaining the underlying molecular and spatial data structures (e.g. gene expression variabilities of different cell types[11, 12] and spatial closeness of various cell types[13, 14]). Among the published methods, the t-distributed stochastic neighbor embedding (t-SNE)[15-17] and the uniform manifold approximation and projection (UMAP)[18] have been the most widely used tools for dimensionality reduction and visualization of single cell sequencing data. Compared with the linear dimensionality reduction methods such as principal component analysis (PCA), both t-SNE and UMAP have great advantages in reliably visualizing cell clusters in single cell sequencing datasets[15-20]. Some recent variants of t-SNE and UMAP further extended the power of these two algorithms in revealing gene expression variabilities of single

cells[12] or visualizing multimodal omics data[21]. Therefore, recent spatially resolved profiling studies have been using either t-SNE[22-28] or UMAP[2, 13, 14, 29-32] for data visualization. However, different from routine single-cell omics data with molecular information alone, the most unique feature of spatially resolved profiling data is that it contains both molecular information from next generation sequencing and spatial organization information from images. The current design of t-SNE or UMAP does not leverage both molecular and spatial information simultaneously for analyzing spatially resolved profiling data. Therefore, new dimensionality reduction and visualization algorithms that are able to integrate both molecular and spatial information are in urgent need, because they can visualize cell clusters in the context of tissues' spatial organization and are promising to help uncover more biological insights in the studies of cellular communications[13, 22-24, 33-35] or developmental trajectories[14, 29-31, 36] in the spatial fashion.

Here we developed a spatially resolved t-SNE (SpaSNE) method by adapting t-SNE to more adequately leverage both molecular and spatial information in the spatially resolved profiling data. SpaSNE could provide a comprehensive low dimensional visualization that better preserves the molecular data structure and spatial organization of cells simultaneously. Because spatially resolved gene expression profiling technologies are well developed so far, in this study we will mainly use spatially resolved gene expression datasets to demonstrate the utility of SpaSNE. To compare the performances of SpaSNE, t-SNE, and UMAP, we applied them to four spatially resolved profiling datasets obtained from three distinct experimental platforms (Visium, STARmap, and MERFISH) on both diseased and normal tissues. The analytical results showed that SpaSNE achieves the most accurate embedding and most meaningful visualization of the spatially resolved profiling data.

Methods

Datasets

The four spatial gene expression datasets used in the manuscript were presented in **Supplementary Table 1** (the details are also included in the "Data availability" section). For the human breast cancer data, we took the 1272 spots with known manual annotations; for mouse hypothalamus data, we took the left side of the whole slide, which contains 2693 cells. The annotations of the four datasets were provided in **Supplementary Tables 2-5**.

SpaSNE's embedding

The t-distributed stochastic neighbor embedding (t-SNE) algorithm[16] has been widely used in nonlinear dimensionality reduction and visualization for the gene expression data. Given a dataset with N spots or cells with gene expression vectors $(x_1, x_2, \dots, x_N)$, t-SNE defines the pairwise similarities of data points p_{ij} by the following form:

$$p_{j|i} = \frac{\exp\left(-\left\|x_i - x_j\right\|^2 / 2\sigma_i^2\right)}{\sum_{i \neq j} \exp\left(-\left\|x_i - x_j\right\|^2 / 2\sigma_i^2\right)} \quad (1)$$

$$p_{ij} = \frac{p_{i|j} + p_{j|i}}{2N} \quad (2)$$

In the low-dimensional ($d = 2\ or\ 3$) representation, the pairwise similarities of points $(y_1, y_2, \dots, y_N)$ is defined as:

$$q_{ij} = \frac{\left(1 + \left\|y_i - y_j\right\|^2\right)^{-1}}{\sum_{i \neq j} \left(1 + \left\|y_i - y_j\right\|^2\right)^{-1}} \quad (3)$$

The loss function L_t is defined as the discrepancy between data and embedding points, which is measured by the Kullback–Leibler (KL) divergence of the pairwise similarities:

$$L_t = KL(P||Q) = \sum_i \sum_j p_{ij} \log\left(\frac{p_{ij}}{q_{ij}}\right) \quad (4)$$

The loss function L_t is minimized to achieve the optimal low dimensional representation of the data. The gradient of the loss function L_t with respect to y_i is calculated as:

$$\frac{\partial L_t}{\partial y_i} = 4 \sum_j (p_{ij} - q_{ij})(y_i - y_j)\left(1 + \|y_i - y_j\|^2\right)^{-1} \quad (5)$$

With this definition, t-SNE only preserves local structure of the gene expression because Eq. 1 and 3 are only sensitive to small-scale distance variations.

The spatially resolved profiling data provides the spatial positions of spots or cells $(z_1, z_2, \dots, z_N)$, which cannot be used in t-SNE. SpaSNE improves t-SNE by introducing two new loss functions to preserve both the large-scale gene expression distances and the spatial distances of data. The first loss function measures the KL divergence L_g between the large-scale gene expression distances $\hat{p}_{ij}$ and the large-scale embedding distances $\hat{q}_{ij}$:

$$\hat{p}_{ij} = \frac{1 + \|x_i - x_j\|^2}{\sum_{i \neq j}\left(1 + \|x_i - x_j\|^2\right)} \quad (6)$$

$$\hat{q}_{ij} = \frac{1 + \|y_i - y_j\|^2}{\sum_{i \neq j}(1 + \|y_i - y_j\|^2)} \quad (7)$$

$$L_g = KL(\hat{P}||\hat{Q}) = \sum_i \sum_j \hat{p}_{ij} \log\left(\frac{\hat{p}_{ij}}{\hat{q}_{ij}}\right) \quad (8)$$

Introducing L_g helps preserve large-scale inter-cluster structure of gene expression because Eq. 6 and 7 are sensitive to large-scale distance variations. The second loss function measures the KL divergence L_s between the large-scale spatial distances $\hat{s}_{ij}$ from image and the large-scale embedding distances $\hat{q}_{ij}$:

$$\hat{s}_{ij} = \frac{1 + \|z_i - z_j\|^2}{\sum_{i \neq j}(1 + \|z_i - z_j\|^2)} \quad (9)$$

$$L_s = KL(\hat{S}||\hat{Q}) = \sum_i \sum_j \hat{s}_{ij} \log\left(\frac{\hat{s}_{ij}}{\hat{q}_{ij}}\right) \quad (10)$$

Integrating the two new loss functions to the original loss function L_t of t-SNE, we get the total loss function with two weighting parameters α and β:

$$L_{total} = L_t + \alpha L_g + \beta L_s \quad (11)$$

The gradient of the loss function L_{total} has a simple form:

$$\frac{\partial L_t}{\partial y_i} = 4 \sum_j [(p_{ij} - q_{ij}) - \alpha(\hat{p}_{ij} - \hat{q}_{ij}) - \beta(\hat{s}_{ij} - \hat{q}_{ij})](y_i - y_j)\left(1 + \|y_i - y_j\|^2\right)^{-1} \quad (12)$$

Quantitative evaluation of the embedding quality

Three quantitative measures were defined to evaluate the embedding quality: (1) Pearson correlation coefficient (r_1) between the pairwise Euclidean distances of the gene expressions and the embedding distances of points, which was used to measure the preservations of the gene expressions; (2) Pearson correlation coefficient (r_2) between pairwise spatial distances and embedding distances of points, which was used to measure the preservation of the spatial structure; (3) Silhouette score (s) which was used to measure the consistency between the clusters of embedding points and the ground-truth annotation of clusters. r_1 and r_2 were calculated using the "scipy" python package, and Silhouette score was calculated using the "sklearn" python package.

Parameters of visualization algorithms

We applied three algorithms for visualization of the spatially resolved gene expression profiling data: t-SNE, UMAP and SpaSNE. We ran UMAP using the "umap" Python package[18] with default parameters. For SpaSNE, we screened the optimal combination of parameters α and β that gave the best performances in terms of the gene expression preservation (r_1), spatial structure preservation (r_2), and the clustering quality (s), as well as the reproducibility of the embeddings. The two parameters α and β in Eq. 11 represent the weights of the large-scale gene expression' loss function L_g and the spatial loss function L_s. Therefore, a larger α leads to a larger r_1 and a smaller r_2, a larger β leads to a larger r_2 and a smaller r_1. Both parameters collectively affect the clustering quality s. Thus, a proper ratio between α and β is required to give a good preservation of both the gene expression structure and the spatial structure, as well as a good clustering quality. In addition to the ratio of α and β, we found that the magnitude of α influenced the reproducibility of the embedding. A larger α resulted in a higher chance of failure in the embedding, probably because the contribution of the local cost function L_t in the original t-SNE

was weakened by a large α (Eq. 11) and the embedding became more unstable, especially when the data size is small. Based on the above considerations and experiences on four real datasets, we give the following recommendations for setting α and β: $\alpha \in [6, 15]$, $\beta \in [1, 7.5]$, $\alpha/\beta \geq 2$. The default parameters for SpaSNE were set as $\alpha = 8,\ \beta = 2$ if spatial information is available, and $\alpha = 8,\ \beta = 0$ if the spatial information is not provided . The parameters for the four datasets were shown in Supplementary Table 1. The perplexity values in SpaSNE and t-SNE were set as 50.

Results

Overview of SpaSNE

As one of the most widely used dimensionality reduction tools for single cell sequencing data analysis, t-SNE has recently been adopted to analyze spatially resolved gene expression profiling data. It takes the gene expression data as input and performs dimensionality reduction and visualization for the data. The primary purpose of t-SNE is to preserve the small-scale local structure of gene expression (i.e. cell clustering) by minimizing the loss function L_{tsne}, which is the Kullback-Leibler (KL) divergence between similarities of data points and embedding points. Therefore, the t-SNE map was mainly used to generate a low dimensional visualization map that reliably displays the clustering of cells (**Fig. 1a**). SpaSNE extends the function of t-SNE by not only preserving the local structure of gene expression, but also maintaining the large-scale inter-cluster structure of gene expression and integrating spatial information of the cells. SpaSNE takes both gene expression data and spatial positions as input. It introduces two new loss functions to preserve large-scale gene expression distances and spatial distances respectively (see Methods). The contributions of these two loss functions are controlled by two independent parameters α and β, which can be adjusted by users to balance gene expression preservation and spatial structure preservation. With this adaptation, SpaSNE can generate a low dimensional visualization map that not only displays the clustering of cells as t-SNE does, but also reveals inter-cluster features in spatially resolved expression profiling data, including gene expression variabilities of different cell clusters, spatial organization of cell types and developmental trajectory of tissues (**Fig. 1b**). We will provide detailed examples to show all these applications of SpaSNE in the following sections. Because spatially resolved epigenomics and proteomics profiling technologies are now under development, here we focused on using available spatially resolved gene expression profiling datasets to demonstrate the utility of SpaSNE.

Application to the diseased breast tissue data based on the Visium spatial transcriptomics technology

We first analyzed spatially resolved transcriptomics data of the human breast cancer tissues from 10X Genomics data portal (**Supplementary Fig. 1a**). We extracted 1272 annotated spots from the original dataset and performed SpaSNE, t-SNE and UMAP embeddings to compare their performances on visualization of cells. We showed ten cell clusters, which were colored according to both cell types (based on pathological annotation from 10X Genomics data portal) and spatial locations in SpaSNE, t-SNE and UMAP embeddings, respectively (**Fig. 2a-c**). SpaSNE presented separated and compact clusters for most of the immune and tumor cells of different spatial locations (**Fig. 2a**). t-SNE and UMAP produced two large clusters that distinguished tumor and non-tumor cells based on gene expression information. However, they could not distinguish the cell clusters with distinct spatial locations. For example, t-SNE and UMAP both presented the six tumor clusters with different spatial locations (tumor 1-6) as one large, disperse cluster and therefore lost the spatial information for them (**Fig. 2b-c**). This result showed that SpaSNE could produce a more delicate visualization that distinguishes a larger number of cell clusters by leveraging both gene expression and spatial information. To comprehensively evaluate the performances of SpaSNE, t-SNE, and UMAP in a quantitative manner, we defined three quantitative measures: (1) Pearson correlation coefficient (r_g) between pairwise gene expression distances and embedding distances of points, which was used to measure gene expression preservation; (2) Pearson correlation coefficient (r_s) between pairwise spatial position distances and embedding distances of points, which was used to measure spatial structure preservation; (3) Silhouette score[37] (s) which was used to measure the consistency of clustering with the ground truth annotations. The comparison of the three algorithms showed that SpaSNE outperformed t-SNE and UMAP in all three measures (**Fig. 2d**).

The quantitative advantages of SpaSNE indicate better performances in revealing the underlying data structures. To demonstrate this, we highlighted several representative cell types with different spatial locations from the visualization maps in **Fig. 2a-c** to exam the performances of SpaSNE in detail (**Fig. 2e-g**). First, we highlighted two types of necrosis cells that have different levels of gene expression variabilities: necrosis 1 (blue) and necrosis 2 (orange) ("image" panel in **Fig. 2e**). The cells in necrosis 2 have a higher overall gene expression variability than the cells in necrosis 1 (**Supplementary Fig. 2a**). The difference in gene expression variability of these two cell clusters cannot be reflected in the image, but can be revealed in SpaSNE, t-SNE and UMAP maps (**Fig. 2e**). In SpaSNE map, the necrosis 2 cluster has a larger size and smaller point density than that of necrosis 1 (**Fig. 2e**), which is consistent with the smaller gene expression variability of necrosis 2 (**Supplementary Fig. 2a**). t-SNE and UMAP also displayed similar properties, but the differences of cluster sizes and point densities between the two clusters are not as big as in SpaSNE (**Fig. 2e**). This example showed that the SpaSNE map can better reveal the gene expression variabilities of different cell clusters, which cannot be displayed by image alone.

In addition, we want to highlight that the better performance of SpaSNE in revealing gene expression variabilities can be explained by the higher r_g value that reflects better preservation of gene expression distances (r_g = 0.63 in SpaSNE, 0.28 in t-SNE, and 0.24 in UMAP). When the gene expression preservation was tuned to be extremely high ($\alpha = 10, \beta = 0,$ r_g = 0.91, **Fig. S3c**), the difference of gene expression variabilities was even larger (**Fig. S3c**), but the spatial structure preservation became worse (r_s = 0.10, **Fig. S3c**). SpaSNE allows users to have the flexibility to adjust the preservation of gene expression and spatial structure according to their own research purposes.

Second, we highlighted two different cell types that were spatially close to each other: immune 2 (green) and tumor 2 (purple) ("image" panel in **Fig. 2f**). We observed that SpaSNE was able to preserve the relative spatial distances of these two cell populations by keeping them close to each other, while both t-SNE and UMAP displayed these two cell populations far away from each other without keeping the spatial contacts between them (**Fig. 2f**). The preservation of spatial organization in SpaSNE is due to the preservation of spatial distances (r_s = 0.76 in SpaSNE, 0.10 in t-SNE, and 0.11 in UMAP**)**. When the spatial distances preservation was tuned to be extremely high ($\alpha = 0, \beta = 5, r_s$ = 0.98, **Fig. S3d**), the spatial structure more approximated the image (**Fig. S3d**), but the gene expression preservation became worse (r_g = 0.15, **Fig. S3d**). This example indicated that SpaSNE could outperform t-SNE and UMAP in preserving spatial organization of cells in the micro-environment (e.g. in human cancers) without harming the capability in distinguishing distinct cell populations.

Third, we highlighted two different cell types in two spatially separated regions: immune 1 (red) and necrosis 1 (blue) ("image" panel in **Fig. 2g**). We observed that SpaSNE presented these two cell populations as two distinct clusters indicated by the pathological annotation, while both t-SNE and UMAP displayed them close to each other, though they are different cell types and spatially separated from each other (**Fig. 2g**). The better performance of SpaSNE in cell cluster separation is attributed to the higher clustering quality (s = 0.02 in SpaSNE, −0.14 in t-SNE, and −0.15 in UMAP). This example showed that the SpaSNE map better distinguishes cell clusters than t-SNE and UMAP, especially for the cell types that cannot be distinguished by gene expression information alone.

In summary, the above three examples show that SpaSNE gives an integrated low dimensional visualization for spatially resolved profiling data and preserves information of both image and

gene expression. SpaSNE visualization better reveals gene expression variabilities of cell clusters that are not visible from image. It also outperforms t-SNE and UMAP in preserving spatial organization of cells and better distinguishing different cell clusters.

Application to the diseased prostate tissue data

To demonstrate the general applicability of SpaSNE on different diseased tissue types, we shifted from the breast cancer tissues of female patients to the prostate cancer tissues of male patients, which were also obtained from the 10X Genomics data portal. This dataset consists of 4371 spots with three highly mixed cell types: immune, stroma and tumor cells. The cell type annotations were defined by the MaskCNN[38] algorithm that was developed for classifying cell nuclei and cell types in the images (**Supplementary Fig. 1b**). We performed SpaSNE, t-SNE, and UMAP embeddings on this dataset and colored the cells according to cell types and spatial locations (**Fig. 3a-c**). SpaSNE presented separated and compact clusters for most of the colored cells, while t-SNE and UMAP could not well distinguish many of the cell clusters, for example, stroma 1 (orange) and stroma 2 (magenta) (**Fig. 3b-c**). SpaSNE also outperformed t-SNE and UMAP in the three quantitative measures (**Fig. 3d**), which is consistent with the results in human breast cancer data (**Fig. 2d**). We then evaluated the three qualitative performances accordingly (**Fig. 3e-g**) following the steps in **Fig. 2e-g**. First, we highlighted two types of immune cells: immune 1(red) and immune 2 (blue). The cells in immune 1 have a larger overall gene expression variability than cells in immune 2 (**Supplementary Fig. 2b**), which is consistent with larger cluster size of immune 1 than that of immune 2 in SpaSNE map. t-SNE and UMAP also displayed similar properties but the differences in sizes between the two clusters were not as big as in SpaSNE (**Fig. 3e**). Second, we highlighted stroma 1 (orange) and tumor 4 (purple) that were spatially close to each other. The relative spatial distances between these two cell populations were better preserved in SpaSNE than in t-SNE and UMAP (**Fig. 3f**). Third, we highlighted immune 1 (blue) and tumor 3

(green) that were spatially far from each other. These two cell populations were presented as two tight and separable clusters in SpaSNE. t-SNE and UMAP displayed the similar properties but were slightly less separatable (**Fig. 3g**). The above three qualitative evaluations were consistent with the results in human breast cancer data (**Fig. 3e-g**).

In summary, despite the differences in the disease types and data sources, SpaSNE could outperform t-SNE and UMAP in revealing gene expression variabilities of cell clusters, preserving spatial organization of cells and distinguishing different cell clusters. These results demonstrated SpaSNE's potential in serving as a reliable tool for visualizing molecular and spatial information in diverse spatially resolved profiling datasets.

Application to normal tissues based on image-based spatially resolved profiling platforms

We have demonstrated the advantages of SpaSNE on NGS-based experimental platforms. Next we will apply SpaSNE to image-based spatially resolved profiling platforms. Different from the diseased tissues, the cells in normal tissues are more homogenous in gene expression and are usually labeled by tissue types (e.g. developmental layers), and the spatial information mainly represents the global organization of the tissues (e.g. developmental trajectory). Therefore, instead of examining the visualization of gene expression variabilities and spatial closeness in diseased tissues (**Figs. 2-3**), we focused on the following two comparisons among SpaSNE, t-SNE, and UMAP by analyzing the spatially resolved data from normal tissues: 1) distinguishing different tissue types, and 2) revealing the global organization of the tissues.

We first analyzed a mouse visual cortex STARmap dataset[2] that was obtained from normal eyes. In this dataset, 1020 genes were measured in 1207 cells from seven layers: Hippocampus

(HPC), corpus callosum (CC), layer 1 (L1), layer 2/3 (L2/3), layer 4 (L4), layer 5 (L5) and layer 6 (L6) (**Supplementary Fig. 1c**). We performed SpaSNE, t-SNE, and UMAP's embeddings for this dataset and observed that SpaSNE better distinguishes these seven layers than t-SNE and UMAP (**Fig. 4a-c**). This unique feature of SpaSNE can be useful for developmental biologists who are interested in studying the tissue and organ level morphogenesis, where the cells organize themselves into distinct layers, but the gene expression differences might be subtle. SpaSNE also outperformed t-SNE and UAMP in the three quantitative measures (**Fig. 4d**). To study whether SpaSNE can reveal the developmental trajectory in normal tissues, we mimicked a classic analysis approach in the original UMAP publication[18]. In their analysis, the authors utilized several known marker genes to represent different cell types and studied the impacts of dimensionality reduction (e.g. UMAP and t-SNE) on visualization of the differentiation trajectory based on the expression trend of these marker genes. Similarly, we leveraged four known marker genes that were differentially expressed in different developmental layers: LAMP5 (L2/3), NRSN (L4), CPLX1 (L5/HPC), and MOBP (CC). The expressions of the four marker genes peaked at different areas (dashed boxes) and formed a clear developmental trajectory that moves sequentially from the top right to the bottom left in the SpaSNE map, as shown by the arrows in **Fig. 4e**. In t-SNE and UMAP, the expression of the four markers genes did not show a smooth trend and the developmental trajectory is not as clear as in SpaSNE (**Fig. 4f-g**).

Besides the STARmap experimental platform, we analyzed another type of image-based spatially resolved expression profiling platform: MERFISH. This MERFISH dataset[25] contains 5665 cells and 161 genes from the mouse brain hypothalamus. Since the whole hypothalamus image is symmetric, we took 2693 cells from the left half of the image for analysis. The cells were colored according to the nucleus types (**Fig. 5**, **Supplementary Fig. 1d**). We performed SpaSNE, t-SNE, and UMAP's embeddings for this dataset (**Fig. 5a-c**). We observed that SpaSNE better distinguishes different nucleus types compared with t-SNE and UMAP (**Fig. 5a-c**). SpaSNE also

outperformed t-SNE and UAMP in the three quantitative measures (**Fig. 5d**). Following the analysis in **Fig. 4e-g**, we selected four nucleus type specific marker genes: BDNF (MnPO), PRLR (SHy), SOX6 (MPA), and LEPR (VMPO). The expressions of the four marker genes peaked at different areas (dashed boxes) and formed a clear trajectory from the top right to the bottom left in the SpaSNE map, as shown by the arrows in **Fig. 5e**, while the gene expression patterns in the t-SNE or UMAP maps are not as clear as in SpaSNE (**Fig. 5f-g**).

In summary, the above analyses on two normal tissue datasets consistently show that SpaSNE outperforms t-SNE and UMAP in 1) distinguishing different tissue types (e.g. developmental layers), and 2) revealing global organization of the tissues (e.g. developmental trajectory), regardless of organ types, data sources, and experimental platforms.

Discussion

SpaSNE extends the function of t-SNE by preserving not only the local structure of molecular data (e.g. gene expression data), but also maintaining the large-scale structure of molecular data and integrating the spatial information of the cells. With this adaptation, SpaSNE better preserves both molecular data structure and spatial organization of spatially resolved profiling data, which leads to multiple advantages over t-SNE and UMAP. First and most importantly, SpaSNE outperforms t-SNE and UMAP in presenting more accurate and delicate clustering of the cell types with different spatial locations, which is the key step for multiple subsequent statistical and bioinformatics analyses that require correct information of cell types, including but not limited to the differential expression between different cell types, cellular communications among various cell types, and network/pathway-based analysis on each cell type.

Second, SpaSNE can preserve both the spatial organization of cells in the micro-environment and the developmental trajectory in the tissues. Exploring cellular communications in the micro-environment[13, 22-24, 33, 34] and developmental process[14, 29-31, 36] have been the primary goals of many spatially resolved profiling studies[39]. The better spatial structure preservation of SpaSNE over t-SNE and UMAP can support the better preservation of cellular communications in the microenvironment and the spatial organization in developmental tissues. Therefore, SpaSNE could serve as an ideal dimensionality reduction and visualization tool in these research directions, regardless of tissue types and experimental platforms.

Third, SpaSNE offers tunable parameters to adjust the users' requests on the preservation of molecular or spatial information. SpaSNE is capable of integrating two independent sources of data – molecular data (e.g. gene expression data) and spatial position data of cells into a single map. These two aspects represent different biological information and are balanced by the two

weighting parameters α and β. Emphasizing the gene expression information (larger α and smaller β) would enhance the gene expression preservation but diminish the spatial structure preservation (r_g = 0.91, r_s = 0.10, **Supplementary Fig. 3c**; r_g = 0.87, r_s = 0.55, **Supplementary Fig. 4c**), while emphasizing spatial structure (smaller α and larger β) would make the visualization more like the image but not able to reliably reveal gene expression variabilities (r_g = 0.15, r_s = 0.98, **Supplementary Fig. 3d**; r_g = 0.47, r_s = 0.97, **Supplementary Fig. 4d**). SpaSNE allows users to have the flexibility to adjust the balance between gene expression preservation and spatial structure preservation according to their own research purposes, so that users could make the most use of spatially resolved profiling data for data interpretation and hypothesis generation.

Fourth, SpaSNE is a data integration method that is capable of integrating multiple independent features from the same samples (e.g., spatial positions and gene expression that do not share common features but are from the same samples). Traditional data integration methods, such as MultiMAP[40], were usually designed to integrate multiple related features from two or more different samples (e.g., scATAC-seq and scRNA-seq data that share common genes but are from different samples). SpaSNE and MultiMAP serve as two complementary methods. When applied to spatially resolved profiling data (e.g., STARmap[2]), MultiMAP helps to improve the clustering of cells by leveraging transcriptomics information from another scRNAseq data from a different sample. However, we found that MultiMAP cannot preserve the spatial structure of the cells when comparing with ground truth spatial position annotation from the original image[2] (**Supplementary Figure S5a, c**). SpaSNE can make better use of spatially resolved profiling data by preserving both gene expression and spatial organization of the cells (**Supplementary Figure S5a, d**). These two methods could

potentially be integrated to build a more powerful visualization method that can integrate both independent and related features from multi-omics datasets.

We have demonstrated that SpaSNE outperforms t-SNE and UAMP in achieving more accurate clustering for diseased tissues and more meaningful global structure for normal tissues. The advantages in clustering and global structure preservation in low dimensional visualization could have direct impacts on downstream analyses, such as cellular communications among different cell types or cells at different spatial locations, differential gene expression across cell types or along developmental trajectories, etc. Working on these directions is warranted in our follow-up studies.

Despite its extensive utility, SpaSNE has several limitations. First, SpaSNE was designed as a visualization tool with similar purposes as t-SNE or UMAP, but not as a clustering tool for cell type clustering tasks such as BayesSpace[9] and SpaGCN[10]. It serves as a dimensionality reduction tool that can be combined with other clustering methods (e.g. SpaGCN) for better visualization and interpretation of spatially resolved profiling data. Second, we realized that published spatially resolved profiling datasets usually contain a relatively limited number of cells (or spots) in each slide, for which the SpaSNE package is efficient at completing the analysis (**Supplementary Table 1**). However, spatially resolved profiling datasets are rapidly growing and handling large datasets might be needed in the near future. The current SpaSNE package has not been optimized for handling datasets with a large number of cells. A further improvement in this direction may be considered in our follow-up research of the algorithm development. Third, in this study, we have demonstrated that SpaSNE is suitable for both NGS-based (**Figs. 2-3**) and imaging-based spatially resolved experimental platforms (**Figs. 4-5**). Because new spatially resolved profiling technologies are still emerging, a more complete evaluation of these new spatially resolved profiling platforms by SpaSNE would be considered in our follow-up study. Last

but not least, we are working on developing a plug-in to run SpaSNE in the popular single-cell and spatial data analysis's software platforms and toolkits (e.g. Seurat[41]), in order to support wider applications of SpaSNE on a variety of rapidly emerging spatially resolved profiling datasets.

Because spatially resolved epigenomics and proteomics profiling technologies are now under development, here we focused on using available spatially resolved gene expression profiling data to demonstrate that SpaSNE can serve as a powerful dimensionality reduction and visualization tool for analyzing the spatially resolved profiling datasets with both molecular and spatial information, a key data structure that spatially resolved transcriptomes, epigenomes, and proteomes all share. Nowadays, biological and medical research are trending toward a large number of dimensions in tens of thousands of cells or spots with the spatial organization's information. Providing a reliable and robust interpretation on cell types based on both molecular and spatial information by a dimensionality reduction approach can set the foundation for many subsequent analysis steps (e.g., differential gene expression, epigenetic regulation, or protein expression among cell types with spatial organization patterns), and therefore would play an important role in analyzing various spatially resolved profiling data.

Conclusions

This study highlights the versatile utility of SpaSNE in facilitating accurate and resilient interpretation of cell types by leveraging a combination of molecular and spatial information. This framework establishes a solid groundwork for various subsequent analytical procedures, including but not limited to, differential gene expression, trajectory analysis, and pseudotime analysis, thereby enhancing the depth and precision of spatially resolved profiling data exploration.

Availability of data and materials

Code availability

A standalone SpaSNE package is available at https://github.com/Lin-Xu-lab/SpaSNE.git. A guideline of using SpaSNE and the scripts of applying it to the two datasets used for **Fig. 2** and **Fig. 4** in this manuscript were presented in the package. This SpaSNE software was adapted from the bhtsne code (github.com/lvdmaaten/bhtsne).

Data availability

1. Human breast cancer data: https://www.10xgenomics.com/resources/datasets/human-breast-cancer-ductal-carcinoma-in-situ-invasive-carcinoma-ffpe-1-standard-1-3-0

2. Human prostate cancer data:

https://support.10xgenomics.com/spatial-gene-expression/datasets/1.3.0/Visium_FFPE_Human_Prostate_Cancer

3. Mouse visual cortex data:

https://www.dropbox.com/sh/f7ebheru1lbz91s/AADm6D54GSEFXB1feRy6OSASa/visual_1020/20180505_BY3_1kgenes?dl=0&subfolder_nav_tracking=1

4. Mouse hypothalamus data:

https://datadryad.org/stash/dataset/doi:10.5061/dryad.8t8s248

Data annotations: https://github.com/Lin-Xu-lab/SpaSNE.git

Acknowledgements

The resources of the high-performance computing environment from Quantitative Biomedical Research Center (QBRC) and BioHPC at UT Southwestern Medical Center, as well as the Texas Advanced Computing Center (TACC) at The University of Texas at Austin, are gratefully acknowledged. We also thank Ms. Jessie Norris for proofreading this manuscript.

Funding

This work was supported by the following funding: the Rally Foundation, Children's Cancer Fund (Dallas), the Cancer Prevention and Research Institute of Texas (RP180319, RP200103, RP220032, RP170152 and RP180805), and the National Institutes of Health funds (R21CA259771, P30CA142543, HG011996, and R01HL144969) (to L.X.); the National Institutes of Health (1R01GM115473, 1R01GM140012, 5R01CA152301, P30CA142543, P50CA70907, R35GM136375); and the Cancer Prevention and Research Institute of Texas (RP180805, RP190107) (to G. X.).

Contributions

YZ and LX conceived and designed the study. YZ developed the SpaSNE algorithm and performed the data analysis. CT generated the scripts and GitHub page for SpaSNE software. LX and GX acquired the funding. YZ, GX and LX wrote and revised the manuscript. YZ, TC, XX, TW, XZ, GX and LX have read, revised, and approved the final manuscript.

Corresponding authors

Correspondence to Guanghua Xiao or Lin Xu.

Ethics declarations

Ethics approval and consent to participate

Not applicable.

Consent for publication

Not applicable.

Competing interests

The authors declare they have no conflict of interest.

References

1. Rao, A., et al., *Exploring tissue architecture using spatial transcriptomics.* Nature, 2021. **596**(7871): p. 211-220.
2. Wang, X., et al., *Three-dimensional intact-tissue sequencing of single-cell transcriptional states.* Science, 2018. **361**(6400).
3. Shah, S., et al., *Dynamics and Spatial Genomics of the Nascent Transcriptome by Intron seqFISH.* Cell, 2018. **174**(2): p. 363-376 e16.
4. Rodriques, S.G., et al., *Slide-seq: A scalable technology for measuring genome-wide expression at high spatial resolution.* Science, 2019. **363**(6434): p. 1463-1467.
5. Merritt, C.R., et al., *Multiplex digital spatial profiling of proteins and RNA in fixed tissue.* Nat Biotechnol, 2020. **38**(5): p. 586-599.
6. Longo, S.K., et al., *Integrating single-cell and spatial transcriptomics to elucidate intercellular tissue dynamics.* Nat Rev Genet, 2021. **22**(10): p. 627-644.
7. Zeng, Z., et al., *Statistical and machine learning methods for spatially resolved transcriptomics data analysis.* Genome Biol, 2022. **23**(1): p. 83.
8. Shang, L. and X. Zhou, *Spatially aware dimension reduction for spatial transcriptomics.* Nat Commun, 2022. **13**(1): p. 7203.
9. Zhao, E., et al., *Spatial transcriptomics at subspot resolution with BayesSpace.* Nat Biotechnol, 2021. **39**(11): p. 1375-1384.
10. Hu, J., et al., *SpaGCN: Integrating gene expression, spatial location and histology to identify spatial domains and spatially variable genes by graph convolutional network.* Nat Methods, 2021. **18**(11): p. 1342-1351.
11. Grün, D., *Revealing dynamics of gene expression variability in cell state space.* Nature Methods, 2020. **17**: p. 45-49.
12. Narayan, A., B. Berger, and H. Cho, *Assessing single-cell transcriptomic variability through density-preserving data visualization.* Nat Biotechnol, 2021. **39**(6): p. 765-774.
13. Andersson, A., et al., *Spatial deconvolution of HER2-positive breast cancer delineates tumor-associated cell type interactions.* Nat Commun, 2021. **12**(1): p. 6012.
14. Ratz, M., et al., *Clonal relations in the mouse brain revealed by single-cell and spatial transcriptomics.* Nat Neurosci, 2022. **25**(3): p. 285-294.
15. Kobak, D. and P. Berens, *The art of using t-SNE for single-cell transcriptomics.* Nat Commun, 2019. **10**(1): p. 5416.
16. Maaten, L. and G. Hinton, *Visualizing Data using t-SNE.* Journal of Machine Learning Research, 2008. **9**: p. 2579-2605.
17. Maaten, L., *Accelerating t-SNE using Tree-Based Algorithms.* Journal of Machine Learning Research, 2014. **15**: p. 3221-3245.
18. Becht, E., et al., *Dimensionality reduction for visualizing single-cell data using UMAP.* Nat Biotechnol, 2019. **37**(37): p. 38-44.
19. Linderman, G.C., et al., *Fast interpolation-based t-SNE for improved visualization of single-cell RNA-seq data.* Nat Methods, 2019. **16**(3): p. 243-245.
20. Kobak, D. and G.C. Linderman, *Initialization is critical for preserving global data structure in both t-SNE and UMAP.* Nat Biotechnol, 2021. **39**(2): p. 156-157.
21. Do, V.H. and S. Canzar, *A generalization of t-SNE and UMAP to single-cell multimodal omics.* Genome Biol, 2021. **22**(1): p. 130.

22. Moncada, R., et al., *Integrating microarray-based spatial transcriptomics and single-cell RNA-seq reveals tissue architecture in pancreatic ductal adenocarcinomas.* Nat Biotechnol, 2020. **38**(3): p. 333-342.
23. Chen, W.T., et al., *Spatial Transcriptomics and In Situ Sequencing to Study Alzheimer's Disease.* Cell, 2020. **182**(4): p. 976-991 e19.
24. Jackson, H.W., et al., *The single-cell pathology landscape of breast cancer.* Nature, 2020. **578**(7796): p. 615-620.
25. Moffitt, J.R., et al., *Molecular, spatial, and functional single-cell profiling of the hypothalamic preoptic region.* Science, 2018. **362**(6416).
26. Xia, C., et al., *Spatial transcriptome profiling by MERFISH reveals subcellular RNA compartmentalization and cell cycle-dependent gene expression.* Proc Natl Acad Sci U S A, 2019. **116**(39): p. 19490-19499.
27. Maynard, K.R., et al., *Transcriptome-scale spatial gene expression in the human dorsolateral prefrontal cortex.* Nat Neurosci, 2021. **24**(3): p. 425-436.
28. Baccin, C., et al., *Combined single-cell and spatial transcriptomics reveal the molecular, cellular and spatial bone marrow niche organization.* Nat Cell Biol, 2020. **22**(1): p. 38-48.
29. Asp, M., et al., *A Spatiotemporal Organ-Wide Gene Expression and Cell Atlas of the Developing Human Heart.* Cell, 2019. **179**(7): p. 1647-1660 e19.
30. Lohoff, T., et al., *Integration of spatial and single-cell transcriptomic data elucidates mouse organogenesis.* Nat Biotechnol, 2022. **40**(1): p. 74-85.
31. Chow, K.K., et al., *Imaging cell lineage with a synthetic digital recording system.* Science, 2021. **372**(6538).
32. Deng, Y., et al., *Spatial-CUT&Tag: Spatially resolved chromatin modification profiling at the cellular level.* Science, 2022. **375**(6581): p. 681-686.
33. Berglund, E., et al., *Spatial maps of prostate cancer transcriptomes reveal an unexplored landscape of heterogeneity.* Nat Commun, 2018. **9**(1): p. 2419.
34. Ji, A.L., et al., *Multimodal Analysis of Composition and Spatial Architecture in Human Squamous Cell Carcinoma.* Cell, 2020. **182**(2): p. 497-514 e22.
35. Hunter, M.V., et al., *Spatially resolved transcriptomics reveals the architecture of the tumor-microenvironment interface.* Nat Commun, 2021. **12**(1): p. 6278.
36. van den Brink, S.C., et al., *Single-cell and spatial transcriptomics reveal somitogenesis in gastruloids.* Nature, 2020. **582**(7812): p. 405-409.
37. Rousseeuw, R., *Silhouettes: A graphical aid to the interpretation and validation of cluster analysis.* Journal of Computational and Applied Mathematics, 1987. **20**: p. 53-65.
38. Wang, S., et al., *Computational Staining of Pathology Images to Study the Tumor Microenvironment in Lung Cancer.* Cancer Res, 2020. **80**(10): p. 2056-2066.
39. Palla, G., et al., *Spatial components of molecular tissue biology.* Nat Biotechnol, 2022. **40**(3): p. 308-318.
40. Jain, M.S., et al., *MultiMAP: dimensionality reduction and integration of multimodal data.* Genome Biol, 2021. **22**(1): p. 346.
41. Butler, A., et al., *Integrating single-cell transcriptomic data across different conditions, technologies, and species.* Nat Biotechnol, 2018. **36**(5): p. 411-420.

Figure Legends

Figure 1. Workflow of t-SNE and SpaSNE methods. (**a**) Workflow of single cell transcriptomic data analysis. (**b**) Workflow of spatially resolved transcriptomic data analysis. SpaSNE adapts t-SNE by introducing two parameters α and β to better preserve large-scale gene expression distances and spatial structure. The dataset used for visualization is mouse visual cortex STRAmap data[2].

Figure 2. Visualizations of human breast cancer data. (**a-c)** 2-D visualization of cells colored according to ground truth labels from pathologist annotations in (**a**) SpaSNE, (**b**) t-SNE and (**c**) UMAP embeddings. Tumor, immune and other regions that have different spatial locations are labelled by different numbers. (**d**) Using three quantitative measures to evaluate SpaSNE, t-SNE, and UMAP embeddings: Pearson correlation coefficient between embedding distances and gene expression distances (r_g), Pearson correlation coefficient between embedding distances and spatial distances (r_s) and Silhouette score of embedding (s). The error bars show 95% confidence interval of 100 embedding repeats. The stars above the bar plots represent p values of two-sided t-test between results of SpaSNE and t-SNE/UMAP: p<0.001(***). (**e-g**) Visualization of cells in raw image, SpaSNE, t-SNE, and UMAP embeddings by highlighting different pairs of cell states: (**e**) necrosis 1 (blue) and necrosis 2 (orange), (**f**) immune 2 (green) and tumor 2 (purple), (**g**) immune 1 (red) and necrosis 1 (blue).

Figure 3. Visualizations of human prostate cancer data. (**a-c)** 2-D visualization of cells colored according to ground truth labels in (**a**) SpaSNE, (**b**) t-SNE and (**c**) UMAP embeddings. Tumor, immune and other regions that have different spatial locations are labelled by different numbers. (**d**) Using three quantitative measures to evaluate SpaSNE, t-SNE, and UMAP embeddings as in Figure 1**d**. (**e-g**) Visualization of cells in raw image, SpaSNE, t-SNE, and

UMAP embeddings by highlighting different pairs of cell states: (**e**) immune 1 (red) and immune 2 (blue), (**f**) stroma 1 (orange) and tumor 4 (purple), (**g**) immune 1 (blue) and tumor 3 (green).

Figure 4. Visualizations of mouse visual cortex STARmap data. (**a-c)** 2-D visualization of cells colored according to ground truth labels in (**a**) SpaSNE, (**b**) t-SNE and (**c**) UMAP embeddings. (**d**) Using three quantitative measures to evaluate SpaSNE, t-SNE and UMAP embeddings as in Figure **1d.** (**e-f**) Gene expression patterns of four layer-marker genes LAMP5, NRSN1, CPLX1 and MOBP in (**e**) SpaSNE, (**f**) t-SNE and (**g**) UMAP embeddings. The dashed boxes in (**e**) highlight the areas with high gene expression, the arrows represent the developmental trajectory. The magenta represents high expression and cyan low expression.

Figure 5. Visualizations of mouse hypothalamus MERFISH data. (**a-c)** 2-D visualization of cells colored according to ground truth nucleus type labels in (**a**) SpaSNE (**b**) t-SNE and (**c**) UMAP embeddings. (**d**) Using three quantitative measures to evaluate SpaSNE, t-SNE and UMAP embeddings as in Figure 1**d.** (**e-g**) Gene expression patterns of four nucleus-marker genes BDNF, PRLR, SOX6, and LEPR in (**e**) SpaSNE, (**f**) t-SNE and (**g**) UMAP embeddings. The dashed boxes in (**e**) highlight the areas with high gene expression, the arrows represent the nucleus organization. The magenta represents high expression and cyan low expression.

 Click here to access/download;Figure;Figures.pdf

Figure 1. Workflow of t-SNE and SpaSNE methods. (**a**) Workflow of single cell transcriptomic data analysis. (**b**) Workflow of spatially resolved transcriptomic data analysis. SpaSNE adapts t-SNE by introducing two parameters α and β to better preserve large-scale gene expression distances and spatial structure. The dataset used for visualization is mouse visual cortex STRAmap data[2].

Figure 2. Visualizations of human breast cancer data. (**a-c)** 2-D visualization of cells colored according to ground truth labels from pathologist annotations in (**a**) SpaSNE, (**b**) t-SNE and (**c**) UMAP embeddings. Tumor, immune and other regions that have different spatial locations are labelled by different numbers. (**d**) Using three quantitative measures to evaluate SpaSNE, t-SNE, and UMAP embeddings: Pearson correlation coefficient between embedding distances and gene expression distances (r_g), Pearson correlation coefficient between embedding distances and spatial distances (r_s) and Silhouette score of embedding (s). The error bars show 95% confidence interval of 100 embedding repeats. The stars above the bar plots represent p values of two-sided t-test between results of SpaSNE and t-SNE/UMAP: p<0.001(***). (**e-g**) Visualization of cells in raw image, SpaSNE, t-SNE, and UMAP embeddings by highlighting different pairs of cell states: (**e**) necrosis 1 (blue) and necrosis 2 (orange), (**f**) immune 2 (green) and tumor 2 (purple), (**g**) immune 1 (red) and necrosis 1 (blue).

Figure 3. Visualizations of human prostate cancer data. (**a-c)** 2-D visualization of cells colored according to ground truth labels in (**a**) SpaSNE, (**b**) t-SNE and (**c**) UMAP embeddings. Tumor, immune and other regions that have different spatial locations are labelled by different numbers. (**d**) Using three quantitative measures to evaluate SpaSNE, t-SNE, and UMAP embeddings as in Figure 1**d**. (**e-g**) Visualization of cells in raw image, SpaSNE, t-SNE, and UMAP embeddings by highlighting different pairs of cell states: (**e**) immune 1 (red) and immune 2 (blue), (**f**) stroma 1 (orange) and tumor 4 (purple), (**g**) immune 1 (blue) and tumor 3 (green).

Figure 4. Visualizations of mouse visual cortex STARmap data. (**a-c)** 2-D visualization of cells colored according to ground truth labels in (**a**) SpaSNE, (**b**) t-SNE and (**c**) UMAP embeddings. (**d**) Using three quantitative measures to evaluate SpaSNE, t-SNE and UMAP embeddings as in Figure **1d.** (**e-f**) Gene expression patterns of four layer-marker genes LAMP5, NRSN1, CPLX1 and MOBP in (**e**) SpaSNE, (**f**) t-SNE and (**g**) UMAP embeddings. The dashed boxes in (**e**) highlight the areas with high gene expression, the arrows represent the developmental trajectory. The magenta represents high expression and cyan low expression.

Figure 5. Visualizations of mouse hypothalamus MERFISH data. (**a-c)** 2-D visualization of cells colored according to ground truth nucleus type labels in (**a**) SpaSNE (**b**) t-SNE and (**c**) UMAP embeddings. (**d**) Using three quantitative measures to evaluate SpaSNE, t-SNE and UMAP embeddings as in Figure 1**d.** (**e-g**) Gene expression patterns of four nucleus-marker genes BDNF, PRLR, SOX6, and LEPR in (**e**) SpaSNE, (**f**) t-SNE and (**g**) UMAP embeddings. The dashed boxes in (**e**) highlight the areas with high gene expression, the arrows represent the nucleus organization. The magenta represents high expression and cyan low expression.

Figure 1. Workflow of t-SNE and SpaSNE methods.

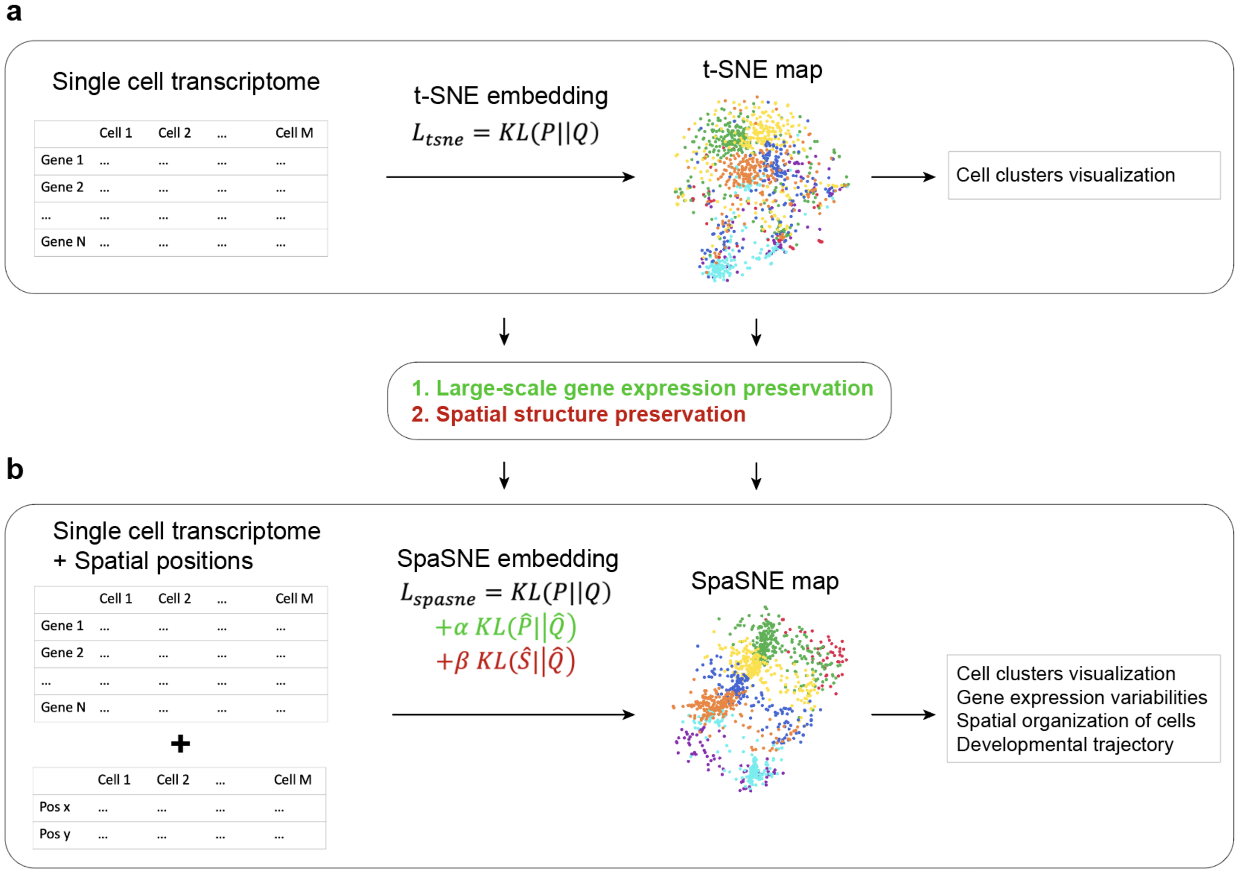

Figure 2. SpaSNE visualization of human breast cancer data

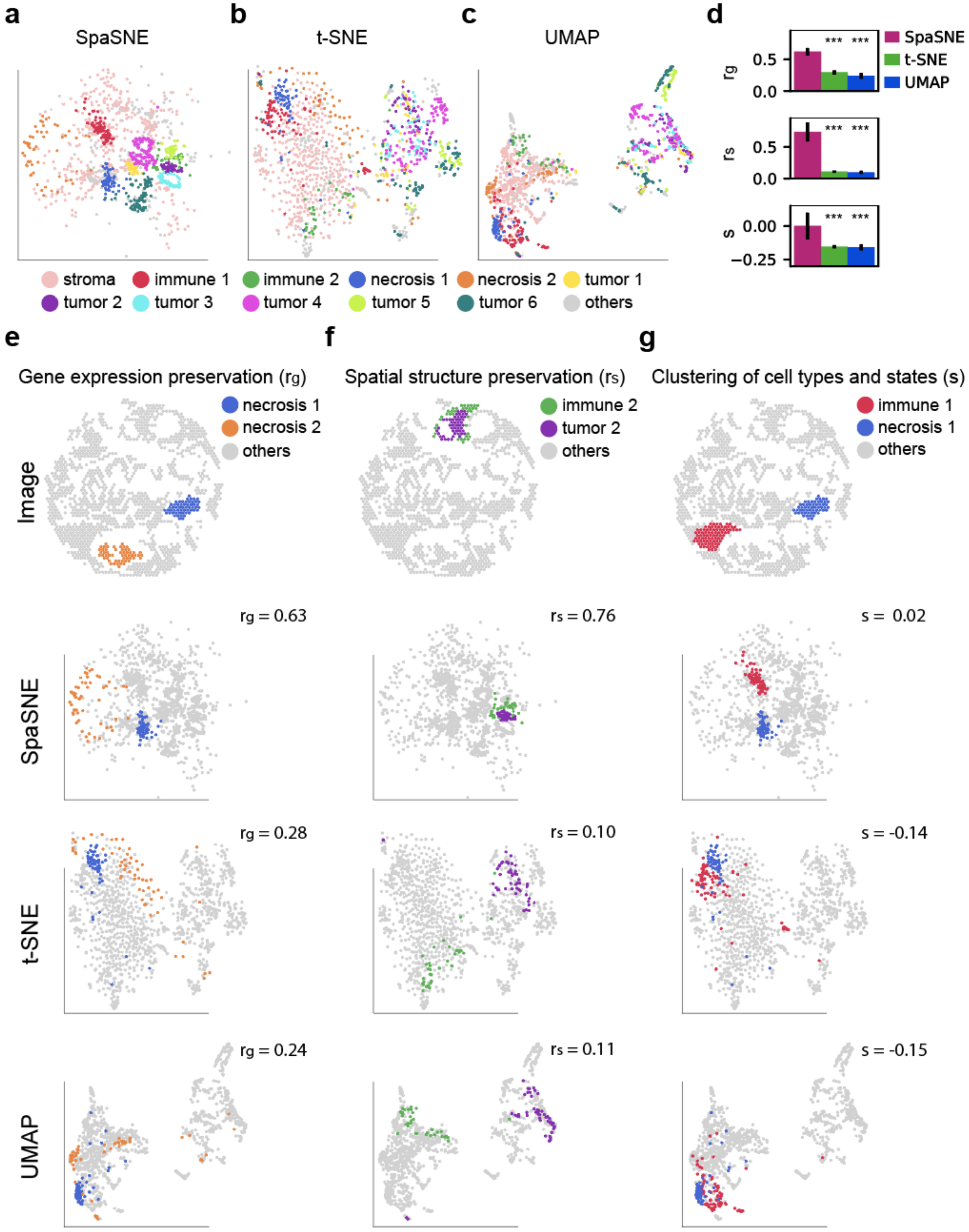

Figure 3. SpaSNE visualization of human prostate cancer data.

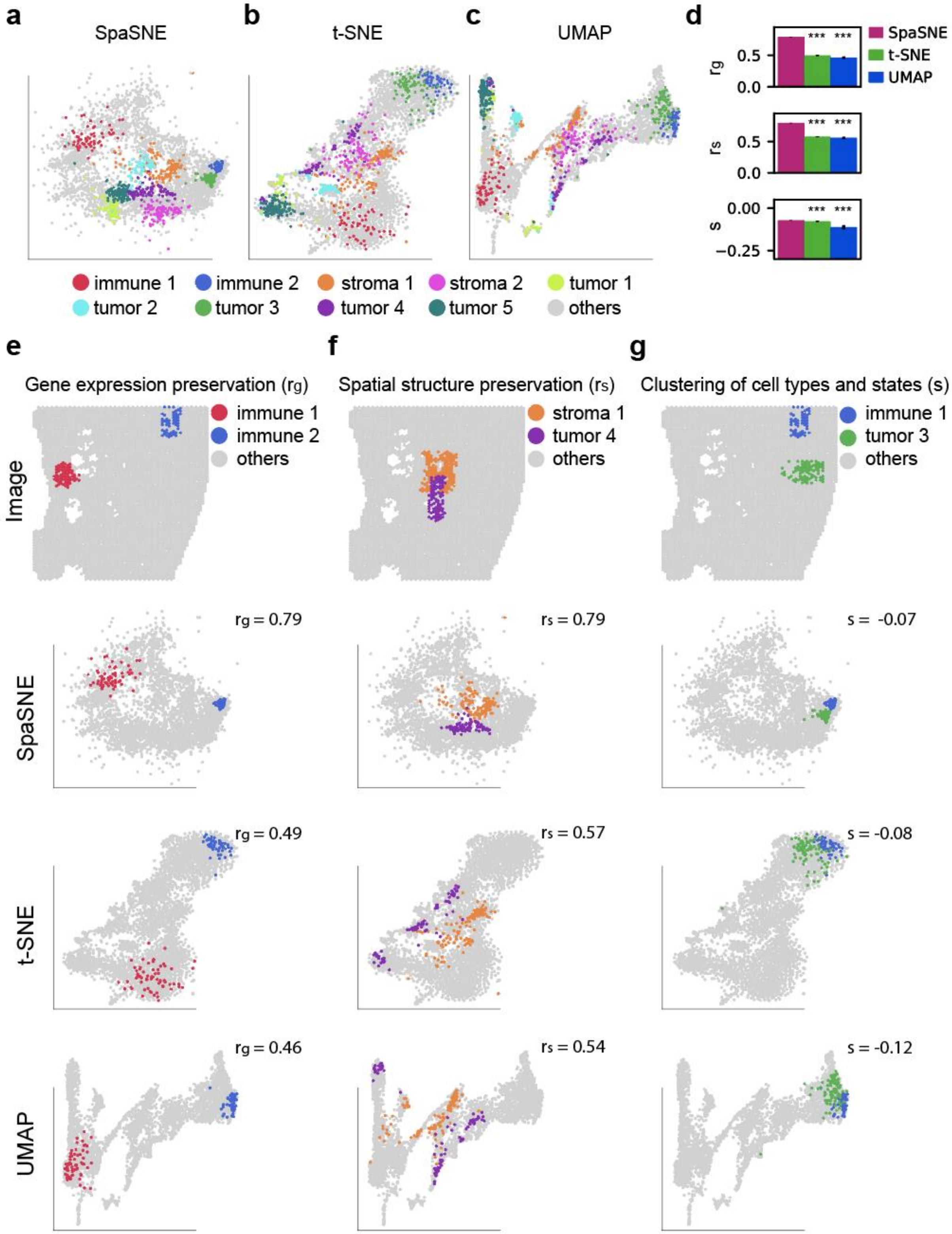

Figure 4. SpaSNE visualization of mouse visual cortex STARmap data

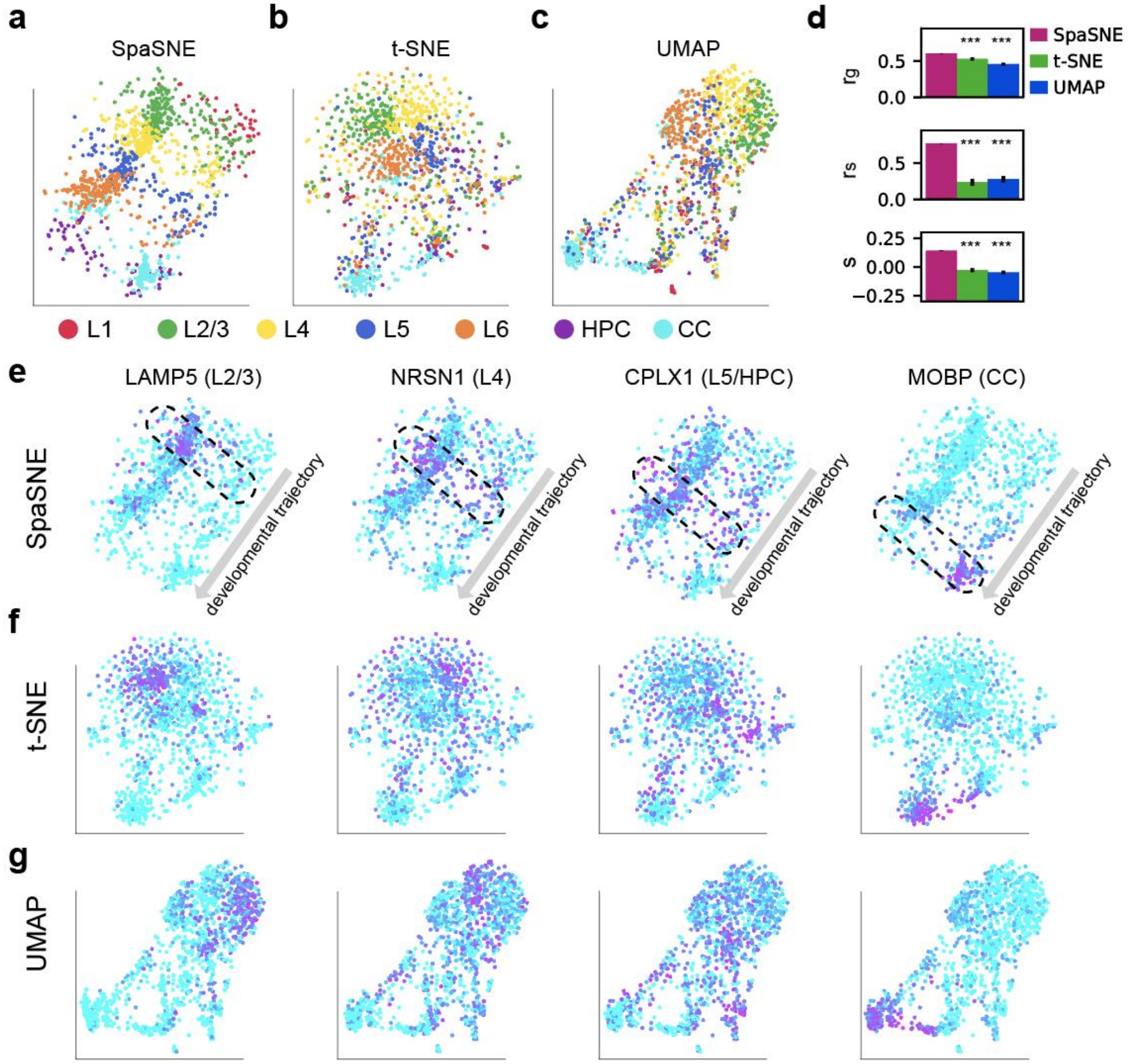

Figure 5. SpaSNE visualization of mouse hypothalamus MERFISH data

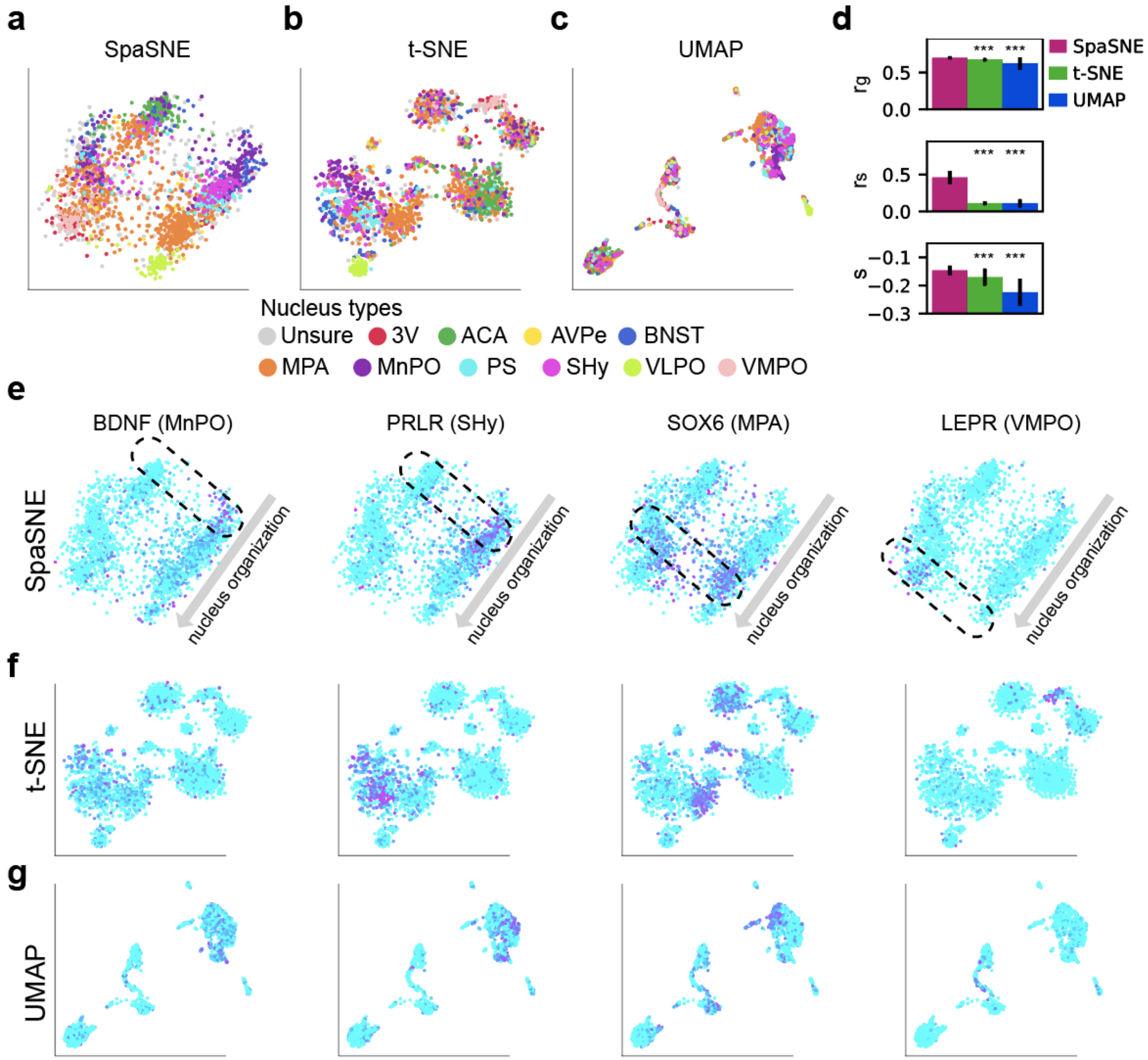

Click here to access/download
Supplementary Material
Supplementary Figures.pdf

Click here to access/download
Supplementary Material
Supplementary Tables.xlsx

Cover letter Click here to access/download;Personal Cover;Cover Letter.pdf

Editorial Board May 3, 2024
GigaScience

Dear Editor:

Please find enclosed a manuscript entitled "Dimensionality reduction for visualizing spatially resolved profiling data using SpaSNE", which we respectfully ask that you consider for publication in *GigaScience*. The manuscript describes original research that has not been published nor presented in a public forum.

Our report addresses a topic of wide interest: How can we leverage spatially resolved profiling technologies on DNA, RNA, and proteins to inform our understanding of biology and medicine? Upon spatially resolved profiling data acquisition, one major hurdle is the subsequent visualization and interpretation of the datasets acquired through dimensionality reduction. Existing state-of-the-art dimensionality reduction methods, such as t-SNE and UMAP, are widely applied in routine single cell sequencing data analysis. However, t-SNE and UMAP do not include spatial information and therefore cannot leverage the complete information of spatially resolved profiling data in low-dimensional visualization. In addition, they do not preserve well the global inter-cluster structure of molecular data (e.g. DNA, RNA, and protein information) when the molecular data is small-sized and highly heterogenous. Direct application of t-SNE and UMAP methods on spatially resolved profiling data might lead to various misleading conclusions, as described in our manuscript in detail.

To address these challenges, we have developed a novel computational algorithm, SpaSNE, with improved performance in preserving both molecular and spatial structure of spatially resolved profiling data. Leveraging four recently published spatially resolved profiling datasets with a variety of disease types, tissue types, and experimental platforms, we keep observing that SpaSNE outperforms t-SNE and UMAP in 1) maintaining accurate and delicate clustering of cell types and states, and 2) preserving spatial organization of cells in the micro-environment and the developmental trajectory of tissues. All these results demonstrate that SpaSNE is a powerful tool for dimensionality reduction and visualization steps in analyzing spatially resolved profiling data.

Our algorithm and the findings have important applications in biology and medicine. First, cell type clustering is the key step that influences various follow-up analysis steps for spatially resolved profiling data (e.g., differential expression, trajectory, and network analysis). Improved accuracy of SpaSNE over t-SNE and UMAP approaches on cell type clustering will significantly increase the reliability and robustness of spatially resolved profiling data analysis, and therefore allow for wider usage of this new technology. Second, cell-cell communication in the micro-environment and developmental trajectory are among the most intensely studied research directions; they have important implications in basic biological mechanisms as well as identification of potential therapeutic targets and predictive biomarkers. Improved performance of SpaSNE over t-SNE and UMAP approaches on preserving spatial information of cell-cell communication and developmental trajectory will also support application of spatially resolved technologies on these topics. Last but not least, SpaSNE is the first computational algorithm that successfully integrates spatial information into dimensionality reduction and visualization, which will inspire researchers to continue to develop similar computational methods for spatially resolved transcriptomics, epigenomics, and proteomics technologies.

For all these reasons, our report will broadly appeal to the *GigaScience* readership.

UT Southwestern Medical Center, 5323 Harry Hines Boulevard, Dallas, Texas 75390-9063 / Tel. 214-648-1403
Guanghua.Xiao@UTSouthwestern.edu, Lin.Xu@utsouthwestern.edu

All co-authors have reviewed the submitted manuscript and have indicated their approval of the findings and conclusions. Please feel free to contact me if you need additional information.

Kind regards,

Lin Xu, Ph.D.
Assistant Professor
Quantitative Biomedical Research Center
Peter O'Donnell Jr. School of Public Health
Department of Pediatrics
Harold C. Simmons Comprehensive Cancer Center
University of Texas Southwestern Medical Center
5323 Harry Hines Blvd. Ste. H9.124
Dallas, TX 75390-8821

Guanghua Xiao, Ph.D.
Professor
Mary Dees McDermott Hicks Chair in Medical Science
Quantitative Biomedical Research Center
Department of Population and Data Sciences
Department of Bioinformatics
University of Texas Southwestern Medical Center
5323 Harry Hines Blvd. Ste. H9.124
Dallas, TX 75390-8821
