# Supplementary material for: Dimensionality reduction for visualizing spatially resolved profiling data using SpaSNE
Source: Gigascience. 2025 Feb 17;14:giaf002. doi: 10.1093/gigascience/giaf002 (PMC11831803; doi:10.1093/gigascience/giaf002)
Supplement: giaf002_GIGA-D-24-00148_R1 [file giaf002_giga-d-24-00148_r1.pdf]

GigaScience

Dimensionality reduction for visualizing spatially resolved profiling data using SpaSNE

--Manuscript Draft--

**Manuscript Number:**	GIGA-D-24-00148R1
**Full Title:**	Dimensionality reduction for visualizing spatially resolved profiling data using SpaSNE
**Article Type:**	Research
**Funding Information:**	
**Abstract:**	Background<br>Spatially resolved profiling technologies to quantify transcriptomes, epigenomes, and proteomes have been emerging as groundbreaking methods for comprehensive molecular characterizations. Dimensionality reduction and visualization is an essential step to analyze and interpret spatially resolved profiling data. However, state-of-the-art dimensionality reduction methods for single-cell sequencing data, such as the t-SNE and UMAP, were not tailored for spatially resolved profiling data.<br><br>Results<br>Here we developed a spatially resolved t-SNE (SpaSNE) method to integrate both spatial and molecular information. We applied it to a variety of public spatially resolved profiling datasets that were generated from three experimental platforms and consisted of cells from different diseases, tissues, and cell types. To compare the performances of SpaSNE, t-SNE, and UMAP, we applied them to four spatially resolved profiling datasets obtained from three distinct experimental platforms (Visium, STARmap, and MERFISH) on both diseased and normal tissues. Comparisons between SpaSNE and these state-of-the-art approaches reveal that SpaSNE achieves more accurate and meaningful visualization that better elucidates the underlying spatial and molecular data structures.<br><br>Conclusions<br>This work demonstrates the broad application of SpaSNE for reliable and robust interpretation of cell types based on both molecular and spatial information, which can set the foundation for many subsequent analysis steps, such as differential gene expression and trajectory or pseudotime analysis on the spatially resolved profiling data.
**Corresponding Author:**	Lin Xu, Ph.D.<br>UT Southwestern: The University of Texas Southwestern Medical Center<br>Dallas, TX UNITED STATES
**Corresponding Author Secondary Information:**	
**Corresponding Author's Institution:**	UT Southwestern: The University of Texas Southwestern Medical Center
**Corresponding Author's Secondary Institution:**	
**First Author:**	Lin Xu, Ph.D.
**First Author Secondary Information:**	
**Order of Authors:**	Lin Xu, Ph.D.
	Yuansheng Zhou, PhD
	Chen Tang, PhD
	Xue Xiao, PhD
	Xiaowei Zhan, PhD
	Tao Wang, PhD
	Guanghua Xiao, PhD
**Order of Authors Secondary Information:**	

Powered by Editorial Manager® and ProduXion Manager® from Aries Systems Corporation

**Response to Reviewers:**	We sincerely thank the editor and the three reviewers for the opportunity to revise our work. In response to the feedback, we have substantially revised the manuscript and added 12 new supplementary figures. Major updates include demonstrating the necessity of preserving spatial information using SpaSNE, adding new biological analyses on the SpaSNE embedding results, and developing an automatic strategy for screening the two key parameters of SpaSNE. The reviewers' insightful comments have significantly enhanced the quality of our paper, and we hope the revised version is now suitable for publication in GigaScience. By uploading a PDF file named "Point-by-point response.pdf", we have addressed each point the editor and reviewers raised, referencing the relevant sections, figures, and tables in the revised paper and supplementary materials. All changes in the manuscript have been highlighted for clarity.
**Additional Information:**	
**Question**	**Response**
Are you submitting this manuscript to a special series or article collection?	No
**Experimental design and statistics**<br><br>Full details of the experimental design and statistical methods used should be given in the Methods section, as detailed in our Minimum Standards Reporting Checklist. Information essential to interpreting the data presented should be made available in the figure legends.<br><br>Have you included all the information requested in your manuscript?	Yes
**Resources**<br><br>A description of all resources used, including antibodies, cell lines, animals and software tools, with enough information to allow them to be uniquely identified, should be included in the Methods section. Authors are strongly encouraged to cite Research Resource Identifiers (RRIDs) for antibodies, model organisms and tools, where possible.<br><br>Have you included the information requested as detailed in our Minimum Standards Reporting Checklist?	Yes
**Availability of data and materials**	Yes

All datasets and code on which the conclusions of the paper rely must be either included in your submission or deposited in publicly available repositories (where available and ethically appropriate), referencing such data using a unique identifier in the references and in the “Availability of Data and Materials” section of your manuscript.<br><br>Have you have met the above requirement as detailed in our Minimum Standards Reporting Checklist?	

Powered by Editorial Manager® and ProduXion Manager® from Aries Systems Corporation

Manuscript Click here to access/download;Manuscript;Manuscript-Jan 6.docx

Dimensionality reduction for visualizing spatially resolved profiling data using SpaSNE

Yuansheng Zhou[1], Chen Tang[1], Xue Xiao[1], Xiaowei Zhan[1,2], Tao Wang[1,2], Guanghua Xiao[1,3 *], Lin Xu[1,4 *]

[1]Quantitative Biomedical Research Center, Peter O'Donnell Jr. School of Public Health, University of Texas Southwestern Medical Center, Dallas, TX, USA

[2]Center for the Genetics of Host Defense, University of Texas Southwestern Medical Center, Dallas, TX, USA

[3]Department of Bioinformatics, University of Texas Southwestern Medical Center, Dallas, TX 75390, USA

[4]Department of Pediatrics, Division of Hematology/Oncology, University of Texas Southwestern Medical Center, Dallas, TX, USA

*Corresponding Authors:

Guanghua Xiao, PhD (Guanghua.Xiao@UTSouthwestern.edu)

Lin Xu, PhD (Lin.Xu@UTSouthwestern.edu)

Lin Xu [0000-0001-5815-4457]; Xiaowei Zhan [0000-0002-6249-7193]; Tao Wang [0000-0002-4355-149X]; Guanghua Xiao [0000-0001-9387-9883].

Abstract

Background

Spatially resolved profiling technologies to quantify transcriptomes, epigenomes, and proteomes have been emerging as groundbreaking methods for comprehensive molecular characterizations. Dimensionality reduction and visualization is an essential step to analyze and interpret spatially resolved profiling data. However, state-of-the-art dimensionality reduction methods for single-cell sequencing data, such as the t-SNE and UMAP, were not tailored for spatially resolved profiling data.

Results

Here we developed a spatially resolved t-SNE (SpaSNE) method to integrate both spatial and molecular information. We applied it to a variety of public spatially resolved profiling datasets that were generated from three experimental platforms and consisted of cells from different diseases, tissues, and cell types. To compare the performances of SpaSNE, t-SNE, and UMAP, we applied them to four spatially resolved profiling datasets obtained from three distinct experimental platforms (Visium, STARmap, and MERFISH) on both diseased and normal tissues. Comparisons between SpaSNE and these state-of-the-art approaches reveal that SpaSNE achieves more accurate and meaningful visualization that better elucidates the underlying spatial and molecular data structures.

Conclusions

This work demonstrates the broad application of SpaSNE for reliable and robust interpretation of cell types based on both molecular and spatial information, which can set the foundation for many subsequent analysis steps, such as differential gene expression and trajectory or pseudotime analysis on the spatially resolved profiling data.

Keywords

Spatially resolved omics, dimensionality reduction, low dimensional visualization, molecular data structure, spatial organization of cells.

Background

Due to the capability to uncover spatial organization and intercellular communication, spatially resolved profiling technologies on DNA, RNA, and proteins have become one of the latest frontiers for cutting-edge research in both basic biology and medicine. While a large number of distinct spatial profiling platforms have been developed so far, a recent review[1] proposed that spatially resolved profiling technologies can be primarily categorized into two major directions: imaging-based approaches (e.g. STARMap[2] and seqFISH[3]) and NGS-based approaches (e.g. Slide-seq[4] and Visium by 10X Genomics). These innovative technologies are promising to transform the way that we think about cell differentiation, tissue development, and disease progression in a spatial fashion, and therefore could lead to novel discoveries on elucidating detailed cellular and molecular mechanisms, as well as identifying effective biomarkers and therapeutic targets[1, 5, 6].

Dimensionality reduction and visualization is an essential step to analyze and interpret the spatially resolved profiling data from DNA, RNA, and proteins[7, 8]. Different from the clustering methods (e.g. BayesSpace[9] or SpaGCN[10]), the aim of developing dimensionality reduction and visualization approaches for spatially resolved profiling data is to visualize the cells in a low dimensional space while maintaining the underlying molecular and spatial data structures (e.g. gene expression variabilities of different cell types[11, 12] and spatial closeness of various cell types[13, 14]). Among the published methods, the t-distributed stochastic neighbor embedding (t-SNE)[15-17] and the uniform manifold approximation and projection (UMAP)[18] have been the most widely used tools for dimensionality reduction and visualization of single-cell sequencing data. Compared with the linear dimensionality reduction methods such as principal component analysis (PCA), both t-SNE and UMAP have great advantages in reliably visualizing cell clusters in single-cell sequencing datasets[15-20]. Some recent variants of t-SNE and UMAP further extended the power of these two algorithms in revealing gene expression variabilities of single

cells[12] or visualizing multimodal omics data[21]. Therefore, recent spatially resolved profiling studies have been using either t-SNE[22-28] or UMAP[2, 13, 14, 29-32] for data visualization. However, different from routine single-cell omics data with molecular information alone, the most unique feature of spatially resolved profiling data is that it contains both molecular information from next-generation sequencing and spatial organization information from images. The current design of t-SNE or UMAP does not leverage both molecular and spatial information simultaneously for analyzing spatially resolved profiling data. Therefore, new dimensionality reduction and visualization algorithms that are able to integrate both molecular and spatial information are in urgent need, because they can visualize cell clusters in the context of tissues' spatial organization and are promising to help uncover more biological insights in the studies of cellular communications[13, 22-24, 33-35] or developmental trajectories[14, 29-31, 36] in the spatial fashion.

Here we developed a spatially resolved t-SNE (SpaSNE) method by adapting t-SNE to more adequately leverage both molecular and spatial information in the spatially resolved profiling data. SpaSNE could provide a comprehensive low-dimensional visualization that better preserves the molecular data structure and spatial organization of cells simultaneously. Because spatially resolved gene expression profiling technologies are well developed so far, in this study we will mainly use spatially resolved gene expression datasets to demonstrate the utility of SpaSNE. To compare the performances of SpaSNE, t-SNE, and UMAP, we applied them to four spatially resolved profiling datasets obtained from three distinct experimental platforms (Visium, STARmap, and MERFISH) on both diseased and normal tissues. The analytical results showed that SpaSNE achieves the most accurate embedding and most meaningful visualization of the spatially resolved profiling data.

Methods

Preprocessing of data

The four spatial gene expression datasets used in the manuscript were presented in **Supplementary Table 1** (the details are also included in the “Data availability” section). For the human breast cancer dataset, there are a total of 2,518 spots in the image, but only 1,272 of them were annotated by a pathologist and the rest spots cannot be determined. We selected these 1,272 spots to evaluate the performance of our algorithm based on the ground truth annotation. For the mouse hypothalamus dataset, we took the left side of the whole slide, which contains 2,693 cells. The annotations of the four datasets were provided in **Supplementary Tables S2-5**. Given a unique molecular identifier (UMI) count matrix, we first used the “scanpy” Python package to normalize the counts. Each cell or spot has a total count equal to the median of total counts per cell. We then transformed them to a natural log scale. For the human breast cancer, human prostate cancer, and mouse visual cortex datasets, we reduced the dimensionality to 200 principal components prior to performing the embedding. For the mouse hypothalamus dataset, we used all 161 genes without dimensionality reduction.

Data annotations

The annotations of the human breast cancer, mouse visual cortex, and mouse hypothalamus datasets were obtained from original papers or websites (the details are included in the “Data availability” section). The human prostate cancer dataset was annotated by the HD-Staining [37] algorithm developed for classifying cell nuclei and cell types in the pathology images. The annotations of the four datasets were provided in the metadata files in **Supplementary Tables S2-S5**. The metadata includes the spatial positions and different kinds of cell type labels for each cell/spot.

Differential expression analysis

The differential expression analysis results in **Supplementary Figures S2-S4, and S7-S12** were performed using Scanpy Toolkit "Wilcoxon" method was used for human breast cancer and prostate cancer data to generate the results in **Supplementary Figures S2-S4, S7-S8.** The "t-test" method was used for mouse visual cortex and hypothalamus to generate the results in **Supplementary Figures S9-12.**

SpaSNE's embedding

The t-distributed stochastic neighbor embedding (t-SNE) algorithm[16] has been widely used in nonlinear dimensionality reduction and visualization for gene expression data. Given a dataset with N spots or cells with gene expression vectors $(x_1, x_2, \ldots, x_N)$, t-SNE defines the pairwise similarities of data points p_{ij} by the following form:

$$p_{j|i} = \frac{\exp(-\|x_i - x_j\|^2/2\sigma_i^2)}{\sum_{i \neq j} \exp(-\|x_i - x_j\|^2/2\sigma_i^2)} \quad (1)$$

$$p_{ij} = \frac{p_{i|j} + p_{j|i}}{2N} \quad (2)$$

Where σ_i is the variance of the Gaussian distribution that centers on x_j. In the low-dimensional ($d = 2\ or\ 3$) representation, the pairwise similarities of points $(y_1, y_2, \ldots, y_N)$ is defined as:

$$q_{ij} = \frac{\left(1 + \|y_i - y_j\|^2\right)^{-1}}{\sum_{i \neq j} \left(1 + \|y_i - y_j\|^2\right)^{-1}} \quad (3)$$

The loss function L_t is defined as the discrepancy between data and embedding points, which is measured by the Kullback–Leibler (KL) divergence of the pairwise similarities:

$$L_t = KL(P||Q) = \sum_i \sum_j p_{ij} \log\left(\frac{p_{ij}}{q_{ij}}\right) \quad (4)$$

The loss function L_t is minimized to achieve the optimal low dimensional representation of the data. The gradient of the loss function L_t with respect to y_i is calculated as:

$$\frac{\partial L_t}{\partial y_i} = 4\sum_j (p_{ij} - q_{ij})(y_i - y_j)\left(1 + \left\|y_i - y_j\right\|^2\right)^{-1} \quad (5)$$

With this definition, t-SNE only preserves local structure of the gene expression because Eq. 1 and 3 are only sensitive to small-scale distance variations.

The spatially resolved profiling data provides the spatial positions of spots or cells $(z_1, z_2, \dots, z_N)$, which cannot be used in t-SNE. SpaSNE improves t-SNE by introducing two new loss functions to preserve both the large-scale gene expression distances and the spatial distances of data. The first loss function measures the KL divergence L_g between the large-scale gene expression distances $\hat{p}_{ij}$ and the large-scale embedding distances $\hat{q}_{ij}$:

$$\hat{p}_{ij} = \frac{1 + \left\|x_i - x_j\right\|^2}{\sum_{i \neq j}\left(1 + \left\|x_i - x_j\right\|^2\right)} \quad (6)$$

$$\hat{q}_{ij} = \frac{1 + \left\|y_i - y_j\right\|^2}{\sum_{i \neq j}(1 + \left\|y_i - y_j\right\|^2)} \quad (7)$$

$$L_g = KL(\hat{P}||\hat{Q}) = \sum_i \sum_j \hat{p}_{ij} \log\left(\frac{\hat{p}_{ij}}{\hat{q}_{ij}}\right) \quad (8)$$

Introducing L_g helps preserve large-scale inter-cluster structure of gene expression because Eq. 6 and 7 are sensitive to large-scale distance variations. The second loss function measures the KL divergence L_s between the large-scale spatial distances $\hat{s}_{ij}$ from image and the large-scale embedding distances $\hat{q}_{ij}$:

$$\hat{s}_{ij} = \frac{1 + \left\|z_i - z_j\right\|^2}{\sum_{i \neq j}\left(1 + \left\|z_i - z_j\right\|^2\right)} \quad (9)$$

$$L_s = KL(\hat{S}||\hat{Q}) = \sum_i \sum_j \hat{s}_{ij} \log\left(\frac{\hat{s}_{ij}}{\hat{q}_{ij}}\right) \quad (10)$$

Integrating the two new loss functions to the original loss function L_t of t-SNE, we get the total loss function with two weighting parameters α and β:

$$L_{total} = L_t + \alpha L_g + \beta L_s \quad (11)$$

The gradient of the loss function L_{total} has a simple form:

$$\frac{\partial L_t}{\partial y_i} = 4 \sum_j \left[(p_{ij} - q_{ij}) - \alpha(\hat{p}_{ij} - \hat{q}_{ij}) - \beta(\hat{s}_{ij} - \hat{q}_{ij})\right](y_i - y_j)\left(1 + \left\|y_i - y_j\right\|^2\right)^{-1} \quad (12)$$

Quantitative evaluation of the embedding quality

Three quantitative measures were defined to evaluate the embedding quality: (1) Pearson correlation coefficient (r_g) between the pairwise Euclidean distances of the gene expressions and the embedding distances of points, this metric is equivalent to the Shephard diagram [38] which was usually used to measure the goodness of fit by low dimensional visualization algorithms[18] [39, 40] [41]; (2) Pearson correlation coefficient (r_s) between pairwise spatial distances and embedding distances of points, which was used to measure the preservation of the spatial structure; (3) Silhouette score (s) which was used to measure the goodness of clustering [41]. This metric was widely used as an evaluation tool for clustering quality analysis [40] [42] [43] [44]. When using the ground truth annotation as the predicted cluster labels, it measures the consistency between the clusters of embedding points and the ground-truth annotation of points. An alternative method of evaluating the embedding quality is the “trustworthiness” metric which measures the preservation of the local structure of data [45]. For spatially resolved data, both the local structure of spatial positions and gene expressions should be considered. The design of SpaSNE leads to increased trustworthiness of spatial structure and decreased trustworthiness of transcriptomic structure compared with t-SNE. However, SpaSNE achieves a higher value of the product of the two scores than t-SNE (**Supplementary Fig. S15**). The metric of the product of the two trustworthiness scores gives similar results as the silhouette score and it might be used as an alternative metric to replace the silhouette score when the ground truth annotation is not available. In our analysis, r_g and r_s were calculated using the “scipy” python package, Silhouette score and trustworthiness were calculated using the “sklearn” python package.

Parameters of visualization algorithms

We applied three algorithms for visualization of the spatially resolved gene expression profiling data: t-SNE, UMAP and SpaSNE. We ran UMAP using the “umap” Python package[18] with default parameters.

For SpaSNE, we screened the combination of parameters α and β on the four datasets and showed how the parameters influence gene expression preservation (r_g), spatial structure preservation (r_s), as well as the stability of the embeddings. The two parameters α and β in Eq. 11 represent the weights of the large-scale gene expression' loss function L_g and the spatial loss function L_s. Therefore, a larger α leads to a larger r_g and a smaller r_s, a larger β leads to a larger r_s and a smaller r_g. In addition to the ratio of α and β, the magnitude of α and β may also influence the stability of the embedding because the contribution of the local cost function L_t in the original t-SNE will be weakened by a large α and β (Eq. 11) and the embedding will become more unstable, especially when the data size is small. We measured stability by the standard deviation (std) of r_g in multiple repeated embeddings of SpaSNE with a given set of parameters (**Supplementary Fig. S16b, d, f, h**). Smaller std is preferred when selecting the parameters.

To determine the optimal combination of parameters for a given data, we developed a heuristic screening approach that consists of two stages: rough screening and fine screening. In rough screening, we screened the two parameters on a larger scale to determine the range where the optimal parameters may fall (**Supplementary Fig. S16 a,c,e,g**). In fine screening, we determined the optimal parameter with a finer resolution (**Supplementary Fig. S16 b,d,f,h**). Here we use the example of the human breast cancer dataset to demonstrate the two-stage screening process in detail:

1. Running 100 repeats of t-SNE with default parameters on human breast cancer datasets and calculating (r_g, r_s) for each repeat. The maximal value of r_g is marked as r_{thres}.
2. Performing rough screening with SpaSNE.
   2.1. Taking α and β from $\{(\alpha, \beta) | \alpha \in [2, 5, 10, 20, 30, 50], \beta \in [1, 5, 10, 15, 25]\}$.
   2.2. In each parameter combination, running 10 repeats of SpaSNE. Setting $r_g = 0$ if $r_g \leq r_{thres}$ in each repeat. Calculating (r_g, r_s) for each repeat. Selecting the optimal embedding that gives maximal value of $r_g \times r_s$ in the 10 repeats and recording the optimal r_g^{opt} and r_s^{opt}.
   2.3. Showing the values of $r_g^{opt} \times r_s^{opt}$ for all the parameter combinations by heatmap (**Supplementary Fig. S16a**).
3. Performing fine screening with SpaSNE.
   3.1. Based on the heatmap results in step 2.3, selecting the range where the optimal parameters may fall: $\{(\alpha, \beta) | \alpha \in [5, 6, 7, .., 20], \beta \in [1, 2, 3, ..., 10]\}$.
   3.2. In each parameter combination, running 20 repeats of SpaSNE. Setting $r_g = 0$ if $r_g \leq r_{thres}$ in each repeat. Calculating (r_g, r_s) for each repeat and standard deviation (std) of r_g of the 20 repeats. Selecting the optimal embedding that gives maximal value of $r_g \times r_s$ in the 20 repeats and recording the optimal values r_g^{opt}, r_s^{opt} and std.
   3.3. Showing the values of $r_g^{opt} \times r_s^{opt}$ (left), std (middle) and $r_g^{opt} \times r_s^{opt} \times \exp(1 - std)$ (right) for all the parameter combinations by heatmap (**Supplementary Fig. S16b**).
4. Determining the optimal parameter combination by selecting the maximal value of $r_g^{opt} \times r_s^{opt} \times \exp(1 - std)$ obtained in 3.3.
5. Running 100 repeats of SpaSNE with the optimal parameter combination obtained in step 4 and selecting the embedding with the maximal values of $r_g \times r_s$.

The results on the four datasets show that the optimal parameters depend on both the size and the type of the data. For example, both the human breast cancer and prostate cancer datasets are generated from the 10X Visium platform, the optimal parameters are larger for the dataset with a larger size (comparing the human prostate cancer dataset with N = 4371, α = 30, to the human breast cancer dataset with N = 1272, α = 9) (**Supplementary Fig. S16b, d**). However, the mouse hypothalamus MERFISH dataset has a larger size than the mouse visual cortex dataset but smaller optimal parameters (comparing the mouse visual cortex dataset with N = 1207, α = 14, to mouse hypothalamus dataset with N = 2693, α = 10) (**Supplementary Fig. S16b, h**). Despite the complex dependence of parameters on the data types, our heuristic two-stage screening approach works for all four diverse datasets and can hopefully be applied to other types of data. The default parameters for SpaSNE were set as $\alpha = 10,\ \beta = 5$ if spatial information is available, and $\alpha = 5,\ \beta = 0$ if the spatial information is not available. The ranges used in tough and fine screenings and the optimal parameters for the four datasets can be found in **Supplementary Table S1**.

All three algorithms were initialized with default setting: UMAP was initialized using a spectral embedding of the fuzzy 1-skeleton, both t-SNE and SpaSNE were initialized from a truncated eigen-vector matrix with the dimension of 50. The stopping criterion for SpaSNE is the same as t-SNE which stops when the maximal iteration (1000 by default) is reached. The perplexity values in SpaSNE and t-SNE were set as the default value which is 50. Increasing perplexity will improve the global structure preservation (r_g) in SpaSNE, but this parameter does not influence the embedding of SpaSNE as much as that of t-SNE (**Supplementary Fig. S17**). The reason is that perplexity determines the number of neighbors in local structure preservation, while SpaSNE embedding largely depends on the two added parameters α and β which preserves the global gene expression structure and spatial structure. The perplexity's influence becomes weaker as α

and β grow larger (comparing human breast cancer dataset with $\alpha = 9$ to human prostate cancer dataset with $\alpha = 30$) (**Supplementary Fig. S17a, b**).

Computational complexity of SpaSNE, t-SNE and UMAP

The computational complexity of t-SNE (implemented by Barnes-Hut-SNE [46]) is $O(NlogN)$. The computational complexity of UMAP is empirically $O(N^{1.14})$ [47]. The computational cost of SpaSNE consists of three parts: the local loss of gene expression $\mathrm{L_t}$, global loss of gene expression $\mathrm{L_g}$ and global loss of spatial positions $\mathrm{L_s}$ (Eq. 11). By applying the vantage-point trees approximation used in Barnes-Hut-SNE, the cost of $\mathrm{L_t}$ can be reduced from $O(N^2)$ to $O(NlogN)$. However, the global loss L_g and L_s cannot be approximated by the local-structure-based strategy in Barnes-Hut-SNE or the Nearest-Neighbor-Descent algorithm [48] used in UMAP. Thus, the computational cost in the current form of SpaSNE is $O(N^2)$. The running time of SpaSNE on a MacBook Pro with a two GHz Quad-Core Intel Core i5 processer and 16 GB 3733 MHz LPDDR4X memory varies from 18 seconds for the human breast cancer dataset with 1272 spots, to 3 minutes for the human prostate cancer dataset with 4371 spots. One possible approach to reducing computational time for large datasets is to mimic the strategy in the SpaceFlow algorithm [49] algorithm to use a fixed number of randomly selected edges to approximate the pairwise distance calculation in global terms $\hat{p}_{ij}$, $\hat{q}_{ij}$ and $\hat{s}_{ij}$ (Eq. 6-9). In this way, the $O(N^2)$ in global loss will be constant and the total cost will become $O(NlogN)$.

Results

Overview of SpaSNE

As one of the most widely used dimensionality reduction tools for single-cell sequencing data analysis, t-SNE has recently been adopted to analyze spatially resolved gene expression profiling data. It takes the gene expression data as input and performs dimensionality reduction and visualization for the data. The primary purpose of t-SNE is to preserve the small-scale local structure of gene expression (i.e. cell clustering) by minimizing the loss function L_{tsne}, which is the Kullback-Leibler (KL) divergence between similarities of data points and embedding points. Therefore, the t-SNE map was mainly used to generate a low dimensional visualization map that reliably displays the clustering of cells (**Fig. 1a**). SpaSNE extends the function of t-SNE by not only preserving the local structure of gene expression, but also maintaining the large-scale inter-cluster structure of gene expression and integrating spatial information of the cells. SpaSNE takes both gene expression data and spatial positions as input. It introduces two new loss functions to preserve large-scale gene expression distances and spatial distances respectively (see Methods). The contributions of these two loss functions are controlled by two independent parameters α and β, which can be adjusted by users to balance gene expression preservation and spatial structure preservation. With this adaptation, SpaSNE can generate a low dimensional visualization map that not only displays the clustering of cells as t-SNE does, but also reveals inter-cluster features in spatially resolved expression profiling data, including gene expression variabilities of different cell clusters, spatial organization of cell types and developmental trajectory of tissues (**Fig. 1b**). We will provide detailed examples to show all these applications of SpaSNE in the following sections. Because spatially resolved epigenomics and proteomics profiling technologies are now under development, here we focused on using available spatially resolved gene expression profiling datasets to demonstrate the utility of SpaSNE.

Application to the diseased breast tissue data based on the Visium spatial transcriptomics technology

We first analyzed spatially resolved transcriptomics data of the human breast cancer tissues from the 10X Genomics data portal (**Supplementary Fig. S1a**). We extracted 1272 annotated spots from the original dataset and performed SpaSNE, t-SNE, and UMAP embeddings to compare their performances on visualization of cells. We showed ten cell clusters, which were colored according to both cell types (based on pathological annotation from 10X Genomics data portal) and spatial locations in SpaSNE, t-SNE, and UMAP embeddings, respectively (**Fig. 2a-c**). SpaSNE presented separated and compact clusters for most of the immune and tumor cells of different spatial locations (**Fig. 2a**). t-SNE and UMAP produced two large clusters that distinguished tumor and non-tumor cells based on gene expression information. However, they could not distinguish the cell clusters with distinct spatial locations. For example, t-SNE and UMAP both presented the six tumor clusters with different spatial locations (tumor 1-6) as one large, disperse cluster and therefore lost the spatial information for them (**Fig. 2b-c**). The separation of cell clusters with different spatial location is important because the cell states are influenced by their neighboring cells. For example, cells in tumor_2 and tumor_5, which are surrounded by immune cells and stroma cells respectively (**Supplementary Fig. S2a**), have distinct expression patterns of marker genes such as IFI27, LGALS3BP and B2M (**Supplementary Fig. S2b-c**). The genes that are highly expressed in tumor_2 (surrounded by immune cells) are involved in biological processes related to immune responses with high enrichment scores in Gene Ontology analysis (**Supplementary Fig. S3a-c, -log10(p) >9**), while the genes that are highly expressed in tumor_5 (surrounded by stroma cells) are involved in translation activities with low enrichment scores (**Supplementary Fig. S3d-f, -log10(p) < 7**). Similarly, cells in immune_2 and immune_1, which are surrounded by tumor and stroma

respectively (**Supplementary Fig. S2a**), have distinct expression patterns of marker genes such as ISG15, IFI6, and IFI27 (**Supplementary Fig. S2b-c**). The highly expressed genes in immune_2 are involved in immune responses while the highly expressed genes in immune_1 are involved in other biological processes (**Supplementary Fig. S4a-f**). These results showed that SpaSNE could produce a more delicate visualization that distinguishes different cell states of the same cell type that interact with different spatial environments by leveraging both gene expression and spatial information. To comprehensively evaluate the performances of SpaSNE, t-SNE, and UMAP in a quantitative manner, we defined three quantitative measures: (1) Pearson correlation coefficient (r_g) between pairwise gene expression distances and embedding distances of points, which was used to measure gene expression preservation; (2) Pearson correlation coefficient (r_s) between pairwise spatial position distances and embedding distances of points, which was used to measure spatial structure preservation; (3) Silhouette score (s) which was used to measure the consistency of clustering with the ground truth annotations. The comparison of the three algorithms showed that SpaSNE outperformed t-SNE and UMAP in all three measures (**Fig. 2d**).

The quantitative advantages of SpaSNE indicate better performances in revealing the underlying data structures. To demonstrate this, we highlighted several representative cell types with different spatial locations from the visualization maps in **Fig. 2a-c** to examine the performances of SpaSNE in detail (**Fig. 2e-g**). First, we highlighted two types of necrosis cells that have different levels of gene expression variabilities: necrosis_1 (blue) and necrosis_2 (orange) ("image" panel in **Fig. 2e**). The cells in necrosis_2 have a higher overall gene expression variability than the cells in necrosis_1 (**Supplementary Fig. S5a**). The difference in gene expression variability of these two cell clusters cannot be reflected in the image, but can be revealed in SpaSNE, t-SNE, and UMAP maps (**Fig. 2e**). In the SpaSNE map, the necrosis_2 cluster has a larger size and smaller point density than that of necrosis_1 (**Fig. 2e**), which is consistent with the smaller gene expression variability of necrosis_2 (**Supplementary Fig. S5a**). t-SNE and UMAP also displayed

similar properties, but the differences of cluster sizes and point densities between the two clusters are not as big as in SpaSNE (**Fig. 2e**). This example showed that the SpaSNE map can better reveal the gene expression variabilities of different cell clusters, which cannot be displayed by image alone.

In addition, we want to highlight that the better performance of SpaSNE in revealing gene expression variabilities can be explained by the higher r_g value that reflects better preservation of gene expression distances (r_g = 0.66 in SpaSNE, 0.33 in t-SNE, and 0.26 in UMAP). When the gene expression preservation was tuned to be extremely high ($\alpha = 9, \beta = 0, r_g$ = 0.90, **Supplementary Fig. S6c**), the difference in gene expression variabilities was even larger, but the spatial structure preservation became worse (r_s = 0.11, **Supplementary Fig. S6c**). SpaSNE allows users to have the flexibility to adjust the preservation of gene expression and spatial structure according to their own research purposes.

Second, we highlighted two different cell types that were spatially close to each other: immune_2 (green) and tumor_2 (purple) ("image" panel in **Fig. 2f**). We observed that SpaSNE was able to preserve the relative spatial distances of these two cell populations by keeping them close to each other, while both t-SNE and UMAP displayed these two cell populations far away from each other without keeping the spatial contacts between them (**Fig. 2f**). The preservation of spatial organization in SpaSNE is due to the preservation of spatial distances (r_s = 0.72 in SpaSNE, 0.11 in t-SNE, and 0.12 in UMAP**)**. When the spatial distances preservation was tuned to be extremely high ($\alpha = 0, \beta = 4, r_s$ = 0.97, **Supplementary Fig. 6d**), the spatial structure more approximated the image, but the gene expression preservation became worse (r_g = 0.16, **Supplementary Fig. S6d**). This example indicated that SpaSNE could outperform t-SNE and UMAP in preserving

spatial organization of cells in the micro-environment (e.g. in human cancers) without harming the capability in distinguishing distinct cell populations.

Third, we highlighted two different cell types in two spatially separated regions: immune_1 (red) and necrosis_1 (blue) ("image" panel in **Fig. 2g**). We observed that SpaSNE presented these two cell populations as two distinct clusters indicated by the pathological annotation, while both t-SNE and UMAP displayed them close to each other, though they are different cell types and spatially separated from each other (**Fig. 2g**). The better performance of SpaSNE in cell cluster separation is attributed to the higher clustering quality (s = 0.003 in SpaSNE, −0.16 in t-SNE, and −0.16 in UMAP). This example showed that the SpaSNE map better distinguishes cell clusters than t-SNE and UMAP, especially for the cell types that cannot be distinguished by gene expression information alone.

In summary, the above three examples show that SpaSNE gives an integrated low dimensional visualization for spatially resolved profiling data and preserves information of both image and gene expression. SpaSNE visualization better reveals gene expression variabilities of cell clusters that are not visible from image. It also outperforms t-SNE and UMAP in preserving the spatial organization of cells and better distinguishing different cell clusters.

Application to the diseased prostate tissue data

To demonstrate the general applicability of SpaSNE on different diseased tissue types, we shifted from the breast cancer tissues of female patients to the prostate cancer tissues of male patients, which were also obtained from the 10X Genomics data portal. This dataset consists of 4371 spots with three highly mixed cell types: immune, stroma, and tumor cells. The cell type annotations were defined by the HD-Staining [37] algorithm that was developed for classifying cell nuclei and

cell types in the images (**Supplementary Fig. S1b**). We performed SpaSNE, t-SNE, and UMAP embeddings on this dataset and colored the cells according to cell types and spatial locations (**Fig. 3a-c**). SpaSNE presented separated and compact clusters for most of the colored cells, while t-SNE and UMAP could not well distinguish many of the cell clusters, for example, tumor 1 (light green) and tumor_2 (cyan) (**Fig. 3b-c**). The more delicate cell clusters separated by SpaSNE represent different cell states with different spatial environments. For example, cells in immune_2 and immune_1 are surrounded by tumor cells and stroma cells respectively, and have distinct expression patterns of marker genes such as CNN1, DES, and TMEFF2 (**Supplementary Fig. S7a-c**). The genes highly expressed in immune_2 cells are involved in biological processes including smooth muscle and smooth muscle cells, which play important roles in prostate cancer [50], with high enrichment scores (-log10(p) > 10) (**Supplementary Fig. S8a-c**). The genes highly expressed in immune_1 cells are involved in vesicle-related biological processes with low enrichment scores (-log10(p) < 4) (**Supplementary Fig. S8d-f**). SpaSNE also outperformed t-SNE and UMAP in the three quantitative measures (**Fig. 3d**), which is consistent with the results in human breast cancer data (**Fig. 2d**). We then evaluated the three qualitative performances accordingly (**Fig. 3e-g**) following the steps in **Fig. 2e-g**. First, we highlighted two types of immune cells: immune_1(red) and immune_2 (blue). The cells in immune_1 have a larger overall gene expression variability than cells in immune_2 (**Supplementary Fig. S5b**), which is consistent with a larger cluster size of immune_1 than that of immune_2 in the SpaSNE map. t-SNE and UMAP also displayed similar properties but the differences in sizes between the two clusters were not as big as in SpaSNE (**Fig. 3e**). Second, we highlighted stroma_1 (orange) and tumor_4 (purple) that were spatially close to each other. The relative spatial distances between these two cell populations were better preserved in SpaSNE than in t-SNE and UMAP (**Fig. 3f**). Third, we highlighted immune_1 (blue) and tumor_3 (green) that were spatially far from each other. These two cell populations were presented as two tight and separable clusters in SpaSNE. t-SNE and

UMAP displayed similar properties but were slightly less separatable (**Fig. 3g**). The above three qualitative evaluations were consistent with the results in human breast cancer data (**Fig. 3e-g**).

In summary, despite the differences in the disease types and data sources, SpaSNE could outperform t-SNE and UMAP in revealing gene expression variabilities of cell clusters, preserving the spatial organization of cells, and distinguishing different cell clusters. These results demonstrated SpaSNE's potential to serve as a reliable tool for visualizing molecular and spatial information in diverse spatially resolved profiling datasets.

Application to normal tissues based on image-based spatially resolved profiling platforms

We have demonstrated the advantages of SpaSNE on NGS-based experimental platforms. Next, we will apply SpaSNE to image-based spatially resolved profiling platforms. Different from the diseased tissues, the cells in normal tissues are more homogenous in gene expression and are usually labeled by tissue types (e.g. developmental layers), and the spatial information mainly represents the global organization of the tissues (e.g. developmental trajectory). Therefore, instead of examining the visualization of gene expression variabilities and spatial closeness in diseased tissues (**Figs. 2-3**), we focused on the following two comparisons among SpaSNE, t-SNE, and UMAP by analyzing the spatially resolved data from normal tissues: 1) distinguishing different tissue types, and 2) revealing the global organization of the tissues.

We first analyzed a mouse visual cortex STARmap dataset[2] that was obtained from normal eyes. In this dataset, 1020 genes were measured in 1207 cells from seven layers: Hippocampus (HPC), corpus callosum (CC), layer 1 (L1), layer 2/3 (L2/3), layer 4 (L4), layer 5 (L5) and layer 6 (L6) (**Supplementary Fig. S1c**). We performed SpaSNE, t-SNE, and UMAP's embeddings for

this dataset and observed that SpaSNE better distinguishes these seven layers than t-SNE and UMAP (**Fig. 4a-c**). This unique feature of SpaSNE can be useful for developmental biologists who are interested in studying the tissue and organ level morphogenesis, where the cells organize themselves into distinct layers, but the gene expression differences might be subtle. SpaSNE also outperformed t-SNE and UAMP in the three quantitative measures (**Fig. 4d**). To study whether SpaSNE can reveal the developmental trajectory in normal tissues, we mimicked a classic analysis approach in the original UMAP publication[18]. In their analysis, the authors utilized several known marker genes to represent different cell types and studied the impacts of dimensionality reduction (e.g. UMAP and t-SNE) on visualization of the differentiation trajectory based on the expression trend of these marker genes. Here, we performed differential expression analysis based on the six layers and found that the differentially expressed genes are involved in biological processes including system development and neurogenesis (**Supplementary Fig. S9a-c**). By comparing each layer with the rest layers, we identified layer-specific markers genes and selected five of them for visualization: FOSB(L1), CAMK2N1(L2/3), CPLX1(L5), PCP1(L6) and MBP(CC) (**Supplementary Fig. S10**). The expressions of the five marker genes peaked at different areas (dashed boxes) and formed a clear developmental trajectory that moves sequentially from the top right to the bottom left in the SpaSNE map, as shown by the arrows in **Fig. 4e**. In t-SNE and UMAP, the expression of the four markers genes did not show a smooth trend and the developmental trajectory is not as clear as in SpaSNE (**Fig. 4f-g**).

Besides the STARmap experimental platform, we analyzed another type of image-based spatially resolved expression profiling platform: MERFISH. This MERFISH dataset[25] contains 5665 cells and 161 genes from the mouse brain hypothalamus. Since the whole hypothalamus image is symmetric, we took 2693 cells from the left half of the image for analysis. The cells were colored according to the nucleus types (**Fig. 5**, **Supplementary Fig. S1d**). We performed SpaSNE, t-SNE, and UMAP's embeddings for this dataset (**Fig. 5a-c**). We observed that SpaSNE better

distinguishes different nucleus types compared with t-SNE and UMAP (**Fig. 5a-c**). SpaSNE also outperformed t-SNE and UAMP in the three quantitative measures (**Fig. 5d**). Following the analysis in **Supplementary Fig. S9-S10**, we performed differential expression analysis based on the eleven nucleus types and found that the differentially expressed genes are involved in biological processes including multicellular organismal process and nervous system development (**Supplementary Fig. S11a-c**). By comparing each layer with the rest layers, we identified layer-specific markers genes and selected five of them for visualization: MBP(ACA), IRS4(BNST), HTR2C (AVPe), SOX6(MPA) and GDA(VLPO) (**Supplementary Fig. S12**). The expressions of the five marker genes peaked at different areas (dashed boxes) and formed a clear trajectory from the top right to the bottom left in the SpaSNE map, as shown by the arrows in **Fig. 5e**, while the gene expression patterns in the t-SNE or UMAP maps are not as clear as in SpaSNE (**Fig. 5f-g**).

In summary, the above analyses on two normal tissue datasets consistently show that SpaSNE outperforms t-SNE and UMAP in 1) distinguishing different tissue types (e.g. developmental layers), and 2) revealing the global organization of the tissues (e.g. developmental trajectory), regardless of organ types, data sources, and experimental platforms.

Discussion

SpaSNE extends the function of t-SNE by preserving not only the local structure of molecular data (e.g. gene expression data), but also maintaining the large-scale structure of molecular data and integrating the spatial information of the cells. With this adaptation, SpaSNE better preserves both molecular data structure and spatial organization of spatially resolved profiling data, which leads to multiple advantages over t-SNE and UMAP. First and most importantly, SpaSNE outperforms t-SNE and UMAP in presenting more accurate and delicate clustering of the cell types with different spatial locations, which is the key step for multiple subsequent statistical and bioinformatics analyses that require correct information of cell types, including but not limited to the differential expression between different cell types, cellular communications among various cell types, and network/pathway-based analysis on each cell type.

Second, SpaSNE can preserve both the spatial organization of cells in the micro-environment and the developmental trajectory in the tissues. Exploring cellular communications in the micro-environment[13, 22-24, 33, 34] and developmental process[14, 29-31, 36] have been the primary goals of many spatially resolved profiling studies[51]. The better spatial structure preservation of SpaSNE over t-SNE and UMAP can support the better preservation of cellular communications in the microenvironment and the spatial organization in developmental tissues. Therefore, SpaSNE could serve as an ideal dimensionality reduction and visualization tool in these research directions, regardless of tissue types and experimental platforms.

Third, SpaSNE offers tunable parameters to adjust the users' requests for the preservation of molecular or spatial information. SpaSNE is capable of integrating two independent sources of data – molecular data (e.g. gene expression data) and spatial position data of cells into a single map. These two aspects represent different biological information and are balanced by the two

weighting parameters α and β. Emphasizing the gene expression information (larger α and smaller β) would enhance the gene expression preservation but diminish the spatial structure preservation (r_g = 0.90, r_s = 0.11, **Supplementary Fig. S6a-c**), while emphasizing spatial structure (smaller α and larger β) would make the visualization more like the image but not able to reliably reveal gene expression variabilities (r_g = 0.16, r_s = 0.97, **Supplementary Fig. S6d**). SpaSNE allows users to have the flexibility to adjust the balance between gene expression preservation and spatial structure preservation according to their own research purposes, so that users can make the most use of spatially resolved profiling data for data interpretation and hypothesis generation.

Fourth, SpaSNE is a data integration method that is capable of integrating multiple independent features from the same samples (e.g., spatial positions and gene expression that do not share common features but are from the same samples). Traditional data integration methods, such as MultiMAP [52], were usually designed to integrate multiple related features from two or more different samples (e.g., scATAC-seq and scRNA-seq data that share common genes but are from different samples). SpaSNE and MultiMAP serve as two complementary methods. When applied to spatially resolved profiling data (e.g., STARmap [2]), MultiMAP helps to improve the clustering of cells by leveraging transcriptomics information from another scRNAseq data from a different sample. However, we found that MultiMAP cannot preserve the spatial structure of the cells when comparing with ground truth spatial position annotation from the original image [2] (**Supplementary Figure S13a, c**). SpaSNE can make better use of spatially resolved profiling data by preserving both gene expression and spatial organization of the cells (**Supplementary Figure S13a, d**). These two methods could potentially be integrated to build a more powerful visualization method that can integrate both independent and related features from multi-omics datasets.

We have demonstrated that SpaSNE outperforms t-SNE and UAMP in achieving more accurate clustering for diseased tissues and more meaningful global structure for normal tissues. The advantages in clustering and global structure preservation in low dimensional visualization could have direct impacts on downstream analyses, such as cellular communications among different cell types or cells at different spatial locations, differential gene expression across cell types or along developmental trajectories, etc. Working on these directions is warranted in our follow-up studies.

Despite its extensive utility, SpaSNE has several limitations. First, SpaSNE was designed as a visualization tool in two-dimensional space with similar purposes as t-SNE or UMAP, but not as a clustering tool for spatially resolved cell-type clustering tasks such as SpatialPCA [8]. Thus, there is no direct comparison between SpaSNE and SpatialPCA. However, a comparison cannot still be made if setting the embedding dimension of SpatialPCA to be 2, and we showed that SpaSNE outperformed SpatialPCA (d = 2) in the quantitative evaluations on all the four datasets (**Supplementary Fig. S14a-h**).

Second, we realized that published spatially resolved profiling datasets usually contain a relatively limited number of cells (or spots) in each slide, for which the SpaSNE package is efficient at completing the analysis (**Supplementary Table S1**). However, spatially resolved profiling datasets are rapidly growing and handling large datasets might be needed in the near future. The current SpaSNE package has not been optimized for handling datasets with a large number of cells. A further improvement in this direction may be considered in our follow-up research of the algorithm development. Third, in this study, we have demonstrated that SpaSNE is suitable for both NGS-based (**Figs. 2-3**) and imaging-based spatially resolved experimental platforms (**Figs. 4-5**). Because new spatially resolved profiling technologies are still emerging, a more complete

evaluation of these new spatially resolved profiling platforms by SpaSNE would be considered in our follow-up study. Fourthly, the design of SpaSNE assumes that the spatial information in spatially resolved transcriptomics data will contribute to the identification of cell states or the global organization of the cells. It may be less effective when there is no correlation between the phenotype and spatial positions of cells [53]. In addition, its performance may be compromised in situations where spatial transcriptomics measurements are taken at multicellular resolution where a single spot contains multiple cell types, or in subcellular resolution where a single cell covers multiple spatial positions such as Visium HD data [54]. We may adapt SpaSNE to analyze such datasets by incorporating current decomposition [55] or aggregation [56] methods for the preprocessing of data in the future. For the four datasets, we found that SpaSNE achieved better embedding quality for the human breast cancer dataset and human prostate cancer dataset generated from the 10x Visium platform, than the mouse visual cortex dataset from the STARmap platform and mouse hypothalamus dataset from the MERFISH platform. The reason might be that 10x Visium platform measures a larger number of genes than STARmap and MERFISH and therefore helps better define cell types. Last but not least, we are working on developing a plug-in to run SpaSNE in the popular single-cell and spatial data analysis software platforms and toolkits (e.g. Seurat[57]), in order to support wider applications of SpaSNE on a variety of rapidly emerging spatially resolved profiling datasets.

We currently focused on using available spatially resolved gene expression profiling data to demonstrate that SpaSNE can serve as a powerful dimensionality reduction and visualization tool for analyzing the spatially resolved profiling datasets with both molecular and spatial information. The design of SpaSNE allows it to analyze not only spatially resolved gene transcriptomic data, but also other types of datasets with similar data structures such as spatially resolved epigenomic [58] and proteomic datasets[59]. Nowadays, biological and medical researches are trending toward a large number of dimensions in tens of thousands of cells or spots with the spatial

organization’s information. Providing a reliable and robust interpretation of cell types based on both molecular and spatial information by a dimensionality reduction approach can set the foundation for many subsequent analysis steps (e.g., differential gene expression, epigenetic regulation, or protein expression among cell types with spatial organization patterns), and therefore would play an important role in analyzing various spatially resolved profiling data.

Conclusions

This study highlights the versatile utility of SpaSNE in facilitating the accurate and resilient interpretation of cell types by leveraging a combination of molecular and spatial information. This framework establishes a solid groundwork for various subsequent analytical procedures, including but not limited to, differential gene expression, trajectory analysis, and pseudotime analysis, thereby enhancing the depth and precision of spatially resolved profiling data exploration.

Availability of Source Code and Requirements

Project name: SpaSNE

Project home page: https://github.com/Lin-Xu-lab/SpaSNE

Operating system(s): Linux or MacOS

Programming language: Python and C++

Other requirements: Python 3.8 and GCC 11.4

License: BSD 3-clause license

RRID: SCR_026223

SpaSNE is also archived in Software Heritage [60]. The guidelines for installing the SpaSNE package, and the tutorials for screening optimal parameters of SpaSNE and performing SpaSNE embeddings with different parameters on human breast cancer data are available on the GitHub page [61]. The SpaSNE software was adapted from the bhtsne scripts [62].

Data availability

The test datasets are available from links [63-66].

Acknowledgements

The resources of the high-performance computing environment from the Quantitative Biomedical Research Center (QBRC) and BioHPC at UT Southwestern Medical Center, as well as the Texas Advanced Computing Center (TACC) at The University of Texas at Austin, are gratefully acknowledged. We also thank Ms. Jessie Norris for proofreading this manuscript.

Funding

This work was supported by the following funding: the Rally Foundation, Children's Cancer Fund (Dallas), the Cancer Prevention and Research Institute of Texas (RP180319, RP200103, RP220032, RP170152 and RP180805), and the National Institutes of Health funds

(R21CA259771, P30CA142543, HG011996, and R01HL144969) (to L.X.); the National Institutes of Health (1R01GM115473, 1R01GM140012, 5R01CA152301, P30CA142543, P50CA70907, R35GM136375); and the Cancer Prevention and Research Institute of Texas (RP180805, RP190107) (to G. X.).

Contributions

YZ and LX conceived and designed the study. YZ developed the SpaSNE algorithm and performed the data analysis. CT generated the scripts and GitHub page for SpaSNE software. LX and GX acquired the funding. YZ, GX, and LX wrote and revised the manuscript. YZ, TC, XX, TW, XZ, GX, and LX have read, revised, and approved the final manuscript.

Corresponding authors

Correspondence to Guanghua Xiao or Lin Xu.

Ethics declarations

Ethics approval and consent to participate

Not applicable.

Consent for publication

Not applicable.

Competing interests

The authors declare they have no conflict of interest.

References

1. Rao, A., et al., Exploring tissue architecture using spatial transcriptomics. Nature, 2021. 596(7871): p. 211-220.
2. Wang, X., et al., Three-dimensional intact-tissue sequencing of single-cell transcriptional states. Science, 2018. 361(6400).
3. Shah, S., et al., Dynamics and Spatial Genomics of the Nascent Transcriptome by Intron seqFISH. Cell, 2018. 174(2): p. 363-376 e16.
4. Rodriques, S.G., et al., Slide-seq: A scalable technology for measuring genome-wide expression at high spatial resolution. Science, 2019. 363(6434): p. 1463-1467.
5. Merritt, C.R., et al., Multiplex digital spatial profiling of proteins and RNA in fixed tissue. Nat Biotechnol, 2020. 38(5): p. 586-599.
6. Longo, S.K., et al., Integrating single-cell and spatial transcriptomics to elucidate intercellular tissue dynamics. Nat Rev Genet, 2021. 22(10): p. 627-644.
7. Zeng, Z., et al., Statistical and machine learning methods for spatially resolved transcriptomics data analysis. Genome Biol, 2022. 23(1): p. 83.
8. Shang, L. and X. Zhou, Spatially aware dimension reduction for spatial transcriptomics. Nat Commun, 2022. 13(1): p. 7203.
9. Zhao, E., et al., Spatial transcriptomics at subspot resolution with BayesSpace. Nat Biotechnol, 2021. 39(11): p. 1375-1384.
10. Hu, J., et al., SpaGCN: Integrating gene expression, spatial location and histology to identify spatial domains and spatially variable genes by graph convolutional network. Nat Methods, 2021. 18(11): p. 1342-1351.
11. Grün, D., Revealing dynamics of gene expression variability in cell state space. Nature Methods, 2020. 17: p. 45-49.
12. Narayan, A., B. Berger, and H. Cho, Assessing single-cell transcriptomic variability through density-preserving data visualization. Nat Biotechnol, 2021. 39(6): p. 765-774.
13. Andersson, A., et al., Spatial deconvolution of HER2-positive breast cancer delineates tumor-associated cell type interactions. Nat Commun, 2021. 12(1): p. 6012.
14. Ratz, M., et al., Clonal relations in the mouse brain revealed by single-cell and spatial transcriptomics. Nat Neurosci, 2022. 25(3): p. 285-294.
15. Kobak, D. and P. Berens, The art of using t-SNE for single-cell transcriptomics. Nat Commun, 2019. 10(1): p. 5416.
16. Maaten, L. and G. Hinton, Visualizing Data using t-SNE. Journal of Machine Learning Research, 2008. 9: p. 2579-2605.
17. Maaten, L., Accelerating t-SNE using Tree-Based Algorithms. Journal of Machine Learning Research, 2014. 15: p. 3221-3245.
18. Becht, E., et al., Dimensionality reduction for visualizing single-cell data using UMAP. Nat Biotechnol, 2019. 37(37): p. 38-44.
19. Linderman, G.C., et al., Fast interpolation-based t-SNE for improved visualization of single-cell RNA-seq data. Nat Methods, 2019. 16(3): p. 243-245.
20. Kobak, D. and G.C. Linderman, Initialization is critical for preserving global data structure in both t-SNE and UMAP. Nat Biotechnol, 2021. 39(2): p. 156-157.
21. Do, V.H. and S. Canzar, A generalization of t-SNE and UMAP to single-cell multimodal omics. Genome Biol, 2021. 22(1): p. 130.

22. Moncada, R., et al., Integrating microarray-based spatial transcriptomics and single-cell RNA-seq reveals tissue architecture in pancreatic ductal adenocarcinomas. Nat Biotechnol, 2020. 38(3): p. 333-342.
23. Chen, W.T., et al., Spatial Transcriptomics and In Situ Sequencing to Study Alzheimer's Disease. Cell, 2020. 182(4): p. 976-991 e19.
24. Jackson, H.W., et al., The single-cell pathology landscape of breast cancer. Nature, 2020. 578(7796): p. 615-620.
25. Moffitt, J.R., et al., Molecular, spatial, and functional single-cell profiling of the hypothalamic preoptic region. Science, 2018. 362(6416).
26. Xia, C., et al., Spatial transcriptome profiling by MERFISH reveals subcellular RNA compartmentalization and cell cycle-dependent gene expression. Proc Natl Acad Sci U S A, 2019. 116(39): p. 19490-19499.
27. Maynard, K.R., et al., Transcriptome-scale spatial gene expression in the human dorsolateral prefrontal cortex. Nat Neurosci, 2021. 24(3): p. 425-436.
28. Baccin, C., et al., Combined single-cell and spatial transcriptomics reveal the molecular, cellular and spatial bone marrow niche organization. Nat Cell Biol, 2020. 22(1): p. 38-48.
29. Asp, M., et al., A Spatiotemporal Organ-Wide Gene Expression and Cell Atlas of the Developing Human Heart. Cell, 2019. 179(7): p. 1647-1660 e19.
30. Lohoff, T., et al., Integration of spatial and single-cell transcriptomic data elucidates mouse organogenesis. Nat Biotechnol, 2022. 40(1): p. 74-85.
31. Chow, K.K., et al., Imaging cell lineage with a synthetic digital recording system. Science, 2021. 372(6538).
32. Deng, Y., et al., Spatial-CUT&Tag: Spatially resolved chromatin modification profiling at the cellular level. Science, 2022. 375(6581): p. 681-686.
33. Berglund, E., et al., Spatial maps of prostate cancer transcriptomes reveal an unexplored landscape of heterogeneity. Nat Commun, 2018. 9(1): p. 2419.
34. Ji, A.L., et al., Multimodal Analysis of Composition and Spatial Architecture in Human Squamous Cell Carcinoma. Cell, 2020. 182(2): p. 497-514 e22.
35. Hunter, M.V., et al., Spatially resolved transcriptomics reveals the architecture of the tumor-microenvironment interface. Nat Commun, 2021. 12(1): p. 6278.
36. van den Brink, S.C., et al., Single-cell and spatial transcriptomics reveal somitogenesis in gastruloids. Nature, 2020. 582(7812): p. 405-409.
37. Wang, S., et al., Computational Staining of Pathology Images to Study the Tumor Microenvironment in Lung Cancer. Cancer Res, 2020. 80(10): p. 2056-2066.
38. Shepard, R.N., Multidimensional scaling, tree-fitting, and clustering. Science, 1980. 210(4468): p. 390-398.
39. Zhou, Y. and T.O. Sharpee, Using Global t-SNE to Preserve Intercluster Data Structure. Neural Comput, 2022. 34(8): p. 1637-1651.
40. Zhou, Y. and T.O. Sharpee, Hyperbolic geometry of gene expression. iScience, 2021. 24(3): p. 102225.
41. Rousseeuw, R., Silhouettes: A graphical aid to the interpretation and validation of cluster analysis. Journal of Computational and Applied Mathematics, 1987. 20: p. 53-65.
42. Chatzimparmpas, A., R.M. Martins, and A. Kerren, t-viSNE: Interactive Assessment and Interpretation of t-SNE Projections. IEEE Trans Vis Comput Graph, 2020. 26(8): p. 2696-2714.

43. Shahapure, K.R. and C. Nicholas. Cluster Quality Analysis Using Silhouette Score. in 2020 IEEE 7th International Conference on Data Science and Advanced Analytics (DSAA). 2020.
44. Shutaywi, M. and N.N. Kachouie, Silhouette Analysis for Performance Evaluation in Machine Learning with Applications to Clustering. Entropy (Basel), 2021. 23(6).
45. Maaten, L.v.d., Learning a Parametric Embedding by Preserving Local Structure, in Proceedings of the Twelfth International Conference on Artificial Intelligence and Statistics, D. David van and W. Max, Editors. 2009, PMLR: Proceedings of Machine Learning Research. p. 384--391.
46. Maaten, L.V.D., Accelerating t-SNE using tree-based algorithms. J. Mach. Learn. Res., 2014. 15(1): p. 3221–3245.
47. McInnes, L., J. Healy, and J. Melville UMAP: Uniform Manifold Approximation and Projection for Dimension Reduction. 2018. arXiv:1802.03426 DOI: 10.48550/arXiv.1802.03426.
48. Dong, W., C. Moses, and K. Li, Efficient k-nearest neighbor graph construction for generic similarity measures, in Proceedings of the 20th international conference on World wide web. 2011, Association for Computing Machinery: Hyderabad, India. p. 577–586.
49. Ren, H., et al., Identifying multicellular spatiotemporal organization of cells with SpaceFlow. Nature communications, 2022. 13(1): p. 4076.
50. Pederzoli, F., et al., Stromal cells in prostate cancer pathobiology: friends or foes? British Journal of Cancer, 2023. 128(6): p. 930-939.
51. Palla, G., et al., Spatial components of molecular tissue biology. Nat Biotechnol, 2022. 40(3): p. 308-318.
52. Jain, M.S., et al., MultiMAP: dimensionality reduction and integration of multimodal data. Genome Biol, 2021. 22(1): p. 346.
53. Russell, A.J.C., et al., Slide-tags enables single-nucleus barcoding for multimodal spatial genomics. Nature, 2024. 625(7993): p. 101-109.
54. Nagendran, M., et al., 1457 Visium HD enables spatially resolved, single-cell scale resolution mapping of FFPE human breast cancer tissue. 2023, BMJ Specialist Journals.
55. Elosua-Bayes, M., et al., SPOTlight: seeded NMF regression to deconvolute spatial transcriptomics spots with single-cell transcriptomes. Nucleic acids research, 2021. 49(9): p. e50-e50.
56. Benjamin, K., et al., Multiscale topology classifies cells in subcellular spatial transcriptomics. Nature, 2024: p. 1-7.
57. Butler, A., et al., Integrating single-cell transcriptomic data across different conditions, technologies, and species. Nat Biotechnol, 2018. 36(5): p. 411-420.
58. Zhang, D., et al., Spatial epigenome–transcriptome co-profiling of mammalian tissues. Nature, 2023. 616(7955): p. 113-122.
59. Liu, Y., et al., High-plex protein and whole transcriptome co-mapping at cellular resolution with spatial CITE-seq. Nature Biotechnology, 2023. 41(10): p. 1405-1409.
60. Zhou Y, Tang C, Xiao X, Zhan X, Wang T, Xiao G, Xu L. Dimensionality reduction for visualizing spatially resolved profiling data using SpaSNE (Version 1). [Computer software]. Software Heritage, 2025. https://archive.softwareheritage.org/browse/revision/adef46ef7b4bc0a223b0cb6ccd39b9f3d02fc487/?origin_url=https://github.com/Lin-Xu-lab/SpaSNE.

61. SpaSNE package. [Computer software]. GitHub. https://github.com/Lin-Xu-lab/SpaSNE.git. Acessed on June 30, 2024.
62. bhtsne scripts. [Computer software]. GitHub. https://github.com/lvdmaaten/bhtsne. Acessed on June 30, 2024.
63. Human breast cancer data: https://www.10xgenomics.com/resources/datasets/human-breast-cancer-ductal-carcinoma-in-situ-invasive-carcinoma-ffpe-1-standard-1-3-0
64. Human prostate cancer data: https://support.10xgenomics.com/spatial-gene-expression/datasets/1.3.0/Visium_FFPE_Human_Prostate_Cancer
65. Mouse visual cortex data: https://www.dropbox.com/sh/f7ebheru1lbz91s/AADm6D54GSEFXB1feRy6OSASa/visual_1020/20180505_BY3_1kgenes?dl=0&subfolder_nav_tracking=1
66. Mouse hypothalamus data: https://datadryad.org/stash/dataset/doi:10.5061/dryad.8t8s248

Figure Legends

Figure 1. Workflow of t-SNE and SpaSNE methods. (**a**) Workflow of single cell transcriptomic data analysis. (**b**) Workflow of spatially resolved transcriptomic data analysis. SpaSNE adapts t-SNE by introducing two parameters α and β to better preserve large-scale gene expression distances and spatial structure. The dataset used for visualization is mouse visual cortex STRAmap data[2].

Figure 2. Visualizations of human breast cancer data. (**a-c)** 2-D visualization of cells colored according to ground truth labels from pathologist annotations in (**a**) SpaSNE, (**b**) t-SNE and (**c**) UMAP embeddings. Tumor, immune and other regions that have different spatial locations are labelled by different numbers. (**d**) Using three quantitative measures to evaluate SpaSNE, t-SNE, and UMAP embeddings: Pearson correlation coefficient between embedding distances and gene expression distances (r_g), Pearson correlation coefficient between embedding distances and spatial distances (r_s) and Silhouette score of embedding (s). The error bars show 95% confidence interval of 100 embedding repeats. The stars above the bar plots represent p values of two-sided t-test between results of SpaSNE and t-SNE/UMAP: p<0.001(***). (**e-g**) Visualization of cells in raw image, SpaSNE, t-SNE, and UMAP embeddings by highlighting different pairs of cell states: (**e**) necrosis_1 (blue) and necrosis_2 (orange), (**f**) immune_2 (green) and tumor_2 (purple), (**g**) immune_1 (red) and necrosis_1 (blue).

Figure 3. Visualizations of human prostate cancer data. (**a-c)** 2-D visualization of cells colored according to ground truth labels in (**a**) SpaSNE, (**b**) t-SNE and (**c**) UMAP embeddings. Tumor, immune and other regions that have different spatial locations are labelled by different numbers. (**d**) Using three quantitative measures to evaluate SpaSNE, t-SNE, and UMAP

embeddings as in Figure 1**d**. (**e-g**) Visualization of cells in raw image, SpaSNE, t-SNE, and UMAP embeddings by highlighting different pairs of cell states: (**e**) immune_1 (red) and immune_2 (blue), (**f**) stroma_1 (orange) and tumor_4 (purple), (**g**) immune_1 (blue) and tumor_3 (green).

Figure 4. Visualizations of mouse visual cortex STARmap data. (**a-c)** 2-D visualization of cells colored according to ground truth labels in (**a**) SpaSNE, (**b**) t-SNE and (**c**) UMAP embeddings. (**d**) Using three quantitative measures to evaluate SpaSNE, t-SNE and UMAP embeddings as in Figure **1d.** (**e-f**) Gene expression patterns of five layer-marker genes FOSB, CAMK2N1, CPLX1, PCP4 and MBP in (**e**) SpaSNE, (**f**) t-SNE and (**g**) UMAP embeddings. The dashed boxes in (**e**) highlight the areas with high gene expression, the arrows represent the developmental trajectory. The magenta represents high expression and cyan low expression.

Figure 5. Visualizations of mouse hypothalamus MERFISH data. (**a-c)** 2-D visualization of cells colored according to ground truth nucleus type labels in (**a**) SpaSNE (**b**) t-SNE and (**c**) UMAP embeddings. (**d**) Using three quantitative measures to evaluate SpaSNE, t-SNE and UMAP embeddings as in Figure 1**d.** (**e-g**) Gene expression patterns of five nucleus-marker genes MBP, IRS4, HTR2C, SOX6 and GDA in (**e**) SpaSNE, (**f**) t-SNE and (**g**) UMAP embeddings. The dashed boxes in (**e**) highlight the areas with high gene expression, the arrows represent the nucleus organization. The magenta represents high expression and cyan low expression.

 Click here to access/download;Figure;Figures.pdf

Figure 1. Workflow of t-SNE and SpaSNE methods. (**a**) Workflow of single-cell transcriptomic data analysis. (**b**) Workflow of spatially resolved transcriptomic data analysis. SpaSNE adapts t-SNE by introducing two parameters α and β to better preserve large-scale gene expression distances and spatial structure. The dataset used for visualization is mouse visual cortex STRAmap data[2].

Figure 2. Visualizations of human breast cancer data. (**a-c)** 2-D visualization of cells colored according to ground truth labels from pathologist annotations in (**a**) SpaSNE, (**b**) t-SNE, and (**c**) UMAP embeddings. Tumor, immune, and other regions that have different spatial locations are labeled by different numbers. (**d**) Using three quantitative measures to evaluate SpaSNE, t-SNE, and UMAP embeddings: Pearson correlation coefficient between embedding distances and gene expression distances (r_g), Pearson correlation coefficient between embedding distances and spatial distances (r_s) and Silhouette score of embedding (s). The error bars show a 95% confidence interval of 100 embedding repeats. The stars above the bar plots represent p values of the two-sided t-test between results of SpaSNE and t-SNE/UMAP: p<0.001(***). (**e-g**) Visualization of cells in the raw image, SpaSNE, t-SNE, and UMAP embeddings by highlighting different pairs of cell states: (**e**) necrosis_1 (blue) and necrosis_2 (orange), (**f**) immune_2 (green) and tumor_2 (purple), (**g**) immune_1 (red) and necrosis_1 (blue).

Figure 3. Visualizations of human prostate cancer data. (**a-c)** 2-D visualization of cells colored according to ground truth labels in (**a**) SpaSNE, (**b**) t-SNE, and (**c**) UMAP embeddings. Tumor, immune, and other regions that have different spatial locations are labeled by different numbers. (**d**) Using three quantitative measures to evaluate SpaSNE, t-SNE, and UMAP embeddings as in Figure 1**d**. (**e-g**) Visualization of cells in the raw image, SpaSNE, t-SNE, and UMAP embeddings by highlighting different pairs of cell states: (**e**) immune_1 (red) and immune_2 (blue), (**f**) stroma_1 (orange) and tumor_4 (purple), (**g**) immune_1 (blue) and tumor_3 (green).

Figure 4. Visualizations of mouse visual cortex STARmap data. (**a-c)** 2-D visualization of cells colored according to ground truth labels in (**a**) SpaSNE, (**b**) t-SNE, and (**c**) UMAP embeddings. (**d**) Using three quantitative measures to evaluate SpaSNE, t-SNE, and UMAP embeddings as in Figure **1d.** (**e-f**) Gene expression patterns of five layer-marker genes FOSB, CAMK2N1, CPLX1, PCP4, and MBP in (**e**) SpaSNE, (**f**) t-SNE, and (**g**) UMAP embeddings. The dashed boxes in (**e**) highlight the areas with high gene expression, and the arrows represent the developmental trajectory. The magenta represents high expression and cyan low expression.

Figure 5. Visualizations of mouse hypothalamus MERFISH data. (**a-c)** 2-D visualization of cells colored according to ground truth nucleus type labels in (**a**) SpaSNE (**b**) t-SNE and (**c**) UMAP embeddings. (**d**) Using three quantitative measures to evaluate SpaSNE, t-SNE, and UMAP embeddings as in Figure 1**d.** (**e-g**) Gene expression patterns of five nucleus-marker genes MBP, IRS4, HTR2C, SOX6, and GDA in (**e**) SpaSNE, (**f**) t-SNE, and (**g**) UMAP embeddings. The dashed boxes in (**e**) highlight the areas with high gene expression, and the arrows represent the nucleus organization. The magenta represents high expression and cyan low expression.

Figure 1. Workflow of t-SNE and SpaSNE methods.

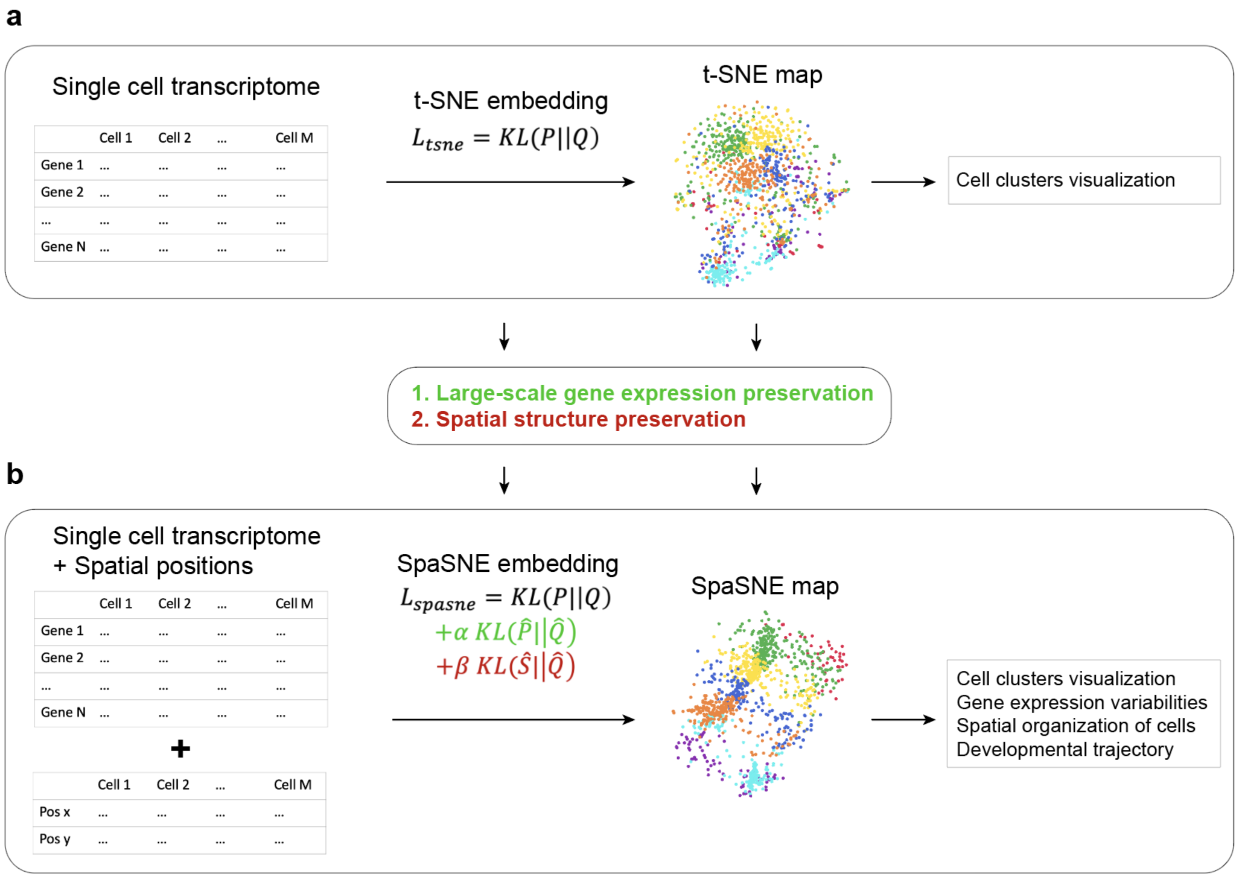

Figure 2. SpaSNE visualization of human breast cancer data

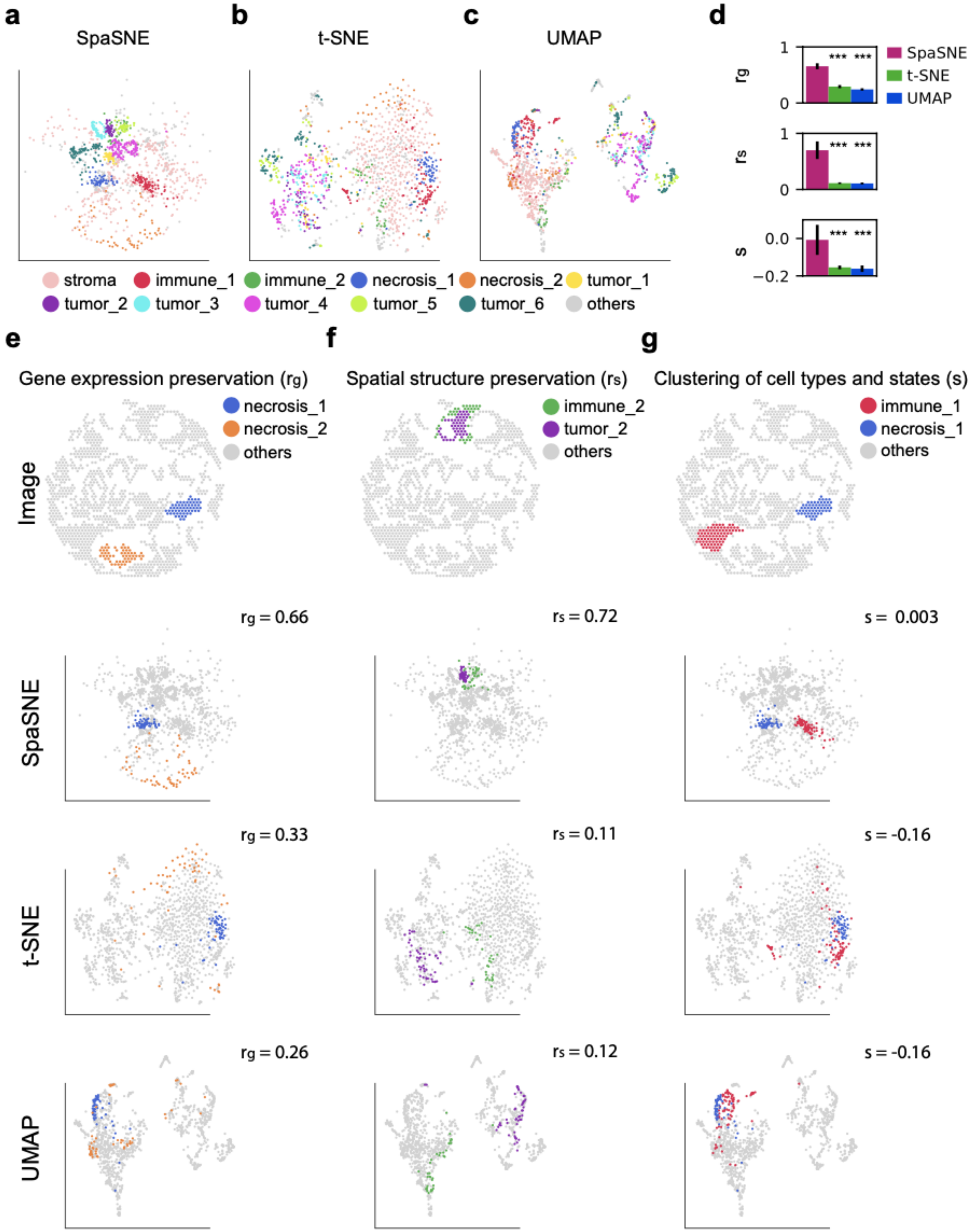

Figure 3. SpaSNE visualization of human prostate cancer data.

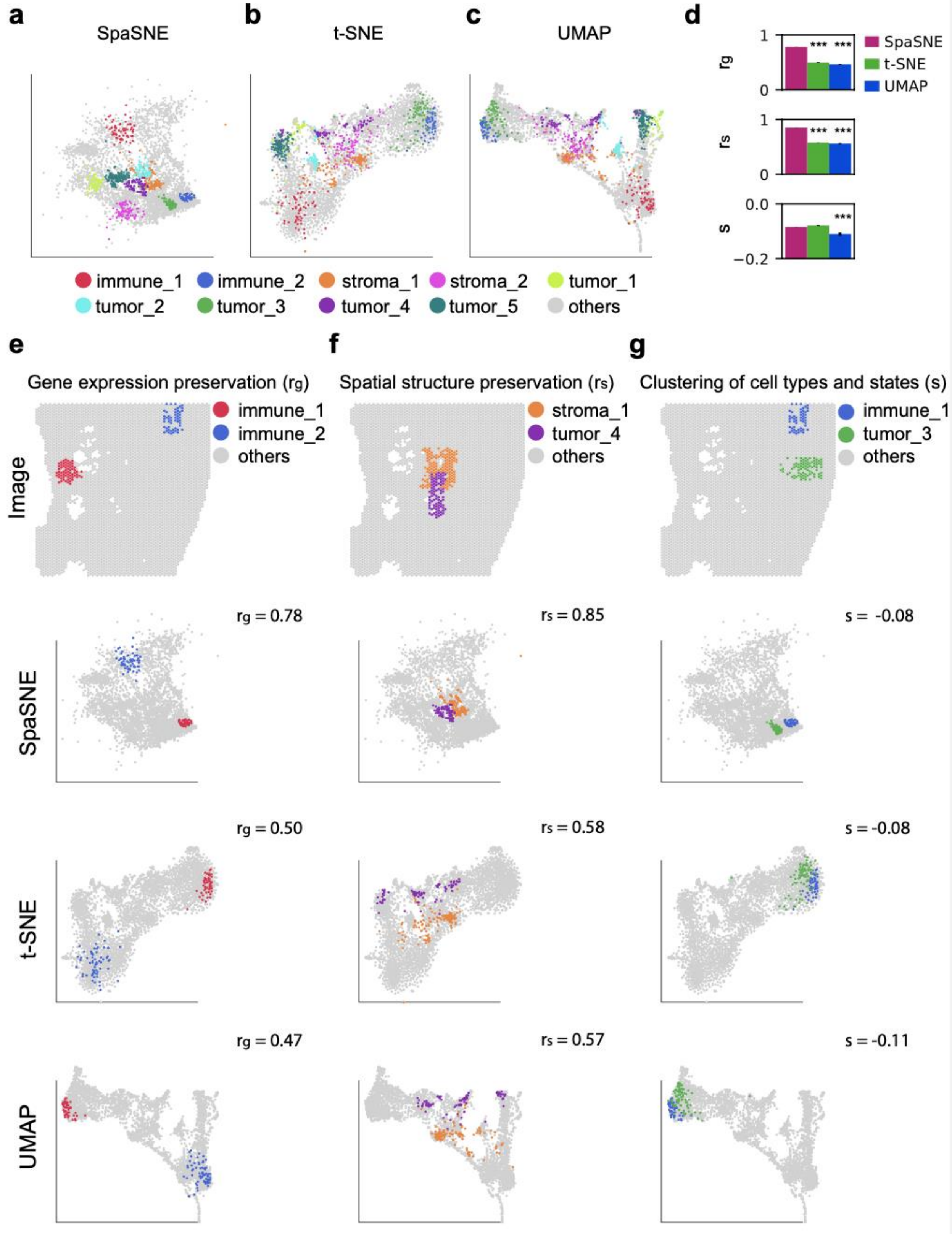

Figure 4. SpaSNE visualization of mouse visual cortex STARmap data

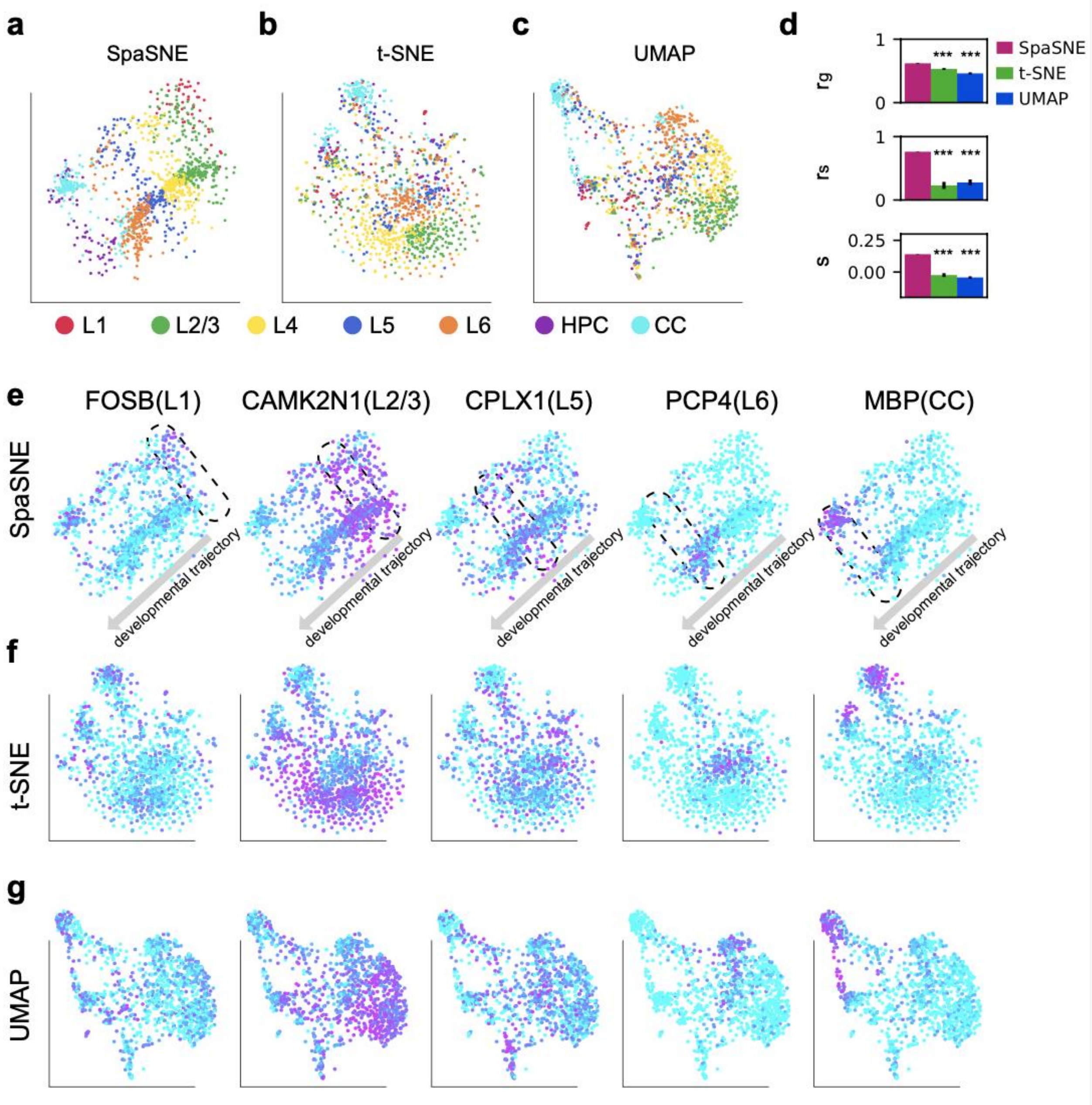

Figure 5. SpaSNE visualization of mouse hypothalamus MERFISH data

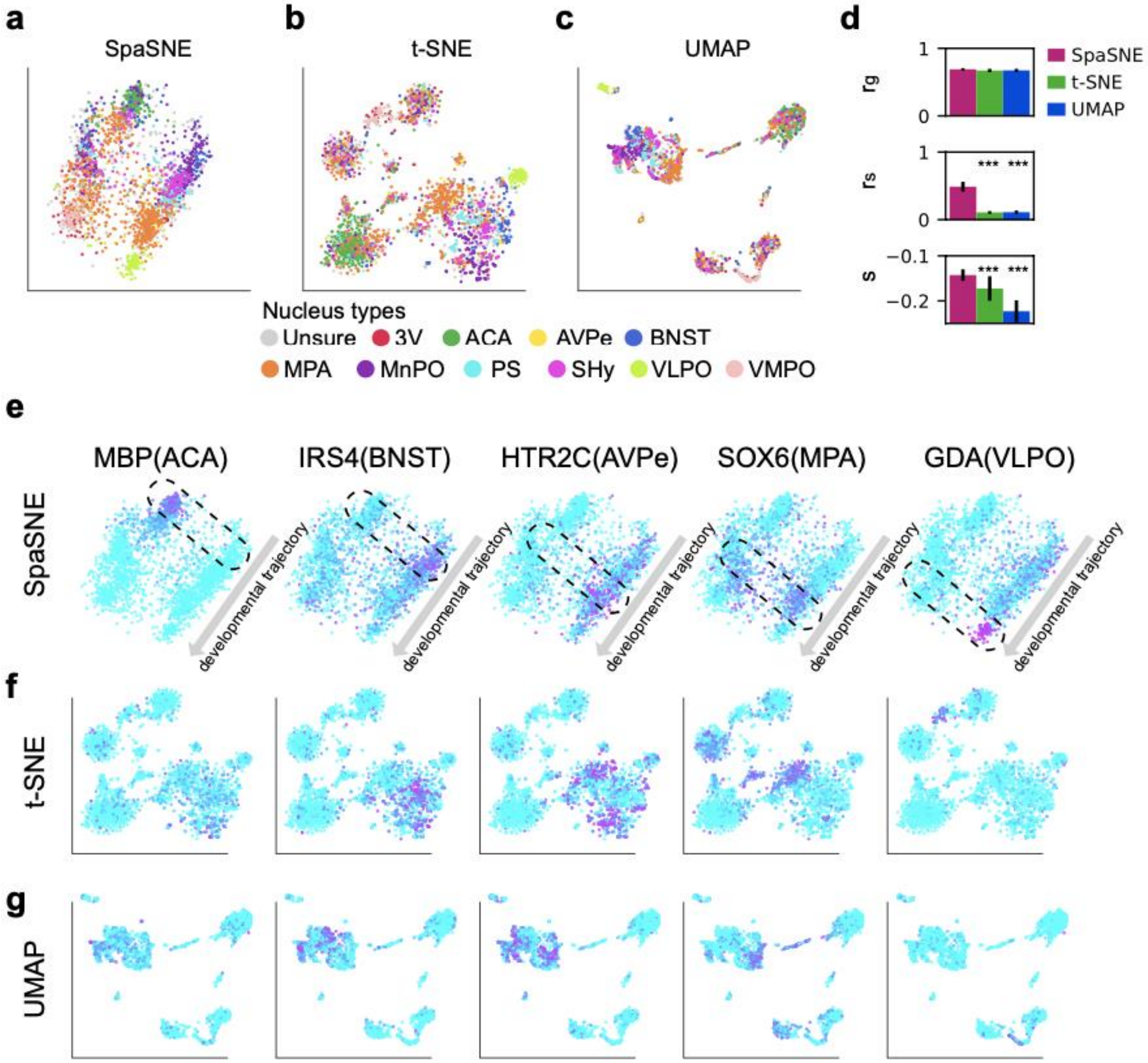

Click here to access/download
Supplementary Material
Supplementary Tables.xlsx

Click here to access/download
Supplementary Material
Supplementary Figures.pdf

Personal Cover Click here to access/download;Personal Cover;Point-by-point response.pdf

Authors’ Responses to the Initial Review of
“Dimensionality reduction for visualizing spatially resolved profiling data using SpaSNE”

Yuansheng Zhou, Chen Tang, Xue Xiao, Xiaowei Zhan, Tao Wang, Guanghua Xiao *, Lin Xu *

November 4, 2024

We sincerely thank the editor and the three reviewers for the opportunity to revise our work. In response to the feedback, we have substantially revised the manuscript and added 12 new supplementary figures. Major updates include demonstrating the necessity of preserving spatial information using SpaSNE, adding new biological analyses on the SpaSNE embedding results, and developing an automatic strategy for screening the two key parameters of SpaSNE. The reviewers' insightful comments have significantly enhanced the quality of our paper, and we hope the revised version is now suitable for publication in GigaScience. Below, we address each point the editor and reviewers raised, referencing the relevant sections, figures, and tables in the revised paper and supplementary materials. All changes in the manuscript have been highlighted for clarity.

Comments and Responses

<u>Reviewer #1</u> (Remarks to the Author)
In this manuscript, Zhou et al. introduce SpaSNE, an algorithm that builds upon Barnes-Hut t-SNE and applicable to spatial data. t-SNE is a non-linear dimensionality reduction methods which finds a 2D representation of high-dimensional data that approximately preserves input neighborhoods. This is achieved by optmizing for a KL-divergence loss between probability distributions in the high- and low-dimensional spaces, which are computed from distances between pairwise data points in the respective spaces. In SpaSNE, the authors add two new terms to the loss function, in addition to the one already present in regular t-SNE: one term favors the preservation of "long" distances (so that points that are far apart remain far apart) and one on "spatial distances" (distances from the spatial coordinates). These two new terms are weighted by hyperparameters. The authors apply SpaSNE to a variety of spatial transcriptomics datasets (from Visium, STARmap and MERFISH), and compare their results to t-SNE and UMAP, the most commonly used non-linear dimensionality reduction methods in computational biology. Qualitative analyses and three metrics (Pearson correlation of embedding distances vs spatial coordinates, Pearson correlation of embedding distances versus gene expression distances and silhouette coefficients) are used to compare the three methods. The authors conclude that SpaSNE is better than t-SNE and UMAP in accuracy and embeddings meaningfulness. The manuscript is clearly written and easy to follow. I however have some concerns in the comprehensiveness and fairness of the comparisons that are presented.

Comment 1: *One of the terms in SpaSNE's loss function aims at preserving spatial distances. It is not entirely clear to me what the goal of preserving spatial distances in embeddings is ? Spatial coordinates have low dimensionality (typically 2 in current omic experiments) and therefore do not need to be reduced. The authors show on a number of examples that "clusters" are better grouped in SpaSNE versus t-SNE and UMAP (as reflected by the Silhouette score), but it seems that these clusters are largely defined by spatial areas in the first place. I would like to recall that many current spatial transcriptomics are limited in their resolution to spots that aggregate multiple cells, which leads to crude phenotyping. Cells can be much more subtly phenotyped (in scRNAseq for instance), and it is likely that the next generation of spatial transcriptomics technologies, such as Visium HD, will have subcellular resolution. In that case, it seems that preserving spatial distances in the embeddings will be detrimental as it will compete with gene expression data.*

Response: We thank the reviewer for this insightful comment regarding the role of spatial distance preservation. Incorporating spatial information is important, as it provides essential biological context that enhances the understanding of cell states and tissue organization. We have added nine new figures and supporting descriptions to demonstrate the importance of spatial information preservation in SpaSNE, as detailed below:

First, preserving spatial information is important because it provides insights into cellular neighborhood interactions and tissue organization, both of which are biologically meaningful. For example, spatial data can reveal distinct cell states that interact with different neighboring cells, offering insights into tissue-specific marker gene expression. We added supplementary figures (Fig. S2-S4, S7-S12) to demonstrate that preserving spatial information allows detailed visualization of cell clusters with biologically relevant separation. The details of these new results were added to

the revised manuscript at lines 346-363 on pages 17-18, lines 436-444 on page 21, lines 491-496 on page 23, and lines 509-515 on page 24.

Second, we acknowledge the reviewer's point about the limitations of spatial transcriptomics. SpaSNE may not work effectively if the spatial information cannot contribute to the cell type identification or the global organization of cells in the tissue. We discussed this limitation of SpaSNE in lines 603-611 on page 28.

<u>For the reviewer's convenience, we have copied the new texts from the revised manuscript below. Please refer to Supplementary Figures S2-S4 and S7-S12 for additional details and visualizations of these results.</u>

Lines 346-363 on pages 17-18:

*The separation of cell clusters with different spatial locations is important because the cell states are influenced by their neighboring cells. For example, cells in tumor_2 and tumor_5, which are surrounded by immune cells and stroma cells respectively (**Supplementary Fig. S2a**), have distinct expression patterns of marker genes such as IFI27, LGALS3BP, and B2M (**Supplementary Fig. S2b-c**). The genes that are highly expressed in tumor_2 (surrounded by immune cells) are involved in biological processes related to immune responses with high enrichment scores in Gene Ontology analysis(**Supplementary Fig. S3a-c, -log10(p) >9**), while the genes that are highly expressed in tumor_5 (surrounded by stroma cells) are involved in translation activities with low enrichment scores(**Supplementary Fig. S3d-f, -log10(p)** < 7). Similarly, cells in "immune_2" and "immune_1", which are surrounded by tumor and stroma respectively (**Supplementary Fig. S2a**), have distinct expression patterns of marker genes such as ISG15, IFI6, and IFI27 (**Supplementary Fig. S2b-c**). The highly expressed genes in immune_2 are involved in immune responses while the highly expressed genes in immune_1 are involved in other biological processes (**Supplementary Fig. S4a-f**). These results showed that SpaSNE could produce a more delicate visualization that distinguishes different cell states of the same cell type that interact with different spatial environments by leveraging both gene expression and spatial information.*

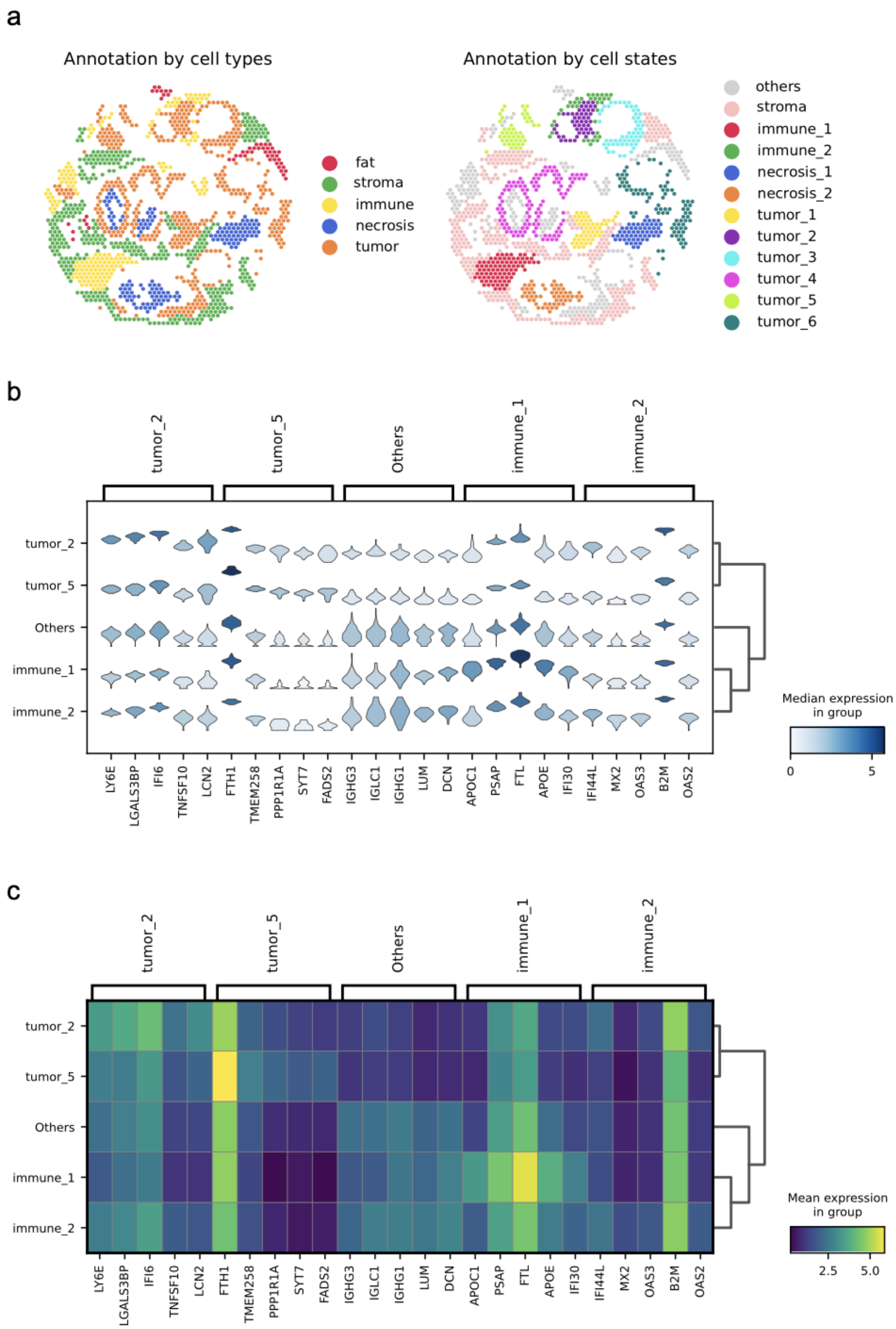

Supplementary Figure S2. Differential gene expressions of selected cell clusters in human breast cancer tissue. *(a) Annotation of human breast cancer cell clusters according to cell types (left) and cell states (right). The cell states annotation is the same as in Fig.2a-c in the manuscript. (b) Violin plots of top differentially expressed genes in tumor_2, tumor_5, immune_1, immune_2, and the rest cell types. (c) Mean expression values of top differentially expressed genes in the selected cell types.*

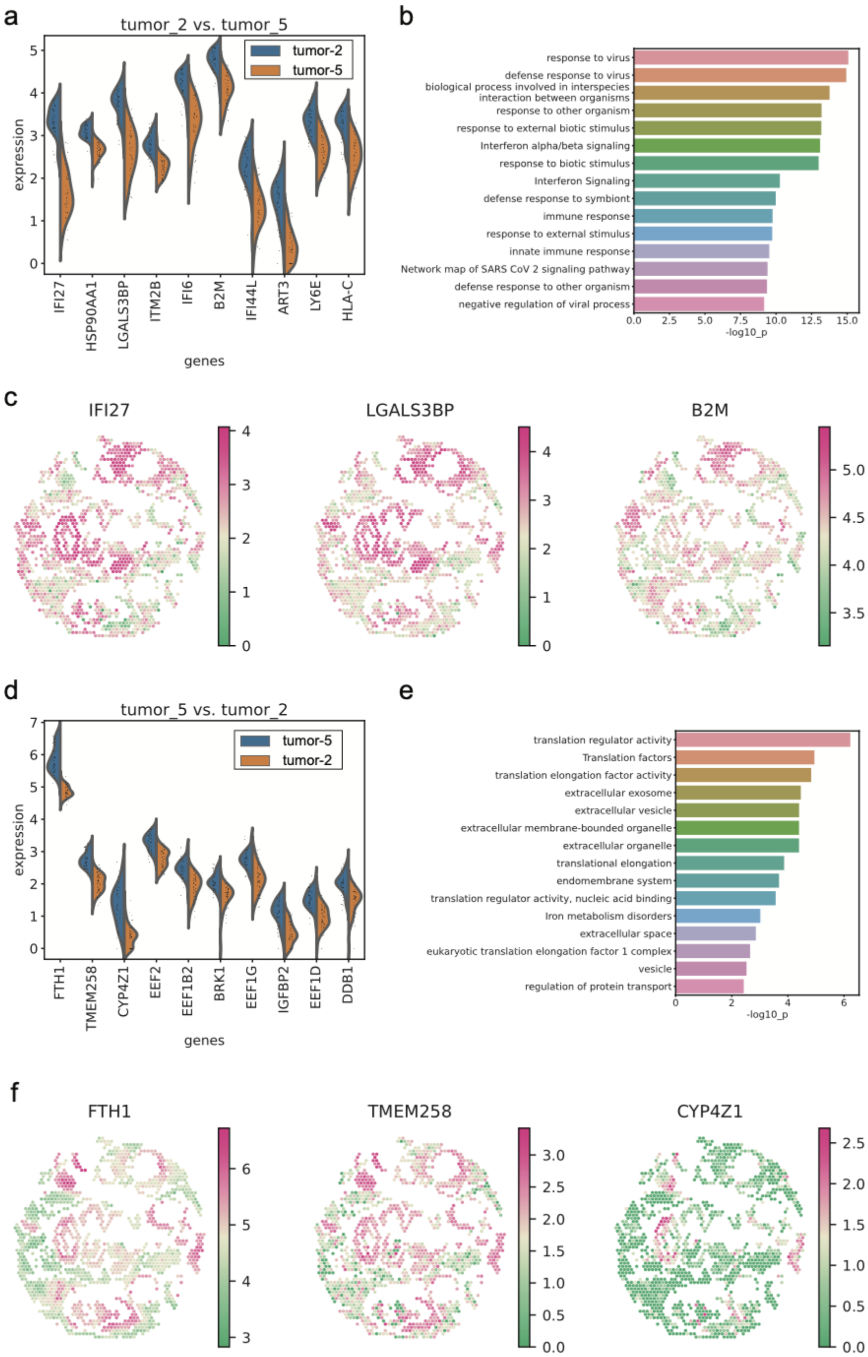

Supplementary Figure S3. Comparison of two tumor subtypes. *(a-c) Differentially expressed genes analysis with tumor_5 as reference. (a) Violin plots of the top differentially expressed genes. (b) The top 15 Gene Ontology Biological Processes obtained from the top 30 differentially expressed genes. (c) Expressions of three differentially expressed genes IFI27, LGALS3BP, and B2M. (d-f) Differentially expressed genes analysis with tumor_2 as reference. (d) Violin plots of the top differentially expressed genes. (e) The top 15 Gene Ontology Biological Processes obtained from the top 30 differentially expressed genes. (f) Expressions of three differentially expressed genes FTH1, TMEM258, and CYP4Z1.*

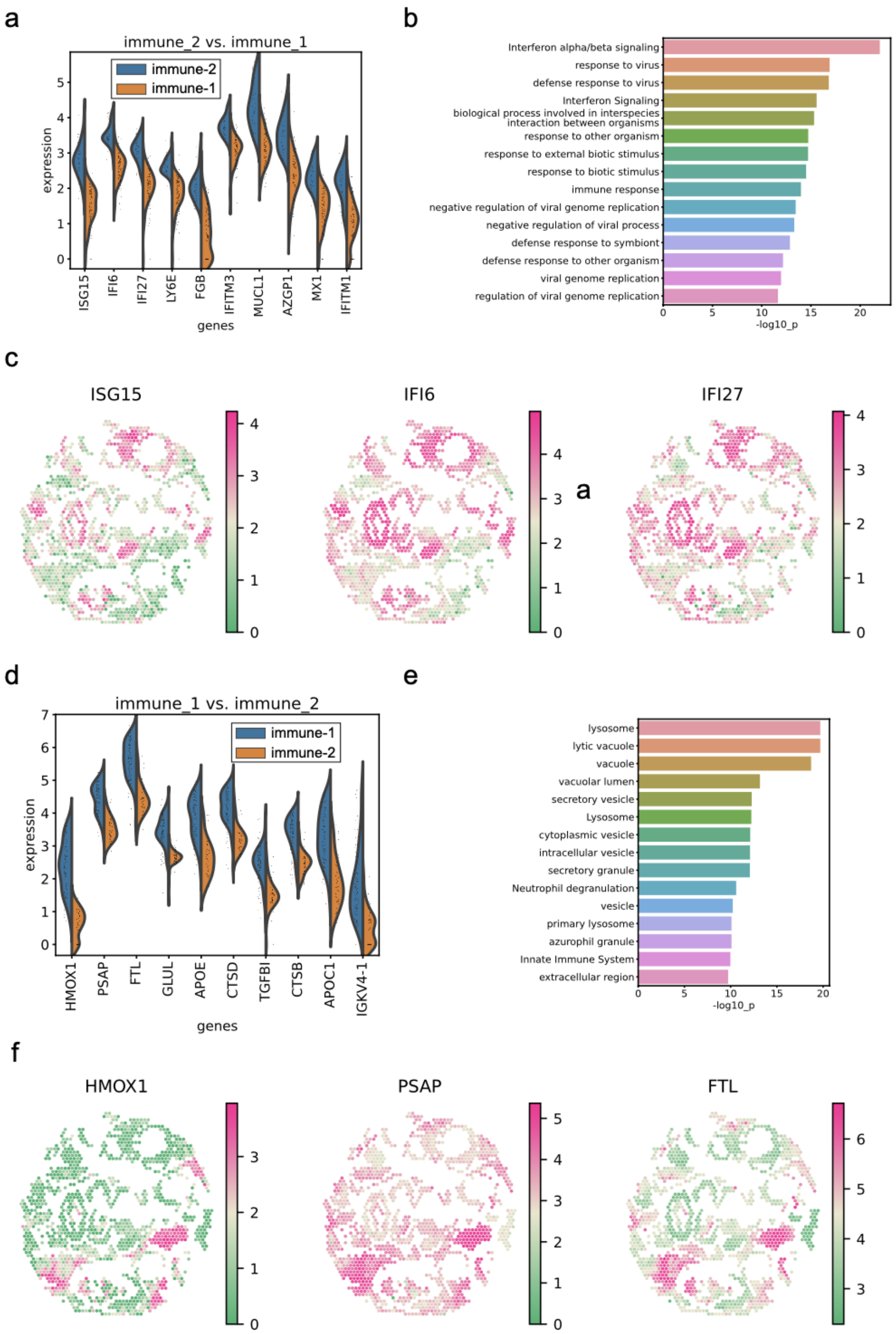

Supplementary Figure S4. Comparison of two immune subtypes. *(a-c) Differentially expressed genes analysis with immune_1 as reference. (a) Violin plots of the top differentially expressed genes. (b) The top 15 Gene Ontology Biological Processes obtained from the top 30 differentially expressed genes. (c) Expressions of three differentially expressed genes ISG15, IFI6, and IFI27. (d-f) Differentially expressed genes analysis with immune_2 as reference. (d) Violin plots of the top differentially expressed genes. (e) The top 15 Gene Ontology Biological Processes obtained from the top 30 differentially expressed genes. (f) Expressions of three differentially expressed genes HMOX1, PSAP, and FTL.*

The following text that describes Fig. S2, S3, and S4 were added to the revised manuscript at lines 436-444 on page 21:

The more delicate cell clusters separated by SpaSNE represent different cell states with different spatial environments. For example, cells in immune_2 and immune_1 are surrounded by tumor cells and stroma cells respectively, and have distinct expression patterns of marker genes such as CNN1, DES, and TMEFF2 (***Supplementary Fig. S7a-c*** *). The genes highly expressed in immune_2 cells are involved in biological processes including smooth muscle and smooth muscle cells, which play important roles in prostate cancer, with high enrichment scores (-log10(p) > 10) (* ***Supplementary Fig. S8a-c*** *). The genes highly expressed in immune_1 cells are involved in vesicle-related biological processes with low enrichment scores (-log10(p) < 4) (* ***Supplementary Fig. S8d-f*** *).*

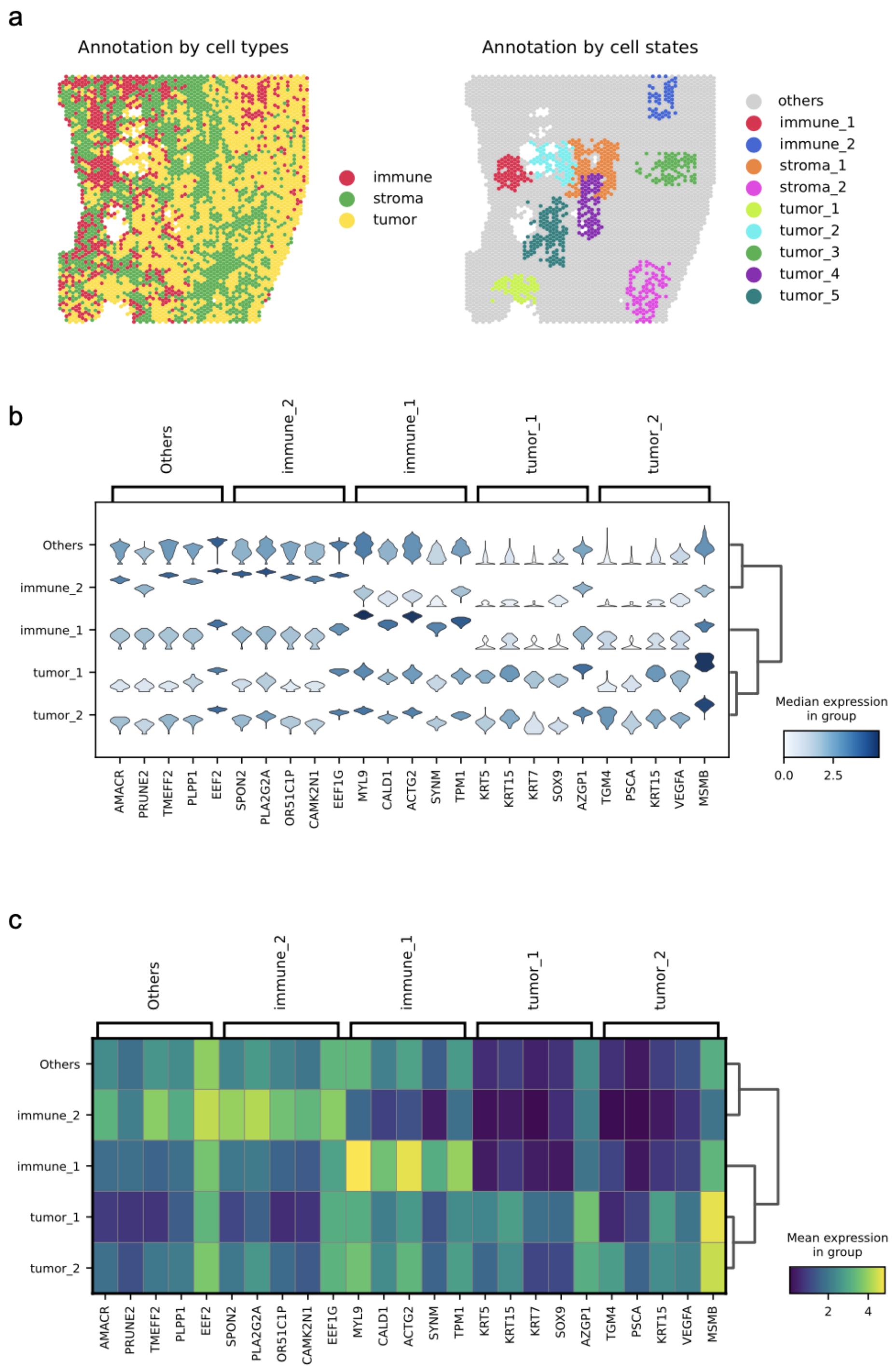

Supplementary Figure S7. Differential gene expressions of selected cell clusters in human prostate cancer tissue. *(a) Annotation of human prostate cancer cell clusters according to cell types (left) and cell states (right). This annotation is the same as in Fig.3a-c in the manuscript. (b) Violin plots of top differentially expressed genes in tumor_1, tumor_2, immune_1, immune_2, and the rest cell types. (c) Mean expression values of top differentially expressed genes in the selected cell types.*

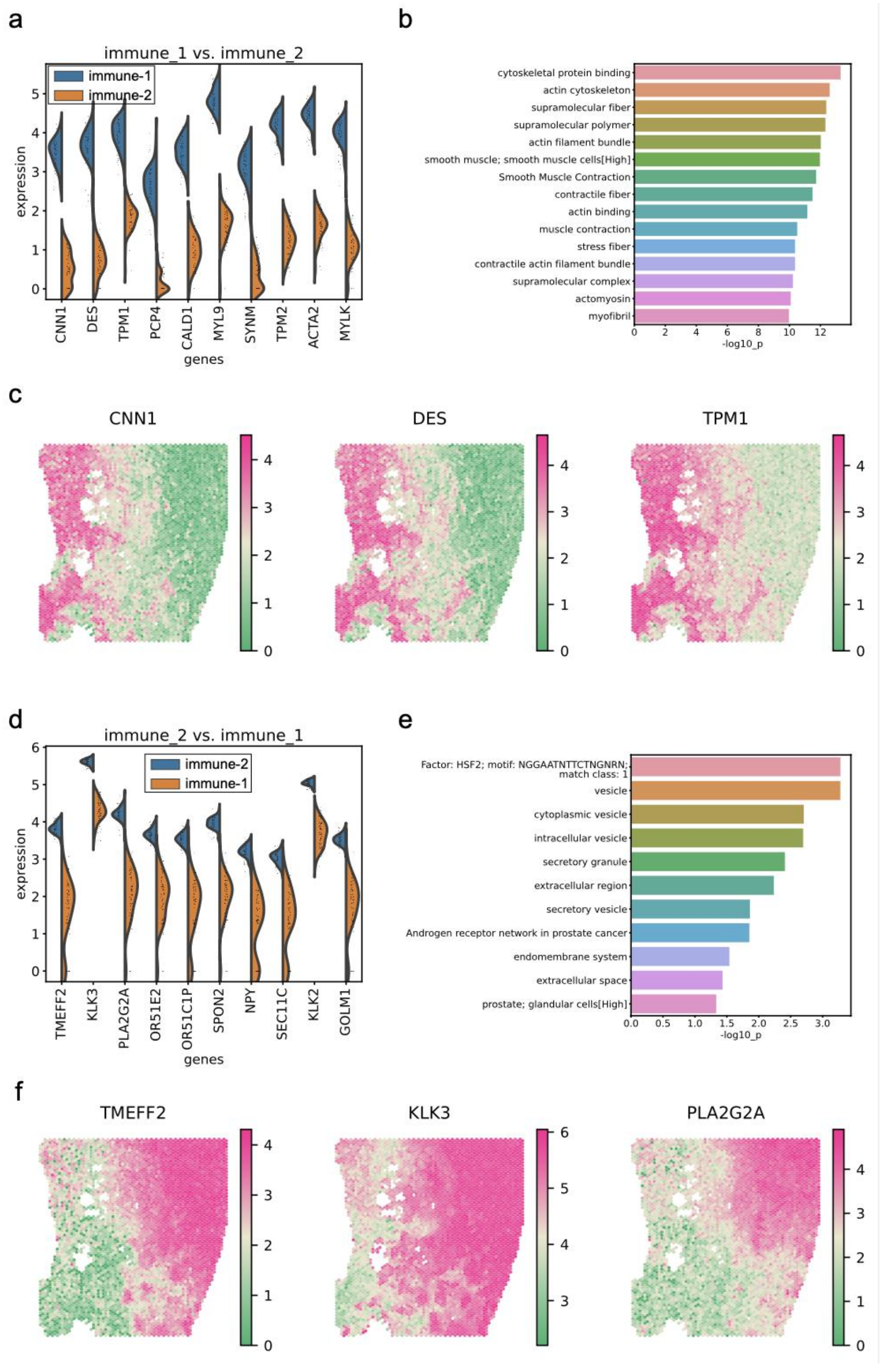

Supplementary Figure S8. Comparison of two immune subtypes. *(a-c) Differentially expressed genes analysis with immune_2 as reference. (a) Violin plots of the top differentially expressed genes. (b) The top 15 Gene Ontology Biological Processes obtained from the top 30 differentially expressed genes. (c) Expressions of three differentially expressed genes CNN1, DES, and TPM1. (d-f) Differentially expressed genes analysis with immune_1 as reference. (d) Violin plots of the top differentially expressed genes. (e) The top 15 Gene Ontology Biological Processes obtained from the top 30 differentially expressed genes. (f) Expressions of three differentially expressed genes TMEFF2, KLK3, and PLA2G2A.*

The following text that describes Fig. S9 and S10 were added to the revised manuscript at lines 491-496 on page 23.

Lines 491-496 on page 23:

*Here, we performed differential expression analysis based on the six layers and found that the differentially expressed genes are involved in biological processes including system development and neurogenesis (**Supplementary Fig. S9a-c**). By comparing each layer with the rest layers, we identified layer-specific markers genes and selected five of them for visualization: FOSB(L1), CAMK2N1(L2/3), CPLX1(L5), PCP1(L6) and MBP(CC) (**Supplementary Fig. S10**).*

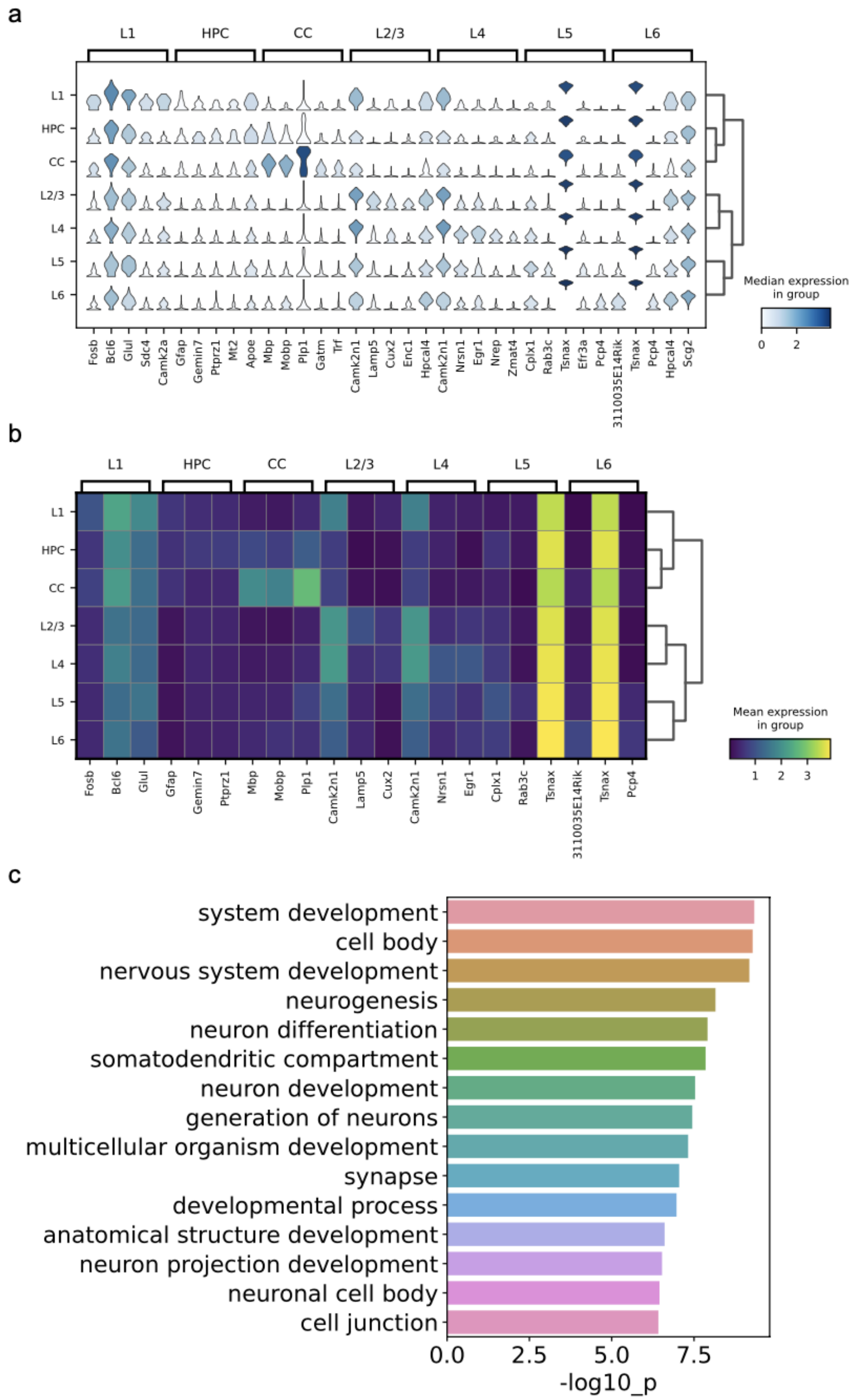

Supplementary Figure S9. Differential gene expressions of seven layers in mouse visual cortex tissue. *(a) Violin plots of top differentially expressed genes in the seven layers. (b) Mean expression values of top differentially expressed genes in the seven layers. (c) The top 15 Gene Ontology Biological Processes obtained from 70 differentially expressed genes.*

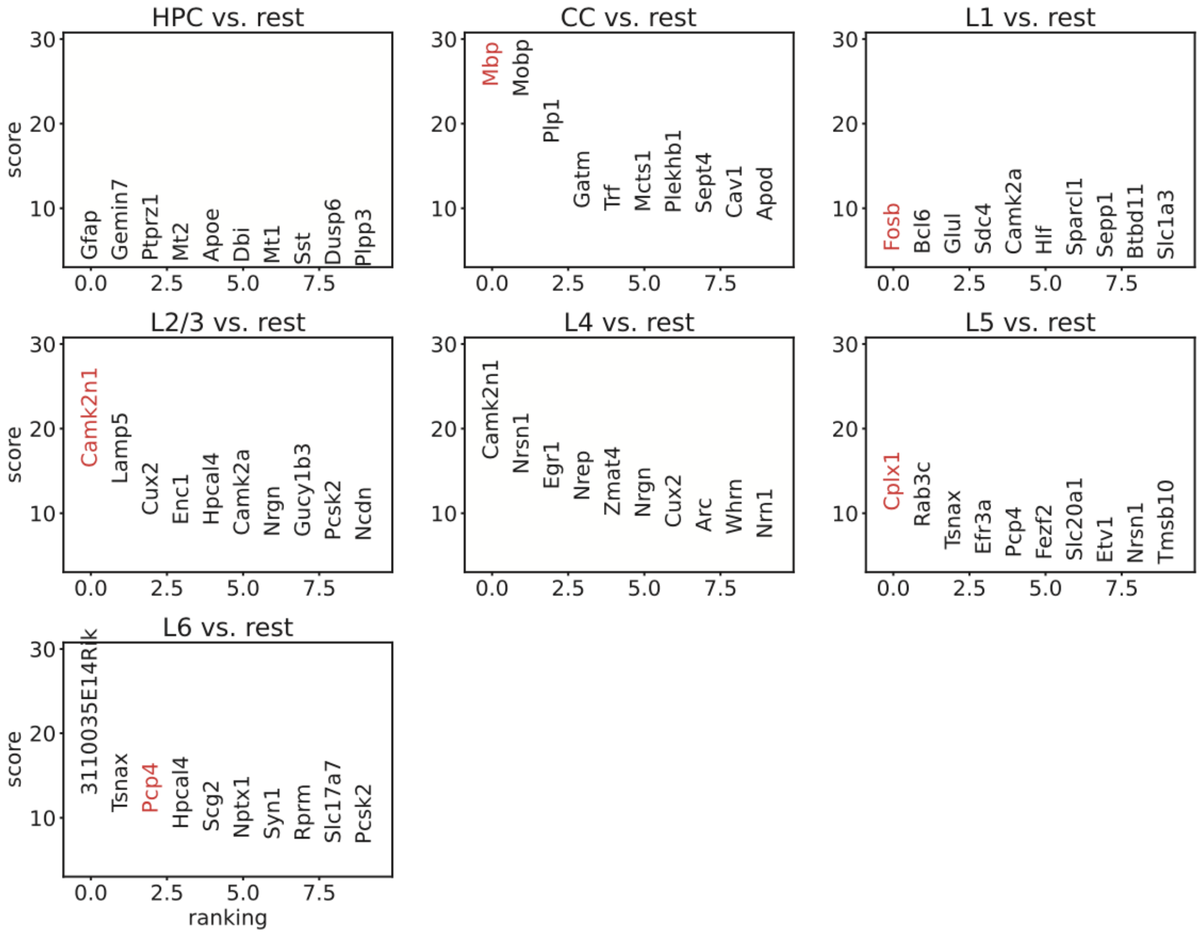

Supplementary Figure S10. Differentially expressed genes from the comparison between each layer type and the rest layers in mouse visual cortex dataset. *The genes marked red are the genes shown in Fig. 4e-g in the manuscript.*

The following text that describes Fig. S9-S12 were added to the revised manuscript at lines 509-515 on page 24.

Lines 509-515 on page 24:

*Following the analysis in **Supplementary Fig. S9-S10**, we performed differential expression analysis based on the eleven nucleus types and found that the differentially expressed genes are involved in biological processes including multicellular organismal process and nervous system development (**Supplementary Fig. S11a-c**). By comparing each layer with the rest layers, we identified layer-specific markers genes and selected five of them for visualization: MBP(ACA), IRS4(BNST), HTR2C (AVPe), SOX6(MPA), and GDA(VLPO) (**Supplementary Fig. S12**).*

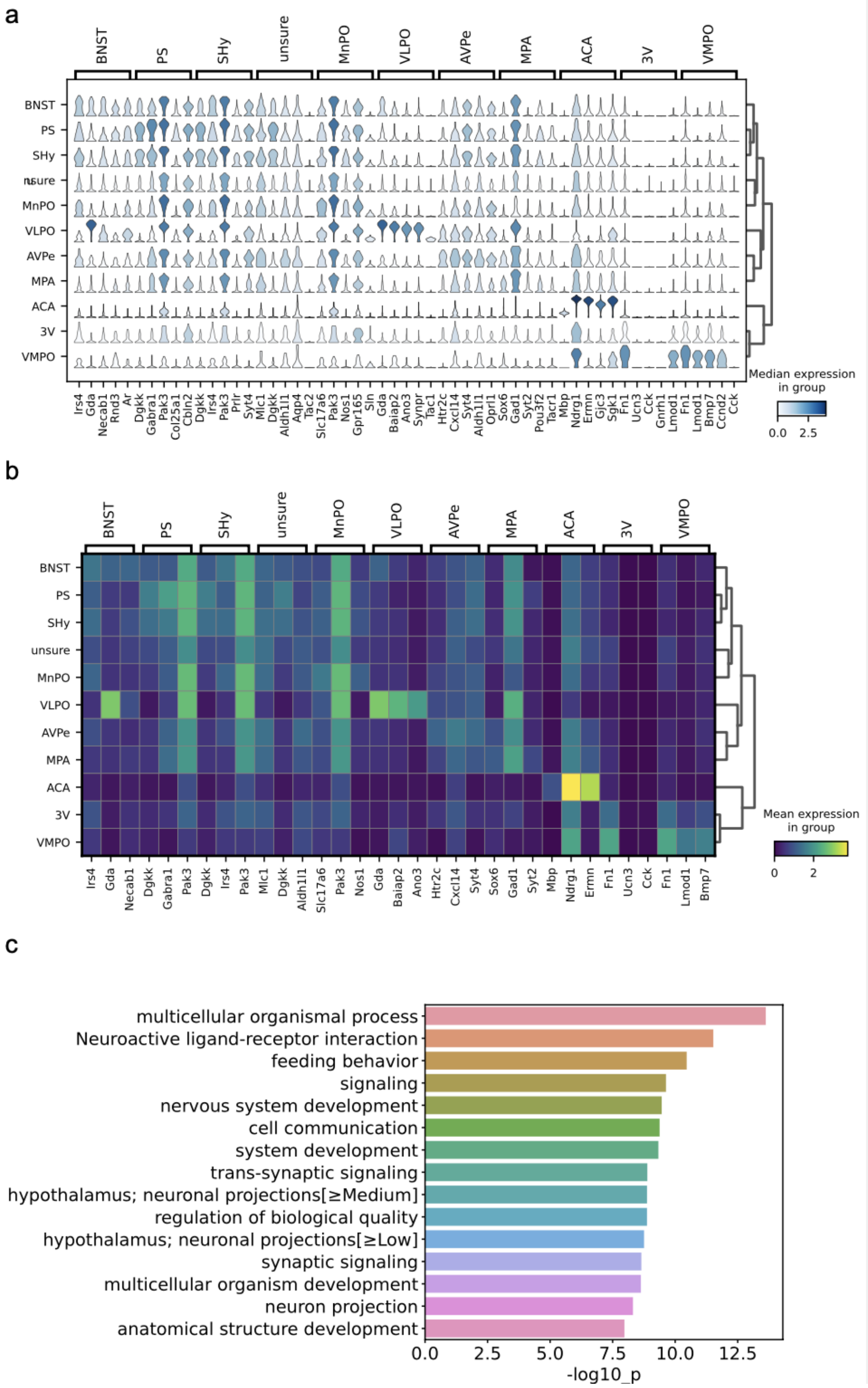

Supplementary Figure S11. Differential gene expressions of seven layers in mouse hypothalamus tissue. *(a) Violin plots of top differentially expressed genes in the seven layers. (b) Mean expression values of top differentially expressed genes in the seven layers. (c) The top 15 Gene Ontology Biological Processes obtained from 90 differentially expressed genes.*

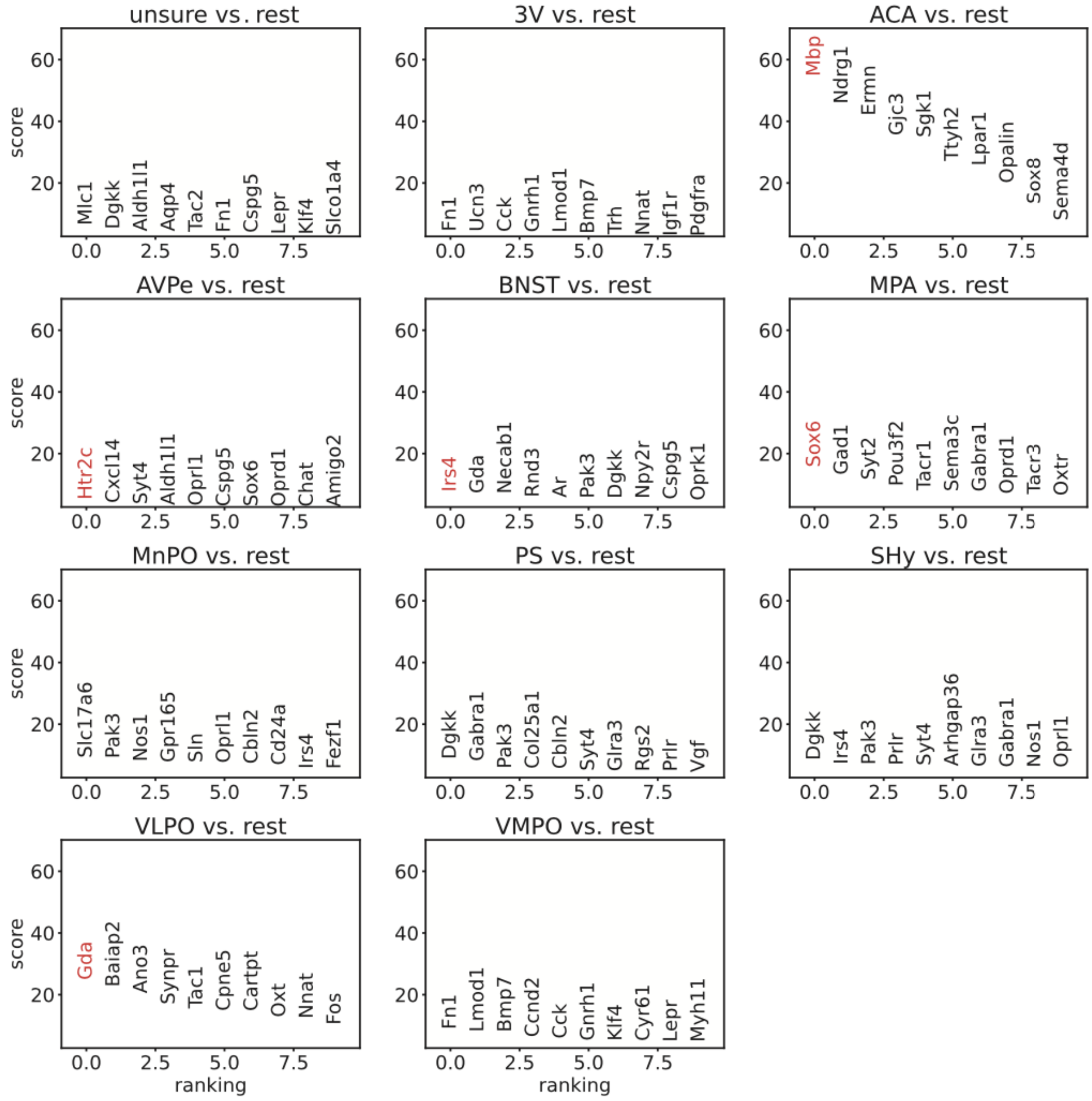

Supplementary Figure S12. Differentially expressed genes from the comparison between each layer type and the rest layers in mouse hypothalamus data. *The genes marked red are the genes shown in Fig. 5e-g in the manuscript.*

The following text that describes Fig. S9-S12 were added to the revised manuscript at lines 603-611 on page 28.

Lines 603-611 on page 28:

Fourthly, the design of SpaSNE assumes that the spatial information in spatially resolved transcriptomics data will contribute to the identification of cell states or the global organization of the cells. It may be less effective when there is no correlation between the phenotype and spatial positions of cells. In addition, its performance may be compromised in situations where spatial transcriptomics measurements are taken at multicellular resolution where a single spot contains multiple cell types, or in subcellular resolution where a single cell covers multiple spatial positions such as Visium HD data. For the latter case, we may adapt SpaSNE to analyze such datasets by incorporating current decomposition or aggregation methods for the preprocessing of data in the future.

Comment 2: *The two new terms in the loss function come at the cost of two extra hyperparameters which is a major disadvantage. The authors show very limited data on the sensitivity of their results to these hyperparameters - in figures S3 and S4. The values shown in S3 and S4 are however extreme, with one of the coefficients set to 0 (thus entirely ignoring the corresponding term in the loss function). I think the authors should, for a fixed initialization, present embeddings obtained from hyperparameters spanning the whole range of recommended values ([6;15] for alpha, [1;7.5] for beta) across datasets. In their comparisons, the authors use t-SNE and UMAP with default parameters but adjust SpaSNE's hyperparameters for each dataset. This increases the risk of biased results. When investigating embeddings for different hyperparameters values, the same metrics could be computed and compared with those obtained from default UMAP and t-SNE. This will be a fairer summary of the method's performance.*

Response: We appreciate the reviewer's suggestion to further examine the sensitivity of the hyperparameters in SpaSNE. To evaluate the sensitivity of α and β, we analyzed a broader range of values of the two parameters ($\alpha \in [2, 50], \beta \in [1, 25]$) with fixed initialization for four datasets in a new **Supplementary Figure S16**. This additional data demonstrates how the embeddings of SpaSNE depend on the selection of parameters.

We have also implemented a two-stage heuristic approach (rough screening followed by fine screening) to provide a systematic and reproducible method for identifying optimal parameter values of SpaSNE (**Supplementary Fig. S16b, d, f, h**). The details of this revised parameter screening process are included in the updated manuscript on lines 229-258 on pages 12-13. We also provide a detailed, step-by-step tutorial on this screening approach for the human breast cancer dataset, which can be found on our GitHub page (https://github.com/Lin-Xu-lab/SpaSNE).

We computed the same metrics (Pearson correlation coefficients and silhouette scores) used to evaluate t-SNE and UMAP, applying these consistently across all methods and datasets.

For the reviewer's convenience, we have included the new manuscript text below. Please refer to **Supplementary Figure S16** for visual representations of the rough and fine screenings for each dataset.

Lines 229-258 on pages 12-13:

*To determine the optimal combination of parameters for a given data, we developed a heuristic screening approach that consists of two stages: rough screening and fine screening. In rough screening, we screened the two parameters on a larger scale to determine the range where the optimal parameters may fall (**Supplementary Fig. S16a, c, e, g**). In fine screening, we determined the optimal parameter with a finer resolution (**Supplementary Fig. S16b, d, f, h**). Here we use the example of the human breast cancer dataset to demonstrate the two-stage screening process in detail:*

1. *Running 100 repeats of t-SNE with default parameters on human breast cancer datasets and calculating (r_g, r_s) for each repeat. The maximal value of r_g is marked as r_{thres}.*
2. *Performing rough screening with SpaSNE.*

2.1. *Taking α and β from* $\{(\alpha, \beta)|\ \alpha \in [2, 5, 10, 20, 30, 50], \beta \in [1, 5, 10, 15, 25]\}$.

2.2. *In each parameter combination, running 10 repeats of SpaSNE. Setting* $r_g = 0$ *if* $r_g \leq r_{thres}$ *in each repeat. Calculating* (r_g, r_s) *for each repeat. Selecting the optimal embedding that gives the maximal value of* $r_g \times r_s$ *in the 10 repeats and recording the optimal* r_g^{opt} *and* r_s^{opt}.

2.3. *Showing the values of* $r_g^{opt} \times r_s^{opt}$ *for all the parameter combinations by heatmap (**Supplementary Fig. S16a**).*

3. *Performing fine screening with SpaSNE.*

3.1. *Based on the heatmap results in step 2.3, selecting the range where the optimal parameters may fall:* $\{(\alpha, \beta)|\ \alpha \in [5, 6, 7, .., 20],\ \beta \in [1, 2, 3, \ldots, 10]\}$.

3.2. *In each parameter combination, running 20 repeats of SpaSNE. Setting* $r_g = 0$ *if* $r_g \leq r_{thres}$ *in each repeat. Calculating* (r_g, r_s) *for each repeat and standard deviation* (std) *of* r_g *of the 20 repeats. Selecting the optimal embedding that gives the maximal value of* $r_g \times r_s$ *in the 20 repeats and recording the optimal values* r_g^{opt}, r_s^{opt} *and* std .

3.3. *Showing the values of* $r_g^{opt} \times r_s^{opt}$ *(left),* std *(middle) and* $r_g^{opt} \times r_s^{opt} \times exp\,(1 - std)$ *(right) for all the parameter combinations by heatmap (**Supplementary Fig. S16b**).*

4. *Determining the optimal parameter combination by selecting the maximal value of* $r_g^{opt} \times r_s^{opt} \times exp\,(1 - std)$ *obtained in 3.3.*

5. *Running 100 repeats of SpaSNE with the optimal parameter combination obtained in step 4 and selecting the embedding with the maximal values of* $r_g \times r_s$.

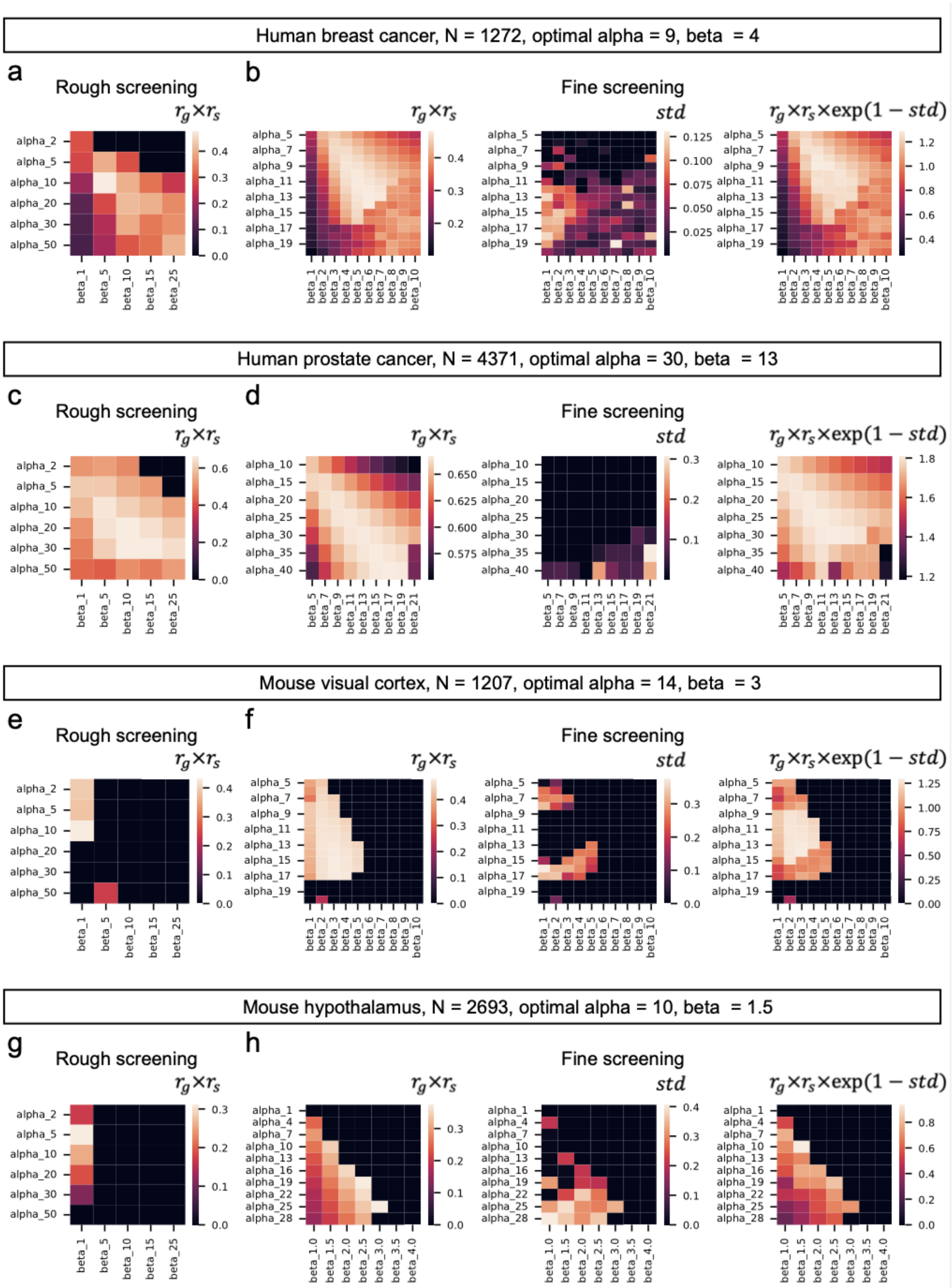
Human breast cancer, N = 1272, optimal alpha = 9, beta = 4
a
Rough screening
$r_g \times r_s$
b
Fine screening
$r_g \times r_s$
std
$r_g \times r_s \times \exp(1-std)$
Human prostate cancer, N = 4371, optimal alpha = 30, beta = 13
c
Rough screening
$r_g \times r_s$
d
Fine screening
$r_g \times r_s$
std
$r_g \times r_s \times \exp(1-std)$
Mouse visual cortex, N = 1207, optimal alpha = 14, beta = 3
e
Rough screening
$r_g \times r_s$
f
Fine screening
$r_g \times r_s$
std
$r_g \times r_s \times \exp(1-std)$
Mouse hypothalamus, N = 2693, optimal alpha = 10, beta = 1.5
g
Rough screening
$r_g \times r_s$
h
Fine screening
$r_g \times r_s$
std
$r_g \times r_s \times \exp(1-std)$

Supplementary Figure S16. Rough and fine screenings of parameters α and β of SpaSNE on the four datasets.

For each dataset, a rough screening was first performed to determine the range of optimal parameters on a large scale (a, c, e, g), then a fine screening was performed to find the optimal parameters with a fine resolution (b, d, f, h). In each embedding, setting $r_g = 0$ *if* $r_g \leq r_{thres}$, *where* r_{thres} *is the maximal* r_g *in 100 repeats of t-SNE embeddings. The heatmaps for rough tuning represent values of* $r_g \times r_s$ *with different combinations of parameters. The heatmaps for fine tuning represent values of* $r_g \times r_s$ *(left),* std *(middle) and* $r_g \times r_s \times exp\,(1 - \mathrm{std})$*(right). The* std *is the standard deviation of* r_g *in multiple repeats of SpaSNE embeddings in each parameter combination. The parameter ranges used in screening and optimal parameters for the four datasets can be found in* ***Supplementary Table S1****. (a-b) Rough (a) and fine (b) screening on human breast cancer data. (c-d) Rough (c) and fine (d) screening on human prostate cancer data. (e-f) Rough (e) and fine (f) screening on mouse visual cortex data. (g-h) Rough (g) and fine (h) screening on mouse hypothalamus data.*

Comment 3: The authors mention that their recommended hyperparameter values provide the best performance in terms of reproducibility of the embeddings (among other things) but nowhere in the manuscript is this reproducibility aspect discussed - how did the authors mention reproducibility and how is it affected by hyperparameter values?

Response: Thank you for this valuable comment and we appreciate the opportunity for clarification. By "reproducibility," we refer to the "stability" of the embeddings generated by SpaSNE across multiple runs with the same parameter settings. In response to the reviewer's comment, we have now replaced the term "reproducibility" with "stability" in the revised manuscript and expanded the discussion on this topic. We have added this explanation to the manuscript in lines 222-227 on page 12, along with Supplementary Figure S16, which illustrates stability across different parameter values.

For the reviewer's convenience, the revised text is included below. Please see **Supplementary Figure S16** for detailed stability assessments.

Lines 222-227 on page 12:

*In addition to the ratio of α and β, the magnitude of α and β may also influence the stability of the embedding because the contribution of the local cost function L_t in the original t-SNE will be weakened by a large α and β (Eq. 11) and the embedding will become more unstable, especially when the dataset size is small. We measured stability by the standard deviation (std) of r_g in multiple repeated embeddings with a given set of parameters in SpaSNE (**Supplementary Fig. S16b, d, f, h**).*

Comment 4: The s^{hat}_{ij} (Equation 9) terms are defined from a (normalized) Cauchy / Student t-distribution of the squared euclidean distance. The squared euclidean distance is not bounded and the s^{hat}_{ij} will therefore be heavily influenced by the most distant pairs of data points. I therefore question whether this is a sensible loss function to preserve "long" distances as it will probably only preserve "farthest" distances, which may not be typically interesting? This intuition seems confirmed based on supplementary figure S3c (and to a lesser extent S4c) where a large number of singleton outliers seem present with alpha = 10 and beta = 0.

Response:
We appreciate this insightful comment. It's true that $\hat{s}_{ij}$ will be heavily influenced by the most distant pairs of data points, it also preserves other long distances of pairs of points, although to a lesser degree. This principle is similar to that used in local similarity term p_{ij} (Equation 1-2): p_{ij} will be heavily influenced by the nearest pairs of data points, but it can also capture other small distances of pairs of points with a lesser degree.

The scattering orange points (necrosis_2) in the original **Supplementary Figure S3** (now it's **Supplementary Figure S6**) are not outliers. Instead, the scattering distribution reflects the large variance of gene expressions of the cells in the necrosis_2 cluster (**Supplementary Figure S5a**). This result demonstrates that large alpha helps preserve the transcriptomic variability of cells by preserving the large distances of gene expressions.

Comment 5: How are each method initialized? What's the stopping criterion in SpaSNE?

Response: Thank you for your question. All three methods—SpaSNE, t-SNE, and UMAP—use their default initialization settings. Specifically, UMAP is initialized using a spectral embedding of the fuzzy 1-skeleton, while both t-SNE and SpaSNE are initialized using a truncated eigenvector matrix with a dimensionality of 50. This choice of initialization ensures a fair comparison and comparable results across datasets. The stopping criterion for SpaSNE is the same as t-SNE which stops when the maximal iteration (1000 by default) is reached. We have included these details in the revised manuscript at lines 274-277 on page 14. For the reviewer's convenience, the revised text is provided below.

Lines 274-277 on page 14:

All three algorithms were initialized with default setting: UMAP was initialized using a spectral embedding of the fuzzy 1-skeleton, both t-SNE and SpaSNE were initialized with truncated eigen-vectors matrix with a dimension of 50. The stopping criterion for SpaSNE is the same as t-SNE which stops when the maximal iteration (1000 by default) is reached.

Comment 6: How were cell / spots assigned phenotyped in each dataset?

Response: The annotations were obtained from original publications or websites of the human breast cancer, mouse visual cortex, and mouse hypothalamus datasets. For the human prostate cancer dataset, we used the HD-Staining algorithm to classify cell nuclei and types based on pathology image features. Metadata files, including spatial positions and cell type labels for each cell/spot, are available in Supplementary Tables S2-S5. We have clarified this in the revised manuscript at lines 117-123 on page 7. For the reviewer's convenience, the revised text is provided below.

Lines 117-123 on page 7:

The annotations of the human breast cancer, mouse visual cortex, and mouse hypothalamus datasets were obtained from original papers or websites (the details are included in the "Data availability" section). The human prostate cancer dataset was annotated by the HD-Staining algorithm developed for classifying cell nuclei and cell types in the pathology images. The annotations of the four datasets were provided in the metadata files in ***Supplementary Tables S2-S5****. The metadata includes the spatial positions and different kinds of cell type labels for each cell/spot.*

Comment 7: What preprocessing steps were applied to each dataset? The authors mention that in the breast cancer dataset, 1272 spots were selected. How and why were these spots selected?

Response: Thank you for this question regarding data preprocessing. We have clarified the preprocessing steps for each dataset in the revised manuscript.

For each dataset, we applied standard preprocessing, including normalization using the "scanpy" Python package, which adjusted each cell or spot to a uniform total count based on the median across all cells, followed by a natural log transformation. Dimensionality reduction was then performed using 200 principal components for the human breast cancer, human prostate cancer, and mouse visual cortex datasets, while the full 161 genes were retained for the mouse hypothalamus dataset without dimensionality reduction.

For the human breast cancer dataset, a pathologist annotated 1,272 of the 2,518 total spots, and only these annotated spots were selected for analysis to ensure that the embedding results were based on ground-truth labels. We included these details in the revised manuscript at lines 102-114 on page 7. For the reviewer's convenience, the revised text is provided below.

Lines 102-114 on page 7:

The four spatial gene expression datasets used in the manuscript were presented in ***Supplementary Table 1*** *(the details are also included in the "Data availability" section). For the human breast cancer dataset, there are a total of 2,518 spots in the image, but only 1,272 of them were annotated by a pathologist and the rest spots cannot be determined. We selected these 1,272 spots to evaluate the performance of our algorithm based on the ground truth annotation. For the mouse hypothalamus dataset, we took the left side of the whole slide, which contains 2,693 cells. The annotations of the four datasets were provided in* ***Supplementary Tables S2-5****. Given a unique molecular identifier (UMI) count matrix, we first used the "scanpy" Python package to normalize the counts. Each cell or spot has a total count equal to the median of total counts per cell. We then transformed them to a natural log scale. For the human breast cancer, human prostate cancer, and mouse visual cortex datasets, we reduced the dimensionality to 200 principal components prior to performing the embedding. For the mouse hypothalamus dataset, we used all 161 genes without dimensionality reduction.*

Comment 8: The metrics (r_g, r_s and s) that the authors use are in my opinion problematic: r_g and r_s measure the conservation of the expression and spatial distances, and therefore mirror the loss function terms. I think that a metric sensitive to local neighborhood preservation (e.g. k nearest neighbors retention) should also be shown for the three methods. The silhouette coefficient is also problematic : it measures how "tight" a spot/cell cluster is in the embeddings. It however appears that in most (all) of the datasets used, clusters are - at least partially - defined by spatial characteristics. Again, this is a current technological limitation of omic technologies but not something that seems desirable - clustering cells by phenotype seem more appealing. Sadly, the only datasets with (sub)cellular resolution shown (the MERFISH and STARmap datasets) are from brain regions where phenotype and spatial coordinates are heavily correlated (along a one-dimensional axis for the STARmap dataset and in 2D areas for the MERFISH dataset). This would not be the case in most organs when analyzing a sizable piece of tissue : the authors for instance had to exclude half of the hypothalamus dataset for that reason.

Response: We appreciate this constructive suggestion and agree that additional metrics can provide a more comprehensive evaluation of SpaSNE's performance. We applied the metric of "trustworthiness" in "sklearn" Python package to measure the preservation of the local structure of both spatial positions and gene expressions. We found that SpaSNE achieves higher trustworthiness of spatial structure and lower trustworthiness of transcriptomic structure than t-SNE, but the product of the two trustworthiness scores is higher in SpaSNE. We showed the results at lines 198- 208 on page 11 and **Supplementary Fig. S15**.

The silhouette coefficient is usually used as an unsupervised method to measure the tightness of clusters. However, when using the ground truth annotation as the predicted labels, it can be used as a supervised metric to measure the consistency between the clusters of embedding points and the ground-truth annotation of points. When the ground truth annotation is not available, the product of the two trustworthiness scores might be used as an alternative metric to replace the silhouette coefficient.

We appreciate the concern that SpaSNE may not work well when the phenotype and spatial positions are not correlated. We discussed this limitation in lines 603-616 on page 28.

For the reviewer's convenience, the revised text is provided below.

Lines 198- 208 on page 11:

*This [silhouette coefficient] metric was widely used as an evaluation tool for clustering quality analysis. When using the ground truth annotation as the predicted cluster labels, it measures the consistency between the clusters of embedding points and the ground-truth annotation of points. An alternative method of evaluating the embedding quality is the "trustworthiness" metric which measures the preservation of the local structure of data. For spatially resolved data, both the local structure of spatial positions and gene expressions should be considered. The design of SpaSNE leads to increased trustworthiness of spatial structure and decreased trustworthiness of transcriptomic structure compared with t-SNE. However, SpaSNE achieves a higher value of the product of the two scores than t-SNE (**Supplementary Fig. S15**). The metric of product of the two trustworthiness scores gives similar results as the silhouette score and it might be used as an alternative metric to replace silhouette score when the ground truth annotation is not available.*

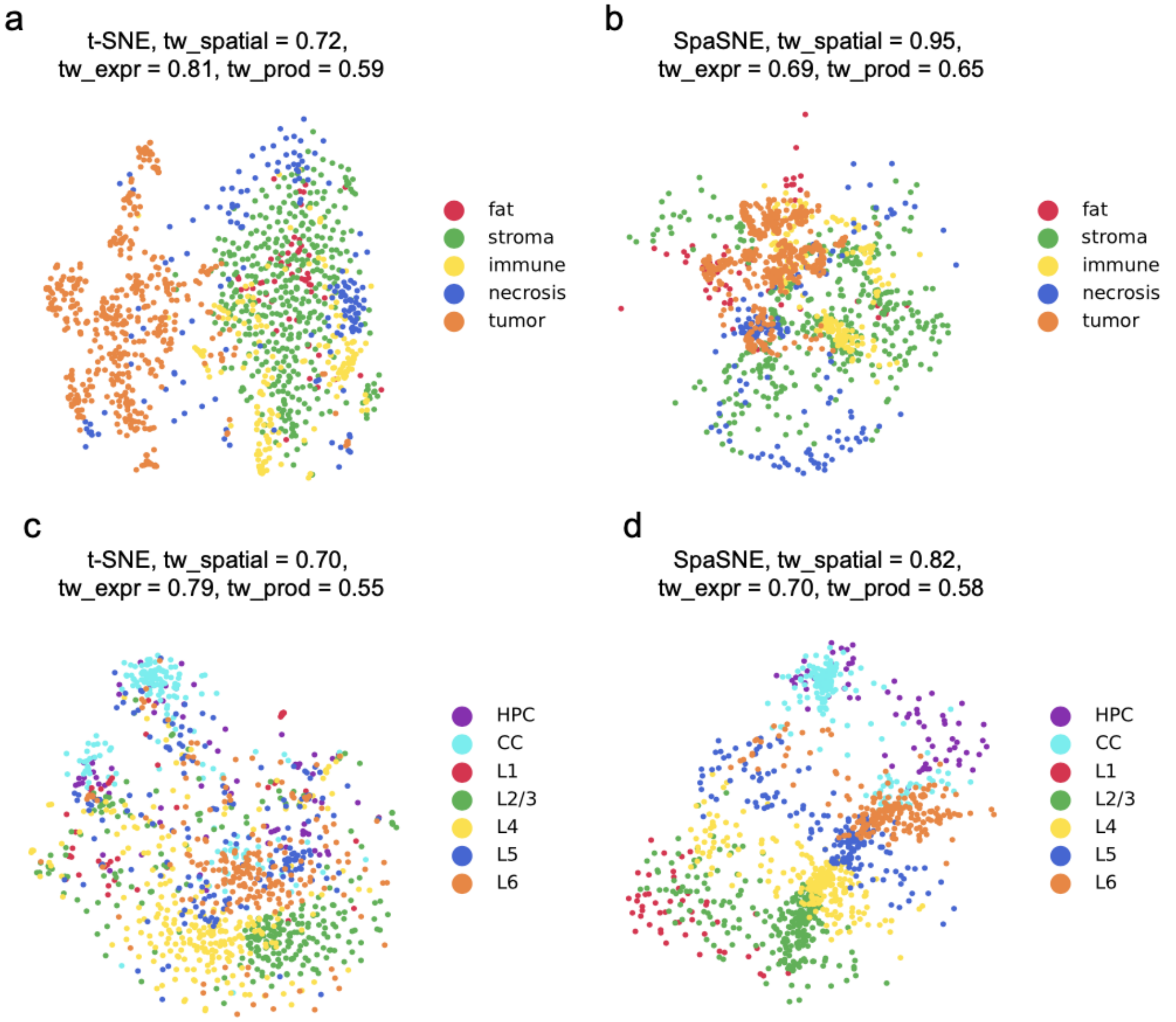

***Supplementary Figure S15. Embedding performances of SpaSNE using a metric of trustworthiness (tw). (a)** Evaluation of t-SNE embedding on human breast cancer dataset with trustworthiness score for spatial local structure (tw_spatial), trustworthiness score for transcriptomic local structure (tw_spatial), and the product of the two scores (tw_prod). (**b**) Evaluation of SpaSNE embedding with the three scores. (**c-d**) The same analyses on mouse visual cortex dataset.*

Lines 603-616 on page 28:

Fourthly, the design of SpaSNE assumes that the spatial information in spatially resolved transcriptomics data will contribute to the identification of cell states or the global organization of the cells. It may be less effective when there is no correlation between the phenotype and spatial positions of cells. In addition, its performance may be compromised in situations where spatial transcriptomics measurements are taken at multicellular resolution where a single spot

contains multiple cell types, or in subcellular resolution where a single cell covers multiple spatial positions such as Visium HD data. For the latter case, we may adapt SpaSNE to analyze such datasets by incorporating current decomposition or aggregation methods for the preprocessing of data in the future. For the four datasets, we found that SpaSNE achieved better embedding quality for the human breast cancer dataset and human prostate cancer dataset generated from the 10x Visium platform, than the mouse visual cortex dataset from the STARmap platform and mouse hypothalamus dataset from the MERFISH platform. The reason might be that 10x Visium platform measures a larger number of genes than STARmap and MERFISH and therefore helps better define cell types.

Comment 9: For clarity and completeness, it would be nice to define sigma_i in equation (1) for readers that are not very familiar with t-SNE.

Response: We have added a description of σ_i in line 143 on page 8.

Line 143 on page 8:

Where σ_i is the variance of the Gaussian distribution that centers on x_j.

Comment 10: In the breast cancer dataset, there is a class of spots phenotyped as "others". Based on available classes shown in the figure, does it correspond to "fat" in supplementary figure 1 ? If that is the case, why is it treated differently ? Are cells phenotyped as "others" used when computing the various metrics ?

Response: The “others” in Figure 2 (as well as in other figures) represent all the other cells besides the highlighted cell clusters. For example, the “others” in Figure 2e represent all other cells besides necrosis_1 and necrosis_2. This labeling strategy is only for visualization purposes; all spots, including those labeled as "others," are used in all metric calculations.

Comment 11: It would be nice to exhaustively display cell / spot phenotypes in supplementary data for each dataset for comprehensiveness. In figure 3, "others" appear to represent the majority of the spots but all spots are phenotyped in Figure S1b.

Response: We provided the annotations for the four datasets in **Supplementary Table S2-S5**. In Figure 3, the “others” represent all the other cell types phenotyped in **Supplementary Figure S1b**. The annotation of “others” in Figure 3 is used only for visualization purposes.

Comment 12: The authors mention that spatial proteomics technologies are "under development" but there are many commercial platform offering spatial protein expression information - albeit with a targeted design.

Response: We revised the sentences in the discussion part and added references for spatial epigenomics and proteomics technologies in lines 621-626 on page 28.

Lines 621-626 on page 28:

We currently focused on using available spatially resolved gene expression profiling data to demonstrate that SpaSNE can serve as a powerful dimensionality reduction and visualization tool for analyzing the spatially resolved profiling datasets with both molecular and spatial information. The design of SpaSNE allows it to analyze not only spatially resolved transcriptomics datasets, but also other types of datasets with similar data structures such as spatially resolved epigenomic and proteomic datasets.

<u>Reviewer #2 (Remarks to the Author)</u>
The overall concept of the paper is pretty interesting. I also managed to install and run the examples provided by the authors in their github on my MAC M1 Max without any issue, unless the need to install scanpy. However, how it is structurated the github it does not provide a sufficiently user-friendly tool.

Comment 1: The developers should offer a Docker container, accessible via JupyterLab, which is more user-friendly than Jupyter Notebook, in addition to the GitHub repository. This will mitigate the uncertainty of compiling the tool on different hardware and improve reproducibility. Additionally, SpaSNE should be accompanied by a suite of functions to simplify its usage and facilitate the testing of different parameters.

Response: Thank you for this helpful suggestion to improve the usability and reproducibility of SpaSNE. We developed the Docker container of SpaSNE and added the pip installation option on the GitHub page: https://github.com/Lin-Xu-lab/SpaSNE. We also provided tutorials for screening the optimal parameters and performing SpaSNE embeddings with different parameters on human breast cancer data on the GitHub page. See the texts in lines 643-646 on page 31.

Lines 643-646 on page 31:

The guidelines for installing the SpaSNE package, and the tutorials for screening optimal parameters of SpaSNE and performing SpaSNE embeddings with different parameters on human breast cancer data are available on the GitHub page (https://github.com/Lin-Xu-lab/SpaSNE.git). The SpaSNE software was adapted from the bhtsne scripts (github.com/lvdmaaten/bhtsne).

Comment 2: More information on the preparation and data format of metadata, such as _labels.csv, must be provided. Additionally, validation functions to test the compatibility of user-created metadata should be included.

Response: Thank you for this suggestion to enhance the clarity and usability of metadata preparation. We have provided annotations for the four datasets in Supplementary Table S2-S5. The metadata includes spatial positions and cell type labels for each cell/spot. The form of user-created metadata can be flexible, and it is usually manually processed by the users. The spatial positions in the metadata were fed into SpaSNE as a matrix or a Data Frame. The cell type labels were used in the form of a list to color the points in the visualization of SpaSNE embedding. We described the metadata in lines 117-123 on page 7.

Lines 117-123 on page 7:

The annotations of the human breast cancer, mouse visual cortex, and mouse hypothalamus datasets were obtained from original papers or websites (the details are included in the "Data availability" section). The human prostate cancer dataset was annotated by the HD-Staining algorithm developed for classifying cell nuclei and cell types in the pathology images. The annotations of the four datasets were provided in the metadata files in ***Supplementary Tables S2-***

S5. The metadata includes the spatial positions and different kinds of cell type labels for each cell/spot.

Comment 3: The authors noted that "the current SpaSNE package has not been optimized for handling datasets with a large number of cells." However, the limitations of the current implementation are unclear. Therefore, the performance of SpaSNE as the number of cells and the size of metadata increase should be evaluated and reported in the paper. Authors must consider that a tool effective on only a few hundreds of cells is practically useless given the current rate of increase in cell numbers in single-cell experiments.

Response: We discussed the computational cost of SpaSNE and the possible strategy to improve the speed of SpaSNE in lines 289-303 on page 15.

Thank you for raising this important point about SpaSNE's scalability. We have clarified SpaSNE's computational complexity and performance limitations in the revised manuscript. Currently, SpaSNE's computational cost comprises three main parts: the local loss of gene expression, the global loss of gene expression, and the global loss of spatial positions. While the local loss can be efficiently approximated using the vantage-point tree approximation, the two global losses require more computational resources, especially as the dataset size increases.

To improve scalability, we are exploring an approach similar to that in SpaceFlow, which uses a fixed number of randomly selected edges to approximate global loss calculations. This adaptation could reduce the computational load for large datasets. We have outlined this potential strategy in the revised manuscript at lines 289-303 on page 15.

Lines 289-303 on page 15:

The computational complexity of t-SNE (implemented by Barnes-Hut-SNE) is $O(NlogN)$. The computational complexity of UMAP is empirically $O(N^{1.14})$. The computational cost of SpaSNE consists of three parts: the local loss of gene expression L_t, global loss of gene expression L_g and global loss of spatial positions L_s (Eq. 11). By applying the vantage-point trees approximation used in Barnes-Hut-SNE, the cost of L_t can be reduced from $O(N^2)$ to $O(NlogN)$. However, the global loss L_g and L_s cannot be approximated by the local-structure-based strategy in Barnes-Hut-SNE or the Nearest-Neighbor-Descent algorithm used in UMAP. Thus, the computational cost in the current form of SpaSNE is $O(N^2)$. The running time of SpaSNE on a MacBook Pro with a two GHz Quad-Core Intel Core i5 processer and 16 GB 3733 MHz LPDDR4X memory varies from 18 seconds for the human breast cancer dataset with 1272 spots, to 3 minutes for the human prostate cancer dataset with 4371 spots. One possible approach to reducing computational time for large dataset is to mimic the strategy in the SpaceFlow algorithm to use a fixed number of randomly selected edges to approximate the pairwise distance calculation in global terms $\hat{p}_{ij}$, $\hat{q}_{ij}$ and $\hat{s}_{ij}$ (Eq. 6-9). In this way, the $O(N^2)$ in global loss will be constant and the total cost will become $O(NlogN)$.

Comment 4: Since the conclusions suggest that SpaSNE could serve as a solid foundation for various subsequent analytical procedures (such as trajectory analysis, and pseudotime analysis), examples of these applications should also be provided.

Response: Thank you for this suggestion to illustrate further applications of SpaSNE. We have added biological analyses of the differentially expressed genes based on the clusters separated or presented by SpaSNE and demonstrated that SpaSNE can produce low dimensional visualizations that better define cell states in two diseased tissue datasets and better preserve global layer structure in two developmental tissue datasets. We added nine new supplementary figures to demonstrate these (Fig.S2-S4, S7-S12). We described details of these new results in lines 346-363 on pages 17-18, lines 436-444 on page 21, lines 491-496 on page 23, and lines 509-515 on page 24, respectively, as follows.

Lines 346-363 on pages 17-18:

*The separation of cell clusters with different spatial locations is important because the cell states are influenced by their neighboring cells. For example, cells in tumor_2 and tumor_5, which are surrounded by immune cells and stroma cells respectively (**Supplementary Fig. S2a**), have distinct expression patterns of marker genes such as IFI27, LGALS3BP, and B2M (**Supplementary Fig. S2b-c**). The genes that are highly expressed in tumor_2 (surrounded by immune cells) are involved in biological processes related to immune responses with high enrichment scores in Gene Ontology analysis(**Supplementary Fig. S3a-c, -log10(p) >9**), while the genes that are highly expressed in tumor_5 (surrounded by stroma cells) are involved in translation activities with low enrichment scores(**Supplementary Fig. S3d-f, -log10(p)** < 7). Similarly, cells in "immune_2" and "immune_1", which are surrounded by tumor and stroma respectively (**Supplementary Fig. S2a**), have distinct expression patterns of marker genes such as ISG15, IFI6, and IFI27 (**Supplementary Fig. S2b-c**). The highly expressed genes in immune_2 are involved in immune responses while the highly expressed genes in immune_1 are involved in other biological processes (**Supplementary Fig. S4a-f**). These results showed that SpaSNE could produce a more delicate visualization that distinguishes different cell states of the same cell type that interact with different spatial environments by leveraging both gene expression and spatial information.*

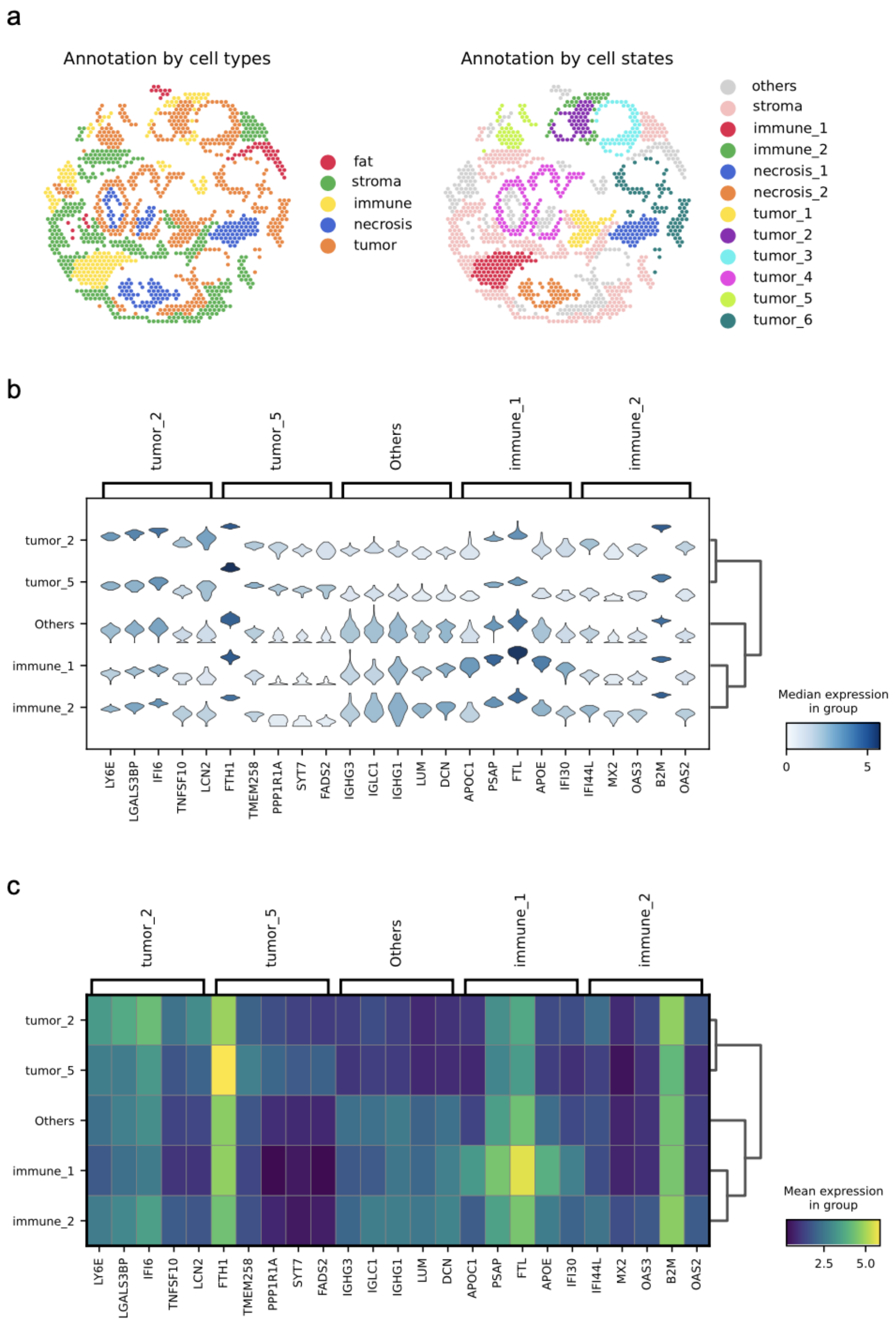

Supplementary Figure S2. Differential gene expressions of selected cell clusters in human breast cancer tissue. *(a) Annotation of human breast cancer cell clusters according to cell types (left) and cell states (right). The cell states annotation is the same as in Fig.2a-c in the manuscript. (b) Violin plots of top differentially expressed genes in tumor_2, tumor_5, immune_1, immune_2, and the rest cell types. (c) Mean expression values of top differentially expressed genes in the selected cell types.*

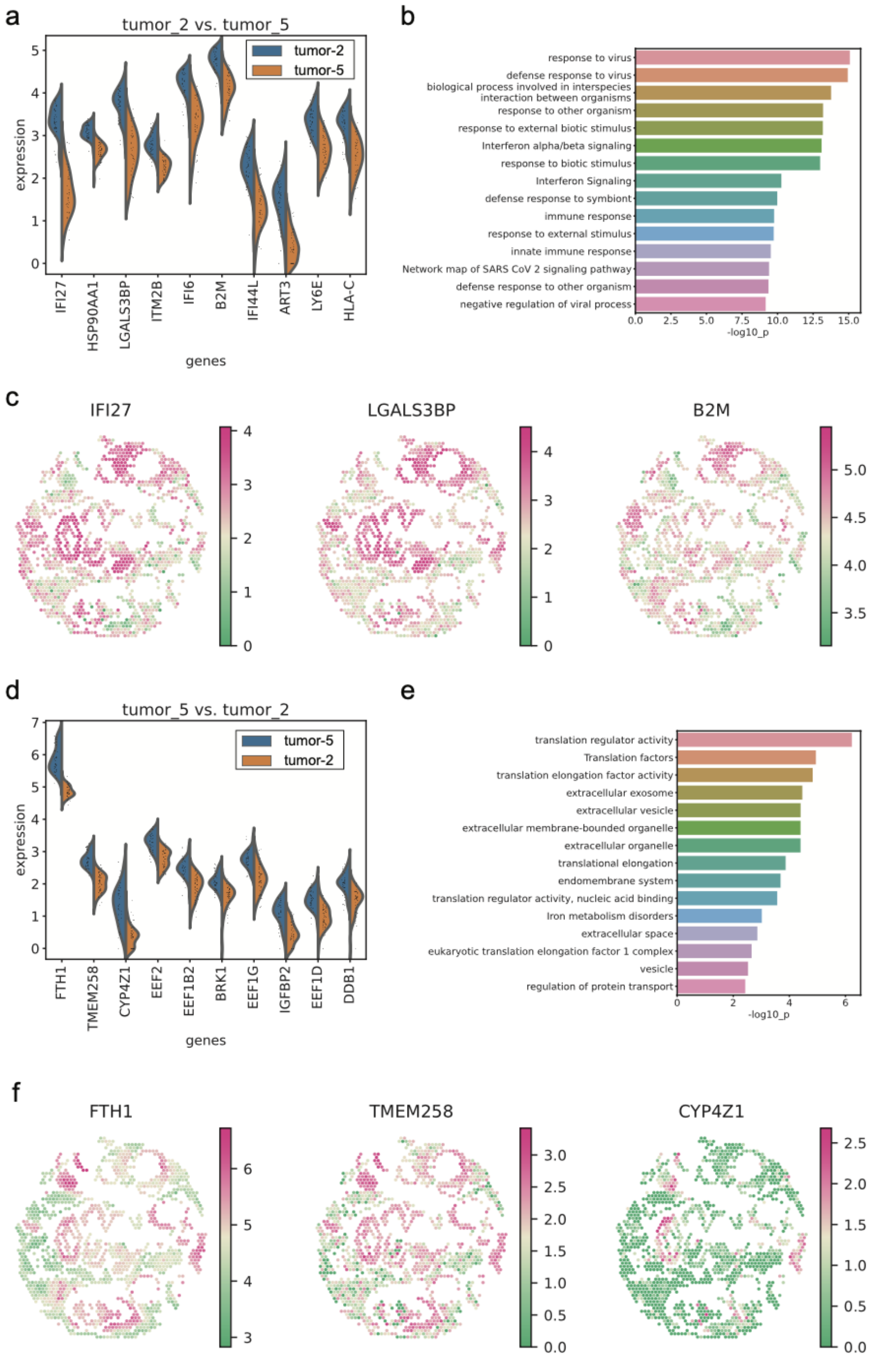

Supplementary Figure S3. Comparison of two tumor subtypes. *(a-c) Differentially expressed genes analysis with tumor_5 as reference. (a) Violin plots of the top differentially expressed genes. (b) The top 15 Gene Ontology Biological Processes obtained from the top 30 differentially expressed genes. (c) Expressions of three differentially expressed genes IFI27, LGALS3BP, and B2M. (d-f) Differentially expressed genes analysis with tumor_2 as reference. (d) Violin plots of the top differentially expressed genes. (e) The top 15 Gene Ontology Biological Processes obtained from the top 30 differentially expressed genes. (f) Expressions of three differentially expressed genes FTH1, TMEM258, and CYP4Z1.*

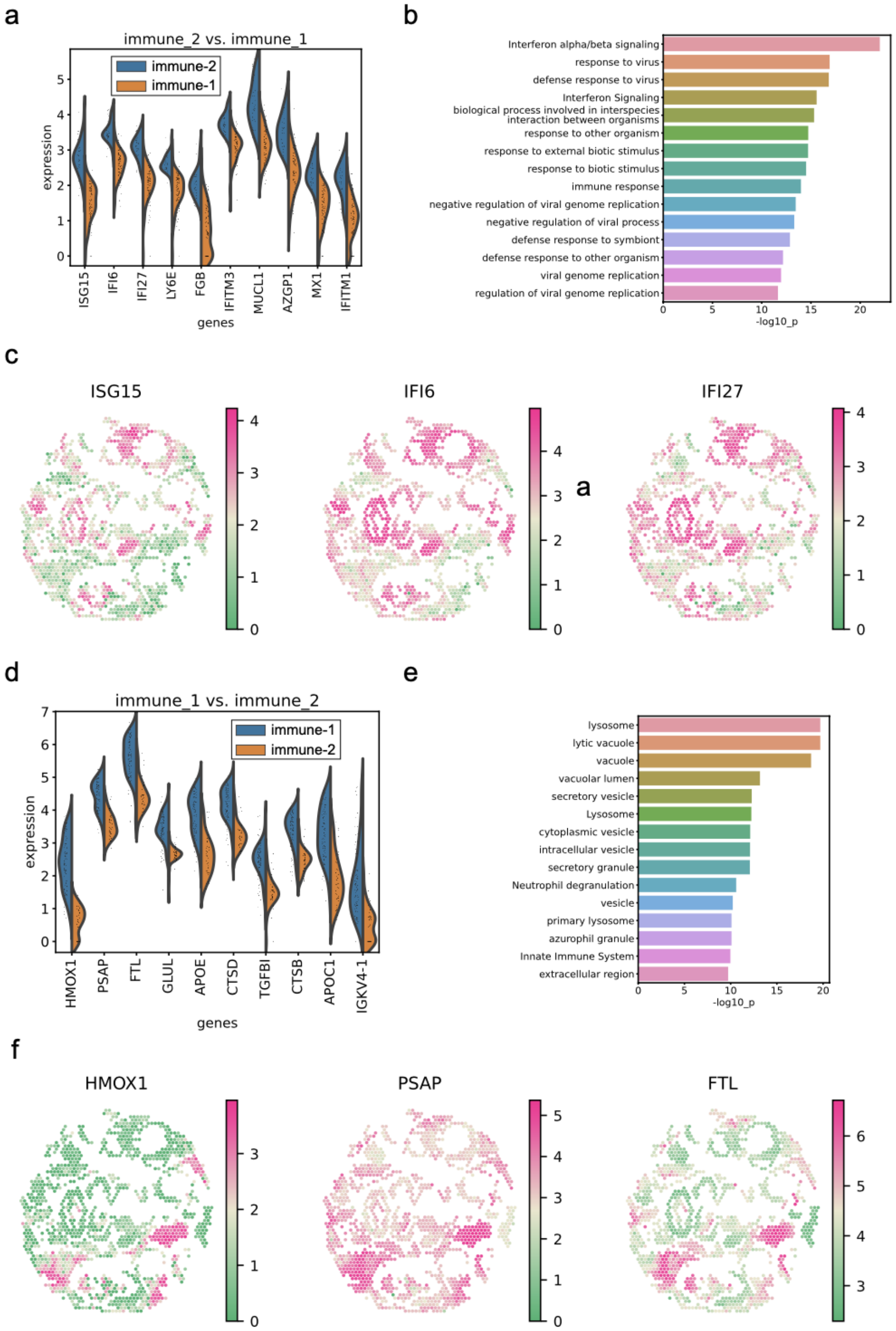

Supplementary Figure S4. Comparison of two immune subtypes. *(a-c) Differentially expressed genes analysis with immune_1 as reference. (a) Violin plots of the top differentially expressed genes. (b) The top 15 Gene Ontology Biological Processes obtained from the top 30 differentially expressed genes. (c) Expressions of three differentially expressed genes ISG15, IFI6, and IFI27. (d-f) Differentially expressed genes analysis with immune_2 as reference. (d) Violin plots of the top differentially expressed genes. (e) The top 15 Gene Ontology Biological Processes obtained from the top 30 differentially expressed genes. (f) Expressions of three differentially expressed genes HMOX1, PSAP, and FTL.*

The following text that describes Fig. S2, S3, and S4 were added to the revised manuscript at lines 436-444 on page 21:

*The more delicate cell clusters separated by SpaSNE represent different cell states with different spatial environments. For example, cells in immune_2 and immune_1 are surrounded by tumor cells and stroma cells respectively, and have distinct expression patterns of marker genes such as CNN1, DES, and TMEFF2 (**Supplementary Fig. S7a-c**). The genes highly expressed in immune_2 cells are involved in biological processes including smooth muscle and smooth muscle cells, which play important roles in prostate cancer, with high enrichment scores (-log10(p) > 10) (**Supplementary Fig. S8a-c**). The genes highly expressed in immune_1 cells are involved in vesicle-related biological processes with low enrichment scores (-log10(p) < 4) (**Supplementary Fig. S8d-f**).*

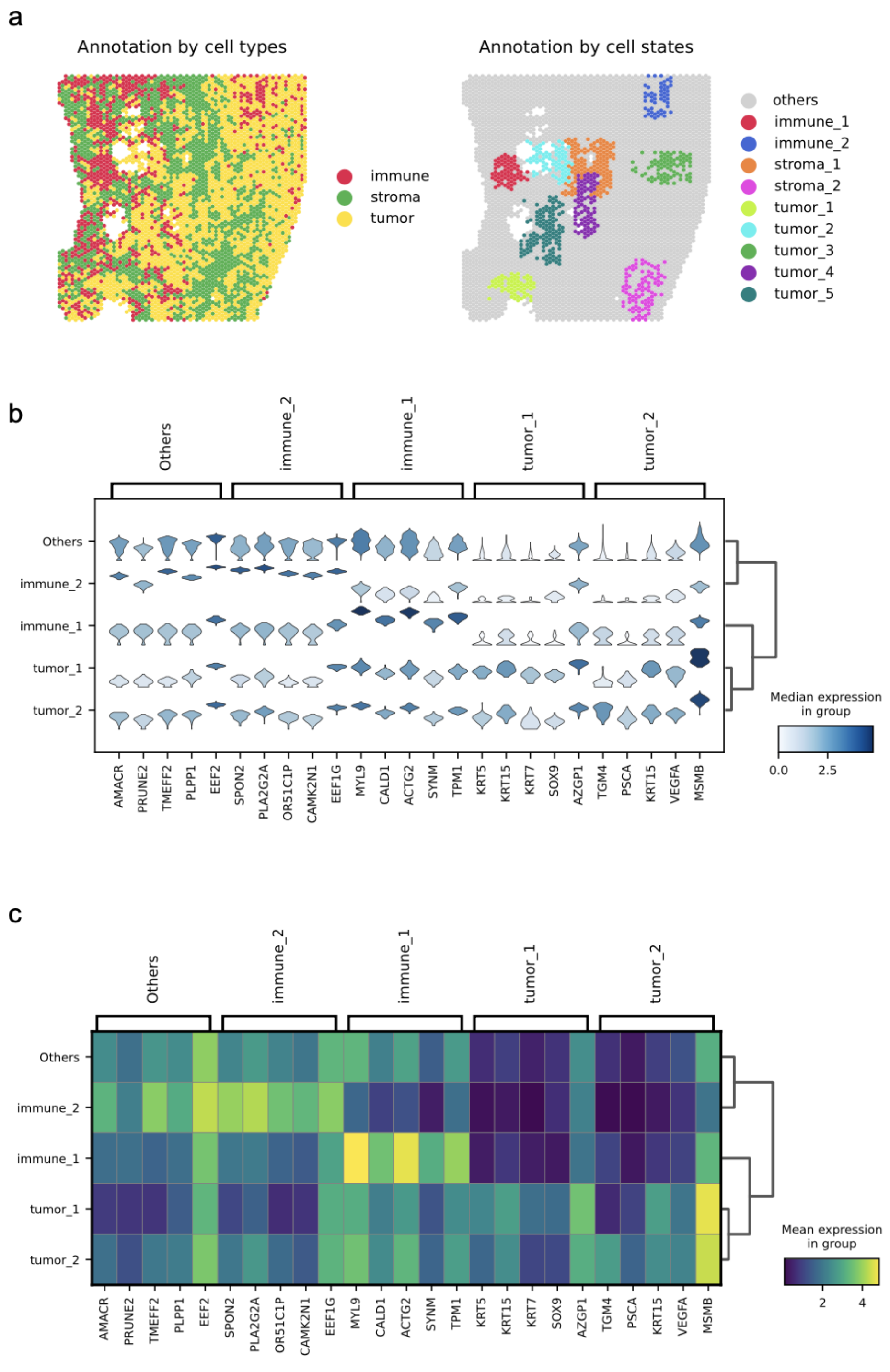

Supplementary Figure S7. Differential gene expressions of selected cell clusters in human prostate cancer tissue. *(a) Annotation of human prostate cancer cell clusters according to cell types (left) and cell states (right). This annotation is the same as in Fig.3a-c in the manuscript. (b) Violin plots of top differentially expressed genes in tumor_1, tumor_2, immune_1, immune_2, and the rest cell types. (c) Mean expression values of top differentially expressed genes in the selected cell types.*

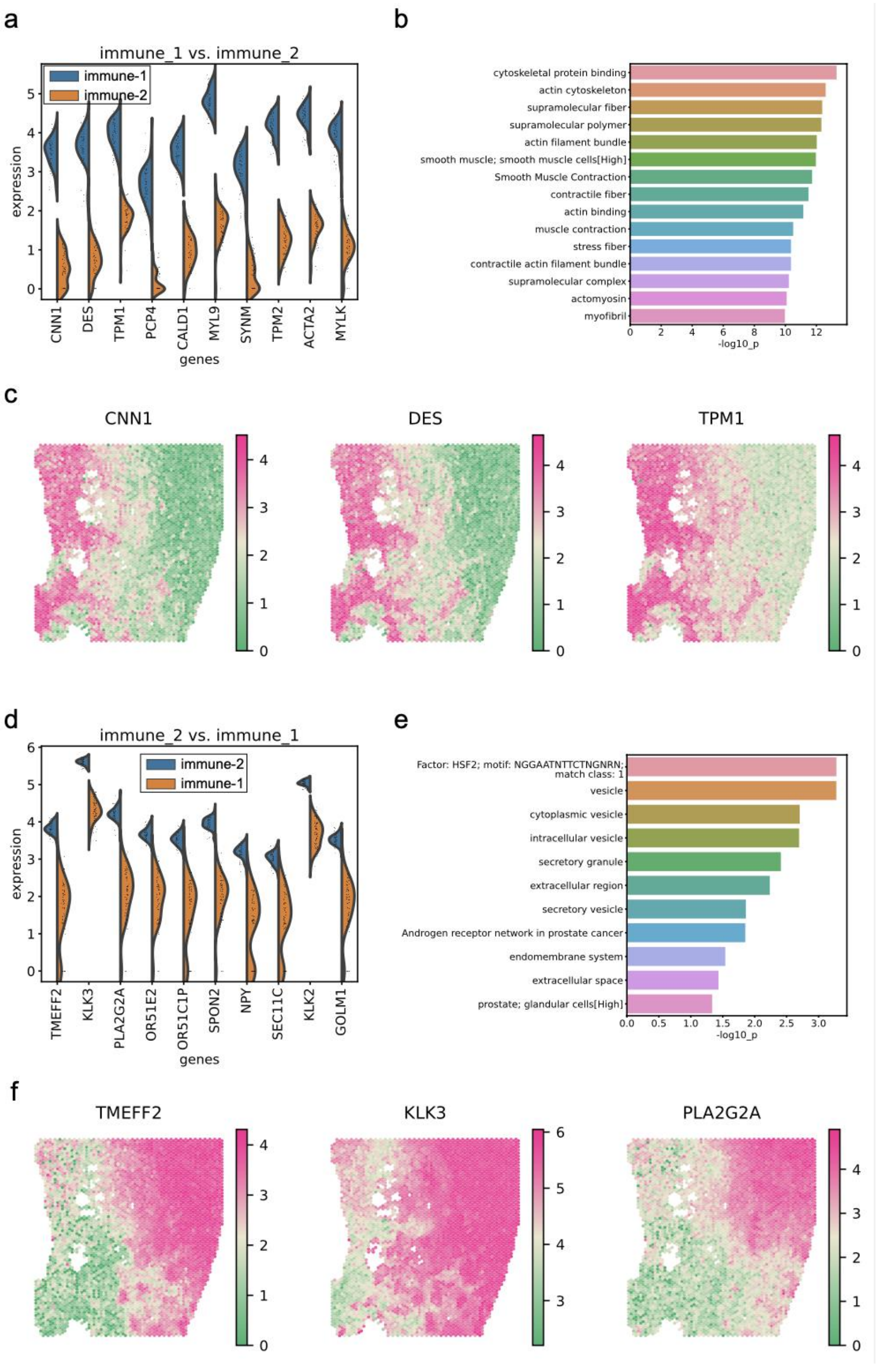

Supplementary Figure S8. Comparison of two immune subtypes. *(a-c) Differentially expressed genes analysis with immune_2 as reference. (a) Violin plots of the top differentially expressed genes. (b) The top 15 Gene Ontology Biological Processes obtained from the top 30 differentially expressed genes. (c) Expressions of three differentially expressed genes CNN1, DES, and TPM1. (d-f) Differentially expressed genes analysis with immune_1 as reference. (d) Violin plots of the top differentially expressed genes. (e) The top 15 Gene Ontology Biological Processes obtained from the top 30 differentially expressed genes. (f) Expressions of three differentially expressed genes TMEFF2, KLK3, and PLA2G2A.*

The following text that describes Fig. S9 and S10 were added to the revised manuscript at lines 491-496 on page 23.

Lines 491-496 on page 23:

*Here, we performed differential expression analysis based on the six layers and found that the differentially expressed genes are involved in biological processes including system development and neurogenesis (**Supplementary Fig. S9a-c**). By comparing each layer with the rest layers, we identified layer-specific markers genes and selected five of them for visualization: FOSB(L1), CAMK2N1(L2/3), CPLX1(L5), PCP1(L6) and MBP(CC) (**Supplementary Fig. S10**).*

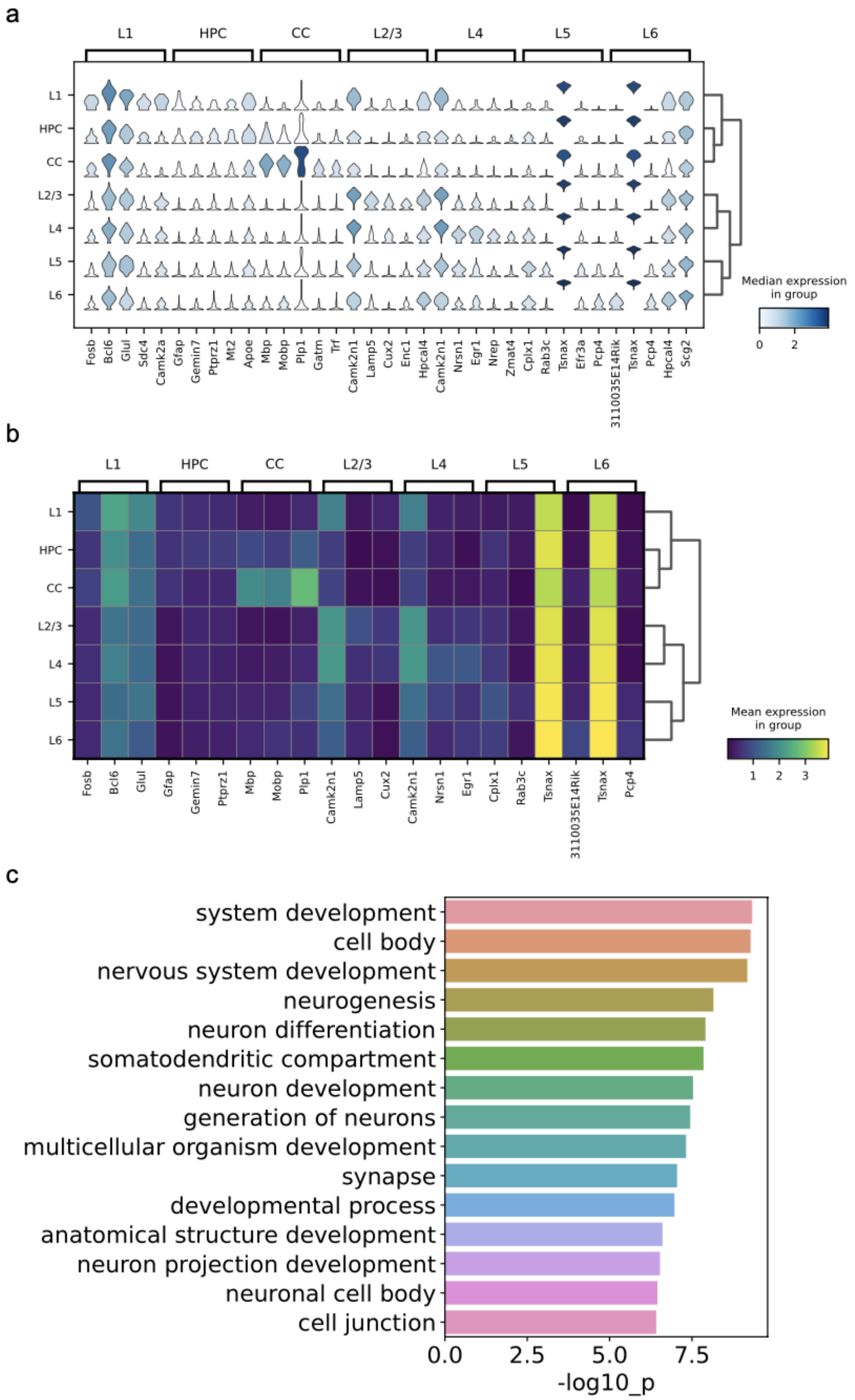

Supplementary Figure S9. Differential gene expressions of seven layers in mouse visual cortex tissue. *(a) Violin plots of top differentially expressed genes in the seven layers. (b) Mean expression values of top differentially expressed genes in the seven layers. (c) The top 15 Gene Ontology Biological Processes obtained from 70 differentially expressed genes.*

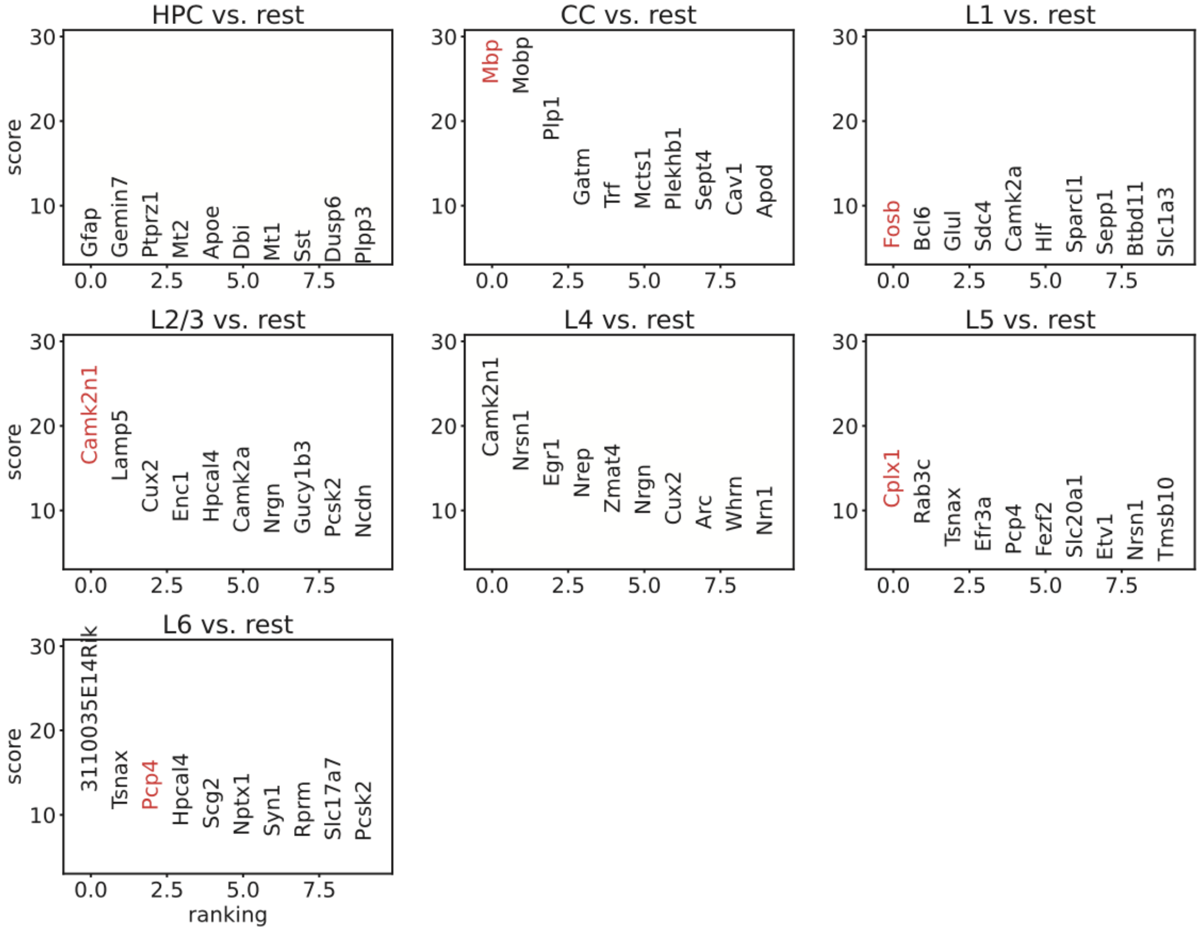

Supplementary Figure S10. Differentially expressed genes from the comparison between each layer type and the rest layers in the mouse visual cortex dataset. *The genes marked red are the genes shown in Fig. 4e-g in the manuscript.*

The following text that describes Fig. S9-S12 were added to the revised manuscript at lines 509-515 on page 24.

Lines 509-515 on page 24:

*Following the analysis in **Supplementary Fig. S9-S10**, we performed differential expression analysis based on the eleven nucleus types and found that the differentially expressed genes are involved in biological processes including multicellular organismal process and nervous system development (**Supplementary Fig. S11a-c**). By comparing each layer with the rest layers, we identified layer-specific markers genes and selected five of them for visualization: MBP(ACA), IRS4(BNST), HTR2C (AVPe), SOX6(MPA), and GDA(VLPO) (**Supplementary Fig. S12**).*

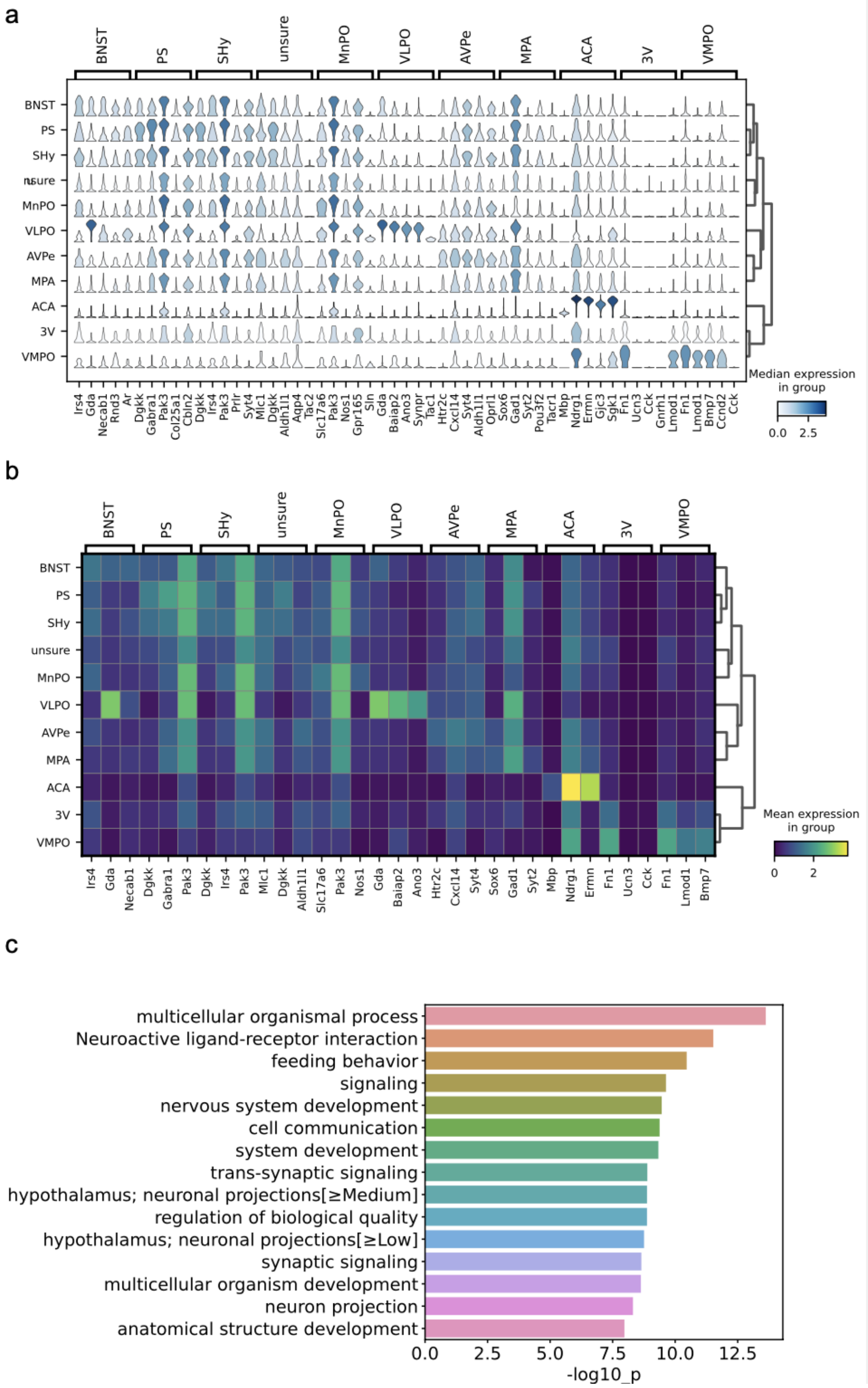

Supplementary Figure S11. Differential gene expressions of seven layers in mouse hypothalamus tissue. *(a) Violin plots of top differentially expressed genes in the seven layers. (b) Mean expression values of top differentially expressed genes in the seven layers. (c) The top 15 Gene Ontology Biological Processes obtained from 90 differentially expressed genes.*

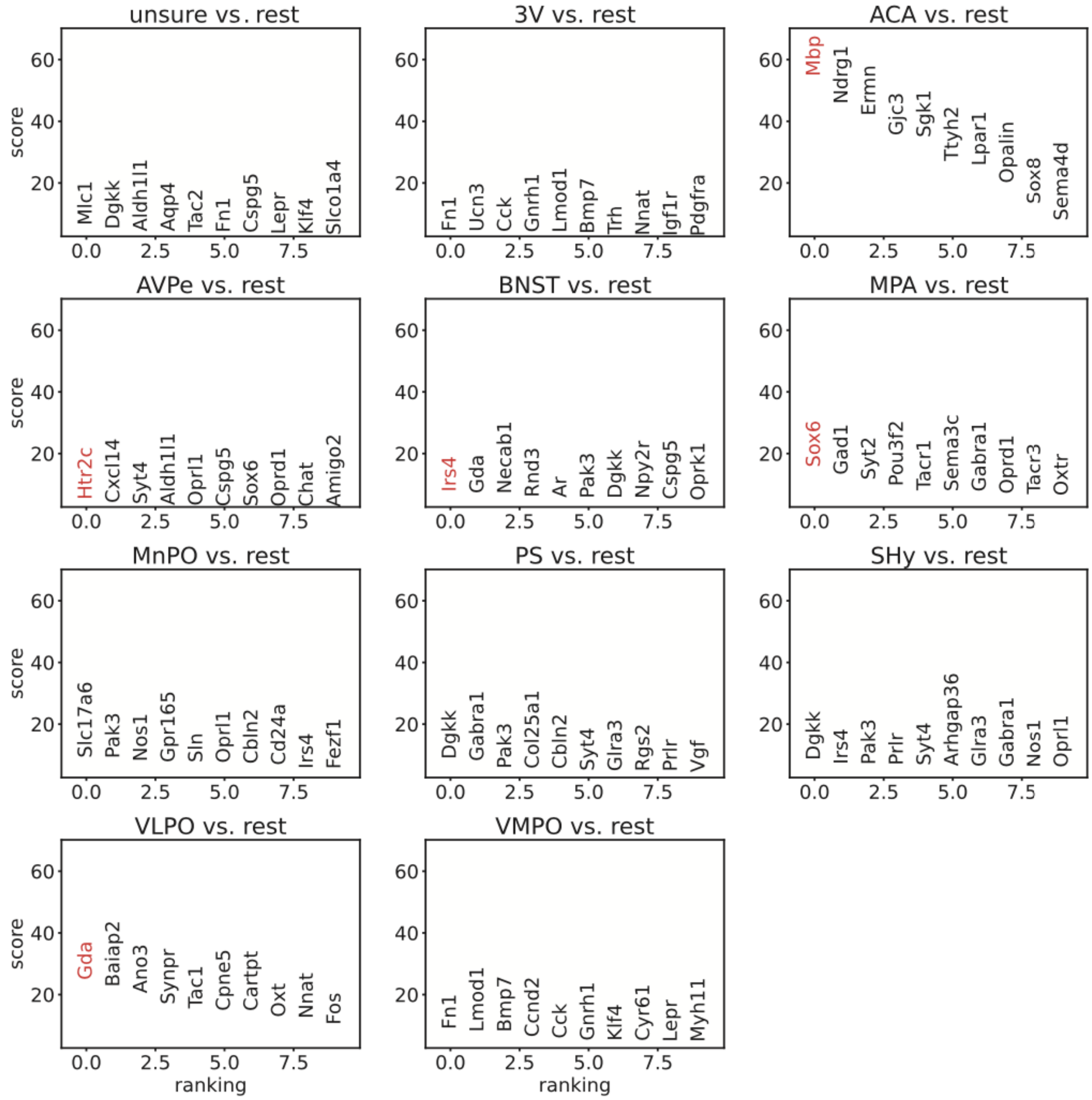

Supplementary Figure S12. Differentially expressed genes from comparison between each layer type and the rest layers in mouse hypothalamus data. *The genes marked red are the genes shown in Fig. 5e-g in the manuscript.*

Comment 5: The authors stated that "SpaSNE offers tunable parameters to adjust the users' requests on the preservation of molecular or spatial information." Therefore, a detailed section on GitHub should be dedicated to thoroughly describing the effects of these tunable parameters.

Response: Thank you for this suggestion. We have updated our GitHub repository to include a tutorial on parameter tuning, describing how the selection of parameters changes the embedding results (See spasne_BreastCancer1272_embeddings.ipynb in https://github.com/Lin-Xu-lab/SpaSNE)

Comment 6: At lane 194 it is stated " we screened the optimal combination of parameters alpha and beta that gave the best performances in terms of the gene expression preservation ..." more details must be provided on what the author mean by "we screened the optimal combination of parameters ..."

Response: We have revised the manuscript to provide a step-by-step explanation of our two-stage heuristic screening method, which includes an initial rough screening to identify a general parameter range, followed by fine-tuning within that range. We also added Supplementary Figure S16, which illustrates the screening results across four datasets. A tutorial detailing this screening process is also available on GitHub (https://github.com/Lin-Xu-lab/SpaSNE)

Lines 229-258 on pages 12-13:

*To determine the optimal combination of parameters for a given data, we developed a heuristic screening approach that consists of two stages: rough screening and fine screening. In rough screening, we screened the two parameters on a larger scale to determine the range where the optimal parameters may fall (**Supplementary Fig. S16a, c, e, g**). In fine screening, we determined the optimal parameter with a finer resolution (**Supplementary Fig. S16b, d, f, h**). Here we use the example of the human breast cancer dataset to demonstrate the two-stage screening process in detail:*

1. *Running 100 repeats of t-SNE with default parameters on human breast cancer datasets and calculating (r_g, r_s) for each repeat. The maximal value of r_g is marked as r_{thres}.*
2. *Performing rough screening with SpaSNE.*
   - 2.1. *Taking α and β from $\{(\alpha, \beta)|\ \alpha \in [2, 5, 10, 20, 30, 50], \beta \in [1, 5, 10, 15, 25]\}$.*
   - 2.2. *In each parameter combination, running 10 repeats of SpaSNE. Setting $r_g = 0$ if $r_g \leq r_{thres}$ in each repeat. Calculating (r_g, r_s) for each repeat. Selecting the optimal embedding that gives the maximal value of $r_g \times r_s$ in the 10 repeats and recording the optimal r_g^{opt} and r_s^{opt}.*
   - 2.3. *Showing the values of $r_g^{opt} \times r_s^{opt}$ for all the parameter combinations by heatmap (**Supplementary Fig. S16a**).*
3. *Performing fine screening with SpaSNE.*
   - 3.1. *Based on the heatmap results in step 2.3, selecting the range where the optimal parameters may fall: $\{(\alpha, \beta)|\ \alpha \in [5, 6, 7, .., 20],\ \beta \in [1, 2, 3, \dots, 10]\}$.*

3.2. *In each parameter combination, running 20 repeats of SpaSNE. Setting* $r_g = 0$ *if* $r_g \leq r_{thres}$ *in each repeat. Calculating* (r_g, r_s) *for each repeat and standard deviation* (std) *of* r_g *of the 20 repeats. Selecting the optimal embedding that gives maximal value of* $r_g \times r_s$ *in the 20 repeats and recording the optimal values* r_g^{opt}, r_s^{opt} *and* std *.*

3.3. *Showing the values of* $r_g^{opt} \times r_s^{opt}$ *(left),* std *(middle) and* $r_g^{opt} \times r_s^{opt} \times exp\,(1 - std)$ *(right) for all the parameter combinations by heatmap (**Supplementary Fig. S16b**).*

4. *Determining the optimal parameter combination by selecting the maximal value of* $r_g^{opt} \times r_s^{opt} \times exp\,(1 - std)$ *obtained in 3.3.*
5. *Running 100 repeats of SpaSNE with the optimal parameter combination obtained in step 4 and selecting the embedding with the maximal values of* $r_g \times r_s$*.*

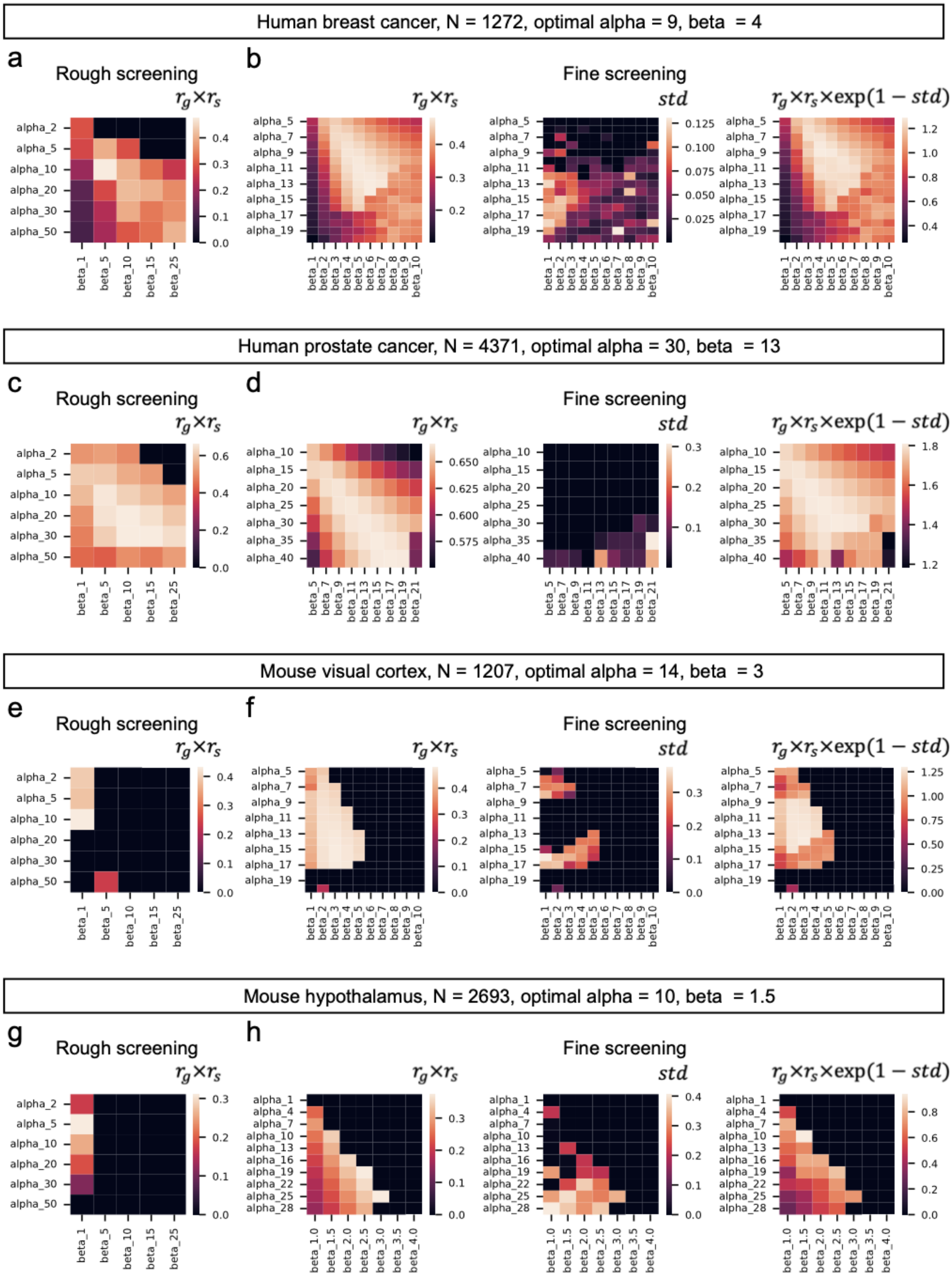
Human breast cancer, N = 1272, optimal alpha = 9, beta = 4
a
Rough screening
$r_g \times r_s$
b
Fine screening
$r_g \times r_s$
std
$r_g \times r_s \times \exp(1 - std)$
Human prostate cancer, N = 4371, optimal alpha = 30, beta = 13
c
Rough screening
$r_g \times r_s$
d
Fine screening
$r_g \times r_s$
std
$r_g \times r_s \times \exp(1 - std)$
Mouse visual cortex, N = 1207, optimal alpha = 14, beta = 3
e
Rough screening
$r_g \times r_s$
f
Fine screening
$r_g \times r_s$
std
$r_g \times r_s \times \exp(1 - std)$
Mouse hypothalamus, N = 2693, optimal alpha = 10, beta = 1.5
g
Rough screening
$r_g \times r_s$
h
Fine screening
$r_g \times r_s$
std
$r_g \times r_s \times \exp(1 - std)$

Supplementary Figure S16. Rough and fine screenings of parameters α and β of SpaSNE on the four datasets.

*For each dataset, a rough screening was first performed to determine the range of optimal parameters on a large scale (a, c, e, g), then a fine screening was performed to find the optimal parameters with a fine resolution (b, d, f, h). In each embedding, setting $r_g = 0$ if $r_g \leq r_{thres}$, where r_{thres} is the maximal r_g in 100 repeats of t-SNE embeddings. The heatmaps for rough tuning represent values of $r_g \times r_s$ with different combinations of parameters. The heatmaps for fine tuning represent values of $r_g \times r_s$ (left), std (middle) and $r_g \times r_s \times exp\,(1 - std)$(right). The std is the standard deviation of r_g in multiple repeats of SpaSNE embeddings in each parameter combination. The parameter ranges used in screening and optimal parameters for the four datasets can be found in **Supplementary Table S1**. (a-b) Rough (a) and fine (b) screening on human breast cancer data. (c-d) Rough (c) and fine (d) screening on human prostate cancer data. (e-f) Rough (e) and fine (f) screening on mouse visual cortex data. (g-h) Rough (g) and fine (h) screening on mouse hypothalamus data.*

Comment 7: In the section "Quantitative Evaluation of the Embedding Quality," Pearson correlation is used. The authors must justify why the Pearson correlation coefficient is the best method for measuring embedding quality.

Response: We selected the Pearson correlation coefficient as a straightforward measure of linear correspondence between high-dimensional distances and low-dimensional embedding distances, which has been widely used in visualization evaluation, including for UMAP. This correlation reflects how well spatial or gene expression distances are preserved in the embeddings. We discussed this in lines 191-194 on page 11.

Lines 191-194 on page 11:

Three quantitative measures were defined to evaluate the embedding quality: (1) Pearson correlation coefficient (r_g) between the pairwise Euclidean distances of the gene expressions and the embedding distances of points, this metric is equivalent to the Shephard diagram which was usually used to measure the goodness of fit by low dimensional visualization algorithms.

Comment 8: As far as I remember, perplexity is a critical parameter in t-SNE. Could the authors provide additional information about the role of perplexity in SpaSNE?

Response: Thank you for this suggestion. We set the perplexity value to its default of 50 in both t-SNE and SpaSNE. In SpaSNE, increasing perplexity can enhance global structure preservation, but its effect is reduced compared to t-SNE, as SpaSNE also depends on α and β for preserving spatial and gene expression structures. We added **Supplementary Figure S17** to illustrate the effects of varying perplexity values across datasets. This discussion has been added to the manuscript at lines 278-286 on pages 14-15.

Lines 278-286 on pages 14-15:

*The perplexity values in SpaSNE and t-SNE were set as the default value which is 50. Increasing perplexity will improve the global structure preservation (r_g) in SpaSNE, but this parameter does not influence the embedding of SpaSNE as much as that of t-SNE (**Supplementary Fig. S17**). The reason is that perplexity determines the number of neighbors in local structure preservation, while SpaSNE embedding largely depends on the two added parameters α and β which preserves the global gene expression structure and spatial structure. The perplexity's influence becomes weaker as α and β grow larger (comparing human breast cancer dataset with $\alpha = 9$ to human prostate cancer dataset with $\alpha = 30$) (**Supplementary Fig. S17a, b**).*

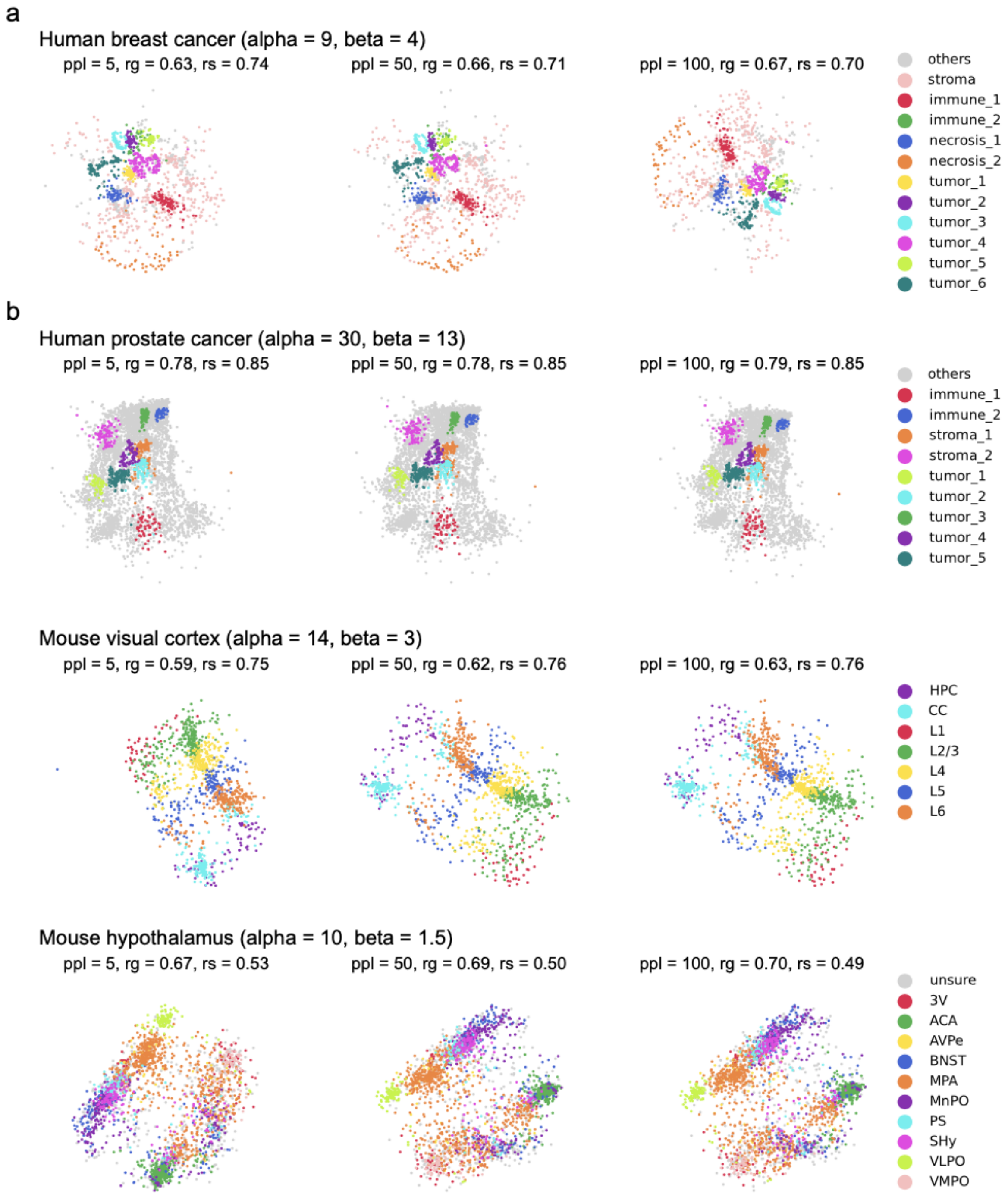

Supplementary Figure S17. SpaSNE embeddings on the four datasets with different perplexity values (ppl). *Three perplexity values were applied on each dataset: ppl = 5, 50, 100. The default value in SpaSNE is 50. (a-d) Results on human breast cancer dataset (a), human prostate cancer dataset (b), mouse visual cortex dataset (c), and mouse hypothalamus dataset (d).*

<u>Reviewer #3 (Remarks to the Author)</u>

This manuscript introduces SpaSNE (Spatially resolved t-SNE), a dimensionality reduction and visualization method designed specifically for spatially resolved profiling data. The authors adapt the t-SNE algorithm to incorporate both gene expression and spatial information, addressing a gap in current analysis tools for spatial omics data. They demonstrate SpaSNE's performance on four datasets from three different experimental platforms (Visium, STARmap, and MERFISH), covering both diseased and normal tissues. The results show that SpaSNE outperforms methods like t-SNE and UMAP in preserving both molecular and spatial data structures, leading to more accurate and meaningful visualizations.

While the study addresses an important need in the rapidly growing field of spatial omics, there are several areas that require further development and clarification. The authors provide a comparative evaluation of SpaSNE's performance against existing methods, but the parameter selection process lacks comprehensive guidance, and the scalability of the method for larger datasets is not adequately addressed. Additionally, the comparison with other spatial-aware methods is limited, and the robustness of SpaSNE across various experimental conditions needs further discussion.

Overall, this work represents a potential contribution to the field of spatial omics data analysis. However, several key issues need to be addressed to fully demonstrate the method's utility and reliability. These include providing clearer guidelines for parameter selection, addressing scalability concerns, expanding comparisons with other spatial-aware methods, and thoroughly discussing the method's robustness. Addressing these points would strengthen the manuscript and better establish SpaSNE's potential as a valuable tool for researchers working with spatially resolved profiling data.

Comment 1: Parameter selection
The authors mention that SpaSNE has tunable parameters (α and β) to adjust the balance between molecular and spatial information preservation. They provide a recommended range for setting α and β based on their experience with four datasets. However, this approach has limitations:
1. The trade-off between L_t,L_g, and L_s should consider the scales of gene expression and spatial location data, which may vary across different datasets and experimental platforms.
2. Even if the recommended range may be suitable for many spatially resolved profiling datasets, the authors do not provide a heuristic strategy for selecting optimal hyperparameters within this range.
3. There is no discussion of how these parameters might need to be adjusted for datasets with significantly different characteristics or scales.
It would be highly beneficial to provide more comprehensive guidance on how users should select these parameters for their specific datasets. This could include:
1. A more detailed explanation of how the scales of gene expression and spatial data might influence parameter selection.
2. A step-by-step heuristic approach for parameter tuning based on dataset characteristics.

3. Discussion of any rules of thumb that users could apply.
4. Exploration of potential automated methods for parameter optimization, such as grid search with cross-validation or Bayesian optimization.

Additionally, the authors should consider providing a small benchmark dataset and a tutorial demonstrating the parameter selection process. This would greatly enhance the usability of SpaSNE for researchers new to the method.

Response: Thanks for these helpful suggestions. We have thoroughly revised the parameter selection process by developing a two-stage screening strategy that includes rough screening to identify a general parameter range, followed by fine screening within this range. This two-stage approach enables us to determine optimal parameters across datasets with different sizes and data characteristics. In addition, we provide guidance on how dataset characteristics, such as size and data type, influence parameter selection. A step-by-step tutorial on parameter selection for the human breast cancer dataset has been added to our GitHub page (https://github.com/Lin-Xu-lab/SpaSNE), along with benchmark data and examples for new users. These additions are discussed in the revised manuscript at lines 217-273 on pages 12-14 and in **Supplementary Figure S16.**

Lines 217-273 on pages 12-14:

For SpaSNE, we screened the combination of parameters α and β on the four datasets and showed how the parameters influence gene expression preservation (r_1), spatial structure preservation (r_2), and the clustering quality (s), as well as the stability of the embeddings. The two parameters α and β in Eq. 11 represent the weights of the large-scale gene expression' loss function L_g and the spatial loss function L_s. Therefore, a larger α leads to a larger r_1 and a smaller r_2, a larger β leads to a larger r_2 and a smaller r_1. Both parameters collectively affect the clustering quality s. In addition to the ratio of α and β, the magnitude of α may also influence the stability of the embedding because the contribution of the local cost function L_t in the original t-SNE will be weakened by a large α (Eq. 11) and the embedding will become more unstable, especially when the dataset size is small.

*To determine the optimal combination of parameters for a given data, we developed a heuristic screening approach that consists of two stages: rough screening and fine screening. In rough screening, we screened the two parameters on a larger scale to determine the range where the optimal parameters may fall (**Supplementary Fig. S16a, c, e, g**). In fine screening, we determined the optimal parameter with a finer resolution (**Supplementary Fig. S16b, d, f, h**). Here we use the example of the human breast cancer dataset to demonstrate the two-stage screening process in detail:*

1. *Running 100 repeats of t-SNE with default parameters on human breast cancer datasets and calculating (r_g, r_s) for each repeat. The maximal value of r_g is marked as r_{thres}.*
2. *Performing rough screening with SpaSNE.*
   2.1. *Taking α and β from $\{(\alpha, \beta) | \alpha \in [2, 5, 10, 20, 30, 50], \beta \in [1, 5, 10, 15, 25]\}$.*

2.2. *In each parameter combination, running 10 repeats of SpaSNE. Setting* $r_g = 0$ *if* $r_g \leq r_{thres}$ *in each repeat. Calculating* (r_g, r_s) *for each repeat. Selecting the optimal embedding that gives maximal value of* $r_g \times r_s$ *in the 10 repeats and recording the optimal* r_g^{opt} *and* r_s^{opt}.

2.3. *Showing the values of* $r_g^{opt} \times r_s^{opt}$ *for all the parameter combinations by heatmap (**Supplementary Fig. S16a**).*

3. *Performing fine screening with SpaSNE.*

3.1. *Based on the heatmap results in step 2.3, selecting the range where the optimal parameters may fall:* $\{(\alpha, \beta)|\ \alpha \in [5, 6, 7, .., 20],\ \beta \in [1, 2, 3, \ldots, 10]\}$.

3.2. *In each parameter combination, running 20 repeats of SpaSNE. Setting* $r_g = 0$ *if* $r_g \leq r_{thres}$ *in each repeat. Calculating* (r_g, r_s) *for each repeat and standard deviation* (std) *of* r_g *of the 20 repeats. Selecting the optimal embedding that gives the maximal value of* $r_g \times r_s$ *in the 20 repeats and recording the optimal values* r_g^{opt}, r_s^{opt} *and* std .

3.3. *Showing the values of* $r_g^{opt} \times r_s^{opt}$ *(left),* std *(middle) and* $r_g^{opt} \times r_s^{opt} \times exp\,(1 - std)$ *(right) for all the parameter combinations by heatmap (**Supplementary Fig. S16b**).*

4. *Determining the optimal parameter combination by selecting the maximal value of* $r_g^{opt} \times r_s^{opt} \times exp\,(1 - std)$ *obtained in 3.3.*

5. *Running 100 repeats of SpaSNE with the optimal parameter combination obtained in step 4 and selecting the embedding with the maximal values of* $r_g \times r_s$.

*The results on the four datasets show that the optimal parameters depend on both the size and the type of the data. For example, both the human breast cancer and prostate cancer datasets are generated from the 10X Visium platform, the optimal parameters are larger for the dataset with a larger size (comparing the human prostate cancer dataset with N = 4371, α = 30, to the human breast cancer dataset with N = 1272, α = 9) (**Supplementary Fig. S16b,d**). However, the mouse hypothalamus MERFISH dataset has a larger size than the mouse visual cortex dataset but smaller optimal parameters (comparing the mouse visual cortex dataset with N = 1207, α = 14, to mouse hypothalamus dataset with N = 2693, α = 10) (**Supplementary Fig. S16b, h**). Despite the complex dependence of parameters on the data types, our heuristic two-stage screening approach works for all four diverse datasets and can hopefully be applied to other types of data. The default parameters for SpaSNE were set as* $\alpha = 10,\ \beta = 5$ *if spatial information is available, and* $\alpha = 5,\ \beta = 0$ *if the spatial information is not available. The ranges used in tough and fine screenings and the optimal parameters for the four datasets can be found in **Supplementary Table S1**.*

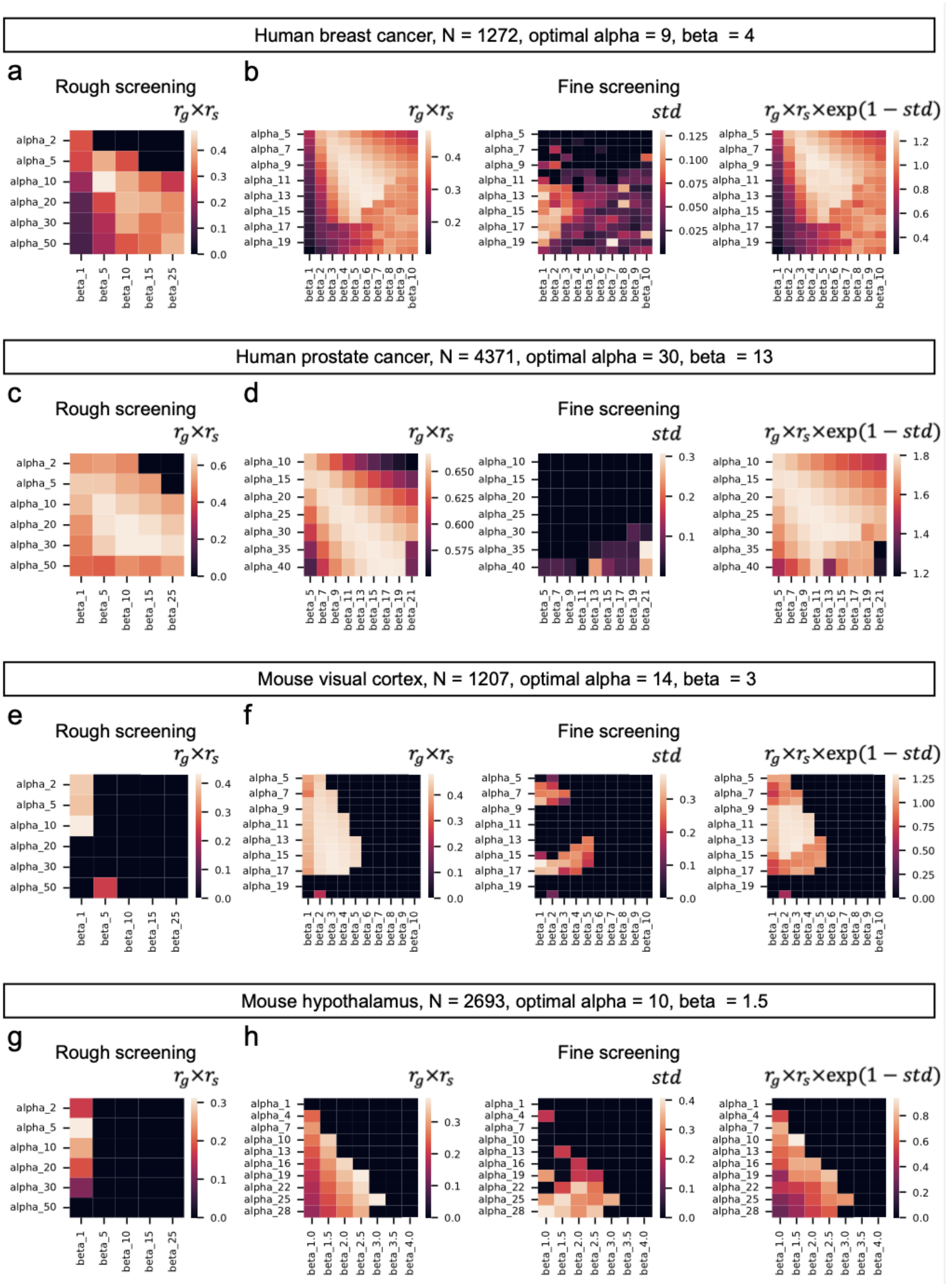
Human breast cancer, N = 1272, optimal alpha = 9, beta = 4
a
Rough screening
$r_g \times r_s$
b
Fine screening
$r_g \times r_s$
std
$r_g \times r_s \times \exp(1 - std)$
Human prostate cancer, N = 4371, optimal alpha = 30, beta = 13
c
Rough screening
$r_g \times r_s$
d
Fine screening
$r_g \times r_s$
std
$r_g \times r_s \times \exp(1 - std)$
Mouse visual cortex, N = 1207, optimal alpha = 14, beta = 3
e
Rough screening
$r_g \times r_s$
f
Fine screening
$r_g \times r_s$
std
$r_g \times r_s \times \exp(1 - std)$
Mouse hypothalamus, N = 2693, optimal alpha = 10, beta = 1.5
g
Rough screening
$r_g \times r_s$
h
Fine screening
$r_g \times r_s$
std
$r_g \times r_s \times \exp(1 - std)$

Supplementary Figure S16. Rough and fine screenings of parameters α and β of SpaSNE on the four datasets.

For each dataset, a rough screening was first performed to determine the range of optimal parameters on a large scale (a, c, e, g), then a fine screening was performed to find the optimal parameters with a fine resolution (b, d, f, h). In each embedding, setting $r_g = 0$ *if* $r_g \leq r_{thres}$*, where* r_{thres} *is the maximal* r_g *in 100 repeats of t-SNE embeddings. The heatmaps for rough tuning represent values of* $r_g \times r_s$ *with different combinations of parameters. The heatmaps for fine tuning represent values of* $r_g \times r_s$ *(left),* std *(middle) and* $r_g \times r_s \times exp\,(1 - std)$*(right). The* std *is the standard deviation of* r_g *in multiple repeats of SpaSNE embeddings in each parameter combination. The parameter ranges used in screening and optimal parameters for the four datasets can be found in* ***Supplementary Table S1****. (a-b) Rough (a) and fine (b) screening on human breast cancer data. (c-d) Rough (c) and fine (d) screening on human prostate cancer data. (e-f) Rough (e) and fine (f) screening on mouse visual cortex data. (g-h) Rough (g) and fine (h) screening on mouse hypothalamus data.*

Comment 2: Scalability
The authors acknowledge that the current implementation of SpaSNE may not be optimized for very large datasets. Given the rapid growth in spatial omics data, it would be valuable to discuss potential strategies for improving scalability or provide estimates of the current limitations in terms of dataset size.

Response: Thank you for highlighting the importance of scalability. We have expanded our discussion of SpaSNE's computational complexity and limitations. Currently, SpaSNE's global loss components require high computational resources for larger datasets. We are exploring approaches to approximate global loss terms, such as randomly selecting edges to reduce computational costs, similar to the SpaceFlow algorithm. These strategies could significantly improve SpaSNE's efficiency on large datasets. The revised discussion is provided at lines 289-303 on page 15.

Lines 289-303 on page 15:

The computational complexity of t-SNE (implemented by Barnes-Hut-SNE) is $O(NlogN)$*. The computational complexity of UMAP is empirically* $O(N^{1.14})$*. The computational cost of SpaSNE consists of three parts: the local loss of gene expression* L_t*, global loss of gene expression* L_g *and global loss of spatial positions* L_s *(Eq. 11). By applying the vantage-point trees approximation used in Barnes-Hut-SNE, the cost of* L_t *can be reduced from* $O(N^2)$ *to* $O(NlogN)$*. However, the global loss* L_g *and* L_s *cannot be approximated by the local-structure-based strategy in Barnes-Hut-SNE or the Nearest-Neighbor-Descent algorithm used in UMAP. Thus, the computational cost in the current form of SpaSNE is* $O(N^2)$*. The running time of SpaSNE on a MacBook Pro with a two GHz Quad-Core Intel Core i5 processer and 16 GB 3733 MHz LPDDR4X memory varies from 18 seconds for the human breast cancer dataset with 1272 spots, to 3 minutes for*

the human prostate cancer dataset with 4371 spots. One possible approach to reducing computational time for large dataset is to mimic the strategy in the SpaceFlow algorithm to use a fixed number of randomly selected edges to approximate the pairwise distance calculation in global terms $\hat{p}_{ij}$, $\hat{q}_{ij}$ *and* $\hat{s}_{ij}$ *(Eq. 6-9). In this way, the* $O(N^2)$ *in global loss will be constant and the total cost will become* $O(NlogN)$.

Comment 3: Comparison to other spatial-aware methods

While the comparison to t-SNE and UMAP is appropriate, and the authors claim SpaSNE is only a visualization tool with purposes similar to t-SNE or UMAP, it would be interesting to see how SpaSNE compares to other recently developed methods that also incorporate spatial information, such as SpatialPCA or graph-based approaches with embedding dimension=2. This comparison would provide a more comprehensive evaluation of SpaSNE's performance relative to state-of-the-art spatial-aware dimensionality reduction techniques.

Response: Thank you for this suggestion to include additional spatial-aware methods in our comparison. We have added a comparison of SpaSNE with SpatialPCA (embedding dimension set to 2) and observed that SpaSNE outperforms SpatialPCA (d = 2) in terms of embedding quality across all datasets, particularly in spatial and gene expression preservation. These new comparisons are shown in **Supplementary Figure S14** and are discussed in the revised manuscript at lines 585-591 on page 27.

Lines 585-591 on page 27:

*SpaSNE was designed as a visualization tool in two-dimensional space with similar purposes as t-SNE or UMAP, but not as a clustering tool for spatially resolved cell-type clustering tasks such as SpatialPCA. Thus, there is no direct comparison between SpaSNE and SpatialPCA. However, a comparison cannot still be made if setting the embedding dimension of SpatialPCA to be 2, and we showed that SpaSNE outperformed SpatialPCA (d = 2) in the quantitative evaluations on all the four datasets (**Supplementary Fig. S14a-h**).*

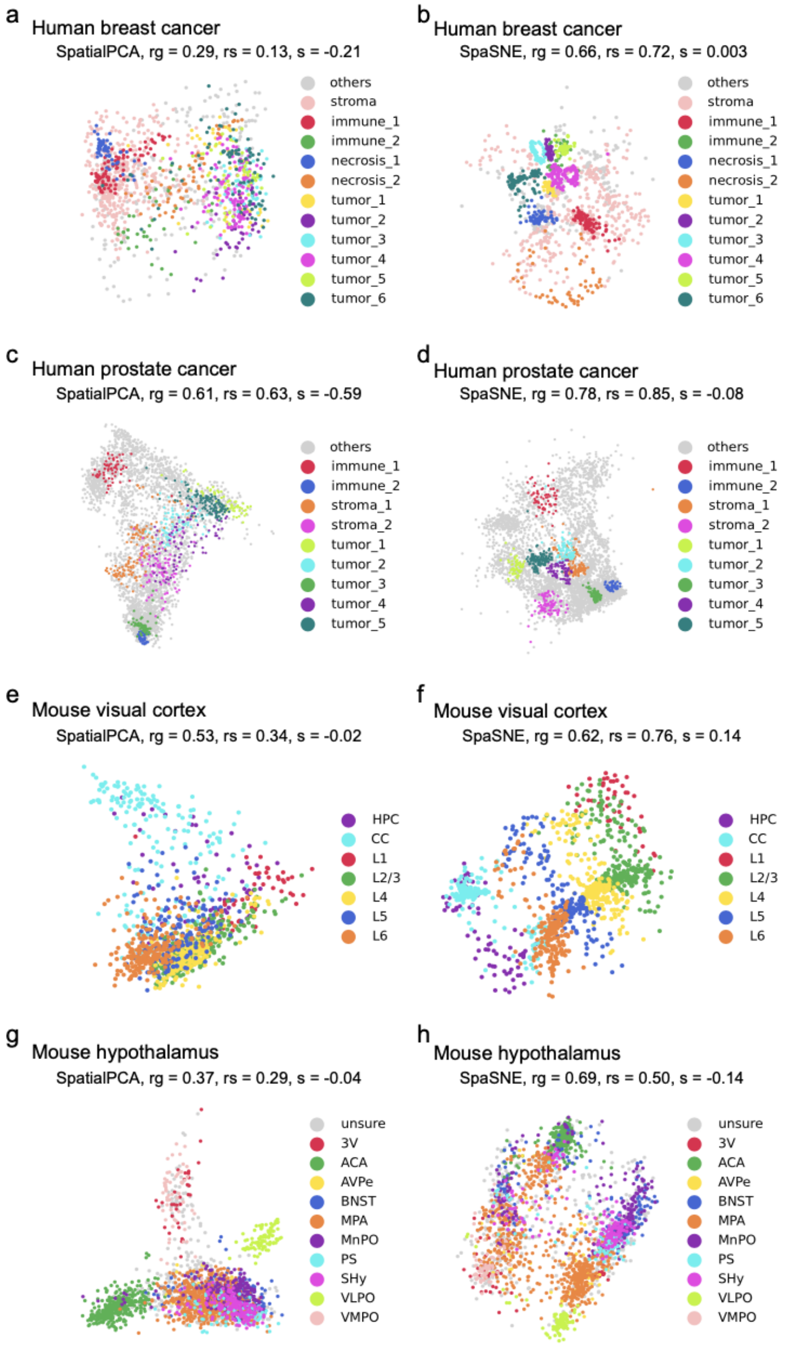

Supplementary Figure S14. Comparison of SpaSNE with SpatialPCA (d = 2) on visualization of the four datasets. *(a) SpatialPCA visualization on human breast cancer dataset with d = 2. (b) SpaSNE visualization on human breast cancer dataset. (c) SpatialPCA visualization on human prostate cancer dataset with d = 2. (d) SpaSNE visualization on human prostate cancer dataset. (e) SpatialPCA visualization on mouse visual cortex dataset with d = 2. (f) SpaSNE visualization on mouse visual cortex dataset. (g) SpatialPCA visualization on Mouse hypothalamus dataset with d = 2. (h) SpaSNE visualization on Mouse hypothalamus dataset.*

Comment 4: Robustness
The authors demonstrate SpaSNE's performance on four datasets. It would be beneficial to include a discussion on the robustness of the method across different tissue structures and experimental noise levels. Are there any situations where SpaSNE might not perform as well?

Response: We have added a discussion addressing SpaSNE's performance across various tissue types, platforms, and spatial resolutions. Specifically, SpaSNE performs well when spatial information correlates with phenotypic clustering, but its performance may be less optimal when this correlation is weak. For example, datasets at multicellular or subcellular resolution may present challenges if spatial data does not directly align with cell states. We outline potential future adaptations to address these limitations in the revised manuscript at lines 603-616 on page 28.

Lines 603-616 on page 28:

Fourthly, the design of SpaSNE assumes that the spatial information in spatially resolved transcriptomics data will contribute to the identification of cell states or the global organization of the cells. It may be less effective when there is no correlation between the phenotype and spatial positions of cells. In addition, its performance may be compromised in situations where spatial transcriptomics measurements are taken at multicellular resolution where a single spot contains multiple cell types, or in subcellular resolution where a single cell covers multiple spatial positions such as Visium HD data. For the latter case, we may adapt SpaSNE to analyze such datasets by incorporating current decomposition or aggregation methods for the preprocessing of data in the future. For the four datasets, we found that SpaSNE achieved better embedding quality for the human breast cancer dataset and human prostate cancer dataset generated from the 10x Visium platform, than the mouse visual cortex dataset from the STARmap platform and mouse hypothalamus dataset from the MERFISH platform. The reason might be that 10x Visium platform measures a larger number of genes than STARmap and MERFISH and therefore helps better define cell types.

Comment 5: Concern Regarding t-SNE Generation in SpaSNE Repository In the SpaSNE repository, the authors generate t-SNE results in two example notebooks using the following code: tsne_pos = spasne.run_spasne(df_data, alpha = 0.0, randseed = 5) This implementation raises a potential issue. While setting alpha = 0.0 aims to eliminate the spatial component, it's important to note that this still uses the default hyperparameter β=2.0, which incorporates spatial loss. As a result, the output is not a true t-SNE representation but rather a modified version that still considers spatial information.

Response: Thank you for this observation. In the SpaSNE algorithm, when the input of spatial positions is absent, β will be set to zeros by default. Thus, spasne.run_spasne(df_data, alpha =

0.0, randseed = 5) will not use spatial information. We have clarified this in the repository documentation to ensure users understand this default setting.

Comment 6: Recommendations:
1. Accuracy in Method Comparisons: It would be beneficial for the authors to review and adjust the settings used for compared methods, especially t-SNE, to ensure fair and accurate comparisons.
2. Documentation Clarity: The authors should clearly document the exact parameters and implementations used for each comparison method in their repository and associated papers to prevent misinterpretation of results.

Response: Thank you for these recommendations. We have ensured that the parameters used in SpaSNE, t-SNE, and UMAP comparisons are consistent across methods. All settings and parameters, including initialization, perplexity, and iteration limits, are now detailed in the manuscript. These details are included in the revised manuscript at lines 275-279 on page 14.

Lines 275-279 on page 14:

All three algorithms were initialized with default setting: UMAP was initialized using a spectral embedding of the fuzzy 1-skeleton, both t-SNE and SpaSNE were initialized from a truncated eigen-vector matrix with the dimension of 50. The stopping criterion for SpaSNE is the same as t-SNE which stops when the maximal iteration (1000 by default) is reached. The perplexity values in SpaSNE and t-SNE were set as the default value which is 50.

Comment 7: Method details
In the Methods section, more details on the implementation of SpaSNE would be helpful, such as computational complexity and runtime comparisons with t-SNE and UMAP.

Response: Thank you for this suggestion. We have added a discussion on SpaSNE's computational complexity relative to t-SNE and UMAP, noting that SpaSNE's global loss components increase computational costs, especially with larger datasets. We also included estimated runtime comparisons for each method on different dataset sizes to provide users with practical benchmarks. These details are in the revised manuscript at lines 289-303 on page 15.

Lines 289-303 on page 15:

The computational complexity of t-SNE (implemented by Barnes-Hut-SNE) is $O(NlogN)$. The computational complexity of UMAP is empirically $O(N^{1.14})$. The computational cost of SpaSNE consists of three parts: the local loss of gene expression L_t, global loss of gene expression L_g and global loss of spatial positions L_s (Eq. 11). By applying the vantage-point trees approximation used in Barnes-Hut-SNE, the cost of L_t can be reduced from $O(N^2)$ to $O(NlogN)$. However, the

global loss L_g and L_s cannot be approximated by the local-structure-based strategy in Barnes-Hut-SNE or the Nearest-Neighbor-Descent algorithm used in UMAP. Thus, the computational cost in the current form of SpaSNE is $O(N^2)$. The running time of SpaSNE on a MacBook Pro with a two GHz Quad-Core Intel Core i5 processer and 16 GB 3733 MHz LPDDR4X memory varies from 18 seconds for the human breast cancer dataset with 1272 spots, to 3 minutes for the human prostate cancer dataset with 4371 spots. One possible approach to reducing computational time for large dataset is to mimic the strategy in the SpaceFlow algorithm to use a fixed number of randomly selected edges to approximate the pairwise distance calculation in global terms $\hat{p}_{ij}$, $\hat{q}_{ij}$ and $\hat{s}_{ij}$ (Eq. 6-9). In this way, the $O(N^2)$ in global loss will be constant and the total cost will become $O(NlogN)$.

Comment 8: Availability
The authors mention that they are working on developing a plug-in for popular single-cell analysis platforms. It would be helpful to update on this effort in the manuscript.

Response: Thank you for this suggestion. We are actively working to enhance SpaSNE's speed and usability and anticipate beginning plug-in development in the near future.
