# Supplementary material for: Dimensionality reduction for visualizing spatially resolved profiling data using SpaSNE
Source: Gigascience. 2025 Feb 17;14:giaf002. doi: 10.1093/gigascience/giaf002 (PMC11831803; doi:10.1093/gigascience/giaf002)
Supplement: giaf002_Reviewer_1_Report_Original_Submission — Etienne Becht -- 6/3/2024 [file giaf002_reviewer_1_report_original_submission.pdf]

Reviewer Report

Title: Dimensionality reduction for visualizing spatially resolved profiling data using SpaSNE

Version: Original Submission **Date:** 6/3/2024

Reviewer name: Etienne Becht

Reviewer Comments to Author:

In this manuscript, Zhou et al. introduce SpaSNE, an algorithm that builds upon Barnes-Hut t-SNE and applicable to spatial data. t-SNE is a non-linear dimensionality reduction methods which finds a 2D representation of high-dimensional data that approximately preserves input neighborhoods. This is achieved by optmizing for a KL-divergence loss between probability distributions in the high- and low-dimensional spaces, which are computed from distances between pairwise data points in the respective spaces. In SpaSNE, the authors add two new terms to the loss function, in addition to the one already present in regular t-SNE: one term favors the preservation of "long" distances (so that points that are far apart remain far apart) and one on "spatial distances" (distances from the spatial coordinates). These two new terms are weighted by hyperparameters. The authors apply SpaSNE to a variety of spatial transcriptomics datasets (from Visium, STARmap and MERFISH), and compare their results to t-SNE and UMAP, the most commonly used non-linear dimensionality reduction methods in computational biology. Qualitative analyses and three metrics (Pearson correlation of embedding distances vs spatial coordinates, Pearson correlation of embedding distances versus gene expression distances and silhouette coefficients) are used to compare the three methods. The authors conclude that SpaSNE is better than t-SNE and UMAP in accuracy and embeddings meaningfulness. The manuscript is clearly written and easy to follow. I however have some concerns in the comprehensiveness and fairness of the comparisons that are presented :

Major points :

- One of the terms in SpaSNE's loss function aims at preserving spatial distances. It is not entirely clear to me what the goal of preserving spatial distances in embeddings is ? Spatial coordinates have low dimensionality (typically 2 in current omic experiments) and therefore do not need to be reduced. The authors show on a number of examples that "clusters" are better grouped in SpaSNE versus t-SNE and UMAP (as reflected by the Silhouette score), but it seems that these clusters are largely defined by spatial areas in the first place. I would like to recall that many current spatial transcriptomics are limited in their resolution to spots that aggregate multiple cells, which leads to crude phenotyping. Cells can be much more subtly phenotyped (in scRNAseq for instance), and it is likely that the next generation of spatial transcriptomics technologies, such as Visium HD, will have subcellular resolution. In that case, it seems that preserving spatial distances in the embeddings will be detrimental as it will compete with gene expression data.
- The two new terms in the loss function come at the cost of two extra hyperparameters which is a major disadvantage. The authors show very limited data on the sensitivity of their results to these hyperparameters - in figures S3 and S4. The values shown in S3 and S4 are however extreme, with one of the coefficients set to 0 (thus entirely ignoring the corresponding term in the loss function). I think

the authors should, for a fixed initialization, present embeddings obtained from hyperparameters spanning the whole range of recommended values ([6;15] for alpha, [1;7.5] for beta) across datasets. In their comparisons, the authors use t-SNE and UMAP with default parameters but adjust SpaSNE's hyperparameters for each dataset. This increases the risk of biased results. When investigating embeddings for different hyperparameters values, the same metrics could be computed and compared with those obtained from default UMAP and t-SNE. This will be a fairer summary of the method's performance.
- The authors mention that their recommended hyperparameter values provide the best performance in terms of reproducibility of the embeddings (among other things) but nowhere in the manuscript is this reproducibility aspect discussed - how did the authors mention reproducibility and how is it affected by hyperparameter values ?
- The s^{hat}_{ij} (Equation 9) terms are defined from a (normalized) Cauchy / Student t-distribution of the squared euclidean distance. The squared euclidean distance is not bounded and the s^{hat}_{ij} will therefore be heavily influenced by the most distant pairs of data points. I therefore question whether this is a sensible loss function to preserve "long" distances as it will probably only preserve "farthest" distances, which may not be typically interesting ? This intuition seems confirmed based on supplementary figure S3c (and to a lesser extent S4c) where a large number of singleton outliers seem present with alpha = 10 and beta = 0.
- Some important details are missing from the methods :
+ How are each method initialized ?
+ What's the stopping criterion in SpaSNE ?
+ How were cell / spots assigned phenotyped in each dataset ?
+ What preprocessing steps were applied to each dataset ?
- The authors mention that in the breast cancer dataset, 1272 spots were selected. How and why were these spots selected ?
- The metrics (r_g, r_s and s) that the authors use are in my opinion problematic : r_g and r_s measure the conservation of the expression and spatial distances, and therefore mirror the loss function terms. I think that a metric sensitive to local neighborhood preservation (e.g. k nearest neighbors retention) should also be shown for the three methods. The silhouette coefficient is also problematic : it measures how "tight" a spot/cell cluster is in the embeddings. It however appears that in most (all) of the datasets used, clusters are - at least partially - defined by spatial characteristics. Again, this is a current technological limitation of omic technologies but not something that seems desirable - clustering cells by phenotype seem more appealing. Sadly, the only datasets with (sub)cellular resolution shown (the MERFISH and STARmap datasets) are from brain regions where phenotype and spatial coordinates are heavily correlated (along a one-dimensional axis for the STARmap dataset and in 2D areas for the MERFISH dataset). This would not be the case in most organs when analyzing a sizable piece of tissue : the authors for instance had to exclude half of the hypothalamus dataset for that reason.
Minor points :
- For clarity and completeness, it would be nice to define sigma_i in equation (1) for readers that are not very familiar with t-SNE.
- In the breast cancer dataset, there is a class of spots phenotyped as "others". Based on available classes shown in the figure, does it correspond to "fat" in supplementary figure 1 ? If that is the case,

why is it treated differently ? Are cells phenotyped as "others" used when computing the various metrics ?
- It would be nice to exhaustively display cell / spot phenotypes in supplementary data for each dataset for comprehensiveness. In figure 3, "others" appear to represent the majority of the spots but all spots are phenotyped in Figure S1b.
- The authors mention that spatial proteomics technologies are "under development" but there are many commercial platform offering spatial protein expression information - albeit with a targeted design.
Etienne Becht

Methods

Are the methods appropriate to the aims of the study, are they well described, and are necessary controls included? Choose an item.

Conclusions

Are the conclusions adequately supported by the data shown? Choose an item.

Reporting Standards

Does the manuscript adhere to the journal's guidelines on minimum standards of reporting? Choose an item.

Choose an item.

Statistics

Are you able to assess all statistics in the manuscript, including the appropriateness of statistical tests used? Choose an item.

Quality of Written English

Please indicate the quality of language in the manuscript: Choose an item.

Declaration of Competing Interests

Please complete a declaration of competing interests, considering the following questions:

- Have you in the past five years received reimbursements, fees, funding, or salary from an organisation that may in any way gain or lose financially from the publication of this manuscript, either now or in the future?
- Do you hold any stocks or shares in an organisation that may in any way gain or lose financially from the publication of this manuscript, either now or in the future?

- Do you hold or are you currently applying for any patents relating to the content of the manuscript?
- Have you received reimbursements, fees, funding, or salary from an organization that holds or has applied for patents relating to the content of the manuscript?
- Do you have any other financial competing interests?
- Do you have any non-financial competing interests in relation to this paper?

If you can answer no to all of the above, write 'I declare that I have no competing interests' below. If your reply is yes to any, please give details below.

I declare that I have no competing interests

I agree to the open peer review policy of the journal. I understand that my name will be included on my report to the authors and, if the manuscript is accepted for publication, my named report including any attachments I upload will be posted on the website along with the authors' responses. I agree for my report to be made available under an Open Access Creative Commons CC-BY license (http://creativecommons.org/licenses/by/4.0/). I understand that any comments which I do not wish to be included in my named report can be included as confidential comments to the editors, which will not be published.

Choose an item.

To further support our reviewers, we have joined with Publons, where you can gain additional credit to further highlight your hard work (see: https://publons.com/journal/530/gigascience). On publication of this paper, your review will be automatically added to Publons, you can then choose whether or not to claim your Publons credit. I understand this statement.

Yes Choose an item.
