# Supplementary material for: Dimensionality reduction for visualizing spatially resolved profiling data using SpaSNE
Source: Gigascience. 2025 Feb 17;14:giaf002. doi: 10.1093/gigascience/giaf002 (PMC11831803; doi:10.1093/gigascience/giaf002)
Supplement: giaf002_Reviewer_2_Report_Original_Submission — Raffaele A Calogero, B.Sc. -- 8/5/2024 [file giaf002_reviewer_2_report_original_submission.pdf]

Reviewer Report

Title: Dimensionality reduction for visualizing spatially resolved profiling data using SpaSNE

Version: Original Submission **Date:** 8/5/2024

Reviewer name: Raffaele A Calogero, B.Sc.

Reviewer Comments to Author:

The overall concept of the paper is pretty interesting. I also managed to install and run the examples provided by the authors in their github on my MAC M1 Max without any issue, unless the need to install scanpy.
However, how it is structurated the github it does not provide a sufficiently user-friendly tool.
Major:
1) The developers should offer a Docker container, accessible via JupyterLab, which is more user-friendly than Jupyter Notebook, in addition to the GitHub repository. This will mitigate the uncertainty of compiling the tool on different hardware and improve reproducibility. Additionally, SpaSNE should be accompanied by a suite of functions to simplify its usage and facilitate the testing of different parameters.
2) More information on the preparation and data format of metadata, such as _labels.csv, must be provided. Additionally, validation functions to test the compatibility of user-created metadata should be included.
3) The authors noted that "the current SpaSNE package has not been optimized for handling datasets with a large number of cells." However, the limitations of the current implementation are unclear. Therefore, the performance of SpaSNE as the number of cells and the size of metadata increase should be evaluated and reported in the paper. Authors must consider that a tool effective on only a few hundreds of cells is practically useless given the current rate of increase in cell numbers in single-cell experiments.
4) Since the conclusions suggest that SpaSNE could serve as a solid foundation for various subsequent analytical procedures (such as trajectory analysis, and pseudotime analysis), examples of these applications should also be provided.
5) The authors stated that "SpaSNE offers tunable parameters to adjust the users' requests on the preservation of molecular or spatial information." Therefore, a detailed section on GitHub should be dedicated to thoroughly describing the effects of these tunable parameters.
6) At lane 194 it is stated " we screened the optimal combination of parameters alpha and beta that gave the best performances in terms of the gene expression preservation ..." more details must be provided on what the author mean by "we screened the optimal combination of parameters ..."
7) In the section "Quantitative Evaluation of the Embedding Quality," Pearson correlation is used. The authors must justify why the Pearson correlation coefficient is the best method for measuring embedding quality.
8) As far as I remember, perplexity is a critical parameter in t-SNE. Could the authors provide additional information about the role of perplexity in SpaSNE?

Methods

Are the methods appropriate to the aims of the study, are they well described, and are necessary controls included? Choose an item.

Conclusions

Are the conclusions adequately supported by the data shown? Choose an item.

Reporting Standards

Does the manuscript adhere to the journal's guidelines on [minimum standards of reporting?] Choose an item.

Choose an item.

Statistics

Are you able to assess all statistics in the manuscript, including the appropriateness of statistical tests used? Choose an item.

Quality of Written English

Please indicate the quality of language in the manuscript: Choose an item.

Declaration of Competing Interests

Please complete a declaration of competing interests, considering the following questions:

- Have you in the past five years received reimbursements, fees, funding, or salary from an organisation that may in any way gain or lose financially from the publication of this manuscript, either now or in the future?
- Do you hold any stocks or shares in an organisation that may in any way gain or lose financially from the publication of this manuscript, either now or in the future?
- Do you hold or are you currently applying for any patents relating to the content of the manuscript?
- Have you received reimbursements, fees, funding, or salary from an organization that holds or has applied for patents relating to the content of the manuscript?
- Do you have any other financial competing interests?
- Do you have any non-financial competing interests in relation to this paper?

If you can answer no to all of the above, write 'I declare that I have no competing interests' below. If your reply is yes to any, please give details below.

'I declare that I have no competing interests'

I agree to the open peer review policy of the journal. I understand that my name will be included on my report to the authors and, if the manuscript is accepted for publication, my named report including any attachments I upload will be posted on the website along with the authors' responses. I agree for my report to be made available under an Open Access Creative Commons CC-BY license (http://creativecommons.org/licenses/by/4.0/). I understand that any comments which I do not wish to be included in my named report can be included as confidential comments to the editors, which will not be published.

Choose an item.

To further support our reviewers, we have joined with Publons, where you can gain additional credit to further highlight your hard work (see: https://publons.com/journal/530/gigascience). On publication of this paper, your review will be automatically added to Publons, you can then choose whether or not to claim your Publons credit. I understand this statement.

Yes Choose an item.
