# Supplementary material for: Dimensionality reduction for visualizing spatially resolved profiling data using SpaSNE
Source: Gigascience. 2025 Feb 17;14:giaf002. doi: 10.1093/gigascience/giaf002 (PMC11831803; doi:10.1093/gigascience/giaf002)
Supplement: giaf002_Reviewer_3_Report_Original_Submission — Zhixiang Lin -- 8/6/2024 [file giaf002_reviewer_3_report_original_submission.pdf]

Reviewer Report

Title: Dimensionality reduction for visualizing spatially resolved profiling data using SpaSNE

Version: Original Submission **Date:** 8/6/2024

Reviewer name: Zhixiang Lin

Reviewer Comments to Author:

This manuscript introduces SpaSNE (Spatially resolved t-SNE), a dimensionality reduction and visualization method designed specifically for spatially resolved profiling data. The authors adapt the t-SNE algorithm to incorporate both gene expression and spatial information, addressing a gap in current analysis tools for spatial omics data. They demonstrate SpaSNE's performance on four datasets from three different experimental platforms (Visium, STARmap, and MERFISH), covering both diseased and normal tissues. The results show that SpaSNE outperforms methods like t-SNE and UMAP in preserving both molecular and spatial data structures, leading to more accurate and meaningful visualizations.
While the study addresses an important need in the rapidly growing field of spatial omics, there are several areas that require further development and clarification. The authors provide a comparative evaluation of SpaSNE's performance against existing methods, but the parameter selection process lacks comprehensive guidance, and the scalability of the method for larger datasets is not adequately addressed. Additionally, the comparison with other spatial-aware methods is limited, and the robustness of SpaSNE across various experimental conditions needs further discussion.
Overall, this work represents a potential contribution to the field of spatial omics data analysis. However, several key issues need to be addressed to fully demonstrate the method's utility and reliability. These include providing clearer guidelines for parameter selection, addressing scalability concerns, expanding comparisons with other spatial-aware methods, and thoroughly discussing the method's robustness. Addressing these points would strengthen the manuscript and better establish SpaSNE's potential as a valuable tool for researchers working with spatially resolved profiling data.
Major Comments
Parameter selection
The authors mention that SpaSNE has tunable parameters (Î± and Î²) to adjust the balance between molecular and spatial information preservation. They provide a recommended range for setting Î± and Î² based on their experience with four datasets. However, this approach has limitations:
1. The trade-off between L_t,L_g, and L_s should consider the scales of gene expression and spatial location data, which may vary across different datasets and experimental platforms.
2. Even if the recommended range may be suitable for many spatially resolved profiling datasets, the authors do not provide a heuristic strategy for selecting optimal hyperparameters within this range.
3. There is no discussion of how these parameters might need to be adjusted for datasets with significantly different characteristics or scales.
It would be highly beneficial to provide more comprehensive guidance on how users should select these parameters for their specific datasets. This could include:
1. A more detailed explanation of how the scales of gene expression and spatial data might influence

parameter selection.
2. A step-by-step heuristic approach for parameter tuning based on dataset characteristics.
3. Discussion of any rules of thumb that users could apply.
4. Exploration of potential automated methods for parameter optimization, such as grid search with cross-validation or Bayesian optimization.

Additionally, the authors should consider providing a small benchmark dataset and a tutorial demonstrating the parameter selection process. This would greatly enhance the usability of SpaSNE for researchers new to the method.

Scalability

The authors acknowledge that the current implementation of SpaSNE may not be optimized for very large datasets. Given the rapid growth in spatial omics data, it would be valuable to discuss potential strategies for improving scalability or provide estimates of the current limitations in terms of dataset size.

Comparison to other spatial-aware methods

While the comparison to t-SNE and UMAP is appropriate, and the authors claim SpaSNE is only a visualization tool with purposes similar to t-SNE or UMAP, it would be interesting to see how SpaSNE compares to other recently developed methods that also incorporate spatial information, such as SpatialPCA or graph-based approaches with embedding dimension=2. This comparison would provide a more comprehensive evaluation of SpaSNE's performance relative to state-of-the-art spatial-aware dimensionality reduction techniques.

Robustness

The authors demonstrate SpaSNE's performance on four datasets. It would be beneficial to include a discussion on the robustness of the method across different tissue structures and experimental noise levels. Are there any situations where SpaSNE might not perform as well?

Minor Comments

Concern Regarding t-SNE Generation in SpaSNE Repository

In the SpaSNE repository, the authors generate t-SNE results in two example notebooks using the following code:

```
tsne_pos = spasne.run_spasne(df_data, alpha = 0.0, randseed = 5)
```

This implementation raises a potential issue. While setting alpha = 0.0 aims to eliminate the spatial component, it's important to note that this still uses the default hyperparameter Î²=2.0, which incorporates spatial loss. As a result, the output is not a true t-SNE representation but rather a modified version that still considers spatial information.

Recommendations:
1. Accuracy in Method Comparisons: It would be beneficial for the authors to review and adjust the settings used for compared methods, especially t-SNE, to ensure fair and accurate comparisons.
2. Documentation Clarity: The authors should clearly document the exact parameters and implementations used for each comparison method in their repository and associated papers to prevent misinterpretation of results.

Method details

In the Methods section, more details on the implementation of SpaSNE would be helpful, such as computational complexity and runtime comparisons with t-SNE and UMAP.

Availability
The authors mention that they are working on developing a plug-in for popular single-cell analysis platforms. It would be helpful to update on this effort in the manuscript.

Methods

Are the methods appropriate to the aims of the study, are they well described, and are necessary controls included? Choose an item.

Conclusions

Are the conclusions adequately supported by the data shown? Choose an item.

Reporting Standards

Does the manuscript adhere to the journal's guidelines on minimum standards of reporting? Choose an item.

Choose an item.

Statistics

Are you able to assess all statistics in the manuscript, including the appropriateness of statistical tests used? Choose an item.

Quality of Written English

Please indicate the quality of language in the manuscript: Choose an item.

Declaration of Competing Interests

Please complete a declaration of competing interests, considering the following questions:

- Have you in the past five years received reimbursements, fees, funding, or salary from an organisation that may in any way gain or lose financially from the publication of this manuscript, either now or in the future?
- Do you hold any stocks or shares in an organisation that may in any way gain or lose financially from the publication of this manuscript, either now or in the future?
- Do you hold or are you currently applying for any patents relating to the content of the manuscript?
- Have you received reimbursements, fees, funding, or salary from an organization that holds or has applied for patents relating to the content of the manuscript?
- Do you have any other financial competing interests?
- Do you have any non-financial competing interests in relation to this paper?

If you can answer no to all of the above, write 'I declare that I have no competing interests' below. If your reply is yes to any, please give details below.

I declare that I have no competing interests

I agree to the open peer review policy of the journal. I understand that my name will be included on my report to the authors and, if the manuscript is accepted for publication, my named report including any attachments I upload will be posted on the website along with the authors' responses. I agree for my report to be made available under an Open Access Creative Commons CC-BY license (http://creativecommons.org/licenses/by/4.0/). I understand that any comments which I do not wish to be included in my named report can be included as confidential comments to the editors, which will not be published.

Choose an item.

To further support our reviewers, we have joined with Publons, where you can gain additional credit to further highlight your hard work (see: https://publons.com/journal/530/gigascience). On publication of this paper, your review will be automatically added to Publons, you can then choose whether or not to claim your Publons credit. I understand this statement.

Yes Choose an item.
