# Supplementary material for: Dimensionality reduction for visualizing spatially resolved profiling data using SpaSNE
Source: Gigascience. 2025 Feb 17;14:giaf002. doi: 10.1093/gigascience/giaf002 (PMC11831803; doi:10.1093/gigascience/giaf002)
Supplement: giaf002_Authors_Response_To_Reviewer_Comments [file giaf002_authors_response_to_reviewer_comments.pdf]

Author's Response To Reviewer Comments

We sincerely thank the editor and the three reviewers for the opportunity to revise our work. In response to the feedback, we have substantially revised the manuscript and added 12 new supplementary figures. Major updates include demonstrating the necessity of preserving spatial information using SpaSNE, adding new biological analyses on the SpaSNE embedding results, and developing an automatic strategy for screening the two key parameters of SpaSNE. The reviewers' insightful comments have significantly enhanced the quality of our paper, and we hope the revised version is now suitable for publication in GigaScience. By uploading a PDF file named "Point-by-point response.pdf", we have addressed each point the editor and reviewers raised, referencing the relevant sections, figures, and tables in the revised paper and supplementary materials. All changes in the manuscript have been highlighted for clarity.
